# Kinetic Resolution of BINAMs by Stereoselective Copper-Catalyzed Dehydrogenative Si–N Coupling with Prochiral Dihydrosilanes

**DOI:** 10.1021/acs.orglett.5c02258

**Published:** 2025-07-01

**Authors:** Finn H. Gattwinkel, Martin Oestreich

**Affiliations:** Institut für Chemie, Technische Universität Berlin, Strasse des 17. Juni 115, 10623 Berlin, Germany

## Abstract

A nonenzymatic kinetic resolution of monoprotected 1,1′-binaphthyl-2,2′-diamine (BINAM) derivatives is reported. This is achieved by a Cu–H-catalyzed dehydrogenative Si–N coupling with prochiral dihydrosilanes using (*R*,*R*)-Ph-BPE as a chiral ligand. The atroposelective as well as diastereoselective *N*-silylation enables the resolution of BINAMs with various substituents in 6,6′- or 7,7′-positions with good to synthetically useful selectivity factors.

## Graphical abstract

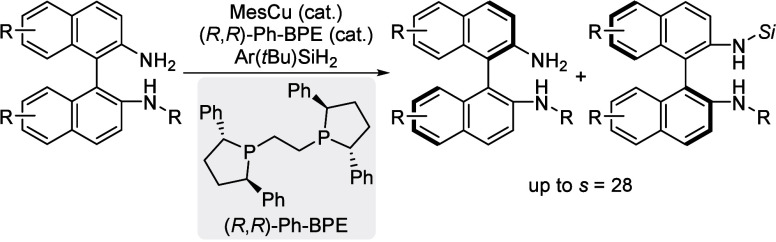

Since the first example over 20 years ago,^1^ the asymmetric silylation of alcohols has enjoyed great success, being utilized for the (dynamic) kinetic resolution of alcohols,^2–11^ desymmetrization of diols,^2–5,12,13^ and the synthesis of silicon compounds with silicon-centered chirality.^14–21^ However, despite many known methods for the silylation of amines,^22–30^ enantioselective variants have so far been limited to the synthesis of silicon-stereogenic silazanes first reported by Guan and Li^31,32^ and, more recently, the rhodium-catalyzed enantioselective desymmetrization of prochiral dihydrosilanes developed by He and co-workers (Scheme 1A).^33–35^ With our continued interest in the kinetic resolution of alcohols by Cu–H-catalyzed, stereoselective Si–O coupling with hydrosilanes^4,36–38^ we became interested in achieving a related resolution of amines by dehydrogenative Si–N coupling. As silylated aniline derivatives are reported to be chemically more stable than their aliphatic counterparts^33^ and encouraged by our recent success in the kinetic resolution of BINOLs and biphenols,^9^ we planned to investigate the kinetic resolution of 1,1′-binaphthyl-2,2′-diamine (BINAM) derivatives. Established approaches for the kinetic resolution of BINAMs include an asymmetric reductive amination that was developed by Tan and co-workers,^39^ a triazane formation with azodicarboxylates reported by Yang and co-workers,^40^ and a protocol using acylation that was recently disclosed by Akiyama and co-workers (Scheme 1B).^41–50^ In this work, we present the kinetic resolution of BINAMs by an enantioselective Cu–H-catalyzed, dehydrogenative Si–N coupling with dihydrosilanes (Scheme 1C). To the best of our knowledge, this work represents the first kinetic resolution of an aniline derivative by silylation.

**Scheme 1:**
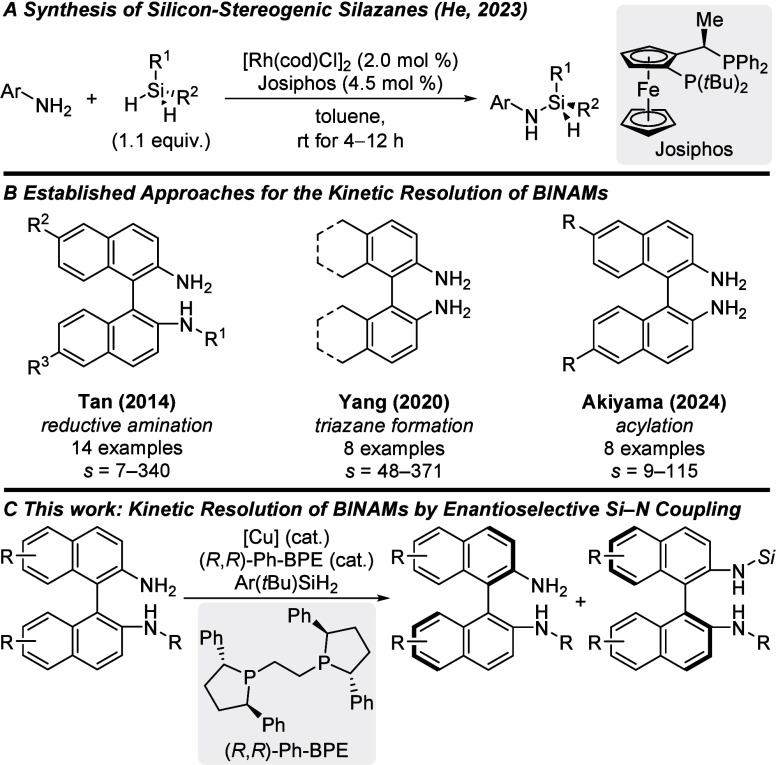
Established Approaches for Asymmetric Si–N Coupling, the Kinetic Resolution of BINAMs, and the Planned Merger of Both

As reports on the Cu–H-catalyzed coupling of amines and hydrosilanes are scarce,^33,51^ we began by exploring the ability of our commonly utilized catalyst system, that is, CuCl/NaO*t*Bu/(*R*,*R*)-Ph-BPE, to catalyze the dehydrogenative Si–N coupling (Table 1). As part of their study (cf. Scheme 1A), He and co-workers had already described the successful coupling of aniline (**1a**) and *tert*-butyl­(phenyl)­dihydrosilane (**2a**) under copper catalysis,^33^ and we decided to make this our starting point. Full conversion was obtained under routine reaction conditions (entry 1). In turn, when 2-aminobiphenyl (**1b**) was used as an approximation of steric congestion encountered in BINAM, no reaction was observed (entry 2). We then examined different copper sources as a possible solution. Using CuCN led to diminished conversion in the reaction with aniline (**1a**) (entry 3), but we were able to observe marginal conversion when using **1b** (entry 4). The conversion further increased when (MeCN)_4_CuPF_6_ was used (entries 5 and 6). In our previous work, we had employed mesitylcopper as a basic copper precatalyst,^52^ and MesCu also facilitates direct metalation of amines without prior activation by the hydrosilane.^53^ We were pleased to find that the use of mesitylcopper not only allowed our reference reaction to proceed cleanly (entry 7) but also gave the best result for silylated 2-aminobiphenyl **3b** (entry 8).

**Table 1:**
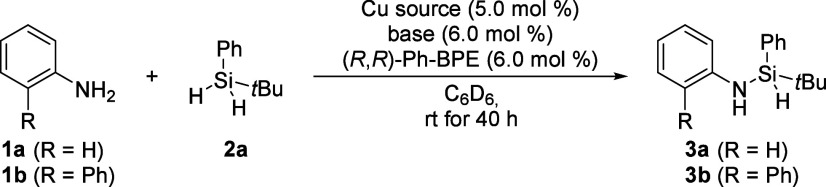
Screening of Suitable Copper Precatalysts^a^

entry	R	copper source	base	conv (%)^b^
1	H (**1a**)	CuCl	NaOtBu	>95
2	Ph (**1b**)	CuCl	NaOtBu	n.r.
3	H (**1a**)	CuCN	NaOtBu	23
4	Ph (**1b**)	CuCN	NaOtBu	8
5	H (**1a**)	(MeCN)_4_CuPF_6_	NaOtBu	71
6	Ph (**1b**)	(MeCN)_4_CuPF_6_	NaOtBu	29
7^c^	H (**1a**)	MesCu		>95
8^c^	Ph (**1b**)	MesCu		40

a Reactions were performed on a 0.2 mmol scale.

b Conversion was monitored by ^1^H NMR spectroscopy.

c Reaction time was 70 h. n.r. = no reaction. Mes = mesityl.

With a suitable catalyst system in hand, we then started looking at binaphthyldiamines using dihydrosilane **2a** (Table 2). Racemic BINAM (*rac*-**4a**) did not react under the standard conditions (entry 1). The *N*,*N*-dimethyl-substituted derivative *rac*-**4b** showed poor reactivity and selectivity (entry 2), so we continued with *N*-monoprotected BINAMs. The *N*-methylated BINAM *rac*-**4c** showed good reactivity and moderate selectivity with *s* = 5.2 (entry 3). Increasing the steric demand using an isopropyl group as in *rac*-**4d** did not improve the selectivity significantly (entry 3). A better selectivity factor of *s* = 8 was observed with the benzyl-substituted substrate *rac*-**4e** (entry 5). Consequently, we increased the steric hindrance further by installing a benzhydryl group as in *rac*-**4f**; this measure improved the selectivity considerably to *s* = 15 along with a tolerable decrease in the reaction rate. Conversely, installation of a trityl group as in *rac*-**4g** resulted in a complete loss of reactivity (entry 7). That selectivity did not extend to NOBIN analogue *rac*-**4h** as it decreased to *s* = 6 (entry 8). In addition to the enantiodifferentiation, we also observed moderate to high diastereoselectivity for formed silylamines **5**, demonstrating that desymmetrization of dihydrosilane **2a** takes place concomitantly. Generally, a higher diastereoselectivity also meant a higher selectivity factor. Unfortunately, the silylamines **5** were not stable toward air and moisture after purification. For this reason, we decided to cleave the silyl group directly after purification, thereby foregoing a deeper analysis of stereoselectivity of the desymmetrization at this stage.

**Table 2:**
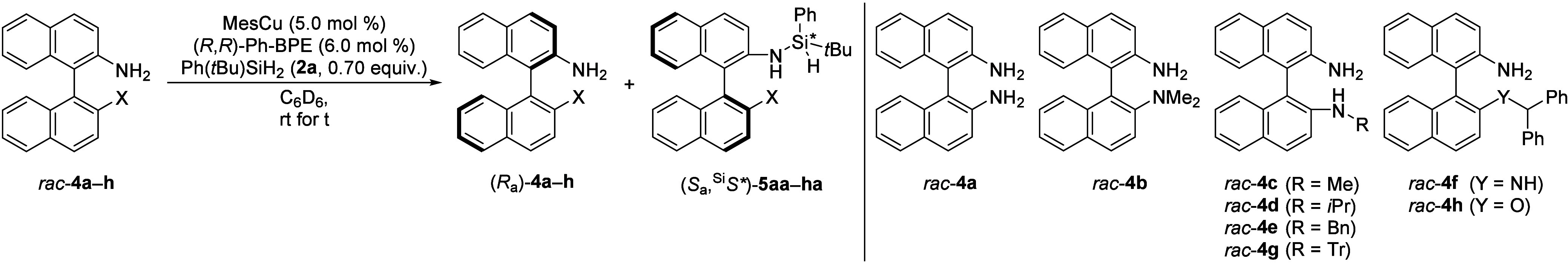
Evaluation of the *N*-Protecting Group^a^ ^,^

entry	X	*t*	conv (%)^b^	*ee* of (*R*_a_)-**4** (%)^c^	*ee* of (*S*_a_)-**4** (%)^d^	d.r. of **5** ^e^	*s* ^f^
1	NH_2_ (**4a**)	48 h	n.r.	–	–	–	–
2	NMe_2_ (**4b**)	13 days	40	22	30	64:26	2
3	NHMe (**4c**)	60 h	50	52	52	77:23	5
4	NH*i*Pr (**4d**)	60 h	58	68	48	75:25	6
5	NHBn (**4e**)	40 h	48	60	64	83:17	8
6^h^	NH[(CH)Ph_2_] (**4f**)	4 days	41	55	79	91:9	15
7	NHTr (**4g**)	48 h	n.r.	–	–	–	–
8	O[(CH)Ph_2_] (**4h**)	6 days	42	42	59	n.d.	6

a Reactions were performed on a 0.2 mmol scale. The configuration at the silicon atom was not determined.

b Conversion was monitored by ^1^H NMR spectroscopy and calculated according to conversion = *ee*_unreacted amine_/(*ee*_unreacted amine_ + *ee*_silylamine_) × 100.

c Determined by HPLC analysis on chiral stationary phases.

d Determined by HPLC analysis on chiral stationary phases after deprotection of the silylated aniline group.

e Determined by ^1^H NMR spectroscopic analysis.

f *s* = ln­[(1 – *C*)­(1 – *ee*)]/ln­[(1 – *C*)­(1 + *ee*)], where *ee* = *ee*_unreacted amine_/100 and *C* = conversion/100.

g Reaction run in CD_2_Cl_2._

h Reaction run in toluene. n.r. = no reaction. n.d. = not determined.

Having established the benzhydryl moiety as a suitable protecting group, we proceeded with the identification of optimal dihydrosilane **2** (Table 3). Monohydrosilanes were generally too unreactive. The *tert*-butyl group was vital to the chemical stability of the silylated aniline moiety, and even its replacement by a cyclohexyl group made purification by chromatography on either silica gel or alumina of different activity essentially impossible (not shown; see the Supporting Information). This led us to a variation of the aryl group starting in the 4-position. Simple substituents such as methyl (**2b**, entry 2) or *tert*-butyl (**2c**, entry 3) did not show any significant effect. Electronic modifications as in 4-methoxy-substituted **2d** or 4-trifluoromethyl-substituted **2e** brought about slightly diminished selectivities (entries 4 and 5). Single methyl substitution in the *meta*-position as in **2e** (entry 6) and naphth-2-yl derivative **2f** (entry 7) gave the same result as that of model silane **2a**. The use of a 3,5-xylyl group as in **2h** increased the selectivity factor to 19 at the expense of the reaction rate (entry 8). Previous studies employing monohydrosilanes had shown that *ortho*-substitution inhibits the reaction,^37^ but when using *tert*-butyl­(2-tolyl)­dihydrosilane (**2i**), we observed a substantial improvement in enantiodifferentiation (*s* = 24), now reaching a synthetically useful *s* value (entry 9). A similar trend was observed for the naphth-1-yl-substituted dihydrosilane **2j** (*s* = 21, entry 10). The increased steric demand also accounts for these rather slow reactions. Additional steric congestion by bis-*ortho*-substitution as in **2k** resulted in a complete loss of reactivity (entry 11).

**Table 3:**
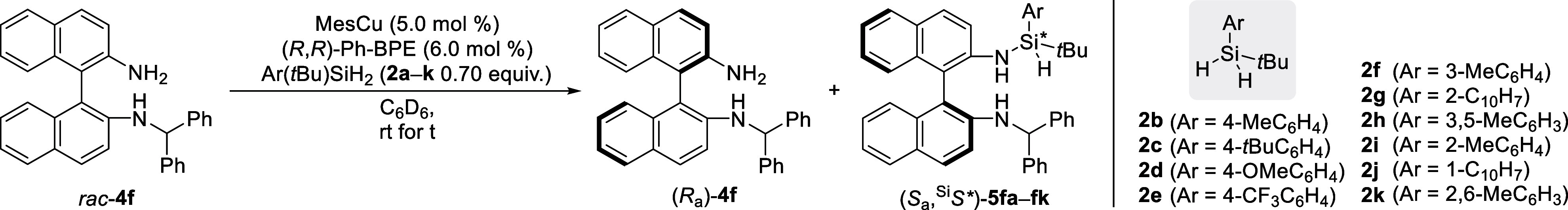
Identification of the Dihydrosilane^a^

entry	Ar	*t*	conv (%)	*ee* of (*R*_a_)-**4f** (%)	*ee* of (*S*_a_)-**4f** (%)	d.r. of **5fa**–**fk**	*s*
1^b^	Ph (**2a**)	4 days	41	55	79	91:9	15
2	4-MeC_6_H_4_ (**2b**)	6 days	51	76	73	88:12	14
3	4-*t*BuC_6_H_4_ (**2c**)	8 days	57	90	68	87:13	16
4	4-OMeC_6_H_4_ (**2d**)	6 days	49	70	72	87:13	13
5	4-CF_3_C_6_H_4_ (**2e**)	5 days	46	62	73	83:17	12
6	3-MeC_6_H_4_ (**2f**)	6 days	50	76	75	90:10	16
7	2-C_10_H_7_ (**2g**)	4 days	44	62	78	89:11	15
8	3,5-MeC_6_H_3_ (**2h**)	8 days	50	78	78	90:10	19
9	2-MeC_6_H_4_ (**2i**)	7 days	36	50	87	96:4	24
10	1-C_10_H_7_ (**2j**)	7 days	42	60	84	93:7	21
11	2,6-MeC_6_H_3_ (**2k**)	4 days	n.r.	–	–	–	–

a For details see footnotes of Table 2.

b Reaction run in toluene.

We decided to proceed with an assessment of the scope of the reaction, first aiming to probe the reactivity using parent dihydrosilane **2a** and then verifying the enhancement of the selectivity with *ortho*-tolyl-substituted congener **2i** (Scheme 2). We began by looking at different substituents in the 6,6′-positions of the binaphthyl backbone starting with different halogen atoms. A fluorine substituent (**4i**) led to a moderately decelerated reaction rate with a slightly lower selectivity (*s* = 13). Both the dichloro- (**4j**) and dibromo-substituted (**4k**) substrates showed improved enantiodifferentiation, and synthetically useful selectivity values were obtained (*s* = 20 for **4j** and *s* = 21 for **4k**). We then moved on to evaluate alkyl and aryl substituents. 6,6′-Dimethylated **4l** showed little improvement (*s* = 17) and comparable reactivity. Increasing the steric hindrance with an isopropyl (**4m**) or phenyl (**4n**) group decreased the reaction rate significantly, but, while selectivity was lower for the isopropyl group (*s* = 10), it was higher for the phenyl group (*s* = 23). Trimethylsilyl substituents (**4o**) at the 6,6′-positions were tolerated well, giving a result (*s* = 16) similar to that of the parent compound **4f**, albeit with a decreased reaction rate. Electron-donating methoxy groups (**4p**) markedly accelerated the reaction with good selectivity (*s* = 15). Of note, we could not access substrates with electron-withdrawing substituents in these positions. We also evaluated substrates **4k**, **4l**, and **4p** with more hindered dihydrosilane **2i**. Only substrate **4l** showed a sufficient level of reactivity, and we were able to obtain an increased selectivity factor of 28. For substrate **4k** the reaction did not proceed at all, while for **4p** the reaction rate decreased drastically (conversion <10% after 48 h). As a consequence, we decided not to use **2i** any further. We then moved on to exploring the compatibility of substituents in the 7,7′-positions. For dibrominated **4q** and dimethyl-substituted **4r** a moderate selectivity was observed (*s* = 11), while a rather low selectivity factor of 8 was obtained for phenyl-substituted **4s**. 7,7′-Dimethoxy derivative **4t** was resolved with increased selectivity (*s* = 19). For **4r**–**t** it was necessary to work at a lower concentration as a result of the poor solubility; this was also detrimental to the reaction rate. Other substitution patterns and modified backbones included 4,4′-dimethylated **4u**, for which the selectivity collapsed (*s* = 4). The same was observed for H_8_–BINAM derivative **4v** (*s* = 3) and biaryldiamine **4w** (*s* = 4). Of note, the synthesis of 3,3′-disubstituted BINAMs with benzhydryl monoprotection failed in our hands; yields were very low. Overall, our method shows good compatibility with diverse substituents in the 6,6′- as well as the 7,7′-positions. Removal of the benzhydryl group under reductive conditions is possible at ambient temperature without racemization (see Supporting Information for details).

**Scheme 2:**
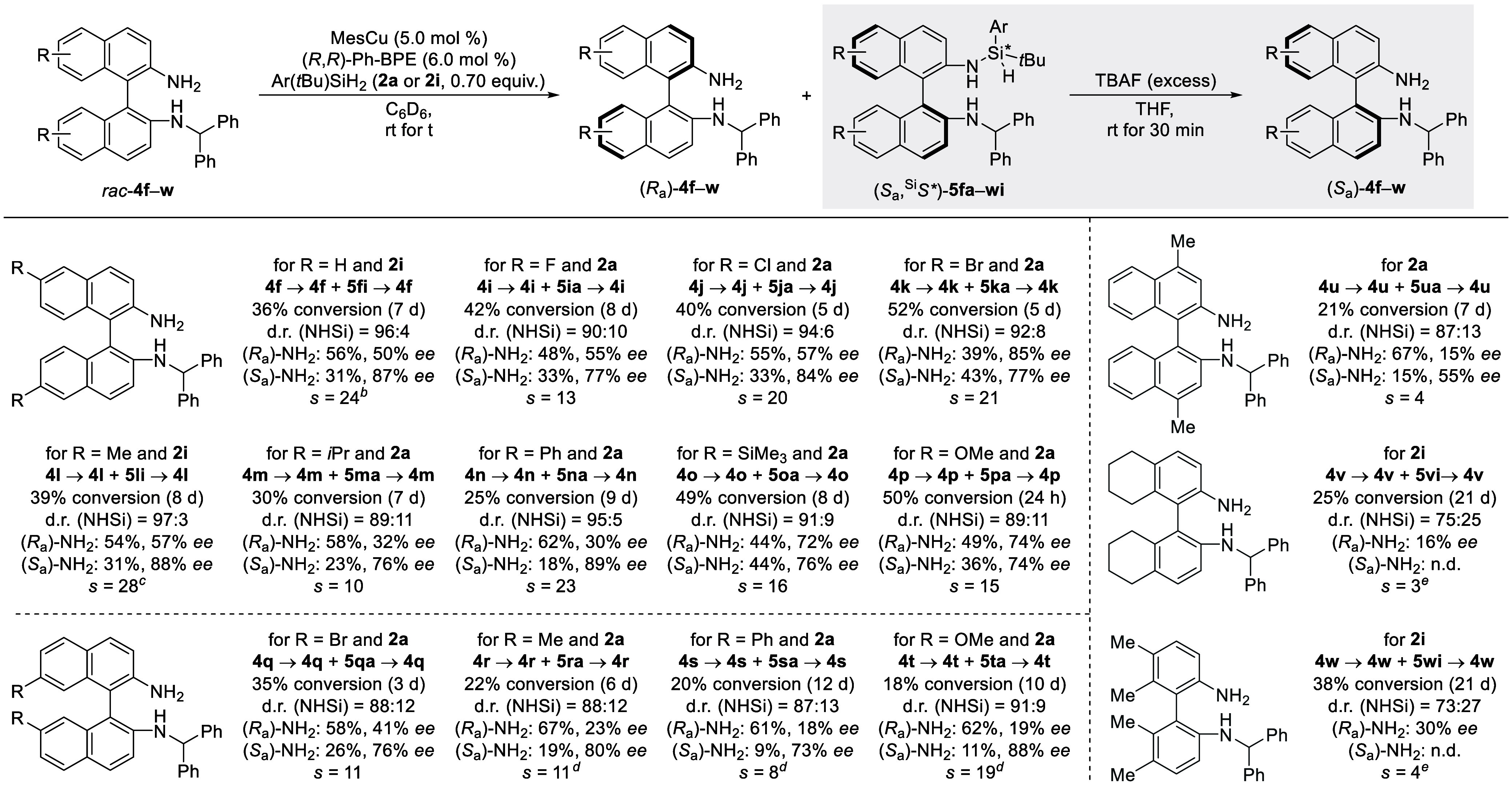
Substrate Scope^a^

a All reactions were carried out on a 0.2 mmol scale except for **4p** (0.18 mmol scale). See footnote of Table 2 for details.
b A selectivity factor of *s* = 25 was obtained when the reaction was performed on a 1.0 mmol scale.
c A selectivity factor of *s* = 17 was obtained with dihydrosilane **2a**.
d Reaction performed at a concentration of 0.18 M instead of 0.33 M.
e The selectivity factor was calculated using the conversion obtained by ^1^H NMR spectroscopic analysis of the crude reaction mixture.

Lastly, we revisited the desymmetrization of the dihydrosilane **2** and its role in our reaction.^16–20,54–56^ For this, we compared the diastereoselectivity of the refined model reaction with dihydrosilane **2i** in the presence of chiral (*R*,*R*)-Ph-BPE or achiral 1,2-bis­(dimethylphosphino)­ethane (dmpe) using enantiopure (*R*_a_)-**4f** and (*S*_a_)-**4f**, respectively (two pairs of reactions, Scheme 3). With the (*R*,*R*)-Ph-BPE ligand, the fast-reacting enantiomer (*S*_a_)-**4f** furnished (*S*_a_,^Si^ S*)-**5fi** with excellent diastereoselectivity (dr > 99:1). Conversely, slow-reacting (*R*_a_)-**4f** afforded the other diastereomer (*R*_a_,^Si^ S*)-**5fi** with diminished diastereoselectivity (dr = 20:80). These findings are consistent with our observation that good diastereoselectivity corresponds to good selectivity factors *s*. To learn about the substrate control in this desymmetrization, we replaced the chiral ligand with achiral dmpe. For both (*S*_a_)-**4f** and (*R*_a_)-**4f** the same diastereomer was favored with low diastereoselectivity of dr ≈ 65:35. This confirms that there is little substrate control in desymmetrization of the prochiral dihydrosilane. Overall, these observations show that the fast-reacting (*S*_a_)-**4f** benefits from matching catalyst and substrate control, whereas there is a mismatch scenario for the slow-reacting (*R*_a_)-**4f**, with the catalyst exerting a stronger asymmetric induction than the substrate.

**Scheme 3:**
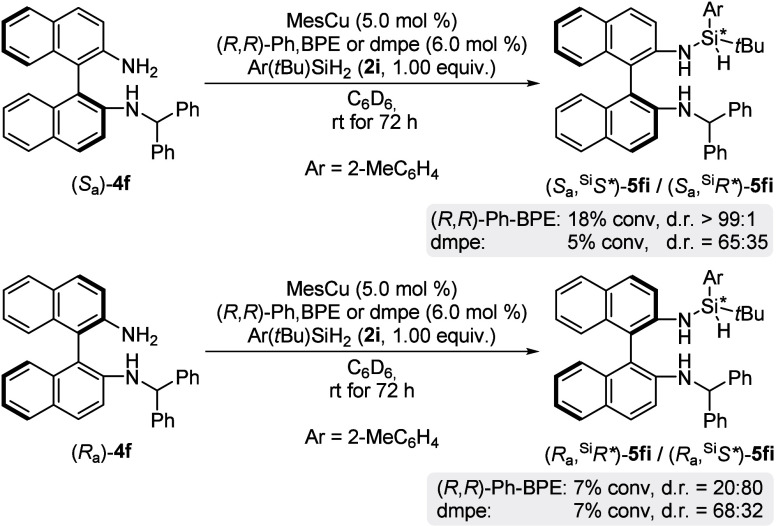
Stereochemical Control Experiments for the Desymmetrization of the Dihydrosilane^a^

a All reactions were carried out on a 0.2 mmol scale. Conversion and diastereomeric ratios were determined by ^1^H NMR spectroscopy of the crude reaction mixture.

In conclusion, we developed a new kinetic resolution of aromatic amines (anilines) by silylation, utilizing an asymmetric Cu–H-catalyzed, dehydrogenative Si–N coupling with a prochiral dihydrosilane that undergoes concomitant desymmetrization. Both the atropo- and the diastereoselectivity are mainly controlled by the chiral ligand (*R*,*R*)-Ph-BPE. Good to synthetically useful selectivity factors were obtained for various 6,6′- or 7,7′-disubstitued BINAM derivatives.

## Supplementary Material



## Data Availability

The data underlying this study are available in the published article and its Supporting Information.

## Notes

General procedures, experimental details, characterization, and spectral data for all new compounds (PDF)
