# Supplementary material for: Kinetic Resolution of BINAMs by Stereoselective Copper-Catalyzed Dehydrogenative Si–N Coupling with Prochiral Dihydrosilanes
Source: Org Lett. 2025 Jul 1;27(27):7428–33. doi: 10.1021/acs.orglett.5c02258 (PMC12261316; doi:10.1021/acs.orglett.5c02258)

Kinetic Resolution of BINAMs by Stereoselective Copper-Catalyzed Dehydrogenative Si–N Coupling with Prochiral Dihydrosilanes

Finn H. Gattwinkel and Martin Oestreich*

Institut für Chemie, Technische Universität Berlin,
Strasse des 17. Juni 115, 10623 Berlin, Germany
martin.oestreich@tu-berlin.de

Supporting Information

Table of Contents

1 General Information 3

2 General Procedures 6

2.1 Method A for the Synthesis of Dihydrosilanes (GP 1) 6

2.2 Method B for the Synthesis of Dihydrosilanes (GP 2) 6

2.3 Method C for the Synthesis of Dihydrosilanes (GP 3) 7

2.4 Method for *O*-Aryl Amination (GP 4) 7

2.5 General Procedure for the Installation of the Benzhydryl Group (GP 5) 8

2.6 General Procedure for the Synthesis of Arylamines (GP 6) 8

2.7 General Procedure for Synthesis of Binaphtylamines (GP 7) 9

2.8 General Procedure for the Kinetic Resolution of Arylamines (GP 8) 9

2.9 General Procedure for the Deprotection of Silylamines (GP 9) 9

3 Preparation of Dihydrosilanes 10

3.1 Preparation of Trichlorosilanes 10

3.2 Preparation of Dihydrosilanes 10

4 Preparation of Substrates 17

5 Kinetic Resolution of BINAM Derivatives 52

6 Scale-Up Experiment 71

7 Deprotection of the Benzhydryl Group 72

8 Determination of Absolute Configuration 73

9 Optimization of Reaction 74

9.1 Screening of the *N*-Protecting Group 74

9.2 Screening of Monohydrosilanes 75

9.3 Screening of Dihydrosilanes 76

10 References 77

11 HPLC traces 80

12 NMR Spectra 97

1 General Information

Reactions

Moisture and air-sensitive reactions were carried out using an *MBraun* glovebox under argon atmosphere (O_2 < 0.5 ppm, H_2O < 1.0 ppm) or conventional Schlenk techniques under a static pressure of nitrogen (fume hood) unless otherwise stated. Standard solvents and reagents were obtained from commercial suppliers and used as received unless otherwise stated. Glassware used in the glovebox was dried overnight at 120 °C or flame dried using a heat gun. All plastic syringes and needles used in the glovebox were dried overnight at 60 °C. Reagents and solvents were added through septa using disposable plastic syringes, cannulas as well as stainless steel cannulas stored at 120 °C. Solvents were added under a counterflow of nitrogen. For reactions performed at elevated temperatures the external oil bath temperature is given. For reactions performed in an autoclave the external heating mantle temperature is given.

Reagents and solvents

Standard reagents were obtained from commercial suppliers and used without further purification, unless otherwise stated. Technical grade solvents for extraction, chromatography, and reactions were distilled prior to use. Tetrahydrofuran (THF) and diethyl ether were dried over sodium and benzophenone, and freshly distilled prior to use. Toluene was dried over sodium and freshly distilled prior to use. Dichloromethane (CH_2Cl_2) and *n*-pentane were dried over calcium hydride and distilled prior to use. Toluene and deuterated benzene (C_6D_6) were degassed by three freeze-pump-thaw cycles, transferred to the glovebox and stored over thermally activated 4 Å molecular sieves.

Chromatography

Thin-layer chromatography was performed on *Macherey-Nagel* Alugram® Xtra SIL G/UV_{254} silica gel 60 or Alugram® Xtra ALOX N UV_{254} plates. Product spots were visualized under UV light (λ = 254 nm) and with a ceric ammonium molybdate stain. Column chromatography was performed on *Grace* 60 (40–63 μm, 230–400 mesh, ASTM) silica gel or *Merck* aluminium oxide 90 active neutral (63–200 μm, 70–230 mesh, ASTM). Automatic column chromatography was performed on Isolera One™ (*Biotage*) using Buchi EcoFlex columns (80 g, 40 g).

Gas liquid chromatography

Analytical gas-liquid chromatography (GLC) was performed on a *Varian* 430-GC gas chromatograph equipped with a *Varian* Factor Four Capillary column (length: 30 m, inner diameter: 0.25 mm, film thickness of the stationary phase: 0.25 μm). Unless otherwise stated, the following program was used: N_2 carrier gas:, injection temperature: 250 °C, detector temperature: 250 °C, flow rate: 4.0 mL/min; temperature program: starting temperature: 40 °C, heating rate 10 °C/min, end temperature: 280 °C for 10 min.

High Performance Liquid Chromatography

The enantiomeric excess was determined by analytical high performance liquid chromatography (HPLC)on an Agilent Technologies 1290 or 1200 infinity or instrument equipped with a chiral stationary phase using *Daicel Chiralpak*® and *Chiralcel*® columns. A mixture of *n*-heptane/isopropanol was utilized as the mobile phase.

Nuclear Magnetic Resonance (NMR) Spectroscopy

The NMR spectra were recorded on a *Bruker* AV 400 or AV 500 instruments and performed in $CDCl_3$, C_6D_6, $(CD_3)_2CO$ or $(CD_3)_2SO$ as deuterated solvent. The chemical shifts are reported in parts per million (ppm) and are referenced to the residual protic solvent signals and the deuterated solvent carbon signals respectively. ($CHCl_3$: δ = 7.26 ppm for ^{1}H NMR and $CDCl_3$: δ = 77.16 ppm for ^{13}C NMR, C_6D_5H: δ = 7.06 ppm for ^{1}H NMR and C_6D_6: δ = 128.06 ppm for ^{13}C NMR, $(CD_3)(CHD_2)CO$: δ = 2.05 ppm for ^{1}H NMR and $(CD_3)_2CO$: δ = 206.68 and 29.92 ppm, $(CD_3)(CHD_2)SO$: δ = 2.50 ppm for ^{1}H NMR and $(CD_3)_2SO$: δ = 39.51 ppm). ^{19}F and ^{29}Si NMR spectra are referenced in compliance with the unified scale for NMR chemical shifts as recommended by the IUPAC stating the chemical shift relative to CCl_3F and TMS, respectively. Data are reported as follows: chemical shift, multiplicity (s = singlet, d = doublet, t = triplet, q = quartet, quint = quintet, sext = sextet, sept = septet, m = multiplet, m_c = centered multiplet br = broad and combinations thereof), coupling constants (Hz), and integration.

Infrared Spectroscopy

Infrared (IR) spectra were recorded on an *Agilent Technologies* Cary 630 FT-IR spectrometer. Selected signals are reported in wavenumbers (cm^{-1}).

Mass Spectrometry

The mass spectrometric analyses were carried out by the Department of Mass Spectrometry at the *Institut für Chemie*, *Technische Universität Berlin* on a *Thermo Fisher* Scientific LTQ Orbitrap XL apparatus using APCI, ESI or LIFDI techniques with a linear ion trap analyzer.

Melting points

Melting points (m.p.) were determined with a *Stuart Scientific* SMP20 melting point apparatus and were not corrected.

Optical Rotation

Optical Rotations were measured on a *Schmidt & Haensch* Polartronic H532 polarimeter. The enantioenriched compound was dissolved in the specified solvent and transferred to a cuvette. The following equation was used to calculate the specific rotation:

$$[\alpha]_{\lambda}^{T} = \frac{[\alpha] \cdot 100}{c \cdot d}$$

The measured rotation is described by [α]. All measurements were carried out using the sodium D-line (λ = 589 nm) as the light source, indicated by the index "$_D$" at room temperature (T). The cuvette length is d = 1 dm and the concentration is reported in c = g/100 mL.

2 General Procedures

2.1 Method A for the Synthesis of Dihydrosilanes (GP 1)

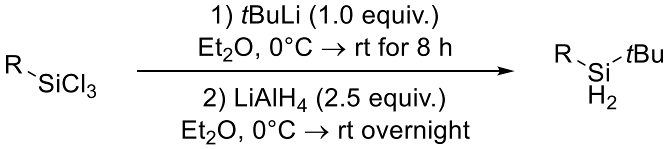

According to a modified literature procedure[1], a flame-dried Schlenk tube is charged with the indicated chlorosilane (1.0 equiv.) and diethyl ether is added (2 ml/mmol). The solution is cooled to 0°C before *tert*-butyllithium (1.0 equiv., 1.8–2.0 M in *n*-pentane) is added dropwise to the solution. The reaction is stirred at 0°C for 15 min and subsequently at room temperature for 8 h. A second flame-dried Schlenk tube is charged with lithium aluminium hydride (2.50 equiv.) and diethyl ether is added (1 mL/mmol chlorosilane). The resulting suspension is cooled to 0°C and the first reaction mixture is added via syringe or cannula transfer. The reaction is stirred at room temperature overnight. The reaction mixture is then cooled to 0°C, diluted with *n*-pentane (10 mL) and a saturated, aqueous potassium sodium tartrate solution (10 mL) is added dropwise. The reaction mixture is stirred until two clear layers are visible and the aqueous phase is extracted with *n*-pentane (3 × 30 mL). The combined organic layers are washed with saturated, aqueous potassium sodium tartrate solution (30 mL), dried over anhydrous Na_2SO_4, filtered and concentrated under reduced pressure. The residue is purified by bulb-to-bulb distillation to afford the respective dihydrosilane.

2.2 Method B for the Synthesis of Dihydrosilanes (GP 2)

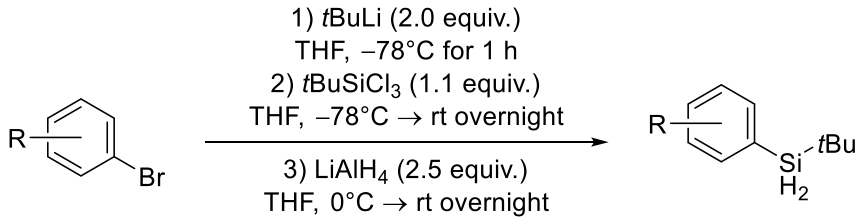

According to a modified literature procedure[1], a flame-dried Schlenk tube is charged with the indicated arylbromide (1.0 equiv.) and tetrahydrofuran is added (2 ml/mmol). The solution is cooled to −78°C before *tert*-butyllithium (2.0 equiv., 1.8–2.0 M in *n*-pentane) is added dropwise to the solution. The reaction is stirred at −78°C for 1 h, and subsequently a solution of *tert*-butyltrichlorosilane (1.1 equiv.) in tetrahydrofuran (1 mL/mmol arylbromide) is added to the reaction. The reaction is stirred at −78°C for 30 min, and subsequently at room temperature overnight. Then, the reaction is cooled down to 0°C and a solution of lithium aluminium hydride (2.50 equiv.) in tetrahydrofuran (1 mL/mmol arylbromide) is added dropwise. The reaction mixture is stirred at room temperature overnight. The reaction mixture is then cooled to 0°C, diluted with *n*-pentane (10 mL) and a saturated, aqueous potassium sodium tartrate solution (10 mL) is added dropwise.

The reaction mixture is stirred until two clear layers are visible and the aqueous phase is extracted with *n*-pentane (3 × 30 mL). The combined organic layers are washed with saturated, aqueous potassium sodium tartrate solution (30 mL), dried over anhydrous Na_2SO_4, filtered and concentrated under reduced pressure. The residue is purified by bulb-to-bulb distillation to afford the respective dihydrosilane.

2.3 Method C for the Synthesis of Dihydrosilanes (GP 3)

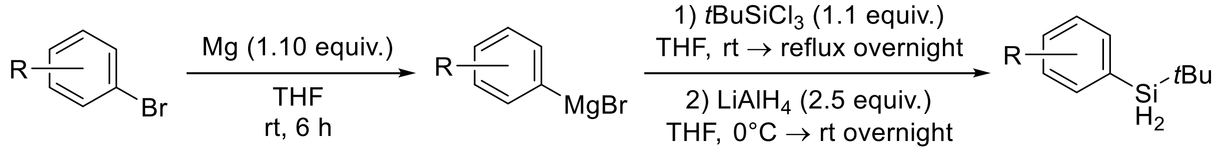

The reaction is carried out in a flame-dried Schlenk flask. To a suspension of activated magnesium turnings (1.50 equiv.) in tetrahydrofuran (1 mL/mmol) a grain of iodine is added. Then, the indicated arylbromide (1.65 equiv.) is added dropwise, while the reaction mixture is gently heated to initiate the reaction. After initiation the addition is continued, keeping the reaction at a gentle reflux. The reaction mixture is stirred at room temperature for 5 h. A flame-dried Schlenk tube, fitted with a reflux condenser, is charged with *tert*-butyltrichlorosilane (1.00 equiv.) and tetrahydrofuran (2 mL/mmol) is added. The solution is cooled to 0°C before the solution of the indicated Grignard reagent (1.00 equiv.) is added. The reaction mixture is heated to reflux stirred at reflux overnight, and then cooled down to room temperature. A second flame-dried Schlenk tube is charged with lithium aluminium hydride (2.50 equiv.) and tetrahydrofuran is added (1 mL/mmol chlorosilane). The resulting suspension is cooled to 0°C and the reaction mixture is added via cannula transfer. The reaction is stirred at room temperature overnight. The reaction mixture is then cooled to 0°C, diluted with *n*-pentane (10 mL) and a saturated, aqueous potassium sodium tartrate solution (10 mL) is added dropwise. The reaction mixture is stirred until two clear layers are visible. The layers are separated and the aqueous phase is extracted with *n*-pentane (3 × 30 mL). The combined organic layers are washed with saturated, aqueous potassium sodium tartrate solution (30 mL), dried over anhydrous Na_2SO_4, filtered and concentrated under reduced pressure. The residue is purified by bulb-to-bulb distillation to afford the respective dihydrosilane.

2.4 Method for *O*-Aryl Amination (GP 4)

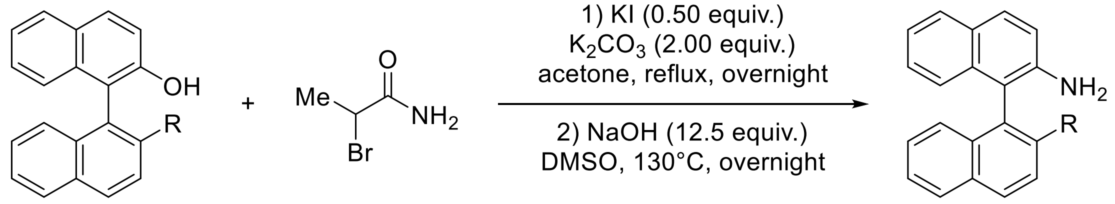

rac-**XX** **XX** (2.00 equiv.) *rac*-**XX**

According to a modified literature procedure[2], a flame-dried Schlenk tube is charged with the respective phenol (1.00 equiv.), 2-bromopropionamide (2.00 equiv.), potassium iodide (0.50 equiv.),

potassium carbonate (2.00 equiv.) and acetone (10 mL/mmol) is added. The reaction is heated to reflux and stirred at reflux overnight. After complete consumption of the starting material (monitored by TLC), the reaction is cooled down to room temperature and filtered through a pad of celite. The solvent is removed under reduced pressure and the crude is dissolved in DMSO (10 mL/mmol). Sodium hydroxide (12.5 equiv.) is added, and the reaction mixture is stirred at 130°C overnight. After complete conversion (monitored by TLC), the reaction mixture is cooled down to room temperature, diluted with ethyl acetate (2 × volume of DMSO) and poured into water. The layers are separated, and the aqueous phase is extracted with ethyl acetate (2 ×). The combined organic layers are washed with water (5 ×) and brine, dried over anhydrous $MgSO_4$, filtered and concentrated under reduced pressure. The residue is purified by flash column chromatography on silica gel using cyclohexane:dichloromethane mixtures as eluent.

2.5 General Procedure for the Installation of the Benzhydryl Group (GP 5)

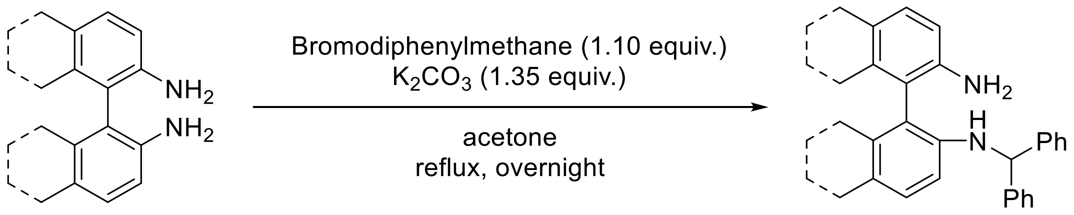

A two-neck-flask fitted with a reflux condenser is charged with the respective diamine (1.00 equiv.) and acetone (10 mL/mmol) is added. Potassium carbonate (1.35 equiv.) is added in one portion and the reaction mixture is stirred for 5 min. Then bromodiphenylmethane (1.10 equiv.) is added, the reaction mixture is heated to reflux and stirred at reflux overnight. The reaction mixture is cooled down to room temperature, filtered over a pad of Celite and concentrated under reduced pressure. The residue is purified by flash column chromatography on silica gel using cyclohexane:dichloromethane mixtures as eluent.

2.6 General Procedure for the Synthesis of Arylamines (GP 6)

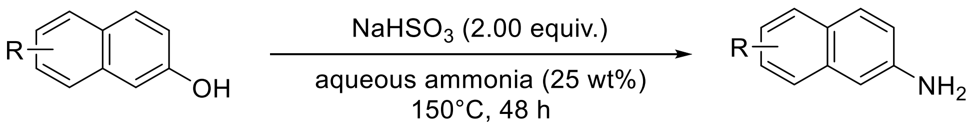

According to a literature procedure[3], in an autoclave the corresponding naphthol (1.00 equiv.) and sodium bisulfite (2.00 equiv.) are suspended in aqueous ammonia (25 wt%, 5 mL/mmol) and the reaction mixture is heated to 150 °C and stirred at that temperature for 48 h. After cooling down to ambient temperature, the reaction mixture is poured into brine (50 mL) and extracted with ethyl acetate (3 × 50 mL). The combined organic layers are washed with brine (50 mL), dried over anhydrous $MgSO_4$, filtered and concentrated under reduced pressure. The residue is purified by flash column chromatography on silica gel or recrystallization.

2.7 General Procedure for Synthesis of Binaphtylamines (GP 7)

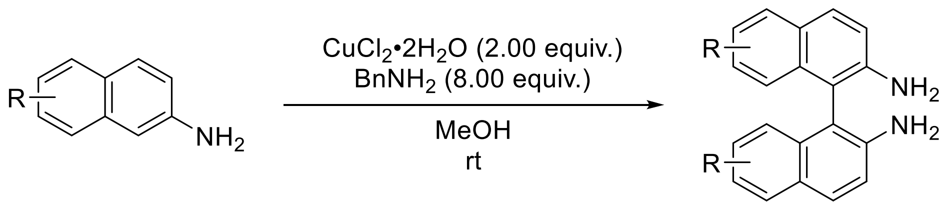

According to a modified literature procedure[3,4], a Schlenk flask is charged with degassed methanol (150 mL), copper(II) chloride dihydrate (2.00 equiv.) is added, and the solution is degassed by cannula for an additional 15 min. A second Schlenk flask is charged with degassed methanol (50 mL), benzylamine (8.00 equiv.) is added, and the solution is degassed by cannula for an additional 15 min. The benzylamine solution is added to the copper(II) chloride dihydrate solution and the reaction mixture is degassed an additional 10 min, before the corresponding arylamine (1.00 equiv.) is added. The reaction mixture is stirred at ambient temperature. After completion, aqueous conc. HCl (50 mL) is added dropwisely to the reaction mixture and stirred for 5 min after complete addition. Then, aqueous ammonia (100 mL) is added dropwisely. After complete addition, the reaction mixture is diluted with water (1 L) and extracted with chloroform (3 × 100 mL). The combined organic layers are washed with brine (100 mL), dried over anhydrous $MgSO_4$, filtered and concentrated under reduced pressure. The residue is purified by flash column chromatography on silica gel.

2.8 General Procedure for the Kinetic Resolution of Arylamines (GP 8)

In an argon-filled glovebox, an oven-dried screw cap vial is charged with MesCu[5] (1.8 mg, 10 µmol, 5.0 mol%), (*R*,*R*)-Ph-BPE (6.1 mg, 12 µmol, 6.0 mol%) and C_6D_6 (0.1 mL). The mixture is stirred at room temperature for 5 min, to give a clear, yellow solution. A solution of the indicated arylamine (0.20 mmol, 1.00 equiv.) and dihydrosilane (0.14 mmol, 0.70 equiv.) in C_6D_6 (0.5 mL) is added and the reaction mixture is stirred in the glovebox at ambient temperature for the indicated time. The reaction is monitored by ^{1}H NMR spectroscopy. After completion, the reaction mixture is directly purified by column chromatography on deactivated neutral alumina (3wt% water added).

2.9 General Procedure for the Deprotection of Silylamines (GP 9)

In a dram vial, the respective silylamine is dissolved in THF (2 mL) and TBAF (1 mL, 1 M in tetrahydrofuran) is added. The reaction mixture is stirred for 30 min at ambient temperature, then water (1 mL) is added. mixture is poured into brine (50 mL) and extracted with ethyl acetate (4 × 1 mL). The combined organic layers are dried over anhydrous Na_2SO_4, filtered and concentrated under reduced pressure. The residue is purified by flash column chromatography on silica gel using a mixture of cyclohexane:dichloromethane as eluent.

3 Preparation of Dihydrosilanes

3.1 Preparation of Trichlorosilanes

Phenyltrichlorosilane was commercially available from *TCI* and used as received. *Tert*-butyltrichlorosilane[6] and 2,6-Dimethylphenyltrichlorosilane[7] were synthesized according to literature known procedures.

3.2 Preparation of Dihydrosilanes

***tert*-Butyl(phenyl)silane (2a)**

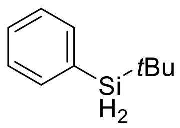

2a

$C_{10}H_{16}Si$

M = 164.32 g/mol

Prepared from $PhSiCl_3$ (6.35 g, 4.81 mL, 30.0 mmol, 1.00 equiv.), *t*BuLi (1.92 M in *n*-pentane, 15.6 mL, 30.0 mmol, 1.00 equiv.) and $LiAlH_4$ (2.85 g, 75.0 mmol, 2.50 equiv.) according to **GP 1**. Bulb-to-bulb distillation (140°C/50 mbar) afforded *tert*-butyl(phenyl)silane (**2a**, 3.95 g, 24.0 mmol, 80%) as a colorless liquid.

^{1}H NMR (500 MHz, $CDCl_3$, 298 K): *δ*/ppm = 7.59 (d, *J* = 7.6 Hz, 2H), 7.46–7.32 (m, 3H), 4.17 (s, 2H), 1.04 (s, 9H).
^{13}C{^{1}H} NMR (126 MHz, $CDCl_3$, 298 K): *δ*/ppm = 136.1, 132.4, 129.7, 128.0, 27.6, 16.6.
^{29}Si{^{1}H} DEPT NMR (99 MHz, $CDCl_3$, 298 K): *δ*/ppm = −14.3.
IR (ATR): ṽ/cm^{-1} = 2948 (m), 2926 (m), 2855 (m), 2124 (s), 1462 (m), 1427 (m), 1117 (s), 1010 (w), 926 (s), 837 (vs), 729 (s), 696 (vs).
HRMS (APCI) calculated for $C_{10}H_{15}Si^+$ $[(M-H)]^+$: 163.0938; found: 163.0936.

The spectroscopic data are in accordance with those reported in the literature.[10]

***tert*-Butyl(4-tolyl)silane (2b)**

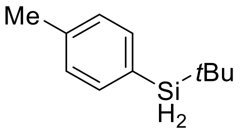

2b
$C_{11}H_{18}Si$
M = 178.35 g/mol

Prepared from 1-bromo-4-methylbenzene (1.71 g, 1.20 mL, 10.0 mmol, 1.00 equiv.), *t*BuLi (1.9 M in *n*-pentane, 10.5 mL, 20.0 mmol, 2.00 equiv.), *t*BuSiCl$_3$ (2.11 g, 11.0 mmol, 1.10 equiv.) and LiAlH$_4$ (949 mg, 25.0 mmol, 2.50 equiv.) according to **GP 2**. Bulb-to-bulb distillation (160°C/50 mbar) afforded *tert*-butyl(4-tolyl)silane (**2b**, 1.17 g, 6.56 mmol, 66%) as a colorless liquid.

^{1}H NMR (500 MHz, C_6D_6, 298 K): *δ*/ppm = 7.49 (d, *J* = 7.7 Hz, 2H), 7.21 (d, *J* = 7.5 Hz, 2H), 4.16 (s, 2H), 2.38 (s, 3H), 1.04 (s, 9H).
^{13}C{^{1}H} NMR (126 MHz, $CDCl_3$, 298 K): *δ*/ppm = 139.6, 136.1, 128.8, 128.7, 27.6, 21.7, 16.6.
^{29}Si{^{1}H} DEPT NMR (99 MHz, $CDCl_3$, 298 K): *δ*/ppm = −14.6.
IR (ATR): ṽ/cm^{-1} = 2948 (w), 2927 (w), 2855 (m), 2121 (s), 1602 (w), 1467 (m), 1108 (s), 1009 (m), 926 (s), 841 (vs), 791 (vs), 709 (w).
HRMS (APCI) calculated for $C_{11}H_{17}Si^+$ [(M−H)]$^+$: 177.1094; found: 177.1094.

***tert*-Butyl(4-(*tert*-butyl)phenyl)silane (2c)**

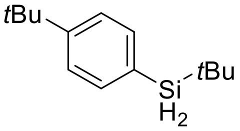

2c
$C_{14}H_{24}Si$
M = 220.43 g/mol

Prepared from 1-bromo-(4-*tert*-butyl)benzene (4.80 g, 3.90 mL, 22.5 mmol, 1.50 equiv.), Mg (602 mg, 24.8 mmol, 1.65 equiv.), *t*BuSiCl$_3$ (2.87 g, 15.0 mmol, 1.00 equiv.) and LiAlH$_4$ (1.42 mg, 37.5 mmol, 2.50 equiv.) according to **GP 3**. Bulb-to-bulb distillation (200°C/50 mbar) afforded *tert*-butyl(3,5-dimethylphenyl)silane (**2c**, 771 mg, 3.50 mmol, 23%) as a colorless liquid.

^{1}H NMR (500 MHz, $CDCl_3$, 298 K): *δ*/ppm = 7.52 (d, *J* = 8.1 Hz, 2H), 7.40 (d, *J* = 8.1 Hz, 2H), 4.15 (s, 2H), 1.34 (s, 9H), 1.04 (s, 9H).
^{29}Si{^{1}H} DEPT NMR (99 MHz, $CDCl_3$, 298 K): *δ*/ppm = −14.9.
^{13}C{^{1}H} NMR (126 MHz, $CDCl_3$, 298 K): *δ*/ppm = 152.7, 136.0, 128.7, 125.0, 34.9, 31.4, 27.6, 16.6.
IR (ATR): ṽ/cm^{-1} = 2951 (m), 2927 (m), 2855 (m), 2122 (s), 1598 (w), 1461 (m), 1386 (m), 1361 (m), 1267 (m), 1088 (s), 1009 (w), 926 (vs), 845 (vs), 815 (vs), 728 (w).
HRMS (APCI) calculated for $C_{14}H_{23}Si^+$ [(M−H)]$^+$: 219.1564; found: 219.1564.

***tert*-Butyl(4-methoxyphenyl)silane (2d)**

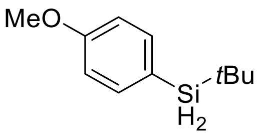

2d
$C_{11}H_{18}OSi$
M = 194.35 g/mol

Prepared from 1-bromo-4-methoxybenzene (935 mg, 0.63 mL, 5.00 mmol, 1.00 equiv.), *t*BuLi (1.9 M in *n*-pentane, 5.26 mL, 10.0 mmol, 2.00 equiv.), *t*BuSiCl$_3$ (1.05 g, 5.50 mmol, 1.10 equiv.) and LiAlH$_4$ (474 mg, 12.5 mmol, 2.50 equiv.) according to **GP 2**. Bulb-to-bulb distillation (160°C/10 mbar) afforded *tert*-butyl(4-methoxyphenyl)silane (**2d**, 679 mg, 3.49 mmol, 70%) as a colorless liquid.

^{1}H NMR (500 MHz, $CDCl_3$, 298 K): *δ*/ppm = 7.51 (d, *J* = 8.5 Hz, 2H), 6.92 (d, *J* = 8.5 Hz, 2H), 4.14 (s, 2H), 3.83 (s, 3H), 1.02 (s, 9H).
^{13}C{^{1}H} NMR (126 MHz, $CDCl_3$, 298 K): *δ*/ppm = 161.1, 137.5, 123.1, 113.8, 55.2, 27.5, 16.6.
^{29}Si{^{1}H} DEPT NMR (99 MHz, $CDCl_3$, 298 K): *δ*/ppm = −14.9.
IR (ATR): ṽ/cm^{-1} = 2926 (m), 2854 (m), 2120 (s), 1592 (s), 1501 (m), 1461 (m), 1278 (s), 1245 (s), 1180 (s), 1111 (s), 1032 (m), 925 (vs), 843 (vs), 808 (vs), 713 (w).
HRMS (APCI) calculated for $C_{11}H_{17}OSi^+$ [(M−H)]$^+$: 193.1043; found: 193.1042.

The spectroscopic data are in accordance with those reported in the literature.[11]

***tert*-Butyl(3-tolyl)silane (2f)**

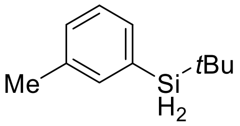

2f
$C_{11}H_{18}Si$
M = 178.35 g/mol

Prepared from 1-bromo-3-methylbenzene (1.71 g, 1.20 mL, 10.0 mmol, 1.00 equiv.), *t*BuLi (1.9 M in *n*-pentane, 10.5 mL, 20.0 mmol, 2.00 equiv.), *t*BuSiCl$_3$ (2.11 g, 11.0 mmol, 1.10 equiv.) and LiAlH$_4$ (949 mg, 25.0 mmol, 2.50 equiv.) according to **GP 2**. Bulb-to-bulb distillation (160°C/50 mbar) afforded *tert*-butyl(3-tolyl)silane (**2f**, 1.06 g, 5.93 mmol, 59%) as a colorless liquid.

^{1}H NMR (500 MHz, C_6D_6, 298 K): *δ*/ppm = 7.44–7.40 (m, 2H), 7.33–7.26 (m, 2H), 4.19 (s, 2H), 2.41 (s, 3H), 1.07 (s, 9H).
^{13}C{^{1}H} NMR (126 MHz, $CDCl_3$, 298 K): *δ*/ppm = 137.3, 136.7, 133.1, 132.2, 130.5, 127.9, 27.7, 21.6, 16.6.
^{29}Si{^{1}H} DEPT NMR (99 MHz, $CDCl_3$, 298 K): *δ*/ppm = −14.3.

IR (ATR): ṽ/cm^{-1} = 2926 (m), 2855 (m), 2123 (s), 1467 (m), 1121 (m), 927 (vs), 858 (vs), 833 (vs), 771 (vs), 696 (s).
HRMS (APCI) calculated for $C_{11}H_{17}Si^+$ $[(M-H)]^+$: 177.1094; found: 177.1094.

***tert*-Butyl(naphthalen-2-yl)silane (2g)**

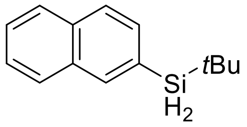

2g
$C_{14}H_{18}Si$
M = 214.38 g/mol

Prepared from 2-bromonaphtalene (2.07 g, 10.0 mmol, 1.00 equiv.), *t*BuLi (1.9 M in *n*-pentane, 10.5 mL, 20.0 mmol, 2.00 equiv.), *t*BuSiCl$_3$ (2.11 g, 11.0 mmol, 1.10 equiv.) and LiAlH$_4$ (949 mg, 25.0 mmol, 2.50 equiv.) according to **GP 2**. Bulb-to-bulb distillation (240°C/10 mbar) afforded *tert*-butyl(naphtalen-2-yl)silane (**2g**, 1.36 g, 6.32 mmol, 63%) as a pale yellow solid.

melting point 54–58 °C.
^{1}H NMR (500 MHz, $CDCl_3$, 298 K): *δ*/ppm = 8.12 (s, 1H), 7.89–7.81 (m, 3H), 7.64 (dd, *J* = 8.1, 1.3 Hz, 1H), 7.52 (m$_c$, 2H), 4.31 (s, 2H), 1.08 (s, 9H).
^{13}C{^{1}H} NMR (126 MHz, $CDCl_3$, 298 K): *δ*/ppm = 137.2, 134.0, 133.0, 131.8, 129.9, 128.2, 127.9, 127.1, 126.8, 126.2, 27.7, 16.8.
^{29}Si{^{1}H} DEPT NMR (99 MHz, $CDCl_3$, 298 K): *δ*/ppm = −13.9.
IR (ATR): ṽ/cm^{-1} = 3051 (w), 2921 (m), 2850 (m), 2138 (s), 1457 (s), 1087 (m), 1008 (w), 920 (vs), 853 (vs), 813 (vs), 737 (vs).
HRMS (APCI) calculated for $C_{14}H_{17}Si^+$ $[(M-H)]^+$: 213.1094; found: 213.1095.

The spectroscopic data are in accordance with those reported in the literature.[11]

***tert*-Butyl(3,5-dimethylphenyl)silane (2h)**

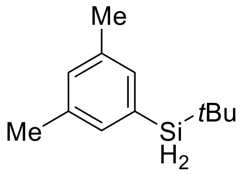

2h
$C_{12}H_{20}Si$
M = 192.38 g/mol

Prepared from 1-bromo-3,5-dimethylbenzene (4.16 g, 3.06 mL, 22.5 mmol, 1.50 equiv.), Mg (602 mg, 24.8 mmol, 1.65 equiv.), *t*BuSiCl$_3$ (2.87 g, 15.0 mmol, 1.00 equiv.) and LiAlH$_4$ (1.42 mg, 37.5 mmol, 2.50 equiv.) according to **GP 3**. Bulb-to-bulb distillation (160°C/50 mbar) afforded *tert*-butyl(3,5-dimethylphenyl)silane (**2h**, 690 mg, 3.59 mmol, 24%) as a colorless liquid.

^{1}H NMR (500 MHz, $CDCl_3$, 298 K): *δ*/ppm = 7.20 (s, 2H), 7.06 (s, 1H), 4.12 (s, 2H), 2.34 (s, 6H), 1.04 (s, 9H).

^{29}Si{^{1}H} DEPT NMR (99 MHz, $CDCl_3$, 298 K): *δ*/ppm = −14.3.

^{13}C{^{1}H} NMR (126 MHz, $CDCl_3$, 298 K): *δ*/ppm = 137.3, 133.8, 132.0, 131.5, 27.7, 21.5, 16.6.

IR (ATR): ṽ/cm^{-1} = 3014 (w), 2925 (m), 2855 (m), 2122 (s), 1596 (w), 1467 (m), 1268 (w), 1141 (m), 1010 (w), 927 (vs), 861 (vs), 817 (vs), 693 (m).

HRMS (APCI) calculated for $C_{12}H_{19}Si^+$ [(M−H)]$^+$: 191.1251; found: 191.1250.

***tert*-Butyl(2-tolyl)silane (2i)**

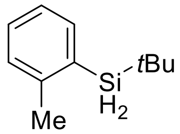

2i
$C_{11}H_{18}Si$
M = 178.35 g/mol

Prepared from 1-bromo-2-methylbenzene (1.71 g, 1.20 mL, 10.0 mmol, 1.00 equiv.), *t*BuLi (1.9 M in *n*-pentane, 10.5 mL, 20.0 mmol, 2.00 equiv.), *t*BuSiCl$_3$ (2.11 g, 11.0 mmol, 1.10 equiv.) and LiAlH$_4$ (949 mg, 25.0 mmol, 2.50 equiv.) according to **GP 2**. Bulb-to-bulb distillation (160°C/50 mbar) afforded *tert*-butyl(2-tolyl)silane (**2i**, 1.10 g, 6.18 mmol, 62%) as a colorless liquid.

^{1}H NMR (500 MHz, $CDCl_3$, 298 K): *δ*/ppm = 7.52 (dd, *J* = 7.2, 1.6 Hz, 1H), 7.31 (td, *J* = 7.5, 1.5 Hz, 1H), 7.22–7.14 (m, 2H), 4.24 (s, 2H), 2.48 (s, 3H), 1.05 (s, 9H).

^{13}C{^{1}H} NMR (126 MHz, $CDCl_3$, 298 K): *δ*/ppm = 144.3, 137.7, 131.7, 130.1, 129.8, 125.0, 28.1, 23.5, 17.3.

^{29}Si{^{1}H} DEPT NMR (99 MHz, $CDCl_3$, 298 K): *δ*/ppm = −19.1.

IR (ATR): ṽ/cm^{-1} = 2948 (w), 2926 (m), 2855 (m), 2126 (s), 1461 (m), 1361 (w), 1128 (w), 1009 (w), 938 (vs), 840 (vs), 820 (vs), 741 (vs).

HRMS (APCI) calculated for $C_{11}H_{17}Si^+$ [(M−H)]$^+$: 177.1094; found: 177.1093.

***tert*-Butyl(naphthalen-1-yl)silane (2j)**

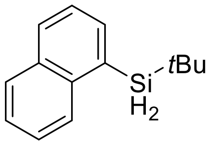

2j
$C_{14}H_{18}Si$
M = 214.38 g/mol

Prepared from 1-bromonaphtalene (2.07 g, 1.40 mL, 10.0 mmol, 1.00 equiv.), *t*BuLi (1.9 M in *n*-pentane, 10.5 mL, 20.0 mmol, 2.00 equiv.), *t*BuSiCl$_3$ (2.11 g, 11.0 mmol, 1.10 equiv.) and LiAlH$_4$ (949 mg, 25.0 mmol, 2.50 equiv.) according to **GP 2**. Bulb-to-bulb distillation (240°C/10 mbar) afforded *tert*-butyl(naphtalen-1-yl)silane (**2j**, 1.16 g, 5.41 mmol, 54%) as a pale yellow oil.

^{1}H NMR (500 MHz, $CDCl_3$, 298 K): *δ*/ppm = 8.17 (d, *J* = 8.1 Hz, 1H), 7.92 (d, *J* = 8.1 Hz, 1H), 7.89–7.85 (m, 1H), 7.81 (d, *J* = 6.9 Hz, 1H), 7.57 – 7.45 (m, 3H), 4.55 (s, 2H), 1.07 (s, 9H).

^{13}C{^{1}H} NMR (126 MHz, $CDCl_3$, 298 K): *δ*/ppm = 137.6, 137.2, 133.4, 131.3, 130.6, 128.9, 128.9, 126.1, 125.8, 125.2, 28.3, 17.5.

^{29}Si{^{1}H} DEPT NMR (99 MHz, $CDCl_3$, 298 K): *δ*/ppm = −17.9.

IR (ATR): ṽ/cm^{-1} = 3053 (w), 2925 (m), 2853 (m), 2124 (s), 1503 (m), 1461 (m), 1361 (w), 1143 (m), 1009 (m), 984 (m), 936 (vs), 837 (vs), 792 (vs), 772 (vs), 731 (s), 662 (m).

HRMS (APCI) calculated for $C_{14}H_{17}Si^+$ [(M−H)]$^+$: 213.1094; found: 213.1094.

***tert*-Butyl(2,6-dimethylphenyl)silane (2k)**

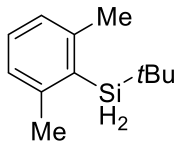

2k
$C_{12}H_{20}Si$
M = 192.38 g/mol

Prepared from 2,6-Dimethylphenyltrichlorosilane (1.76 g, 7.35 mmol, 1.00 equiv.), *t*BuLi (1.76 M in *n*-pentane, 4.2 mL, 7.35 mmol, 1.00 equiv.) and $LiAlH_4$ (697 mg, 18.4 mmol, 2.50 equiv.) according to **GP 1**. Bulb-to-bulb distillation (200°C/25 mbar) afforded *tert*-butyl(2,6-dimethylphenyl)silane (**2k**, 1.01 g, 5.23 mmol, 71%) as a colorless liquid.

^{1}H NMR (500 MHz, $CDCl_3$, 298 K): *δ*/ppm = 7.20 (t, *J* = 7.6 Hz, 1H), 7.04 (d, *J* = 7.6 Hz, 2H), 4.33 (s, 2H), 2.51 (s, 6H), 1.09 (s, 9H).

^{13}C{^{1}H} NMR (126 MHz, $CDCl_3$, 298 K): *δ*/ppm = 145.2, 130.9, 129.7, 127.4, 28.9, 24.6, 18.3.

^{29}Si{^{1}H} DEPT NMR (99 MHz, $CDCl_3$, 298 K): *δ*/ppm = −30.1.

IR (ATR): ṽ/cm^{-1} = 2950 (w), 2926 (m), 2854 (m), 2136 (s), 1586 (w), 1564 (w), 1447 (m), 1377 (w), 1360 (w), 1125 (w), 962 (vs), 841 (vs), 820 (vs), 767 (vs), 715 (m).

HRMS (APCI) calculated for $C_{12}H_{19}Si^+$ [(M−H)]$^+$: 191.1251; found: 191.1250.

Cyclohexyl(phenyl)silane (S1)

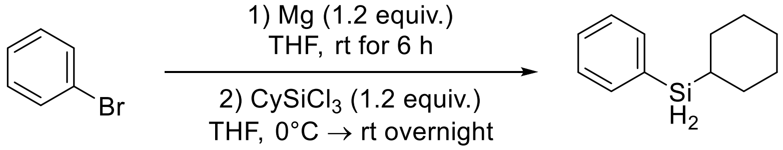

S1
$C_{12}H_{18}Si$
M = 190.36 g/mol

According to a modified literature procedure[8], the reaction was carried out in a flame-dried Schlenk flask. To a suspension of activated magnesium turnings (243 mg, 8.33 mmol, 1.20 equiv.) in tetrahydrofuran (17 mL) a grain of iodine was added. Then bromobenzene (1.31 g, 0.87 mL, 8.33 mmol, 1.00 equiv.) was added dropwise, while the reaction mixture was gently heated to initiate the reaction. After initiation the addition was continued, keeping the reaction at a gentle reflux. The reaction mixture was stirred at room temperature for 5 h. A flame-dried Schlenk tube, was charged with cyclohexyltrichlorosilane (2.18 g, 1.77 mL, 10.0 mmol, 1.20 equiv.) and tetrahydrofuran (20 mL) was added. The solution was cooled to 0°C before the solution of the Grignard reagent (1.0 equiv.) was added dropwise. The reaction mixture was stirred at 0°C for 30 min and overnight at room temperature. A second flame-dried Schlenk tube was charged with lithium aluminium hydride (790 mg, 20.8 mmol, 2.50 equiv.) and tetrahydrofuran was added (9 mL). The resulting suspension was cooled to 0°C and the reaction mixture was added via cannula transfer. The reaction mixture was stirred at room temperature overnight. The reaction mixture was then cooled to 0°C, diluted with *n*-pentane (10 mL) and a saturated, aqueous potassium sodium tartrate solution (10 mL) was added dropwise. The reaction mixture was stirred until two clear layers were visible and the aqueous phase was extracted with *n*-pentane (3 × 30 mL). The combined organic layers were washed with saturated, aqueous potassium sodium tartrate solution (30 mL), dried over anhydrous Na_2SO_4, filtered and concentrated under reduced pressure. The residue was purified by bulb-to-bulb distillation (180°C/25 mbar) to afford cyclohexyl(phenyl)silane (**S1**, 1.12 g, 5.91 mmol, 71%) as a colorless liquid.

^{1}H NMR (500 MHz, $CDCl_3$, 298 K): δ/ppm = 7.57 (m_c, 2H), 7.38 (m_c, 3H), 4.18 (d, J = 3.0 Hz, 2H), 1.82–1.67 (m, 5H), 1.27 (m_c, 5H), 1.15–1.07 (m, 1H).
^{13}C{^{1}H} NMR (126 MHz, $CDCl_3$, 298 K): δ/ppm = 135.8, 132.3, 129.6, 128.0, 29.0, 27.8, 26.8, 22.3.
^{29}Si{^{1}H} DEPT NMR (99 MHz, $CDCl_3$, 298 K): δ/ppm = −24.3.
IR (ATR): $\tilde{\nu}$/cm^{-1} = 2916 (s), 2845 (m), 2212 (s), 1444 (m), 1427 (m), 1116 (m), 996 (w) 929 (s), 884 (m), 860 (s), 827 (vs), 731 (s), 697 (vs).
HRMS (APCI) calculated for $C_{12}H_{17}Si^+$ $[(M-H)]^+$: 189.1094; found: 189.1094.

The spectroscopic data are in accordance with those reported in the literature.[9]

4 Preparation of Substrates

The following compounds were prepared according to literature-known procedures:

6-Chloro-2-naphthoic acid.[S12]

4-Methylnaphthalen-2-ol.[S13]

6-Methylnaphthalen-2-ol.[S14]

7-Methylnaphthalen-2-ol.[S15]

6-Isopropylnaphthalen-2-ol.[S16]

6-Phenylnaphthalen-2-ol.[S17]

7-Phenylnaphthalen-2-ol.[S17]

7-Methoxynaphthalen-2-amin.[S18]

5,5',6,6',7,7',8,8'-Octahydro-[1,1'-binaphthalene]-2,2'-diamine.[S19]

N^2,N^2-Dimethyl-[1,1'-binaphthalene]-2,2'-diamine.[S20]

N^2-Methyl-[1,1'-binaphthalene]-2,2'-diamine.[S21]

N^2-Isopropyl-[1,1'-binaphthalene]-2,2'-diamine.[S21]

N^2-Benzyl-[1,1'-binaphthalene]-2,2'-diamine.[S19]

N^2-Phenyl-[1,1'-binaphthalene]-2,2'-diamine.[S22]

2'-Methyl-[1,1'-binaphthalen]-2-amine (**S30**).[S23]

***rac*-N^2-Benzhydryl-[1,1'-binaphthalene]-2,2'-diamine (*rac*-4f)**

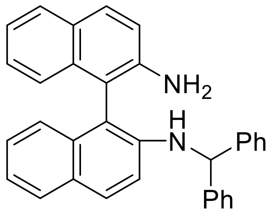

rac-**4f**
$C_{33}H_{26}N_2$
M = 450.59 g/mol

A flame-dried Schlenk tube was charged with *rac*-BINAM (2.56 g, 9.00 mmol, 1.50 equiv.) and dry DMF was added (18 mL). To the solution potassium carbonate (2.49 g, 18.0 mmol, 3.00 equiv.) was added, and the reaction mixture was stirred for 10 min. Then bromodiphenylmethane (1.48 g, 6.00 mmol, 1.00 equiv.) was added and the reaction mixture was stirred at ambient temperature for 72 h. The reaction mixture was diluted with water (20 mL) and extracted with dichloromethane (3 × 50 mL) The combined organic layers were washed with water (3 × 50 mL) brine (50 mL), dried over anhydrous $MgSO_4$, filtered and concentrated under reduced pressure. The residue was purified by flash column chromatography on silica gel (cyclohexane:dichloromethane = 1:1) to afford *rac*-N^2-benzhydryl-[1,1'-binaphthalene]-2,2'-diamine (*rac*-**4f**, 679 mg, 1.51 mmol, 25%) as an off-white solid.

R_f = 0.19.

^{1}H NMR (500 MHz, C_6D_6) δ/ppm = 7.67 (d, J = 8.1 Hz, 1H), 7.64 (d, J = 9.0 Hz, 1H), 7.59 (m_c, 1H), 7.49 (d, J = 8.8 Hz, 1H), 7.34 (m_c, 1H), 7.29 (d, J = 8.4 Hz, 1H), 7.22–7.17 (m, 4H), 7.14–6.98 (m, 7H), 6.98–6.83 (m, 4H), 6.58 (d, J = 8.7 Hz, 1H), 5.63 (d, J = 5.5 Hz, 1H), 4.57 (d, J = 5.9 Hz, 1H), 3.10 (s, 2H).

^{13}C{^{1}H} NMR (126 MHz, C_6D_6) δ/ppm = 143.9, 143.7, 143.5, 143.3, 134.6, 134.3, 129.9, 129.8, 128.9, 128.8, 128.6, 128.6, 128.6, 127.7, 127.5, 127.4, 127.4, 127.2, 124.7, 124.6, 122.7, 122.6, 118.2, 115.4, 113.9, 112.0, 62.9.

IR (ATR): ṽ/cm^{-1} = 3372 (w), 1615 (s), 1592 (s), 1485 (m), 1420 (m), 1343 (m), 1282 (m), 807 (vs), 741 (vs), 697 (vs).

HRMS (APCI) calculated for $C_{33}H_{27}N_2^+$ [(M+H)]$^+$: 451.2169; found: 451.2169.

(R_a)-N^2-Benzhydryl-[1,1'-binaphthalene]-2,2'-diamine [(R_a)-4f)]

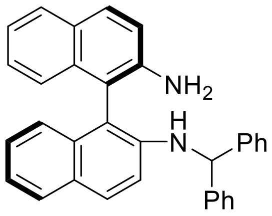

(R_a)-**4f**
$C_{33}H_{26}N_2$
M = 450.59 g/mol

Prepared from (R_a)-BINAM [(R_a)-**4a**, 853 mg, 3.00 mmol, 1.00 equiv.], potassium carbonate (518 mg, 3.75 mmol, 1.25 equiv.) and bromodiphenylmethane (815 mg, 3.30 mmol, 1.10 equiv.) according to **GP 5**. Purification by flash column chromatography on silica gel (cyclohexane:dichloromethane = 1:1) afforded (R_a)-N^2-benzhydryl-[1,1'-binaphthalene]-2,2'-diamine [(R_a)-**4f**, 379 mg, 0.84 mmol, 28%] as an off-white solid.

The NMR spectroscopic data are in agreement with those reported for the racemic amine.

Optical Rotation $[\alpha]_D^{RT}$ = +74.0 (c 0.5, $CHCl_3$, 99% *ee*).
The enantiomeric excess of (R_a)-**4f** was determined by HPLC analysis on a chiral stationary phase (*Daicel Chiralpak*® IA column, column temperature 20°C, mobile phase *n*-heptane:isopropanol = 90:10, flow rate: 0.6 mL/min, λ = 254 nm): t_R =16.2 min (minor), t_R = 18.5 min (major).

(S_a)-N^2-Benzhydryl-[1,1'-binaphthalene]-2,2'-diamine [(S_a)-4f]

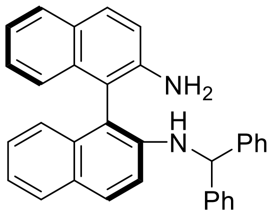

(S_a)-**4f**
$C_{33}H_{26}N_2$
M = 450.59 g/mol

Prepared from (S_a)-BINAM [(S_a)-**4a**, 853 mg, 3.00 mmol, 1.00 equiv.], potassium carbonate (518 mg, 3.75 mmol, 1.25 equiv.) and bromodiphenylmethane (815 mg, 3.30 mmol, 1.10 equiv.) according to **GP 5**. Purification by flash column chromatography on silica gel (cyclohexane:dichloromethane = 1:1) afforded (S_a)-N^2-benzhydryl-[1,1'-binaphthalene]-2,2'-diamine [(S_a)-**4f**, 349 mg, 0.77 mmol, 26%] as an off-white solid.

The NMR spectroscopic data are in agreement with those reported for the racemic sample.

Optical Rotation $[\alpha]_D^{RT}$ = –74.6 (c 0.5, $CHCl_3$, 99% *ee*).

The enantiomeric excess of (S_a)-**4f** was determined by HPLC analysis on a chiral stationary phase (*Daicel Chiralpak*® IA column, column temperature 20°C, mobile phase *n*-heptane:isopropanol = 90:10, flow rate: 0.6 mL/min, λ = 254 nm): t_R =16.2 min (major), t_R = 18.5 min (minor).

***rac*-*N*²-trityl-[1,1'-binaphthalene]-2,2'-diamine (*rac*-4g)**

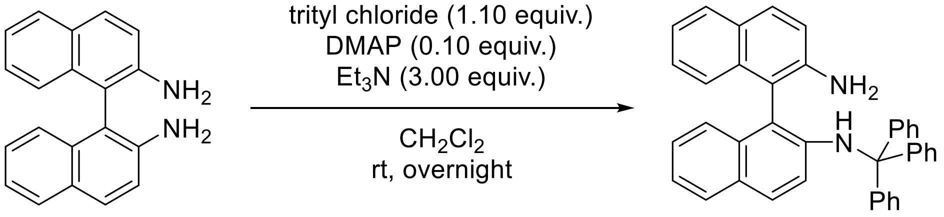

rac-**4a**

rac-**4g**
$C_{39}H_{30}N_2$
M = 526.68 g/mol

A round-bottom flask was charged with *rac*-BINAM (*rac*-**4a**, 853 mg, 3.00 mmol, 1.00 equiv.), trityl chloride (920 mg, 3.30 mmol, 1.10 equiv.) and DMAP (36.7 mg, 0.30 mmol, 0.10 equiv.). Dichloromethane (20 mL) and Et_3N (911 mg, 1.25 mL, 9.00 mmol, 3.00 equiv.) were added, and the resulting solution was stirred at ambient temperature overnight. Then, water (20 mL) was added and the phases separated. The aqueous layer was extracted with dichloromethane (2 × 20 mL) The combined organic layers were washed with brine, dried over anhydrous Na_2SO_4, filtered and concentrated under reduced pressure. The residue was purified by flash column chromatography on silica gel (cyclohexane:dichloromethane:triethylamine = 20:1:1) and subsequently washed with ethanol to afford *rac*-*N*²-trityl-[1,1'-binaphthalene]-2,2'-diamine (*rac*-**4g**, 781 mg, 1.48 mmol, 49%) as a colorless solid.

R_f = 0.23.
^{1}H NMR (500 MHz, C_6D_6) δ/ppm = 7.61 (dd, *J* = 6.7, 3.2 Hz, 1H), 7.57–7.49 (m, 2H), 7.42–7.32 (m, 8H), 7.31–7.26 (m, 1H), 7.12–7.03 (m, 4H), 7.03–6.95 (m, 7H), 6.95–6.87 (m, 3H), 6.61 (dd, *J* = 8.9, 2.9 Hz, 1H), 5.50 (d, *J* = 2.8 Hz, 1H), 3.12 (s, 2H).
^{13}C{^{1}H} NMR (126 MHz, C_6D_6) *δ*/ppm = 146.4, 143.5, 143.2, 134.6, 134.1, 129.9, 129.2, 128.9, 128.6, 128.5, 128.5, 127.2, 127.0, 126.9, 124.7, 124.7, 122.7, 122.5, 118.3, 118.2, 114.7, 112.2, 71.2.
IR (ATR): ṽ/cm^{-1} = 3473 (w), 3376 (w), 1613 (m), 1483 (m), 1284 (m), 811 (s), 772 (m), 749 (vs), 698 (vs).
HRMS (ESI) calculated for $C_{39}H_{31}N_2^+$ [M+H]$^+$: 527.2482; found: 527.2475.

***rac*-2'-(benzhydryloxy)-[1,1'-binaphthalen]-2-ol (*rac*-S2)**

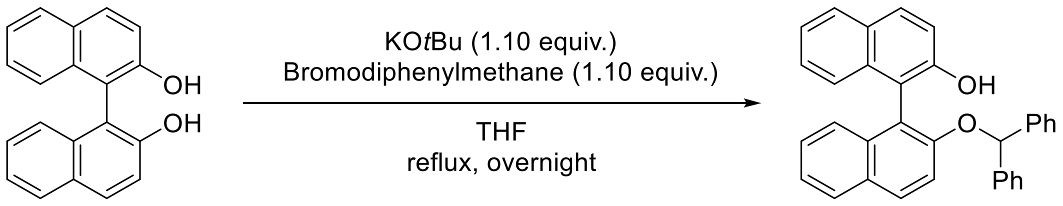

rac-**S2**
$C_{33}H_{24}O_2$
M = 452.55 g/mol

A flame-dried three-neck-flask, fitted with a reflux condenser, was charged with *rac*-BINOL (2.86 g, 10.0 mmol, 1.00 equiv.) and tetrahydrofuran (30 mL) was added. Then potassium *tert*-butoxide (1.23 g, 11.0 mmol, 1.10 equiv.) was added and the reaction mixture was stirred at rt for 5 min. Subsequently, a solution of bromodiphenylmethane (2.72 g, 11.0 mmol, 1.10 equiv.) in tetrahydrofuran (20 mL) was added and the reaction mixture was stirred under reflux overnight. After completion (monitored by TLC), the reaction mixture was cooled down to room temperature and the solvent was removed under reduced pressure. The residue was suspended in dichloromethane (100 mL) and 2 M NaOH (50 mL) was added. The layers were separated, and the aqueous layer was extracted with dichloromethane (2 × 50 mL) The combined organic layers were washed with brine, dried over anhydrous $MgSO_4$, filtered and concentrated under reduced pressure. The residue was purified by flash column chromatography on silica gel (cyclohexane:dichloromethane = 2:1) to afford *rac*-2'-(benzhydryloxy)-[1,1'-binaphthalen]-2-ol (*rac*-**S2**, 2.02 g, 4.46 mmol, 45%) as a colorless solid.

$\mathbf{R_f}$ = 0.15.
^{1}H NMR (500 MHz, C_6D_6) δ/ppm = 7.68 (dd, *J* = 8.4, 5.4 Hz, 2H), 7.59 (d, *J* = 8.1 Hz, 1H), 7.55 (d, *J* = 9.0 Hz, 1H), 7.40 (dd, *J* = 8.7, 4.0 Hz, 2H), 7.26–7.22 (m, 2H), 7.15–7.09 (m, 3H), 7.05–6.89 (m, 8H), 6.89 – 6.83 (m, 3H), 6.07 (s, 1H), 4.91 (s, 1H).
^{13}C{^{1}H} NMR (126 MHz, C_6D_6) δ/ppm 154.5, 152.3, 141.9, 141.7, 134.7, 134.6, 130.7, 130.3, 130.2, 129.7, 128.7, 128.5, 128.5, 128.3, 128.1, 127.9, 127.6, 127.6, 126.9, 126.8, 125.7, 125.6, 124.7, 123.5, 82.6.
IR (ATR): $\tilde{\nu}$/cm^{-1} = 3509 (w), 1589 (m), 1505 (m), 1452 (m), 1237 (s), 1202 (s), 1143 (s), 1015 (m), 806 (s), 743 (vs), 697 (vs).
HRMS (APCI) calculated for $C_{33}H_{24}O_2^+$ $[M]^+$: 452.1771; found: 452.1767.

***rac*-2'-(benzhydryloxy)-[1,1'-binaphthalen]-2-amine (*rac*-4h)**

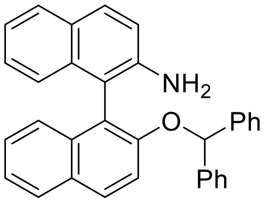

rac-**4h**
$C_{33}H_{25}NO$
M = 451.57 g/mol

Prepared from *rac*-2'-(benzhydryloxy)-[1,1'-binaphthalen]-2-ol (*rac*-**S2**, 905 mg, 2.00 mmol, 1.00 equiv.), potassium iodide (166 mg, 1.00 mmol, 1.00 equiv.), potassium carbonate (276 mg, 2.00 mmol, 1.00 equiv.) and sodium hydroxide (1.00 g, 25.0 mmol, 12.5 equiv.) according to **GP 4**. Purification by flash column chromatography on silica gel (cyclohexane:dichloromethane = 13:7) afforded *rac*-2'-(benzhydryloxy)-[1,1'-binaphthalen]-2-amine (*rac*-**4h**, 383 mg, 0.85 mmol, 42%) as an off-white solid.

R_f = 0.13.
^{1}H NMR (400 MHz, C_6D_6) δ/ppm = 7.72 (dd, *J* = 8.1, 1.4 Hz, 1H), 7.65 (t, *J* = 8.4 Hz, 2H), 7.59 (d, *J* = 9.0 Hz, 1H), 7.50 (dd, *J* = 8.6, 1.1 Hz, 1H), 7.30 (d, *J* = 9.0 Hz, 1H), 7.26 (dd, *J* = 8.7, 1.2 Hz, 1H), 7.15–7.10 (m, 4H), 7.08–6.97 (m, 6H), 6.95–6.85 (m, 4H), 6.72 (d, *J* = 8.7 Hz, 1H), 6.09 (s, 1H), 3.04 (s, 2H).
^{13}C{^{1}H} NMR (101 MHz, C_6D_6) *δ*/ppm = 154.1, 143.0, 142.4, 142.1, 135.1, 134.4, 130.4, 129.7, 129.3, 128.5, 128.4, 127.5, 127.2, 127.1, 127.0, 126.7, 126.0, 125.1, 124.6, 122.3, 122.0, 118.3, 118.0, 113.7, 82.6.
IR (ATR): ṽ/cm^{-1} = 3372 (w), 1616 (m), 1501 (m), 1379 (w), 1349 (w), 1235 (m), 1014 (m), 805 (vs), 741 (vs), 696 (vs).
HRMS (ESI) calculated for $C_{33}H_{26}NO^+$ $[M]^+$: 452.2009; found: 452.2003.

6-Fluoro-2-naphthoic acid (S3)

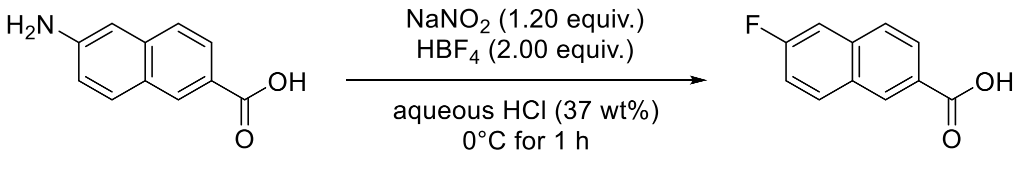

S3
$C_{11}H_7FO_2$
M = 190.17 g/mol

According to a modified literature procedure[S25] 6-amino-2-naphthoic acid (10.0 g, 52.6 mmol, 1.00 equiv.) was suspended in aqueous HCl (37 wt%, 53 mL) and the suspension was cooled to 0°C. A solution of $NaNO_2$ (4.35 g, 63.1 mmol, 1.20 equiv.) in distilled water (10 mL) was added dropwisely. After complete addition the reaction mixture was stirred for an additional 30 min at 0°C. Then, HBF_4 (48 wt% solution in water, 13.8 mL, 105 mmol, 2.00 equiv.) was added portionwise and stirring continued for 30 min at 0°C. Subsequently the reaction mixture was filtered and the solid washed with HBF_4 solution (2 × 10 mL).

The obtained solid was dried under high vacuum for 5 h, then suspended in toluene (200 mL) and stirred under reflux for 13 h. Volatiles were removed under reduced pressure and the residue was partitioned between ethyl acetate (200 mL) and 2 N HCl (100 mL). The phases were separated and the aqueous phase extracted with ethyl acetate (2 × 50 mL). The combined organic layers were washed with brine (50 mL), dried over anhydrous Na_2SO_4, filtered and concentrated under reduced pressure. Recrystallization of the residue (ethanol/water) afforded 6-fluoro-2-naphthoic acid (**S3**, 6.92 g, 36.4 mmol, 69%) as a brown solid.

melting point 234–238 °C.

^{1}H NMR (500 MHz, $(CD_3)_2SO$, 298 K) δ/ppm = 13.09 (s, 1H), 8.65 (s, 1H), 8.22 (dd, J = 9.1, 5.8 Hz, 1H), 8.04–7.97 (m, 2H), 7.80 (dd, J = 10.2, 2.7 Hz, 1H), 7.52 (td, J = 8.9, 2.7 Hz, 1H).

^{13}C{^{1}H} NMR (101 MHz, $(CD_3)_2SO$ 298 K) δ/ppm = 167.4, 161.5 (d, J = 247.1 Hz), 136.1 (d, J = 10.1 Hz), 132.5 (d, J = 9.5 Hz), 130.7, 129.5, 127.9–127.6 (m, 2C), 126.4, 117.17 (d, J = 25.5 Hz), 111.0 (d, J = 20.8 Hz).

^{19}F NMR (471 MHz, $(CD_3)_2SO$, 298 K) δ/ppm = –111.0 (td, J = 9.5, 5.8 Hz).

IR (ATR): $\tilde{v}$/cm^{-1} = 2836 (w, br), 1671 (vs), 1628 (vs), 1476 (vs), 1289 (vs), 1201 (vs), 867 (vs), 803 (vs), 764 (vs).

HRMS (APCI) calculated for $C_{11}H_6FO_2^-$ $[(M-H)]^-$: 189.0357; found: 189.0356.

6-Fluoronaphthalen-2-amine (S4)

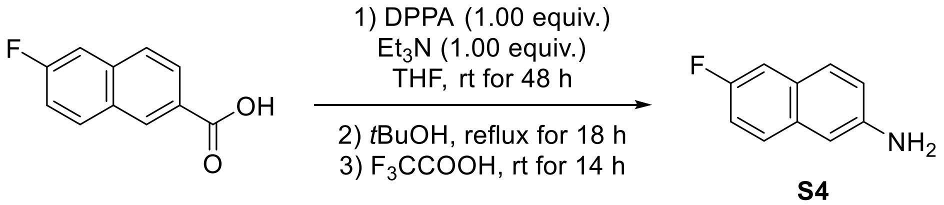

S4

$C_{10}H_8FN$

M = 161.18 g/mol

According to a modified literature procedure[S26] 6-fluoro-2-naphthoic acid (**S3**, 4.75 g, 25.0 mmol, 1.00 equiv.) was dissolved in dry tetrahydrofuran (80 mL) and triethylamine (2.53 g, 3.48 mL, 25.0 mmol, 1.00 equiv.) was added. To this solution diphenylphosphoryl azide (5.38 mL, 6.88 g, 25.0 mmol, 1.00 equiv.) was added and the reaction mixture was stirred at ambient temperature for 48 h. Subsequently, volatiles were removed under reduced pressure, the residue was suspended in *tert*-butanol (90 mL) and the reaction mixture was stirred under reflux for 18 h. After cooling to ambient temperature, the reaction mixture was filtered, the filtrate concentrated under reduced pressure, and the residue dissolved in ethyl acetate (100 mL), water (50 mL) and 1 M NaOH (25 mL). The organic layer was washed with water (2 × 50 mL) and brine (50 mL), dried over anhydrous Na_2SO_4, filtered and concentrated under reduced pressure. The obtained crude was dissolved in trifluoroacetic acid (80 mL) and stirred at ambient temperature for 14 h. Then, aqueous, saturated $NaHCO_3$ solution was added portionwise until the reaction mixture reached pH = 7. The mixture was extracted with dichloromethane (3 × 100 mL).

The combined organic layers were washed with water (50 mL) and brine (50 mL), dried over anhydrous Na_2SO_4, filtered and concentrated under reduced pressure. The residue was purified by flash column chromatography on silica gel (dichloromethane) to afford 6-fluoronaphthalen-2-amine (**S4**, 1.53 g, 9.49 mmol, 38%) as an orange solid.

R_f = 0.42.
^{1}H NMR (500 MHz, $CDCl_3$, 298 K) *δ*/ppm = 7.60 (d, *J* = 9.0 Hz, 1H), 7.56 (dd, *J* = 9.0, 5.5 Hz, 1H), 7.33 (dd, *J* = 9.9, 2.6 Hz, 1H), 7.17 (td, *J* = 8.8, 2.7 Hz, 1H), 6.98 (m_c, 2H), 3.81 (s, 2H).
^{19}F NMR (471 MHz, $CDCl_3$, 298 K) *δ*/ppm = −120.0 (td, *J* = 9.2, 5.5 Hz).
^{13}C{^{1}H} NMR (126 MHz, $CDCl_3$) δ/ppm = 158.9 (d, *J* = 241.3 Hz), 143.6 (d, *J* = 2.4 Hz), 131.9, 128.5 (d, *J* = 5.2 Hz), 128.3 (d, *J* = 8.7 Hz), 127.9 (d, *J* = 8.5 Hz), 119.5, 116.7 (d, *J* = 25.1 Hz), 110.9 (d, *J* = 20.4 Hz), 108.9.
IR (ATR): ṽ/cm^{-1} = 3342 (w), 3324 (w), 3205 (w), 1601 (s), 1509 (s), 1377 (m), 1233 (vs), 1141 (s), 935 (m), 862 (vs), 800 (vs), 675 (m).
HRMS (ESI) calculated for $C_{10}H_9FN^+$ [(M+H)]$^+$: 162.0714; found: 162.0714.

rac-6,6'-Difluoro-[1,1'-binaphthalene]-2,2'-diamine (*rac*-S5)

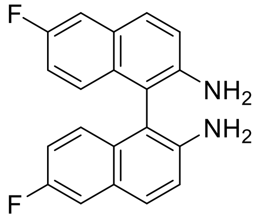

rac-**S5**
$C_{20}H_{14}F_2N_2$
M = 320.34 g/mol

Prepared from 6-fluoronaphthalen-2-amine (**S4**, 1.53 g, 9.49 mmol, 1.00 equiv.), copper(II) chloride dihydrate (3.41 g, 20.0 mmol, 2.11 equiv.) and benzylamine (8.57 g, 8.72 mL, 80.0 mmol, 8.43 equiv.) according to **GP 7**. Purification by flash column chromatography on silica gel (dichloromethane) afforded *rac*-6,6'-difluoro-[1,1'-binaphthalene]-2,2'-diamine (*rac*-**S5**, 890 mg, 2.78 mmol, 59%) as a red solid.

R_f = 0.27.
^{1}H NMR (500 MHz, $CDCl_3$, 298 K) *δ*/ppm = 7.74 (d, *J* = 8.8 Hz, 2H), 7.42 (dd, *J* = 9.7, 2.5 Hz, 2H), 7.17 (d, *J* = 8.8 Hz, 2H), 7.01 (m_c, 4H), 3.65 (s, 4H).
^{19}F NMR (471 MHz, $CDCl_3$, 298 K) *δ*/ppm = −120.4 (td, *J* = 9.0, 5.9 Hz).
^{13}C{^{1}H} NMR (126 MHz, $CDCl_3$) δ/ppm = 159.0 (d, *J* = 242.2 Hz), 142.2 (d, *J* = 2.3 Hz), 130.7, 129.6–128.1 (m, 2C), 126.2 (d, *J* = 8.3 Hz), 119.7, 117.0 (d, *J* = 24.6 Hz), 112.8, 111.5 (d, *J* = 20.3 Hz).
IR (ATR): ṽ/cm^{-1} = 3498 w), 3456 (w), 3399 (m), 3367 (m), 1609 vs), 1509 (s), 1380 (s), 1218 (s), 1143 (m), 1115 (m), 945 (s), 861 (vs), 804 (vs).

HRMS (ESI) calculated for $C_{20}H_{15}F_2N_2^+$ [(M+H)]$^+$: 321.1198; found: 321.1200.

***rac*-*N*²-Benzhydryl-6,6'-difluoro-[1,1'-binaphthalene]-2,2'-diamine (*rac*-4i)**

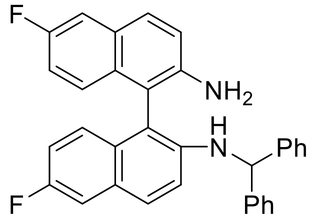

rac-**4i**
$C_{33}H_{24}F_2N_2$
M = 486.57 g/mol

Prepared from *rac*-6,6'-difluoro-[1,1'-binaphthalene]-2,2'-diamine (*rac*-**S5**, 641 mg, 2.00 mmol, 1.00 equiv.), potassium carbonate (346 mg, 2.50 mmol, 1.25 equiv.) and bromodiphenylmethane (544 mg, 2.20 mmol, 1.10 equiv.) according to **GP 5**. Purification by flash column chromatography on silica gel (cyclohexane:dichloromethane = 1:1) afforded *rac*-*N*²-benzhydryl-6,6'-difluoro-[1,1'-binaphthalene]-2,2'-diamine (*rac*-**4i**, 470 mg, 0.97 mmol, 48%) as an off-white solid.

R$_f$ = 0.24
¹H NMR (500 MHz, $CDCl_3$, 298 K) δ/ppm = 7.40 (d, *J* = 9.0 Hz, 1H), 7.28 (dd, *J* = 9.7, 2.7 Hz, 1H), 7.24 (d, *J* = 8.8 Hz, 1H), 7.19–7.14 (m, 4H), 7.08–6.95 (m, 7H), 6.92 (t, *J* = 7.2 Hz, 2H), 6.89–6.81 (m, 3H), 6.52 (d, *J* = 8.8 Hz, 1H), 5.57 (d, *J* = 5.8 Hz, 1H), 4.38 (d, *J* = 5.8 Hz, 1H), 2.98 (s, 2H).
¹⁹F NMR (471 MHz, $CDCl_3$, 298 K) δ/ppm = −120.1 (td, *J* = 9.0, 5.5 Hz), −120.3 (td, *J* = 9.1, 5.6 Hz).
¹³C{1H} NMR (126 MHz, C_6D_6) δ/ppm = 159.3 (d, *J* = 241.9,Hz), 159.3 (d, *J* = 241.9,Hz), 143.4, 143.4, 143.1, 142.8, 131.2, 131.0, 129.1 (m, 3C), 128.9, 128.8, 128.7 (d, *J* = 8.7 Hz), 127.6 (d, *J* = 3.7 Hz), 127.4, 126.9 (d, *J* = 8.2 Hz), 126.6 (d, *J* = 8.2 Hz), 119.4, 117.3 (d, *J* = 24.9 Hz), 117.0 (d, *J* = 24.8 Hz), 116.6, 113.8, 111.9, 111.8 (d, *J* = 20.5 Hz), 111.6 (d, *J* = 20.3 Hz), 63.0.
IR (ATR): ṽ/cm^{-1}= 3375 (w), 3056 (w), 3025 (w), 1603 (vs), 1511 (vs), 1448 (m), 1356 (m), 1236 (s), 1140 (m), 1118 (m), 950 (m), 864 (s), 804 (s), 743 (s), 698 (vs).
HRMS (ESI) calculated for $C_{33}H_{25}F_2N_2^+$ [(M+H)]$^+$: 487.1980 found: 487.1977.

6-Chloronaphthalen-2-amine (S6)

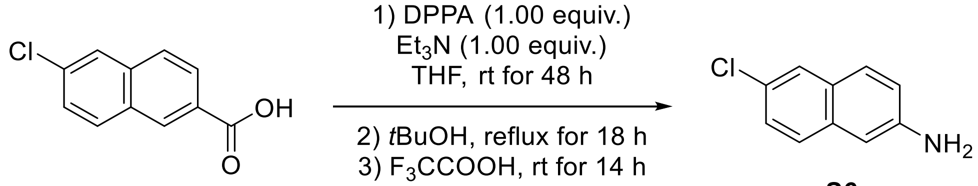

S6
$C_{10}H_8ClN$
M = 177.63 g/mol

According to a modified literature[S26] procedure 6-chloro-2-naphthoic acid (5.17 g, 25.0 mmol, 1.00 equiv.) was suspended in dry tetrahydrofuran (80 mL) and triethylamine (2.53 g, 3.48 mL, 25.0 mmol, 1.00 equiv. was added. To this solution diphenylphosphoryl azide (5.38 mL, 6.88 g, 25.0 mmol, 1.00 equiv.) was added and the reaction mixture was stirred at ambient temperature for 48 h. Subsequently, volatiles were removed under reduced pressure, the residue was suspended in *tert*-butanol (90 mL) and the reaction mixture was stirred under reflux for 18 h. After cooling to ambient temperature, the reaction mixture was filtered, the filtrate concentrated under reduced pressure, and the residue dissolved in ethyl acetate (100 mL) and water (50 mL) and 1 N NaOH (25 mL). The organic layer was washed with water (2 × 50 mL) and brine (50 mL), dried over anhydrous Na_2SO_4, filtered and concentrated under reduced pressure. The obtained crude was dissolved in trifluoroacetic acid (80 mL) and stirred at ambient temperature for 14 h. Then, aqueous, saturated $NaHCO_3$ solution was added portionwise until the reaction mixture reached pH = 7. The mixture was extracted with dichloromethane (3 × 100 mL). The combined organic layers were washed with water (50 mL) and brine (50 mL), dried over anhydrous Na_2SO_4, filtered and concentrated under reduced pressure. The residue was purified by flash column chromatography on silica gel (dichloromethane:cyclohexane) to afford 6-chloronaphthalen-2-amine (**S6**, 1.46 g, 8.22 mmol, 33%) as an orange solid.

R_f = 0.25.
^{1}H NMR (400 MHz, $CDCl_3$, 298 K) δ/ppm = 7.67 (d, J = 2.2 Hz, 1H), 7.57 (d, J = 8.4 Hz, 1H), 7.52 (d, J = 8.8 Hz, 1H), 7.30 (dd, J = 8.8, 2.1 Hz, 1H), 6.96 (m, 2H), 3.87 (s, 2H).
^{13}C{^{1}H} NMR (101 MHz, $CDCl_3$, 298 K) δ/ppm = 144.5, 133.3, 128.5 (2C), 127.9, 127.4, 127.3, 126.5, 119.3, 108.5.
IR (ATR): $\tilde{\nu}$/cm^{-1}= 3441 (w), 3356 (w), 1631 (s), 1591 (s), 1503 (s), 1385 (m), 1283 (m), 1211 (s), 1170 (2), 1072 (m), 910 (m), 886 (s), 864 (vs), 813 (s), 749 (w).
HRMS (ESI) calculated for $C_{10}H_9ClN^+$ [(M+H)]$^+$: 178.0418 found: 178.0418.

6,6'-Dichloro-[1,1'-binaphthalene]-2,2'-diamine (*rac*-S7)

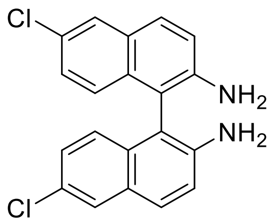

rac-**S7**
$C_{20}H_{14}Cl_2N_2$
M = 353.25 g/mol

Prepared from 6-chloronaphthalen-2-amine (**S6**, 1.42 g, 8.00 mmol, 1.00 equiv.), copper(II) chloride dihydrate (3.41 g, 20.0 mmol, 2.50 equiv.) and benzylamine (8.57 g, 8.72 mL, 80.0 mmol, 10.0 equiv.) according to **GP 7**.

Purification by flash column chromatography on silica gel (dichloromethane:cyclohexane = 3:1) afforded *rac*-6,6'-dichloro-[1,1'-binaphthalene]-2,2'-diamine (*rac*-**S7**, 1.41 g, 3.23 mmol, 81%) as an orange solid.

R_f = 0.22.

^{1}H NMR (400 MHz, $CDCl_3$, 298 K) *δ*/ppm = 7.77 (d, *J* = 2.2 Hz, 2H), 7.72 (d, *J* = 8.8 Hz, 2H), 7.18–7.12 (m, 4H), 6.96 (d, *J* = 8.9 Hz, 2H), 3.71 (s, 4H).

^{13}C{^{1}H} NMR (101 MHz, $CDCl_3$, 298 K) *δ*/ppm = 143.1, 132.0, 129.1, 129.0, 128.2, 127.8, 127.0, 125.6, 119.5, 112.0.

IR (ATR): ṽ/cm^{-1} = 3466 (w), 3372 (m), 1614 (vs), 1493 (vs), 1380 (vs), 1349 (vs), 1077 (m), 925 (m), 878 (s), 806 (vs).

HRMS (ESI) calculated for $C_{20}H_{15}Cl_2N_2^+$ [(M+H)]$^+$: 353.0607 found: 353.0610.

rac-*N*^{2}-Benzhydryl-6,6'-dichloro-[1,1'-binaphthalene]-2,2'-diamine (*rac*-4j)

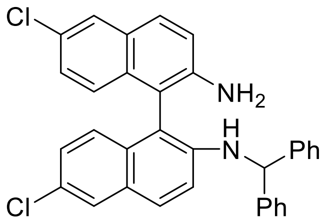

rac-**4j**
$C_{33}H_{24}Cl_2N_2$
M = 519.47 g/mol

Prepared from *rac*-6,6'-dichloro-[1,1'-binaphthalene]-2,2'-diamine (*rac*-**S7**, 707 mg, 2.00 mmol, 1.00 equiv.), potassium carbonate (346 mg, 2.50 mmol, 1.25 equiv.) and bromodiphenylmethane (544 mg, 2.20 mmol, 1.10 equiv.) according to **GP 5**. Purification by flash column chromatography on silica gel (cyclohexane:dichloromethane = 1:1) afforded *rac*-*N*^{2}-benzhydryl-6,6'-dichloro-[1,1'-binaphthalene]-2,2'-diamine (*rac*-**4j**, 520 mg, 1.00 mmol, 50%) as an off-white solid.

R_f = 0.32.

^{1}H NMR (500 MHz, C_6D_6, 298 K) *δ*/ppm = 7.63 (d, *J* = 2.2 Hz, 1H), 7.52 (d, *J* = 2.3 Hz, 1H), 7.34 (d, *J* = 9.0 Hz, 1H), 7.21–7.12 (m, 4H), 7.10 (d, *J* = 9.1 Hz, 1H), 7.08–6.95 (m, 8H), 6.94–6.90 (m, 2H), 6.90–6.84 (m, 1H), 6.45 (d, *J* = 8.9 Hz, 1H), 5.55 (d, *J* = 5.8 Hz, 1H), 4.41 (d, *J* = 5.8 Hz, 1H), 3.01 (s, 2H).

^{13}C{^{1}H} NMR (126 MHz, C_6D_6, 298 K) *δ*/ppm = 144.1, 143.7, 143.3, 142.9, 132.6, 132.3, 129.2, 129.1, 128.9, 128.9, 127.7, 127.6, 127.6, 127.5, 127.4, 127.3, 126.2, 126.0, 119.2, 116.3, 113.1, 111.2, 62.8.

IR (ATR): ṽ/cm^{-1} = 3377 (w), 1588 (vs), 1493 (vs), 1450 (m), 1384 (m), 1348 (s), 1294 (m), 1185 (m), 1077 (m), 927 (m), 874 (m), 815 (s), 742 (s), 698 (vs).

HRMS (ESI) calculated for $C_{33}H_{25}Cl_2N_2^+$ [(M+H)]$^+$: 519.1389 found: 519.1387.

***rac*-6,6'-Dibromo-[1,1'-binaphthalene]-2,2'-diamine (*rac*-S8)**

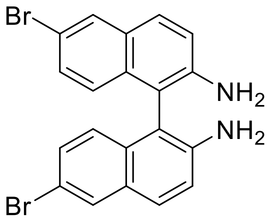

rac-**S8**
$C_{20}H_{14}Br_2N_2$
M = 442.15 g/mol

Prepared from 6-bromonaphthalen-2-amine (2.22 g, 10.0 mmol, 1.00 equiv.), copper(II) chloride dihydrate (3.41 g, 20.0 mmol, 2.00 equiv.) and benzylamine (8.57 g, 8.72 mL, 80.0 mmol, 8.00 equiv.) according to **GP 7**. Purification by flash column chromatography on silica gel (cyclohexane:dichloromethane = 2:3) afforded *rac*-6,6'-dibromo-[1,1'-binaphthalene]-2,2'-diamine (*rac*-**S8**, 1.65 g, 3.72 mmol, 74%) as a yellow solid.

R_f = 0.20.
^{1}H NMR (400 MHz, $CDCl_3$, 298 K) δ/ppm = 7.94 (d, *J* = 2.1 Hz, 2H), 7.71 (d, *J* = 8.8 Hz, 2H), 7.26 (dd, *J* = 9.0, 2.1 Hz, 2H), 7.14 (d, *J* = 8.8 Hz, 2H), 6.89 (d, *J* = 9.0 Hz, 2H), 3.72 (s, 4H).
^{13}C{^{1}H} NMR (101 MHz, $CDCl_3$) δ/ppm = 143.2, 132.3, 130.3, 129.7, 129.0, 125.8, 119.4, 116.2, 111.9.
IR (ATR): $\tilde{\nu}$/cm^{-1} = 3462 (w), 3355 (w), 1611 (s), 1490 (s), 1380 (s), 1348 (s), 1281 (m), 1188 (m), 1066 (m), 820 (s), 873 (s), 809 (vs).
HRMS (APCI) calculated for $C_{20}H_{15}Br_2N_2^+$ $[(M+H)]^+$: 440.9597; found: 440.9592.

The spectroscopic data are in accordance with those reported in the literature.[S24]

***rac*-*N*^{2}-Benzhydryl-6,6'-dibromo-[1,1'-binaphthalene]-2,2'-diamine (*rac*-4k)**

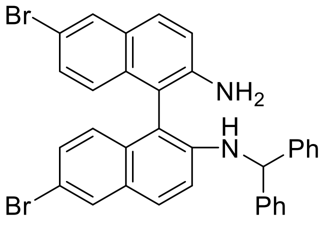

rac-**4k**
$C_{33}H_{24}Br_2N_2$
M = 608.38 g/mol

Prepared from *rac*-6,6'-dibromo-[1,1'-binaphthalene]-2,2'-diamine (*rac*-**S8**, 844 mg, 2.00 mmol, 1.00 equiv.), potassium carbonate (346 mg, 2.50 mmol, 1.25 equiv.) and bromodiphenylmethane (544 mg, 2.20 mmol, 1.10 equiv.) according to **GP 5**. Purification by flash column chromatography on silica gel (cyclohexane:dichloromethane = 2:1) afforded *rac*-*N*^{2}-benzhydryl-6,6'-dibromo-[1,1'-binaphthalene]-2,2'-diamine (*rac*-**4k**, 582 mg, 0.96 mmol, 48%) as an off-white solid.

R$_f$ = 0.14.

^{1}H NMR (500 MHz, C_6D_6) *δ*/ppm = 7.80 (d, *J* = 2.1 Hz, 1H), 7.68 (d, *J* = 2.1 Hz, 1H), 7.32 (d, *J* = 9.0 Hz, 1H), 7.21–7.12 (m, 5H), 7.07 (d, *J* = 9.1 Hz, 1H), 7.02 (t, *J* = 7.5 Hz, 2H), 6.99–6.84 (m, 8H), 6.43 (d, *J* = 8.8 Hz, 1H), 5.54 (d, *J* = 5.8 Hz, 1H), 4.41 (d, *J* = 5.8 Hz, 1H), 3.00 (s, 2H).

^{13}C{^{1}H} NMR (126 MHz, C_6D_6) *δ*/ppm = 144.2, 143.8, 143.2, 142.9, 132.8, 132.5, 130.7, 130.6, 130.5, 130.4, 129.8, 129.5, 129.2, 128.9, 128.9, 127.7, 127.6, 127.6, 127.4, 126.3, 126.1, 119.1, 116.4, 116.3, 113.0, 111.1, 62.8.

IR (ATR): ṽ/cm^{-1} = 3374 (w), 2920 (w), 1585 (vs), 1491 (s), 1450 (w), 1383 (m), 1348 (s), 1185 (m), 1066 (w), 921 (m), 874 (m), 811 (s), 742 (s), 698 (vs).

HRMS (ESI) calculated for $C_{33}H_{25}Br_2N_2^+$ [(M+H)]$^+$: 607.0379; found: 607.0382.

6-Methylnaphthalen-2-amine (S9)

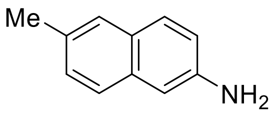

S9
$C_{11}H_{11}N$
M = 157.22 g/mol

Prepared from 6-methylnaphthalen-2-ol (3.16 g, 20.0 mmol, 1.00 equiv.), sodium bisulfite (4.20 g, 40.0 mmol, 2.00 equiv.) and aqueous ammonia (25 wt%, 100 mL) according to **GP 6**. Purification by flash column chromatography on silica gel (dichloromethane:cyclohexane 2:1) afforded 6-methylnaphthalen-2-amine (**S9**, 2.42 g, 15.4 mmol, 77%) as an off-white solid.

melting point 128–132 °C.

R$_f$ = 0.20.

^{1}H NMR (500 MHz, $CDCl_3$, 298 K) *δ*/ppm = 7.59 (d, *J* = 8.6 Hz, 1H), 7.52 (d, *J* = 8.4 Hz, 1H), 7.49 (s, 1H), 7.23 (dd, *J* = 8.4, 1.8 Hz, 1H), 6.97 (d, *J* = 2.3 Hz, 1H), 6.93 (dd, *J* = 8.6, 2.3 Hz, 1H), 3.76 (s, 2H), 2.47 (s, 3H).

^{13}C{^{1}H} NMR (126 MHz, $CDCl_3$, 298 K) *δ*/ppm = 143.5, 133.2, 131.9, 128.7, 128.6, 128.3, 126.8, 125.8, 118.4, 108.8, 21.5.

The spectroscopic data are in accordance with those reported in the literature.[S18]

***rac*-6,6'-Dimethyl-[1,1'-binaphthalene]-2,2'-diamine (*rac*-S10)**

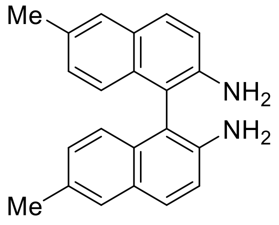

rac-**S10**
$C_{22}H_{20}N_2$
M = 312.42 g/mol

Prepared from 6-methylnaphthalen-2-amine (**S9**, 1.57 g, 10.0 mmol, 1.00 equiv.), copper(II) chloride dihydrate (3.41 g, 20.0 mmol, 2.00 equiv.) and benzylamine (8.57 g, 8.72 mL, 80.0 mmol, 8.00 equiv.) according to **GP 7**. Purification by flash column chromatography on silica gel (dichloromethane) afforded *rac*-6,6'-dimethyl-[1,1'-binaphthalene]-2,2'-diamine (*rac*-**S10**, 858 mg, 2.75 mmol, 55%) as a red solid.

R_f = 0.20.
^{1}H NMR (500 MHz, $CDCl_3$, 298 K) δ/ppm = 7.73 (d, *J* = 8.7 Hz, 2H), 7.58 (s, 2H), 7.12 (d, *J* = 8.7 Hz, 2H), 7.06 (dd, *J* = 8.6, 1.8 Hz, 2H), 7.00 (d, *J* = 8.6 Hz, 2H), 3.62 (s, 4H), 2.44 (s, 6H).
^{13}C{^{1}H} NMR (126 MHz, $CDCl_3$, 298 K) δ/ppm = 142.1, 132.0, 131.9, 129.1, 128.9, 128.8, 127.4, 124.1, 118.6, 113.1, 21.4.

The spectroscopic data are in accordance with those reported in the literature.[S24]

***rac*-N^2-Benzhydryl-6,6'-dimethyl-[1,1'-binaphthalene]-2,2'-diamine (*rac*-4l)**

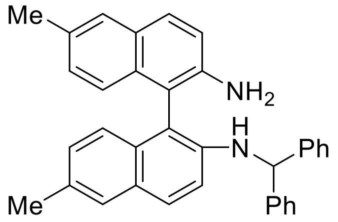

rac-**4l**
$C_{35}H_{30}N_2$
M = 478.64 g/mol

Prepared from *rac*-6,6'-dimethyl-[1,1'-binaphthalene]-2,2'-diamine (*rac*-**S10**, 625 mg, 2.00 mmol, 1.00 equiv.), potassium carbonate (346 mg, 2.50 mmol, 1.25 equiv.) and bromodiphenylmethane (544 mg, 2.20 mmol, 1.10 equiv.) according to **GP 5**. Purification by flash column chromatography on silica gel (cyclohexane:dichloromethane = 1:1) afforded *rac*-N^2-benzhydryl-6,6'-dimethyl-[1,1'-binaphthalene]-2,2'-diamine (*rac*-**4l**, 461 mg, 0.97 mmol, 48%) as an off-white solid.

R_f =0.23.

^{1}H NMR (500 MHz, C_6D_6, 298 K) *δ*/ppm = 7.63 (d, *J* = 8.9 Hz, 1H), 7.50 (d, *J* = 8.7 Hz, 1H), 7.46 (s, 1H), 7.37 (s, 1H), 7.33 (d, *J* = 8.5 Hz, 1H), 7.28 (d, *J* = 8.6 Hz, 1H), 7.24 (d, *J* = 9.0 Hz, 1H), 7.21 (m_c, 2H), 7.11 (m_c, 2H), 7.02 (td, *J* = 7.2, 6.3, 1.3 Hz, 2H), 6.97–6.91 (m, 5H), 6.90 –6.85 (m, 1H), 6.64 (d, *J* = 8.7 Hz, 1H), 5.66 (d, *J* = 5.2 Hz, 1H), 4.56 (d, *J* = 5.7 Hz, 1H), 3.12 (s, 2H), 2.23 (s, 3H), 2.20 (s, 3H).

^{13}C{^{1}H} NMR (126 MHz, C_6D_6, 298 K) *δ*/ppm = 143.9, 143.6, 143.4, 142.8, 132.9, 132.6, 131.6, 129.4, 129.3, 129.2, 129.1, 129.1, 128.9, 128.8, 127.7, 127.6, 127.4, 127.3, 124.8, 124.7, 118.4, 115.6, 114.3, 112.5, 63.1, 21.3, 21.3.

IR (ATR): ṽ/cm^{-1} = 3371 (w), 3023 (w), 2914 (w), 1596 (vs), 1490 (s), 1449 (m), 1350 (m), 1283 (m), 817 (vs), 742 (s), 698 (vs).

HRMS (ESI) calculated for $C_{35}H_{31}N_2^+$ [(M+H)]$^+$: 479.2482 found: 479.2478.

6-Isopropylnaphthalen-2-amine (S11)

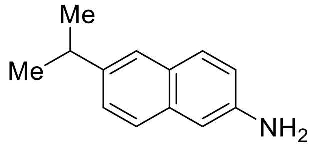

S11
$C_{13}H_{15}N$
M = 185.27 g/mol

Prepared from 6-isopropylnaphthalen-2-ol (3.72 g, 20.0 mmol, 1.00 equiv.), sodium bisulfite (4.20 g, 40.0 mmol, 2.00 equiv.) and aqueous ammonia (25 wt%, 100 mL) according to **GP 6**. Purification by recrystallization (*n*-heptane) afforded 6-isopropylnaphthalen-2-amine (**S11**, 3.01 g, 16.2 mmol, 81%) as a beige solid.

melting point 64–68 °C.

^{1}H NMR (400 MHz, $CDCl_3$, 298 K) *δ*/ppm = 7.64 (d, *J* = 8.6 Hz, 1H), 7.57 (d, *J* = 8.5 Hz, 1H), 7.50 (s, 1H), 7.32 (dd, *J* = 8.3, 2.0 Hz, 1H), 6.98 (d, *J* = 2.3 Hz, 1H), 6.94 (dd, *J* = 8.6, 2.3 Hz, 1H), 3.77 (s, 2H), 3.03 (p, *J* = 6.8 Hz, 1H), 1.35 (d, *J* = 6.8 Hz, 6H).

^{13}C{^{1}H} NMR (101 MHz, $CDCl_3$, 298 K) *δ*/ppm = 143.6, 143.0, 133.5, 129.0, 128.3, 126.3, 125.9, 124.1, 118.4, 108.8, 34.1, 24.1.

IR (ATR): ṽ/cm^{-1} = 3432 (m), 3330 (m), 2950 (m), 2860 (m), 1613 (s), 1505 (s), 1459 (s), 1378 (m), 1279 (s), 1188 (s), 1039 (m), 871 (vs), 807 (vs), 668 (s).

HRMS (ESI) calculated for $C_{13}H_{16}N^+$ [(M+H)]$^+$: 186.1277; found: 186.1277.

***rac*-6,6'-Diisopropyl-[1,1'-binaphthalene]-2,2'-diamine (*rac*-S12)**

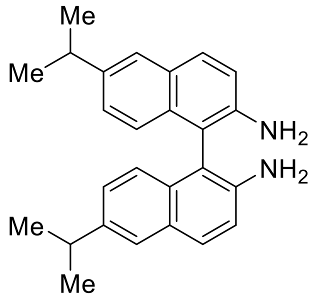

rac-**S12**
$C_{26}H_{28}N_2$
M = 368.52 g/mol

Prepared from 6-isopropylnaphthalen-2-amine (**S11**, 1.85 g, 10.0 mmol, 1.00 equiv.), copper(II) chloride dihydrate (3.41 g, 20.0 mmol, 2.00 equiv.) and benzylamine (8.57 g, 8.72 mL, 80.0 mmol, 8.00 equiv.) according to **GP 7**. Purification by flash column chromatography on silica gel (dichloromethane) afforded *rac*-6,6’-diisopropyl-[1,1'-binaphthalene]-2,2'-diamine (*rac*-**S12**, 470 mg, 1.27 mmol, 25%) as an off-white solid.

$\mathbf{R_f}$ = 0.18.
^{1}H NMR (500 MHz, $CDCl_3$, 298 K) *δ*/ppm = 7.76 (dd, *J* = 8.8, 2.7 Hz, 2H), 7.61 (s, 2H), 7.15–7.10 (m, 4H), 7.08–7.03 (m, 2H), 3.62 (s, 4H), 3.06–2.95 (m, 2H), 1.35–1.26 (m, 12H).
^{13}C{^{1}H} NMR (126 MHz, $CDCl_3$, 298 K) *δ*/ppm = 142.9, 142.2, 132.4, 129.2, 128.8, 126.8, 124.5, 124.2, 118.4, 113.1, 33.9, 24.1, 24.0.
IR (ATR): $\tilde{\nu}$/cm^{-1} = 3458 (w), 3364 (w), 3163 (w), 2956 (m), 1605 (vs), 1479 (m), 1379 (s), 1282 (m), 826 (vs), 744 (m), 689 (vs).
HRMS (ESI) calculated for $C_{26}H_{29}N_2^+$ [(M+H)]$^+$: 369.2325; found: 369.2326.

***rac*-*N*^{2}-Benzhydryl-6,6'-diisopropyl-[1,1'-binaphthalene]-2,2'-diamine (*rac*-4m)**

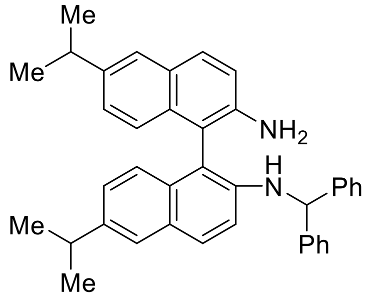

rac-**4m**
$C_{39}H_{38}N_2$
M = 534.75 g/mol

Prepared from *rac*-6,6'-diisopropyl-[1,1'-binaphthalene]-2,2'-diamine (*rac*-**S12**, 405 mg, 1.10 mmol, 1.00 equiv.), potassium carbonate (190 mg, 1.38 mmol, 1.25 equiv.) and bromodiphenylmethane (299 mg, 1.21 mmol, 1.10 equiv.) according to **GP 5**.

Purification by flash column chromatography on silica gel (cyclohexane:dichloromethane = 1:1) afforded *rac*-N^2-benzhydryl-6,6'-diisopropyl-[1,1'-binaphthalene]-2,2'-diamine (*rac*-**4m**, 285 mg, 0.53 mmol, 48%) as a yellow solid.

R_f = 0.25.
^{1}H NMR (500 MHz, C_6D_6, 298 K) *δ*/ppm = 7.70 (dd, *J* = 9.0, 2.6 Hz, 1H), 7.60–7.54 (m, 2H), 7.51 (s, 1H), 7.35–7.30 (m, 1H), 7.30–7.24 (m, 2H), 7.21 (d, *J* = 7.3 Hz, 2H), 7.08 (d, *J* = 7.2 Hz, 2H), 7.05–6.86 (m, 8H), 6.66 (dd, *J* = 8.8, 2.9 Hz, 1H), 5.73–5.63 (m, 1H), 4.57 (d, *J* = 6.7 Hz, 1H), 3.12 (s, 2H), 2.88–2.74 (m, 2H), 1.26–1.11 (m, 12H).
^{13}C{^{1}H} NMR (126 MHz, C_6D_6, 298 K) *δ*/ppm = 143.7, 143.5, 143.0, 142.8, 142.7, 133.3, 133.1, 129.5, 129.4, 129.1, 128.8, 128.7, 127.6, 127.4, 127.3, 126.9, 125.1, 125.0, 124.9, 118.3, 115.5, 114.4, 112.5, 63.1, 34.3, 34.2, 24.2, 24.1.
IR (ATR): ṽ/cm^{-1} = 3368 (w), 2954 (m), 2035 (w), 1595 (vs), 1490 (vs), 1451 (m), 1296 (m), 1177 (w), 823 (m), 742 (m), 697 (vs).
HRMS (ESI) calculated for $C_{39}H_{39}N_2^+$ [(M+H)]$^+$: 535.3108; found: 535.3101.

6-Phenylnaphthalen-2-amine (S13)

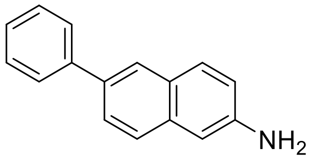

S13
$C_{16}H_{13}N$
M = 219.29 g/mol

Prepared from 6-phenylnaphthalen-2-ol (4.40 g, 20.0 mmol, 1.00 equiv.), sodium bisulfite (4.20 g, 40.0 mmol, 2.00 equiv.) and aqueous ammonia (25 wt%, 100 mL) according to **GP 6**. Purification by recrystallization (toluene) afforded 6-phenylnaphthalen-2-amine (**S13**, 2.56 g, 11.7 mmol, 58%) as a beige solid.

melting point 128–132 °C.
^{1}H NMR (500 MHz, $CDCl_3$, 298 K) *δ*/ppm = 7.93 (s, 1H), 7.72 (t, *J* = 8.6 Hz, 3H), 7.68 (s, 2H), 7.48 (t, *J* = 7.6 Hz, 2H), 7.36 (t, *J* = 7.4 Hz, 1H), 7.01 (d, *J* = 2.4 Hz, 1H), 6.98 (dd, *J* = 8.6, 2.4 Hz, 1H), 3.87 (s, 2H).
^{13}C{^{1}H} NMR (126 MHz, $CDCl_3$, 298 K) *δ*/ppm = 144.4, 141.6, 135.3, 134.3, 129.7, 128.9, 128.3, 127.2, 127.0, 126.4, 126.2, 125.8, 118.8, 108.5.

The spectroscopic data are in accordance with those reported in the literature.[S18]

rac-6,6'-Diphenyl-[1,1'-binaphthalene]-2,2'-diamine (rac-S14)

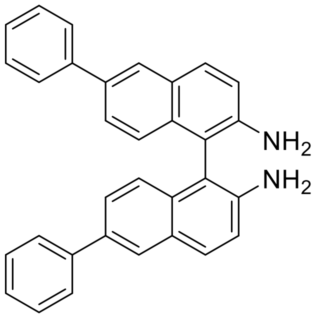

rac-**S14**
$C_{32}H_{24}N_2$
M = 436.56 g/mol

Prepared from 6-phenylnaphthalen-2-amine (**S13**, 2.19 g, 10.0 mmol, 1.00 equiv.), copper(II) chloride dihydrate (3.41 g, 20.0 mmol, 2.00 equiv.) and benzylamine (8.57 g, 8.72 mL, 80.0 mmol, 8.00 equiv.) according to **GP 7**. Purification by flash column chromatography on silica gel (dichloromethane) afforded *rac*-6,6'-diphenyl-[1,1'-binaphthalene]-2,2'-diamine (*rac*-**S14**, 1.47 g, 3.37 mmol, 67%) as a pink solid.

R_f = 0.39.
^{1}H NMR (500 MHz, $CDCl_3$, 298 K) δ/ppm = 8.02 (d, J = 2.0 Hz, 2H), 7.89 (d, J = 8.7 Hz, 2H), 7.70–7.63 (m, 4H), 7.51 (dd, J = 8.8, 2.0 Hz, 2H), 7.48–7.41 (m, 4H), 7.35–7.29 (m, 2H), 7.20 (dd, J = 8.8, 2.4 Hz, 4H), 3.77 (s, 4H).
^{13}C{^{1}H} NMR (101 MHz, $CDCl_3$, 298 K) δ/ppm = 143.1, 141.4, 135.4, 133.1, 130.1, 128.9, 128.9, 127.2, 127.0, 126.7, 126.4, 124.7, 118.9, 112.5.
HRMS (ESI) calculated for $C_{32}H_{25}N_2^+$ $[(M+H)]^+$: 437.2012; found: 437.2009.

The spectroscopic data are in accordance with those reported in the literature.[S28]

rac-N^2-Benzhydryl-6,6'-diphenyl-[1,1'-binaphthalene]-2,2'-diamine (rac-4n)

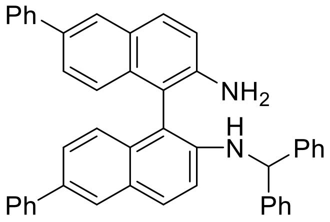

rac-**4n**
$C_{45}H_{34}N_2$
M = 602.78 g/mol

Prepared from *rac*-6,6'-diphenyl-[1,1'-binaphthalene]-2,2'-diamine (*rac*-**S14**, 873 mg, 2.00 mmol, 1.00 equiv.), potassium carbonate (346 mg, 2.50 mmol, 1.25 equiv.) and bromodiphenylmethane (544 mg, 2.20 mmol, 1.10 equiv.) according to **GP 5**.

Purification by flash column chromatography on silica gel (cyclohexane:dichloromethane = 1:1) afforded *rac*-N^2-benzhydryl-6,6'-diphenyl-[1,1'-binaphthalene]-2,2'-diamine (*rac*-**4n**, 451 mg, 0.75 mmol, 37%) as an off-white solid.

R_f = 0.16.

^{1}H NMR (500 MHz, C_6D_6, 298 K) δ/ppm = 8.00 (d, J = 1.8 Hz, 1H), 7.90 (d, J = 1.8 Hz, 1H), 7.73 (d, J = 9.0 Hz, 1H), 7.60–7.53 (m, 5H), 7.49–7.43 (m, 3H), 7.40 (d, J = 8.8 Hz, 1H), 7.29–7.21 (m, 6H), 7.14–7.08 (m, 5H), 7.04 (t, J = 7.7 Hz, 2H), 6.99–6.95 (m, 1H), 6.92 (t, J = 7.5 Hz, 2H), 6.88–6.82 (m, 1H), 6.65 (d, J = 8.7 Hz, 1H), 5.69 (d, J = 5.8 Hz, 1H), 4.67 (d, J = 5.9 Hz, 1H), 3.21 (s, 2H).

^{13}C{^{1}H} NMR (126 MHz, C_6D_6, 298 K) δ/ppm = 144.2, 143.7, 143.6, 143.3, 141.9, 135.6, 133.9, 133.6, 130.3, 130.3, 129.1, 129.1, 129.1, 128.9, 128.9, 128.8, 127.7, 127.6, 127.5, 127.0, 126.9, 126.8, 126.7, 125.4, 125.2, 118.7, 115.9, 113.7, 111.9, 63.0.

IR (ATR): ṽ/cm^{-1} = 3374 (w), 3023 (w), 1582 (s), 1490 (s), 1441 (m), 1293 (m), 1182 (w), 754 (s), 694 (vs).

HRMS (ESI) calculated for $C_{45}H_{35}N_2^+$ [(M+H)]$^+$: 603.2795; found: 603.2789.

***rac*-6,6'-Bis(trimethylsilyl)-[1,1'-binaphthalene]-2,2'-diamine (*rac*-S15)**

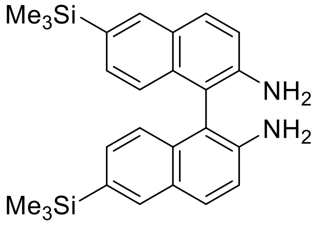

rac-**S15**
$C_{26}H_{32}N_2Si_2$
M = 428.73 g/mol

According to a literature procedure[S27] a flame-dried Schlenk flask was charged with *rac*-6,6'-dibromo-[1,1'-binaphthalene]-2,2'-diamine (*rac*-**S8**, 1.24 g, 2.80 mmol, 1.00 equiv.) and dry tetrahydrofuran (28 mL). The reaction mixture was cooled down to −78°C and *n*-BuLi (2.5 M in hexanes, 11.2 mL, 28.0 mmol, 10.0 equiv.) was added dropwise. After complete addition, the reaction mixture was stirred a further 3 h at −78°C. Then trimethylsilyl chloride (1.83 g, 2.14 mL, 16.8 mmol, 6.00 equiv.) was added dropwise, and stirring continued at −78°C for 30 min. Subsequently the reaction mixture was allowed to warm to ambient temperature and stirred for 14 h. Then, 1 N aqueous HCl (22 mL) was added dropwise and stirring continued for 1 h. The mixture was extracted with ethyl acetate (3 × 75 mL). The combined organic layers were washed with brine (50 mL), dried over anhydrous Na_2SO_4, filtrated and volatiles were removed under reduced pressure. The residue was purified by flash column chromatography on silica gel (dichloromethane:cyclohexane = 9:1) to afford *rac*-6,6'-bis(trimethylsilyl)-[1,1'-binaphthalene]-2,2'-diamine (*rac*-**S15**, 618 mg, 1.44 mmol, 51%) as a beige solid.

R$_f$ = 0.21.

^{1}H NMR (400 MHz, $CDCl_3$, 298 K) *δ*/ppm = 7.96 (s, 2H), 7.81 (d, *J* = 8.8 Hz, 2H), 7.33 (dd, *J* = 8.4, 1.3 Hz, 2H), 7.14 (d, *J* = 8.7 Hz, 2H), 7.09 (d, *J* = 8.3 Hz, 2H), 3.71 (s, 4H), 0.30 (s, 18H).

^{13}C{^{1}H} NMR (101 MHz, $CDCl_3$, 298 K) *δ*/ppm = 143.2, 134.2, 134.1, 133.5, 131.2, 129.9, 128.2, 123.2, 118.3, 112.5, −0.9.

^{29}Si DEPT NMR (99 MHz, $CDCl_3$, 298 K) *δ*/ppm = −4.3

The spectroscopic data are in accordance with those reported in the literature.[S27]

***rac*-*N*^{2}-Benzhydryl-6,6'-bis(trimethylsilyl)-[1,1'-binaphthalene]-2,2'-diamine (*rac*-4o)**

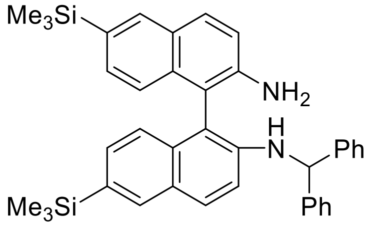

rac-**4o**
$C_{39}H_{42}N_2Si_2$
M = 594.95 g/mol

Prepared from *rac*-6,6'-bis(trimethylsilyl)-[1,1'-binaphthalene]-2,2'-diamine (*rac*-**S15**, 600 mg, 1.40 mmol, 1.00 equiv.), potassium carbonate (247 mg, 1.79 mmol, 1.25 equiv.) and bromodiphenylmethane (389 mg, 1.57 mmol, 1.10 equiv.) according to **GP 5**. Purification by flash column chromatography on silica gel (cyclohexane:dichloromethane = 1:1) afforded *rac*-*N*^{2}-benzhydryl-6,6'-bis(trimethylsilyl)-[1,1'-binaphthalene]-2,2'-diamine (*rac*-**4o**, 386 mg, 0.65 mmol, 46%) as an off-white solid.

R$_f$ = 0.31.

^{1}H NMR (500 MHz, C_6D_6, 298 K) *δ*/ppm = 8.07 (s, 1H), 7.97 (s, 1H), 7.74 (d, *J* = 9.0 Hz, 1H), 7.59 (d, *J* = 8.7 Hz, 1H), 7.35 (d, *J* = 8.3 Hz, 1H), 7.31–7.23 (m, 4H), 7.20 (dd, *J* = 7.4, 1.7 Hz, 2H), 7.06 (m_c, 2H), 7.02 (t, *J* = 7.6 Hz, 2H), 6.98–6.84 (m, 4H), 6.63 (d, *J* = 8.7 Hz, 1H), 5.68 (d, *J* = 5.9 Hz, 1H), 4.65 (d, *J* = 6.0 Hz, 1H), 3.19 (s, 2H), 0.28 (s, 9H), 0.26 (s, 9H).

^{13}C{^{1}H} NMR (126 MHz, C_6D_6, 298 K) *δ*/ppm = 144.4, 143.9, 143.7, 143.3, 135.1, 134.7, 134.6, 133.2, 133.2, 131.4, 131.3, 130.2, 130.1, 128.9, 128.7, 128.5, 127.7, 127.6, 127.5, 127.4, 124.1, 124.0, 118.1, 115.3, 113.8, 111.9, 62.9, −0.9.

^{29}Si DEPT NMR (99 MHz, C_6D_6, 298 K) *δ*/ppm = −4.8.

IR (ATR): ṽ/cm^{-1} = 2950 (w), 1611 (s), 1475 (s), 1245 (s), 1088 (m), 899 (m), 834 (vs), 746 (s), 667 (vs).

HRMS (ESI) calculated for $C_{39}H_{43}N_2Si_2^+$ $[(M+H)]^+$: 595.2959; found: 595.2958.

6-Methoxynaphthalen-2-amine (S16)

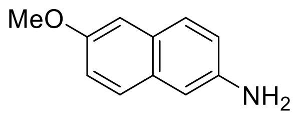

S16
$C_{11}H_{11}NO$
M = 173.22 g/mol

Prepared from 6-methoxynaphthalen-2-ol (2.09 g, 12.0 mmol, 1.00 equiv.), sodium bisulfite (2.52 g, 24.0 mmol, 2.00 equiv.) and aqueous ammonia (25 wt%, 60 mL) according to **GP 6**. Purification by flash column chromatography on silica gel (dichloromethane) afforded 6-methoxynaphthalen-2-amine (**S16**, 1.66 g, 9.58 mmol, 80%) as a pink solid.

R_f = 0.33.
^{1}H NMR (400 MHz, $CDCl_3$) *δ*/ppm = 7.58 (d, *J* = 8.5 Hz, 1H), 7.52 (d, *J* = 8.8 Hz, 1H), 7.11–7.03 (m, 2H), 6.99–6.92 (m, 2H), 3.89 (s, 3H), 3.73 (s, 2H).
^{13}C{^{1}H} NMR (101 MHz, $CDCl_3$) *δ*/ppm = 155.5, 142.4, 130.3, 128.8, 128.0, 127.5, 119.1, 118.9, 109.4, 106.2, 55.4.

The spectroscopic data are in accordance with those reported in the literature.[S29]

***rac*-6,6'-Dimethoxy-[1,1'-binaphthalene]-2,2'-diamine (*rac*-S17)**

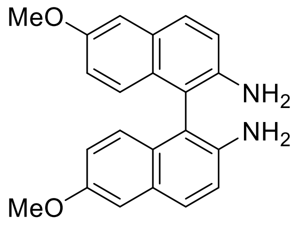

rac-**S17**
$C_{22}H_{20}N_2O_2$
M = 344.41 g/mol

Prepared from 6-methoxynaphthalen-2-amine (**S16**, 1.73 g, 10.0 mmol, 1.00 equiv.), copper(II) chloride dihydrate copper(II) chloride dihydrate (3.41 g, 20.0 mmol, 2.00 equiv.) and benzylamine (8.57 g, 8.72 mL, 80.0 mmol, 8.00 equiv.) according to **GP 7**. Purification by flash column chromatography on silica gel (dichloromethane:ethyl acetate = 20:1) afforded *rac*-6,6'-dimethoxy-[1,1'-binaphthalene]-2,2'-diamine (*rac*-**S17**, 1.35 g, 3.92 mmol, 78%) as a red solid.

R_f = 0.33.
^{1}H NMR (500 MHz, $CDCl_3$, 298 K) *δ*/ppm = 7.71 (d, *J* = 8.7 Hz, 2H), 7.18–7.09 (m, 4H), 7.00 (d, *J* = 9.1 Hz, 2H), 6.90 (dd, *J* = 9.2, 2.7 Hz, 2H), 3.88 (s, 6H), 3.57 (s, 4H).
^{13}C{^{1}H} NMR (126 MHz, $CDCl_3$, 298 K) *δ*/ppm = 155.6, 141.1, 129.4, 129.2, 128.3, 125.8, 119.2, 119.1, 113.7, 107.0, 55.5.

The spectroscopic data are in accordance with those reported in the literature.[S24]

*rac-N*²-Benzhydryl-6,6'-dimethoxy-[1,1'-binaphthalene]-2,2'-diamine (*rac*-4p)

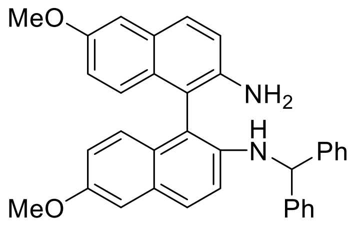

rac-**4p**
$C_{35}H_{30}N_2O_2$
M = 510.64 g/mol

Prepared from *rac*-6,6'-dimethoxy-[1,1'-binaphthalene]-2,2'-diamine (*rac*-**S17**, 689 mg, 2.00 mmol, 1.00 equiv.), potassium carbonate (346 mg, 2.50 mmol, 1.25 equiv.) and bromodiphenylmethane (544 mg, 2.20 mmol, 1.10 equiv.) according to **GP 6**. Purification by automatic column chromatography (cyclohexane:ethyl acetate = 97:3 → 80:20) afforded *rac-N*²-benzhydryl-6,6'-dimethoxy-[1,1'-binaphthalene]-2,2'-diamine (*rac*-**4p**, 155 mg, 0.30 mmol, 15%) as an orange solid.

R$_f$ = 0.33 (cyclohexane:dichloromethane = 3:1).
¹H NMR (500 MHz, C_6D_6) *δ*/ppm 7.64 (d, *J* = 8.8 Hz, 1H), 7.48 (d, *J* = 8.7 Hz, 1H), 7.25 (m$_c$, 5H), 7.11 (d, *J* = 7.5 Hz, 2H), 7.06–7.00 (m, 3H), 7.00–6.91 (m, 5H), 6.87 (m$_c$, 1H), 6.68 (d, *J* = 8.7 Hz, 1H), 5.67 (s, 1H), 4.50 (s, 1H), 3.40 (s, 3H), 3.39 (s, 3H), 3.10 (s, 2H).
¹³C{¹H} NMR (126 MHz, C_6D_6) *δ*/ppm = 156.1, 156.1, 143.9, 143.6, 142.4, 141.8, 129.9, 129.8, 129.7, 129.4, 128.9, 128.8, 128.6, 128.4, 127.8, 127.6, 127.4, 127.3, 126.6, 126.4, 119.9, 119.6, 118.9, 116.1, 115.0, 113.1, 107.4, 106.9, 63.3, 54.9, 54.8.
IR (ATR): $\tilde{\nu}$/cm^{-1} = 3357 (w), 2923 (w), 1598 (vs), 1506 (m), 1450 (s), 1370 (s), 1233 (vs), 1165 (s), 1029 (s), 943 (m), 846 (s), 820 (s), 743 (m), 698 (vs).
HRMS (ESI) calculated for $C_{35}H_{31}N_2O_2^+$ [(M+H)]$^+$: 511.2380; found: 511.2379.

7-Bromonaphthalen-2-amine (S18)

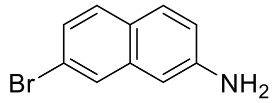

S18
$C_{10}H_8BrN$
M = 222.09 g/mol

Prepared from 7-bromonaphthalen-2-ol (4.46 g, 20.0 mmol, 1.00 equiv.), sodium bisulfite (4.20 g, 40.0 mmol, 2.00 equiv.) and aqueous ammonia (25 wt%, 100 mL) according to **GP 6**. Purification by flash column chromatography on silica gel (cyclohexane:dichloromethane = 1:1) afforded 7-bromonaphtalen-2-amine (**S18**, 3.07 g, 13.8 mmol, 69%) as a pink solid.

R$_f$ = 0.34.

^{1}H NMR (400 MHz, $CDCl_3$) *δ*/ppm = 7.74 (d, *J* = 2.0 Hz, 1H), 7.61 (d, *J* = 8.7 Hz, 1H), 7.54 (d, *J* = 8.6 Hz, 1H), 7.28 (dd, *J* = 8.6, 2.0 Hz, 1H), 6.93 (dd, *J* = 8.7, 2.3 Hz, 1H), 6.86 (d, *J* = 2.3 Hz, 1H), 3.90 (s, 2H).

^{13}C{^{1}H} NMR (101 MHz, $CDCl_3$) *δ*/ppm = 145.1, 136.3, 129.5, 129.4, 127.8, 126.4, 125.8, 120.7, 118.6, 107.5.

The spectroscopic data are in accordance with those reported in the literature.[S18]

***rac*-7,7'-Dibromo-[1,1'-binaphthalene]-2,2'-diamine (*rac*-S19)**

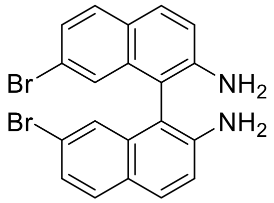

rac-**S19**
$C_{20}H_{14}Br_2N_2$
M = 442.15 g/mol

Prepared from 7-bromonaphthalen-2-amine (**S18**, 2.22 g, 10.0 mmol, 1.00 equiv.), copper(II) chloride dihydrate (3.41 g, 20.0 mmol, 2.00 equiv.) and benzylamine (8.57 g, 8.72 mL, 80.0 mmol, 8.00 equiv.) according to **GP 7**. Purification by flash column chromatography on silica gel (cyclohexane:dichloromethane = 2:3) afforded *rac*-7,7'-dibromo-[1,1'-binaphthalene]-2,2'-diamine (*rac*-**S19**, 1.18 g, 2.67 mmol, 53%) as a yellow solid.

R$_f$ = 0.21.

^{1}H NMR (500 MHz, $CDCl_3$) *δ*/ppm = 7.77 (d, *J* = 8.6 Hz, 2H), 7.65 (d, *J* = 8.6 Hz, 2H), 7.32 (dd, *J* = 8.6, 2.0 Hz, 2H), 7.18 (d, *J* = 1.9 Hz, 2H), 7.13 (d, *J* = 8.8 Hz, 2H), 3.73 (s, 4H).

^{13}C{^{1}H} NMR (126 MHz, $CDCl_3$) *δ*/ppm = 143.9, 135.0, 130.1, 129.9, 127.0, 126.0, 125.7, 121.9, 118.8, 110.7.

IR (ATR): ṽ/cm^{-1} = 3454 (w), 3364 (w), 1608 (vs), 1492 (vs), 1445 (m), 1378 (m), 1345 (s), 1259 (s), 1198 (w), 1147 (s), 1065 (s), 933 (s), 867 (m), 823 (vs).

HRMS (APCI) calculated for $C_{20}H_{15}Br_2N_2^+$ [(M+H)]$^+$: 440.9597; found: 440.9593.

***rac*-*N*^{2}-Benzhydryl-7,7'-dibromo-[1,1'-binaphthalene]-2,2'-diamine (*rac*-4q)**

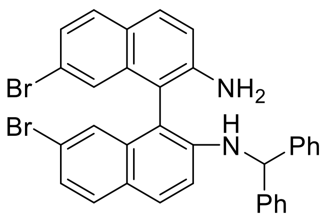

rac-**4q**
$C_{33}H_{24}Br_2N_2$
M = 608.38 g/mol

Prepared from *rac*-7,7'-dibromo-[1,1'-binaphthalene]-2,2'-diamine (*rac*-**S19**, 844 mg, 2.00 mmol, 1.00 equiv.), potassium carbonate (346 mg, 2.50 mmol, 1.25 equiv.) and bromodiphenylmethane (544 mg, 2.20 mmol, 1.10 equiv.) according to **GP 6**. Purification by flash column chromatography on silica gel (cyclohexane:dichloromethane = 2:1) afforded *rac*-N^2-benzhydryl-7,7'-dibromo-[1,1'-binaphthalene]-2,2'-diamine (*rac*-**4q**, 640 mg, 1.05 mmol, 53%) as an off-white solid.

R_f = 0.25.
^{1}H NMR (500 MHz, C_6D_6) δ/ppm = 7.67 (d, J = 1.9 Hz, 1H), 7.59 (d, J = 1.8 Hz, 1H), 7.37 (d, J = 9.0 Hz, 1H), 7.25–7.19 (m, 3H), 7.17 (m_c 1H, partially covered by the solvent signal), 7.14–7.10 (m, 3H), 7.10–7.03 (m, 5H), 6.96 (m_c, 2H), 6.94–6.88 (m, 2H), 6.37 (d, J = 8.8 Hz, 1H), 5.55 (d, J = 5.5 Hz, 1H), 4.44 (d, J = 5.5 Hz, 1H), 3.02 (s, 2H).
^{13}C{^{1}H} NMR (126 MHz, C_6D_6) δ/ppm = 144.6, 144.5, 143.0, 142.8, 135.3, 135.1, 130.7, 130.6, 130.2, 130.1, 129.1, 128.9, 127.7, 127.6, 127.5, 126.7, 126.6, 126.0, 126.0, 125.9, 125.9, 122.5, 122.4, 118.6, 115.5, 111.5, 109.7, 62.6.
IR (ATR): $\tilde{\nu}$/cm^{-1} = 3376 (w), 1606 (vs), 1495 (vs), 1450 (m), 1348 (m), 1266 (m), 1150 (w), 1967 (m), 935 (m), 826 (vs), 741 (m), 698 (vs).
HRMS (ESI) calculated for $C_{33}H_{25}Br_2N_2^+$ [(M+H)]$^+$: 609.0359; found: 609.0361.

7-Methylnaphthalen-2-amine (S20)

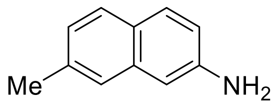

S20
$C_{11}H_{11}N$
M = 157.22 g/mol

Prepared from 7-methylnaphthalen-2-ol (3.16 g, 20.0 mmol, 1.00 equiv.), sodium bisulfite (4.20 g, 40.0 mmol, 2.00 equiv.) and aqueous ammonia (25 wt%, 100 mL) according to **GP 6**. Purification by recrystallization (*n*-heptane) afforded 7-methylnaphthalen-2-amine (**S20**, 2.30 g, 14.6 mmol, 73%) as an off-white solid.

melting point 98–102 °C.
^{1}H NMR (500 MHz, $CDCl_3$, 298 K) δ/ppm = 7.62 (t, J = 7.5 Hz, 2H), 7.38 (s, 1H), 7.09 (dd, J = 8.3, 1.7 Hz, 1H), 6.91 (d, J = 2.3 Hz, 1H), 6.88 (dd, J = 8.6, 2.4 Hz, 1H), 3.80 (s, 2H), 2.48 (s, 3H).

$^{13}C\{^1H\}$ NMR (126 MHz, $CDCl_3$) *δ*/ppm = 144.3, 136.1, 135.3, 129.0, 127.7, 126.4, 125.0, 124.9, 117.5, 108.3, 21.9.

The spectroscopic data are in accordance with those reported in the literature.[S18]

***rac*-7,7'-Dimethyl-[1,1'-binaphthalene]-2,2'-diamine (*rac*-S21)**

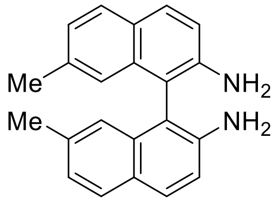

rac-**S21**
$C_{22}H_{20}N_2$
M = 312.42 g/mol

Prepared from 7-methylnaphthalen-2-amine (**S20**, 1.57 g, 10.0 mmol, 1.00 equiv.), copper(**II**) chloride dihydrate (3.41 g, 20.0 mmol, 2.00 equiv.) and benzylamine (8.57 g, 8.72 mL, 80.0 mmol, 8.00 equiv.) according to **GP 7**. Purification by flash column chromatography on silica gel (dichloromethane) afforded *rac*-7,7'-dimethyl-[1,1'-binaphthalene]-2,2'-diamine (*rac*-**S21**, 1.09 g, 3.49 mmol, 70%) as an orange solid.

R_f = 0.18.
^1H NMR (500 MHz, $CDCl_3$, 298 K) *δ*/ppm = 7.77 (d, *J* = 8.7 Hz, 2H), 7.72 (d, *J* = 8.3 Hz, 2H), 7.12–7.05 (m, 4H), 6.90 (s, 2H), 3.63 (s, 4H), 2.27 (s, 6H).
$^{13}C\{^1H\}$ NMR (126 MHz, $CDCl_3$) *δ*/ppm = 142.9, 136.6, 134.0, 129.2, 128.1, 126.9, 124.8, 123.1, 117.5, 112.5, 22.1.

The spectroscopic data are in accordance with those reported in the literature.[S19]

***rac*-*N*²-Benzhydryl-7,7'-dimethyl-[1,1'-binaphthalene]-2,2'-diamine (rac-4r)**

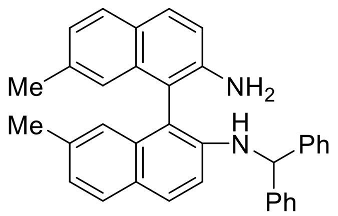

rac-**4r**
$C_{35}H_{30}N_2$
M = 478.64 g/mol

Prepared from *rac*-7,7'-dimethyl-[1,1'-binaphthalene]-2,2'-diamine (*rac*-**S21**, 625 mg, 2.00 mmol, 1.00 equiv.), potassium carbonate (346 mg, 2.50 mmol, 1.25 equiv.) and bromodiphenylmethane (544 mg, 2.20 mmol, 1.10 equiv.) according to **GP 6**.

Purification by flash column chromatography on silica gel (4 × 20, cyclohexane:dichloromethane = 1:1, 150 mL, 20 mL) afforded *rac*-N^2-benzhydryl-7,7'-dimethyl-[1,1'-binaphthalene]-2,2'-diamine (*rac*-**4r**, 488 mg, 1.02 mmol, 51%) as an off-white solid.

R$_f$ = 0.19.
^{1}H NMR (500 MHz, C_6D_6, 298 K) *δ*/ppm = 7.66 (d, *J* = 8.9 Hz, 1H), 7.63 (d, *J* = 8.3 Hz, 1H), 7.55 (d, *J* = 8.2 Hz, 1H), 7.52 (d, *J* = 8.8 Hz, 1H), 7.30 (s, 1H), 7.25 (s, 1H), 7.20 (d, *J* = 8.9 Hz, 1H), 7.17 (d, *J* = 5.2 Hz, 2H), 7.12 (m$_c$, 2H), 7.01–6.87 (m, 8H), 6.60 (d, *J* = 8.7 Hz, 1H), 5.67 (d, *J* = 5.9 Hz, 1H), 4.60 (d, *J* = 5.9 Hz, 1H), 3.16 (s, 2H), 1.99 (s, 3H), 1.94 (s, 3H).
^{13}C{^{1}H} NMR (126 MHz, C_6D_6, 298 K) *δ*/ppm = 144.0, 143.8, 143.7, 143.6, 136.8, 134.8, 134.6, 129.7, 129.6, 128.9, 128.8, 128.7, 128.7, 127.6, 127.5, 127.4, 127.3, 127.2, 126.9, 117.4, 114.5, 113.6, 111.8, 62.9, 21.9, 21.8.
IR (ATR): ṽ/cm^{-1} = 3370 /w), 3022 (w), 2916 /w), 1597 (vs), 1511 (vs), 1448 (s), 1336 (m), 1276 (m), 1149 (m), 825 (vs), 742 (s), 698 (vs).
HRMS (ESI) calculated for $C_{35}H_{31}N_2^+$ [(M+H)]$^+$: 479.2482; found: 479.2477.

7-Phenylnaphthalen-2-amine (S22)

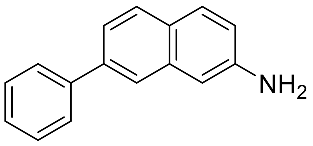

S22
$C_{16}H_{13}N$
M = 219.29 g/mol

Prepared from 7-phenylnaphthalen-2-ol (4.40 g, 20.0 mmol, 1.00 equiv.), sodium bisulfite (4.20 g, 40.0 mmol, 2.00 equiv.) and aqueous ammonia (25 wt%, 100 mL) according to **GP 6**. Purification by recrystallization (toluene) afforded 7-phenylnaphthalen-2-amine (**S22**, 3.82 g, 17.4 mmol, 87%) as a beige solid.

melting point 164–168 °C.
^{1}H NMR (400 MHz, $CDCl_3$, 298 K) *δ*/ppm = 7.81 (d, *J* = 1.8 Hz, 1H), 7.78 (d, *J* = 8.5 Hz, 1H), 7.75–7.68 (m, 3H), 7.54–7.47 (m, 3H), 7.42–7.36 (m, 1H), 7.04 (d, *J* = 2.3 Hz, 1H), 6.96 (dd, *J* = 8.6, 2.3 Hz, 1H), 3.86 (s, 2H).
^{13}C{^{1}H} NMR (101 MHz, $CDCl_3$, 298 K) *δ*/ppm = 144.6, 141.6, 139.2, 135.3, 129.1, 128.9, 128.3, 127.5, 127.3, 123.9, 122.4, 118.4, 109.0.

The spectroscopic data are in accordance with those reported in the literature.[S18]

***rac*-7,7'-Diphenyl-[1,1'-binaphthalene]-2,2'-diamine (*rac*-S23)**

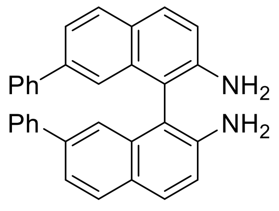

rac-**S23**
$C_{32}H_{24}N_2$
M = 436.56 g/mol

Prepared from 7-Phenylnaphthalen-2-amine (**S22**, 2.19 g, 10.0 mmol, 1.00 equiv.), copper(II) chloride dihydrate (3.41 g, 20.0 mmol, 2.00 equiv.) and benzylamine (8.57 g, 8.72 mL, 80.0 mmol, 8.00 equiv.) according to **GP 7**. Purification by flash column chromatography on silica gel (dichloromethane) afforded *rac*-7,7'-diphenyl-[1,1'-binaphthalene]-2,2'-diamine (*rac*-**S23**, ,777 mg, 1.78 mmol, 36%) as a yellow solid.

R_f = 0.33.
^{1}H NMR (500 MHz, $CDCl_3$, 298 K) δ/ppm = 7.89 (d, J = 8.4 Hz, 2H), 7.84 (d, J = 8.7 Hz, 2H), 7.53 (dd, J = 8.4, 1.8 Hz, 2H), 7.46–j7.42 (m, 4H), 7.37 (d, J = 1.9 Hz, 2H), 7.31 (t, J = 7.6 Hz, 4H), 7.27–7.22 (m, 2H), 7.16 (d, J = 8.7 Hz, 2H), 3.75 (s, 4H).
^{13}C{^{1}H} NMR (126 MHz, C_6D_6, 298 K) δ/ppm = 143.3, 141.8, 139.7, 134.0, 129.4, 128.9, 128.7, 127.9, 127.6, 127.1, 122.4, 122.1, 118.6, 112.8.
IR (ATR): ṽ/cm^{-1} = 3465 /w), 3370 (w), 1610 (vs), 1488 (s), 1376 (m), 1239 (w), 1151 (w), 884 (w), 833 (vs), 752 (vs), 694 (vs).
HRMS (ESI) calculated for $C_{32}H_{25}N_2^+$ [(M+H)]$^+$: 437.2012; found: 437.2007.

***rac*-*N*^{2}-Benzhydryl-7,7'-diphenyl-[1,1'-binaphthalene]-2,2'-diamine (*rac*-4s)**

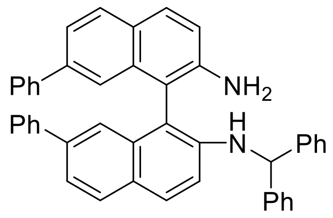

rac-**4s**
$C_{45}H_{34}N_2$
M = 602.78 g/mol

Prepared from *rac*-7,7'-diphenyl-[1,1'-binaphthalene]-2,2'-diamine (*rac*-**S23**, 616 mg, 1.40 mmol, 1.00 equiv.), potassium carbonate (242 mg, 1.75 mmol, 1.25 equiv.) and bromodiphenylmethane (380 mg, 1.54 mmol, 1.10 equiv.) according to **GP 5**. Purification by flash column chromatography on silica gel (cyclohexane:dichloromethane = 1:1) afforded *rac*-*N*^{2}-benzhydryl-7,7'-diphenyl-[1,1'-binaphthalene]-2,2'-diamine (*rac*-**4s**, 350 mg, 0.58 mmol, 41%) as an off-white solid.

R$_f$ = 0.16.

^{1}H NMR (500 MHz, C_6D_6, 298 K) *δ*/ppm = 7.94 (s, 1H), 7.85 (s, 1H), 7.72 (d, *J* = 8.4, 1H), 7.66–7.60 (m, 2H), 7.51–7.42 (m, 7H), 7.19 (d, *J* = 9.3 Hz, 1H), 7.11–7.06 (m, 4H), 7.03–6.93 (m, 8H), 6.92–6.88 (m, 1H), 6.83 (d, *J* = 7.4 Hz, 3H), 6.57 (dd, *J* = 8.8, 2.3 Hz, 1H), 5.60 (d, *J* = 5.7 Hz, 1H), 4.62 (d, *J* = 5.7 Hz, 1H), 3.16 (s, 2H).

^{13}C{^{1}H} NMR (126 MHz, C_6D_6, 298 K) *δ*/ppm = 144.3, 144.1, 143.5, 143.3, 142.2, 142.0, 140.4, 140.2, 129.8, 129.8, 129.6, 129.6, 129.0, 128.9, 128.9, 128.8, 127.7, 127.7, 127.5, 127.5, 127.4, 127.4, 127.2, 127.2, 122.6, 122.5, 122.3, 122.3, 118.5, 115.4, 113.7, 112.0, 62.7.

IR (ATR): ṽ/cm^{-1} = 3376 (w), 3022 (w), 1613 (s), 1489 (s), 1337 (m), 1245 (w), 1153 (w), 885 (w), 832 (s), 753 (s), 695 (vs).

HRMS (ESI) calculated for $C_{45}H_{35}N_2^+$ [(M+H)]$^+$: 603.2795; found: 603.2789.

***rac*-7,7'-Dimethoxy-[1,1'-binaphthalene]-2,2'-diamine (*rac*-S24)**

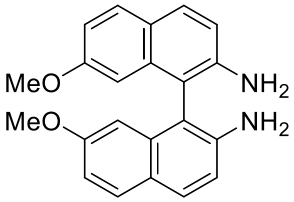

rac-**S24**
$C_{22}H_{20}N_2O_2$
M = 344.41 g/mol

Prepared from 7-methoxynaphthalen-2-amine (1.73 g, 10.0 mmol, 1.00 equiv.), copper(II) chloride dihydrate copper(II) chloride dihydrate (3.41 g, 20.0 mmol, 2.00 equiv.) and benzylamine (8.57 g, 8.72 mL, 80.0 mmol, 8.00 equiv.) according to **GP 7**. Purification by flash column chromatography on silica gel (dichloromethane:ethyl acetate = 20:1) afforded *rac*-7,7'-dimethoxy-[1,1'-binaphthalene]-2,2'-diamine (*rac*-**S24**, 1.35 g, 3.92 mmol, 78%) as a red solid.

R$_f$ = 0.33.

^{1}H NMR (400 MHz, $CDCl_3$, 298 K) *δ*/ppm = 7.70 (dd, *J* = 8.8, 7.1 Hz, 4H), 6.98 (d, *J* = 8.7 Hz, 2H), 6.91 (dd, *J* = 8.8, 2.5 Hz, 2H), 6.45 (d, *J* = 2.5 Hz, 2H), 3.69 (s, 4H), 3.55 (s, 6H). **^{13}C{^{1}H} NMR** (101 MHz, $CDCl_3$, 298 K) *δ*/ppm = 158.7, 143.3, 135.1, 129.8, 129.3, 124.0, 115.9, 114.2, 112.0, 103.3, 55.2.

The spectroscopic data are in accordance with those reported in the literature.[S24]

rac-N^2-Benzhydryl-7,7'-dimethoxy-[1,1'-binaphthalene]-2,2'-diamine (_rac_-4t)

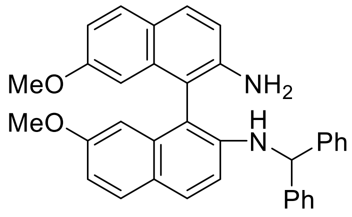

rac-**4t**
$C_{35}H_{30}N_2O_2$
M = 510.64 g/mol

Prepared from *rac*-7,7'-dimethoxy-[1,1'-binaphthalene]-2,2'-diamine (*rac*-**S24**, 689 mg, 2.00 mmol, 1.00 equiv.), potassium carbonate (346 mg, 2.50 mmol, 1.25 equiv.) and bromodiphenylmethane (544 mg, 2.20 mmol, 1.10 equiv.) according to **GP 5**. Purification by automatic column chromatography on silica gel chromatography (cyclohexane:ethyl acetate = 97:3 → 80:20) afforded *rac*-N^2-benzhydryl-7,7'-dimethoxy-[1,1'-binaphthalene]-2,2'-diamine (*rac*-**4t**, 499 mg, 0.98 mmol, 49%) as an off-white solid.

R_f = 0.40 (cyclohexane:dichloromethane = 3:1).
^{1}H NMR (500 MHz, C_6D_6, 298 K) *δ*/ppm 7.60 (d, *J* = 8.9 Hz, 1H), 7.56 (d, *J* = 8.9 Hz, 1H), 7.47 (dd, *J* = 8.8, 6.5 Hz, 2H), 7.19–7.17 (m, 2H, overlapped by solvent signal), 7.14–7.10 (m, 3H), 7.06 (dd, *J* = 8.8, 2.5 Hz, 1H), 7.03–6.87 (m, 7H), 6.82 (d, *J* = 2.6 Hz, 1H), 6.77 (d, *J* = 2.5 Hz, 1H), 6.54 (d, *J* = 8.7 Hz, 1H), 5.68 (d, *J* = 5.9 Hz, 1H), 4.65 (d, *J* = 6.1 Hz, 1H), 3.21 (s, 2H), 3.13 (s, 3H), 3.08 (s, 3H).
^{13}C{^{1}H} NMR (126 MHz, C_6D_6, 298 K) *δ*/ppm = 159.6, 159.6, 144.4, 144.1, 143.7, 143.6, 135.9, 135.6, 130.4, 130.3, 129.8, 128.9, 128.8, 127.6, 127.5, 127.4, 127.4, 124.2, 124.0, 115.6, 115.4, 113.2, 112.7, 111.5, 102.9, 102.7, 62.8, 54.5, 54.5.
IR (ATR): $\tilde{\nu}$/cm^{-1} = 3370 (w), 1614 (vs), 1510 (vs) 1452 (m), 1378 (w), 1338 (w), 1218 (vs), 1029 (m), 825 (s), 743 (m), 698 (s).
HRMS (ESI) calculated for $C_{35}H_{31}N_2O_2^+$ [(M+H)]$^+$: 511.2380; found: 511.2371.

4-Methylnaphthalen-2-amine (S25)

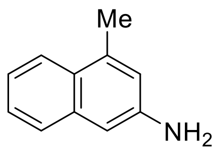

S25
$C_{11}H_{11}N$
M = 157.22 g/mol

Prepared from 4-methylnaphthalen-2-ol (3.16 g, 20.0 mmol, 1.00 equiv.), sodium bisulfite (4.20 g, 40.0 mmol, 2.00 equiv.) and aqueous ammonia (25 wt%, 100 mL) according to **GP 6**.

Purification by flash column chromatography on silica gel (dichloromethane:cyclohexane = 2:1) afforded 4-methylnaphthalen-2-amine (**S25**, 1.81 g, 11.5 mmol, 58%) as an orange solid.

melting point 68–72 °C.

$\mathbf{R_f}$ = 0.25.

^{1}H NMR (500 MHz, $CDCl_3$) *δ*/ppm = 7.86 (d, *J* = 8.4 Hz, 1H), 7.61 (d, *J* = 8.3 Hz, 1H), 7.40 (m_c, 1H), 7.29 (m_c, 1H), 6.87 (d, *J* = 2.3 Hz, 1H), 6.82 (d, *J* = 2.3 Hz, 1H), 3.77 (s, 2H), 2.64 (s, 3H).

^{13}C{^{1}H} NMR (126 MHz, $CDCl_3$) *δ*/ppm = 143.8, 135.4, 127.4, 126.5, 126.2, 124.1, 122.4, 119.2, 107.1, 19.4.

IR (ATR): ṽ/cm^{-1} = 3404 (w), 3311 (w), 3200 (w), 1620 (s), 1508 (m), 1467 (m), 1163 (m), 1026 (w), 1002 (w), 850 (vs), 738 (vs), 668 (s).

HRMS (ESI) calculated for $C_{11}H_{12}N^+$ $[(M+H)]^+$: 158.0964 found: 158.0964.

***rac*-4,4'-Dimethyl-[1,1'-binaphthalene]-2,2'-diamine (*rac*-S26)**

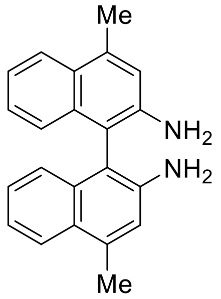

rac-**S26**

$C_{22}H_{20}N_2$

M = 312.42 g/mol

Prepared from 4-methylnaphthalen-2-amine (**S25**, 1.57 g, 10.0 mmol, 1.00 equiv.), copper(**II**) chloride dihydrate (3.41 g, 20.0 mmol, 2.00 equiv.) and benzylamine (8.57 g, 8.72 mL, 80.0 mmol, 8.00 equiv.) according to **GP 7**. Purification by flash column chromatography on silica gel (dichloromethane) afforded *rac*-4,4'-dimethyl-[1,1'-binaphthalene]-2,2'-diamine (*rac*-**S26**, 1.38 g, 4.41 mmol, 88%) as a yellow solid.

$\mathbf{R_f}$ = 0.28.

^{1}H NMR (500 MHz, $CDCl_3$, 298 K) *δ*/ppm = 7.96 (d, *J* = 8.3 Hz, 2H), 7.30– 7.26 (m, 2H), 7.21 (ddd, *J* = 8.2, 6.7, 1.4 Hz, 2H), 7.13 (d, *J* = 8.3 Hz, 2H), 7.01 (s, 2H), 3.63 (s, 4H), 2.86 (m, 6H).

^{13}C{^{1}H} NMR (126 MHz, $CDCl_3$, 298 K) *δ*/ppm = 142.5, 135.9, 134.2, 128.0, 126.6, 124.7, 124.4, 122.3, 119.3, 111.2, 19.6.

IR (ATR): ṽ/cm^{-1} = 3465 (w), 3422 (w), 3342 (w), 1610 (vs), 1381 (s), 1272 (w), 1219 (m), 871 (m), 754 (vs).

HRMS (ESI) calculated for $C_{22}H_{21}N_2^+$ $[(M+H)]^+$: 313.1699 found: 313.1700.

***rac*-*N*²-Benzhydryl-4,4'-dimethyl-[1,1'-binaphthalene]-2,2'-diamine (*rac*-4u)**

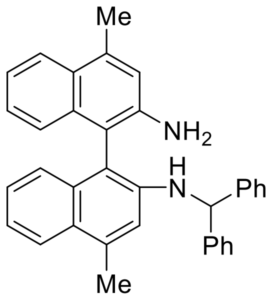

rac-**4u**
$C_{35}H_{30}N_2$
M = 478.64 g/mol

Prepared from *rac*-4,4'-dimethyl-[1,1'-binaphthalene]-2,2'-diamine (*rac*-**S26**, 625 mg, 2.00 mmol, 1.00 equiv.), potassium carbonate (346 mg, 2.50 mmol, 1.25 equiv.) and bromodiphenylmethane (544 mg, 2.20 mmol, 1.10 equiv.) according to **GP 5**. Purification by flash column chromatography on silica gel (cyclohexane:dichloromethane = 1:1) afforded *rac*-*N*²-benzhydryl-4,4'-dimethyl-[1,1'-binaphthalene]-2,2'-diamine (*rac*-**4u**, 451 mg, 0.94 mmol, 47%) as an off-white solid.

$\mathbf{R_f}$ = 0.26.
^{1}H NMR (500 MHz, C_6D_6, 298 K) δ/ppm = 7.86 (d, *J* = 8.3 Hz, 1H), 7.78 (dd, *J* = 7.9, 1.7 Hz, 1H), 7.44 (dd, *J* = 8.1, 1.3 Hz, 1H), 7.36 (d, *J* = 8.3 Hz, 1H), 7.22–7.07 (m, 9H), 7.03–6.98 (m, 2H), 6.96–6.89 (m, 3H), 6.88–6.83 (m, 1H), 6.45 (s, 1H), 5.72 (d, *J* = 6.0 Hz, 1H), 4.62 (d, *J* = 6.2 Hz, 1H), 3.13 (s, 2H), 2.48 (s, 3H), 2.38 (s, 3H).
^{13}C{^{1}H} NMR (126 MHz, C_6D_6, 298 K) *δ*/ppm = 143.8, 143.7, 143.5, 143.3, 135.9, 135.8, 135.0, 134.7, 128.8, 128.7, 127.8, 127.6, 127.4, 127.3, 126.9, 126.9, 125.4, 125.3, 124.8, 122.4, 119.3, 116.4, 112.4, 110.6, 62.8, 20.0, 19.5.
IR (ATR): $\tilde{\nu}$/cm^{-1} = 3369 (w), 1596 (vs), 1478 (m), 1385 (m), 1276 (w), 1222 (w), 1028 (w), 1007 (w), 847 (w), 749 (vs), 698 (vs).
HRMS (ESI) calculated for $C_{35}H_{31}N_2^+$ [(M+H)]$^+$: 479.2482 found: 479.2479.

***rac*-*N*²-Benzhydryl-5,5',6,6',7,7',8,8'-octahydro-[1,1'-binaphthalene]-2,2'-diamine (*rac*-4v)**

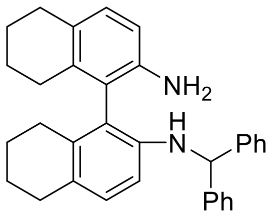

rac-**4v**
$C_{33}H_{34}N_2$
M = 458.65 g/mol

Prepared from *rac*-5,5',6,6',7,7',8,8'-octahydro-[1,1'-binaphthalene]-2,2'-diamine (497 mg, 1.70 mmol, 1.00 equiv.), potassium carbonate (317 mg, 2.30 mmol, 1.35 equiv.) and

bromodiphenylmethane (462 mg, 1.87 mmol, 1.10 equiv.) according to **GP 5**. Purification by flash column chromatography on silica gel (cyclohexane:dichloromethane = 1:1) afforded *rac*-N^2-benzhydryl-5,5',6,6',7,7',8,8'-octahydro-[1,1'-binaphthalene]-2,2'-diamine (*rac*-**4v**, 425 mg, 0.93 mmol, 55%) as an off-white solid.

R$_f$ = 0.17.
^{1}H NMR (500 MHz, C_6D_6, 298 K) δ/ppm = 7.31 (d, J = 7.5 Hz, 2H), 7.19 (m, 2H), 7.09 (td, J = 7.8, 2.4 Hz, 2H), 7.05–6.89 (m, 5H), 6.82 (dd, J = 8.2, 2.5 Hz, 1H), 6.67 (dd, J = 8.3, 2.6 Hz, 1H), 6.36 (dd, J = 8.1, 2.6 Hz, 1H), 5.54 (s, 1H), 4.23 (s, 1H), 2.94 (s, 2H), 2.68 (s, 2H), 2.64–2.45 (m, 4H), 2.40–2.31 (m, 2H), 1.65–1.54 (m, 8H).
^{13}C{^{1}H} NMR (126 MHz, C_6D_6, 298 K) δ/ppm = 129.9, 129.8, 128.8, 128.8, 127.7, 127.4, 127.3, 127.0, 122.7, 121.4, 113.4, 110.4, 63.2, 29.8, 29.7, 27.7, 27.6, 24.1, 24.0, 23.9, 23.8.
IR (ATR): $\tilde{v}$/cm^{-1} = 3371 (w), 2920 (w), 1590 (m), 1491 (m), 1258 (m), 1236 (m), 804 (s), 736 (s), 696 (vs).
HRMS (ESI) calculated for $C_{33}H_{35}N_2^+$ [M]$^+$: 459.2795; found: 459.2790.

2-Iodo-3,4-dimethyl-1-nitrobenzene (S27)

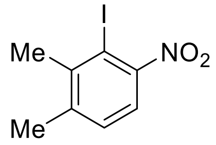

S27
$C_8H_8INO_2$
M = 277.06 g/mol

According to a modified literature procedure[S19] a flask was charged with 6-nitro-2,3-xylidine (4.99 g, 30.0 mmol, 1.00 equiv.) and acetonitrile (100 mL) was added. To this solution *tert*-butyl nitrite (3.40 g, 4.40 mL, 33.0 mmol, 1.10 equiv.) was added and the reaction mixture was cooled to 0°C. Subsequently a solution of methanesulfonic acid (3.17 g, 2.14 mL, 33.0 mmol, 1.10 equiv.) in acetonitrile (60 mL) was added dropwisely, keeping the temperature below 5°C. After complete addition, the reaction mixture was stirred at 0°C for 30 min. Then a solution of tetrabutylammonium iodide (13.3 g, 36.0 mmol, 1.20 equiv.) in acetonitrile (60 mL) was added dropwisely at 0°C. After complete addition, the reaction mixture was allowed to warm to ambient temperature and stirred for a further 3 h. Subsequently, the reaction was terminated by addition of saturated, aqueous sodium metabisulfite solution (100 mL) and saturated, aqueous sodium bicarbonate solution (100 mL). The reaction mixture was concentrated under reduced pressure, until most of the acetonitrile was evaporated. The remaining mixture was extracted with ethyl acetate (3 × 50 mL). The combined organic phases were washed with brine, dried over anhydrous Na_2SO_4, filtered and concentrated under reduced pressure. The residue was dissolved in dichloromethane, concentrated on silica gel and purified by flash column chromatography (cyclohexane:dichloromethane = 10:1) to afford 2-iodo-3,4-dimethyl-1-nitrobenzene (**S27**, 5.35 g, 19.3 mmol, 64%) as a yellow solid with 9% 3,4-dimethyl-1-nitrobenzene.

R$_f$ = 0.19.

^{1}H NMR (500 MHz, $(CD_3)_2CO$, 298 K) *δ*/ppm = 7.47 (d, *J* = 8.1 Hz, 1H), 7.39 (d, *J* = 8.2 Hz, 1H), 2.56 (s, 3H), 2.47 (s, 3H).

^{13}C{^{1}H} NMR (101 MHz, $(CD_3)_2CO$, 298 K) *δ*/ppm = 143.8, 142.2, 131.3, 122.1, 93.4, 26.5, 22.1.

IR (ATR): ṽ/cm^{-1} = 3068 (w), 2926 (w), 1517 (vs), 1344 (vs), 1271 (m), 1009 (m), 024 (s), 908 (vs), 736 (s).

HRMS (APCI) calculated for $C_8H_9INO_2^+$ [(M+H)]$^+$: 277.9672; found: 277.9671.

2,2',3,3'-Tetramethyl-6,6'-dinitro-1,1'-biphenyl (S28)

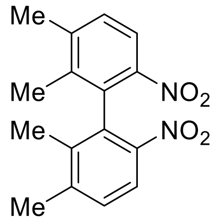

S28
$C_{16}H_{16}N_2O_4$
M = 300.31 g/mol

According to a modified literature procedure[S19] a flame-dried Schlenk tube was charged with 2-iodo-3,4-dimethyl-1-nitrobenzene (**S27**, 4.43 g, 16.0 mmol, 1.00 equiv.) and DMF (16 mL) was added. To the solution was added copper powder (6.41 g, 101 mmol, 6.30 equiv.). The reaction mixture was heated to 150°C and stirred at that temperature for 17 h. After cooling down to room temperature, the reaction mixture was filtered through celite using ethyl acetate (150 mL) as eluent. The filtrate was washed with water (3 × 50 mL) and brine (50 mL). The organic phase was washed with brine, dried over anhydrous Na_2SO_4, filtered and concentrated under reduced pressure. The residue was dissolved in dichloromethane, concentrated on silica gel and purified by flash column chromatography (cyclohexane:dichloromethane = 1:1) to afford 2,2',3,3'-tetramethyl-6,6'-dinitro-1,1'-biphenyl (**S28**, 2.08 g, 6.93 mmol, 87%) as a pale yellow solid.

R$_f$ = 0.30.

^{1}H NMR (500 MHz, Acetone-*d*$_6$, 298 K) *δ*/ppm = 7.92 (d, *J* = 8.4 Hz, 2H), 7.51 (d, *J* = 8.3 Hz, 2H), 2.45 (s, 6H), 1.89 (s, 6H).

^{13}C{^{1}H} NMR (101 MHz, Acetone-*d*$_6$, 298 K) *δ*/ppm = 147.8, 144.7, 138.0, 132.5, 130.9, 122.6, 20.9, 16.6.

IR (ATR): ṽ/cm^{-1} = 3103 (w), 2985 (w), 1578 (w), 1504 (vs), 1338 (vs), 1281 (s), 1137 (m), 807 (vs).

HRMS (APCI) calculated for $C_{16}H_{17}N_2O_4^+$ [(M+H)]$^+$: 301.1183; found: 301.1183.

rac-5,5',6,6'-Tetramethyl-[1,1'-biphenyl]-2,2'-diamine (rac-S29)

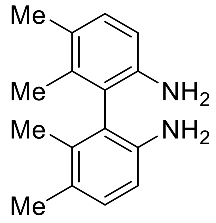

rac-**S29**
$C_{16}H_{20}N_2$
M = 240.35 g/mol

According to a modified literature procedure[S19] a Schlenk tube was charged with 2,2',3,3'-tetramethyl-6,6'-dinitro-1,1'-biphenyl (**S28**, 1.50 g, 5.00 mmol, 1.00 equiv.) and methanol (20 mL) was added. The suspension was degassed by briefly applying vacuum and quickly backfilling with nitrogen (3 ×). To the degassed suspension, Pd/C (299 mg, 0.28 mmol, 5.60 mol%, 10 wt.-%) was added. The suspension was then purged with hydrogen for 15 min and stirred under hydrogen atmosphere (balloon) for 14 h. After completion, the reaction mixture was filtered through Celite using methanol as eluent and the filtrate was concentrated under reduced pressure. The residue was dissolved in dichloromethane concentrated on silica gel, and purified by flash column chromatography (dichloromethane:ethyl acetate = 9:1) to afford *rac*-5,5',6,6'-tetramethyl-[1,1'-biphenyl]-2,2'-diamine (*rac*-**S29**, 1.07 g, 4.45 mmol, 89%) as an orange-brown solid.

R_f = 0.38.
^{1}H NMR (500 MHz, $CDCl_3$, 298 K) δ/ppm = 6.98 (d, J = 8.0 Hz, 2H), 6.63 (dd, J = 8.2, 2.5 Hz, 2H), 3.54 (d, J = 6.9 Hz, 4H), 2.23 (s, 6H), 1.87 (s, 6H).
^{13}C{^{1}H} NMR (126 MHz, C_6D_6, 298 K) δ/ppm = 142.3, 136.1, 129.7, 126.8, 123.5, 112.7, 20.0, 16.2.
IR (ATR): $\tilde{\nu}$/cm^{-1} = 3457 (w), 3432 (w), 3347 (w), 2921 (w), 1605 (s), 1475 (s), 1289 (s), 1015 (m), 813 (vs).
HRMS (ESI) calculated for $C_{16}H_{21}N_2^+$ [(M+H)]$^+$: 241.1699; found: 241.1699.

The spectroscopic data are in accordance with those reported in the literature.[S19]

rac-N^2-Benzhydryl-5,5',6,6'-tetramethyl-[1,1'-biphenyl]-2,2'-diamine (rac-4w)

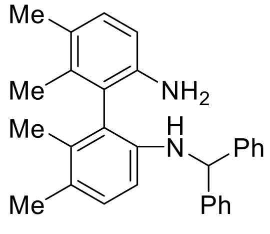

rac-**4w**
$C_{29}H_{30}N_2$
M = 406.57 g/mol

Prepared from *rac*-5,5',6,6'-tetramethyl-[1,1'-biphenyl]-2,2'-diamine (*rac*-**S29**, 480.7 mg, 2.00 mmol, 1.00 equiv.), potassium carbonate (346 mg, 2.50 mmol, 1.25 equiv.) and bromodiphenylmethane (544 mg, 2.20 mmol, 1.10 equiv.) according to **GP 6**. Purification by flash column chromatography on silica gel (cyclohexane:dichloromethane = 1:1) afforded *rac*-N^2-benzhydryl-5,5',6,6'-tetramethyl-[1,1'-biphenyl]-2,2'-diamine (*rac*-**4w**, 232 mg, 0.57 mmol, 29%) as a yellow wax.

R_f = 0.19.
^{1}H NMR (500 MHz, C_6D_6, 298 K) δ/ppm = 7.29 (d, J = 7.2 Hz, 2H), 7.21 (d, J = 7.2 Hz, 2H), 7.07 (t, J = 7.7 Hz, 2H), 7.03 (t, J = 7.7 Hz, 2H), 7.00–6.91 (m, 3H), 6.87 (d, J = 8.0 Hz, 1H), 6.62 (d, J = 8.2 Hz, 1H), 6.33 (d, J = 8.2 Hz, 1H), 5.53 (s, 1H), 4.21 (s, 1H), 2.95 (s, 2H), 2.15 (s, 3H), 2.03 (s, 3H), 1.98 (s, 6H).
^{13}C{^{1}H} NMR (126 MHz, C_6D_6, 298 K) δ/ppm = 144.1, 144.1, 143.7, 143.1, 136.1, 135.9, 130.2, 130.1, 128.8, 128.8, 127.6, 127.5, 127.3, 127.3, 126.3, 125.9, 124.1, 122.9, 112.8, 110.1, 63.3, 20.0, 19.9, 16.4.
IR (ATR): $\tilde{\nu}$/cm^{-1} = 2921 (w), 2853 (w), 1595 (s), 1492 (s), 1450 (s), 1301 (m), 801 (m), 740 (m), 697 (vs).
HRMS (ESI) calculated for $C_{29}H_{31}N_2^+$ [(M+H)]$^+$: 407.2482; found: 407.2479.

5 Kinetic Resolution of BINAM Derivatives

The silylamines are commonly isolated together with remaining dihydrosilane and are subject to decomposition towards air and moisture, therefore no clean NMR spectra can be obtained. Characteristic ^{1}H and $^{29}Si\{^{1}H\}$ NMR signals for both diastereomers and HRMS data are reported, if possible. The crude silylamine is deprotected and the yield of the resulting amine is given.

(*R*$_a$)-*N*^{2}-benzhydryl-[1,1'-binaphthalene]-2,2'-diamine [(*R*$_a$)-4f], (*S*$_a$)-*N*^{2}-benzhydryl-[1,1'-binaphthalene]-2,2'-diamine [(*S*$_a$)-4f], (*S*$_a$,^{Si}*S)-*N*^{2}-benzhydryl-*N*$^{2'}$-(*tert*-butyl(2-tolyl)silyl)-[1,1'-binaphthalene]-2,2'-diamine [(*S*$_a$,^{Si}*S**)-5fi]**

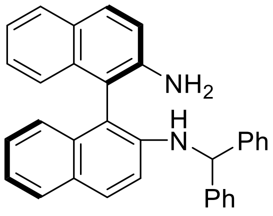

(*R*$_a$)-**4f**
$C_{33}H_{26}N_2$
M = 450.59 g/mol

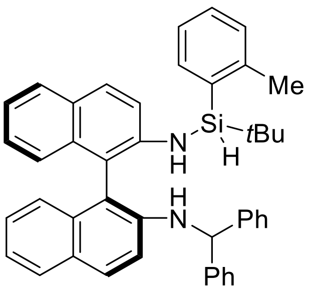

(*S*$_a$,^{Si}*S**)-**5fi**
$C_{44}H_{42}N_2Si$
M = 626.92 g/mol

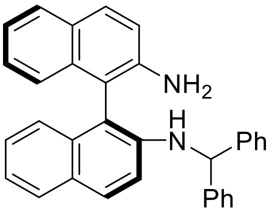

(*S*$_a$)-**5f**
$C_{33}H_{26}N_2$
M = 450.59 g/mol

Prepared according to **GP 8** from amine *rac*-**4f** (90.1 mg, 0.20 mmol, 1.00 equiv.) and *tert*-butyl(2-tolyl)silane (25.0 mg, 0.14 mmol, 0.70 equiv.). The reaction was stirred for 7 d. Purification by flash column chromatography on neutral alumina (cyclohexane:triethylamine = 20:1 → cyclohexane:dichloromethane = 3:1) afforded amine (*R*$_a$)-**4f** (50.6 mg. 0.11 mmol, 56%, 50% *ee*) and crude silylamine (*S*$_a$,^{Si}*S**)-**5fi** (46.3 mg, d.r. = 96:4) as off-white solids.

Silylamine (*S*$_a$,^{Si}*S**)-**5fi** (73.4 mg, d.r. = 96:4) was deprotected according to **GP 9** to afford amine (*S*$_a$)-**4f** (27.9 mg. 0.086 mmol, 31%, 87% *ee*) as an off-white solid.

Analytical data for amine (*R*$_a$)-**4f**:

The NMR spectroscopic data are in agreement with those reported in section 4 for the racemic amine.

Optical Rotation $[\alpha]_D^{RT}$ = +40.4 (c 0.5, $CHCl_3$, 50% *ee*). The enantiomeric excess of (*R*$_a$)-**XX** was determined by HPLC analysis on a chiral stationary phase (*Daicel Chiralpak*® IA column, column temperature 20°C, mobile phase *n*-heptane:isopropanol = 90:10, flow rate: 0.6 mL/min, λ = 254 nm): t_R =16.2 min (minor), t_R = 18.5 min (major).

Analytical data for silylamine (S_a,^{Si}S*)-**5fi**:

^{1}H NMR selected signals for the major diastereomer (500 MHz, C_6D_6, 298 K δ/ppm = 5.67 (d, J = 5.9 Hz, 1H), 5.30 (d, J = 2.9 Hz, 1H), 4.64 (d, J = 5.9 Hz, 1H), 4.16 (d, J = 2.8 Hz, 1H), 2.35 (s, 4H), 0.72 (s, 9H).

^{29}Si{^{1}H} DEPT NMR (99 MHz, C_6D_6, 298 K) δ/ppm = −12.2.

HRMS (APCI) calculated for $C_{44}H_{43}N_2Si^+$ [(M+H)]$^+$: 627.3190; found: 627.3187.

Analytical data for amine (S_a)-**4f**:

The NMR spectroscopic data are in agreement with those reported in section 4 for the racemic amine.

Optical Rotation $[\alpha]_D^{RT}$ = −66.9 (c 0.31, $CHCl_3$, 87% *ee*). The enantiomeric excess of (S_a)-**4f** was determined by HPLC analysis on a chiral stationary phase (*Daicel Chiralpak*® IA column, column temperature 20°C, mobile phase *n*-heptane:isopropanol = 90:10, flow rate: 0.6 mL/min, λ = 254 nm): t_R =16.2 min (major), t_R = 18.5 min (minor).

(*R*a)-*N*2-benzhydryl-6,6’-difluoro-[1,1'-binaphthalene]-2,2'-diamine [(*R*a)-4i], (*S*a)-*N*2-benzhydryl-6,6’-difluoro-[1,1'-binaphthalene]-2,2'-diamine [(*S*a)-4i] and (*S*a,SiS*)-*N*2-benzhydryl-6,6'-difluoro-*N*2'-(*tert*-butyl(phenyl)silyl)-[1,1'-binaphthalene]-2,2'-diamine [(*S*a,SiS*)-5ia]

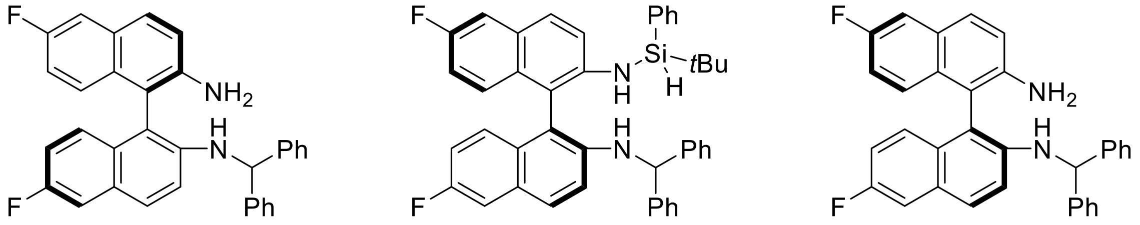

(R_a)-**4i**
$C_{33}H_{24}F_2N_2$
M = 486.57 g/mol

(S_a,^{Si}S*)-**5ia**
$C_{43}H_{38}F_2N_2Si$
M = 648.87 g/mol

(S_a)-**4i**
$C_{33}H_{24}F_2N_2$
M = 486.57 g/mol

Prepared according to **GP 8** from amine *rac*-**4i** (97.3 mg, 0.20 mmol, 1.00 equiv.) and *tert*-butyl(phenyl)silane (23.0 mg, 0.14 mmol, 0.70 equiv.). The reaction was stirred for 8 d. Purification by flash column chromatography on neutral alumina (cyclohexane:triethylamine = 20:1 → cyclohexane:dichloromethane = 3:1) afforded amine (R_a)-**4i** (46.9 mg. 0.097 mmol, 48%, 55% *ee*) and crude silylamine (S_a,^{Si}S*)-**5ia** (50.4 mg, d.r. = 90:10) as off-white solids.

Silylamine (S_a,^{Si}S*)-**5ia** (50.4 mg, d.r. = 90:10) was deprotected according to **GP 9** to afford amine (S_a)-**4i** (32.5 mg. 0.067 mmol, 33%, 77% *ee*) as an off-white solid.

Analytical data for amine (R_a)-**4i**:

The NMR spectroscopic data are in agreement with those reported in section 4 for the racemic amine.

Optical Rotation $[\alpha]_D^{RT}$ = +48.9 (c 0.5, $CHCl_3$, 55% *ee*). The enantiomeric excess of (*R*a)-**4i** was determined by HPLC analysis on a chiral stationary phase (*Daicel Chiralpak*® AD-H column, column temperature 20°C, mobile phase *n*-heptane:isopropanol = 90:10, flow rate: 0.6 mL/min, λ = 254 nm): t_R =14.7 min (minor), t_R = 19.6 min (major).

Analytical data for silylamine ((*S*a,^{Si}*S**)-**5ia**:

^{1}H NMR selected signals for the major diastereomer (500 MHz, C_6D_6, 298 K) δ/ppm = 7.40 (d, *J* = 9.0 Hz, 1H), 5.60 (d, *J* = 5.9 Hz, 1H), 5.10 (d, *J* = 2.9 Hz, 1H), 4.45 (d, *J* = 5.9 Hz, 1H), 3.99 (d, *J* = 2.9 Hz, 1H), 0.70 (s, 9H).

^{1}H NMR selected signals for the minor diastereomer (500 MHz, C_6D_6, 298 K) δ/ppm = 5.07 (d, *J* = 2.5 Hz, 1H), 4.52 (d, *J* = 5.8 Hz, 1H), 4.02 (s, 1H), 0.66 (s, 9H).

^{29}Si{^{1}H} DEPT NMR (99 MHz, C_6D_6, 298 K) δ/ppm = −9.8.

HRMS (APCI) calculated for $C_{43}H_{39}F_2N_2Si^+$ $[(M+H)]^+$: 649.2845; found: 649.2847.

Analytical data for amine (*S*a)-**4i**:

The NMR spectroscopic data are in agreement with those reported in section 4 for the racemic amine.

Optical Rotation $[\alpha]_D^{RT}$ = −59.4 (c 0.4, $CHCl_3$, 77% *ee*). The enantiomeric excess of (*S*a)-**4i** was determined by HPLC analysis on a chiral stationary phase (*Daicel Chiralpak*® AD-H column, column temperature 20°C, mobile phase *n*-heptane:isopropanol = 90:10, flow rate: 0.6 mL/min, λ = 254 nm): t_R =14.7 min (major), t_R = 19.6 min (minor).

(*R*a)-*N*^{2}-benzhydryl-6,6’-dichloro-[1,1'-binaphthalene]-2,2'-diamine [(*R*a)-4j], (*S*a)-*N*^{2}-benzhydryl-6,6’-dichloro-[1,1'-binaphthalene]-2,2'-diamine [(*S*a)-4j] and (*S*a,^{Si}*S)-*N*^{2}-benzhydryl-6,6'-dichloro-*N*$^{2'}$-(*tert*-butyl(phenyl)silyl)-[1,1'-binaphthalene]-2,2'-diamine [(*S*a,^{Si}*S**)-5ja]**

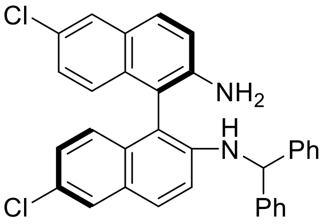

(*R*a)-**4j**
$C_{33}H_{24}Cl_2N_2$
M = 519.47 g/mol

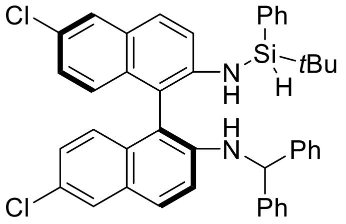

(*S*a,^{Si}*S**)-**5ja**
$C_{43}H_{38}Cl_2N_2Si$
M = 681.78 g/mol

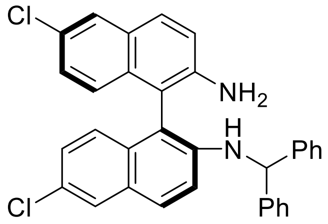

(*S*a)-**4j**
$C_{33}H_{24}Cl_2N_2$
M = 519.47 g/mol

Prepared according to **GP 8** from amine *rac*-**4j** (104 mg, 0.20 mmol, 1.00 equiv.) and *tert*-butyl(phenyl)silane (23.0 mg, 0.14 mmol, 0.70 equiv.). The reaction was stirred for 5 d. Purification by flash column chromatography on neutral alumina (cyclohexane:triethylamine = 20:1 → cyclohexane:dichloromethane = 3:1) afforded amine (*R*a)-**4j** (57.3 mg. 0.11 mmol, 55%, 57% *ee*) and crude silylamine (*S*a,^{Si}*S**)-**5ja** (53.9 mg, d.r. = 94:6) as off-white solids.

Silylamine (S_a,^{Si}S*)-**5ja** (53.9 mg, dr = 94:6) was deprotected according to **GP 9** to afford amine (S_a)-**XX** (33.9 mg. 0.065 mmol, 33%, 84% *ee*) as an off-white solid.

Analytical data for amine (R_a)-**4j**:
The NMR spectroscopic data are in agreement with those reported in section 4 for the racemic amine.
Optical Rotation $[\alpha]_D^{RT}$ = +35.2 (c 0.5, $CHCl_3$, 57% *ee*). The enantiomeric excess of (R_a)-**4j** was determined by HPLC analysis on a chiral stationary phase (*Daicel Chiralpak*® AD-H column, column temperature 20°C, mobile phase *n*-heptane:isopropanol = 95:5, flow rate: 0.6 mL/min, λ = 254 nm): t_R =23.3 min (minor), t_R = 26.7 min (major).

Analytical data for silylamine ((S_a,^{Si}S*)-**5ja**:
^{1}H NMR selected signals for the major diastereomer (500 MHz, C_6D_6, 298 K) *δ*/ppm = 7.62 (d, *J* = 2.1 Hz, 1H), 7.42 (d, *J* = 1.9 Hz, 1H), 7.35 (d, *J* = 9.0 Hz, 1H), 5.59 (d, *J* = 6.0 Hz, 1H), 5.08 (d, *J* = 2.9 Hz, 1H), 4.49 (d, *J* = 6.0 Hz, 1H), 4.04 (d, *J* = 3.0 Hz, 1H), 0.69 (s, 9H).
^{1}H NMR selected signals for the minor diastereomer (500 MHz, C_6D_6, 298 K) *δ*/ppm = 4.56 (d, *J* = 5.8 Hz, 1H), 0.65 (s, 9H).
^{1}H/^{29}Si HMQC NMR (500/99 MHz, C_6D_6, 298 K, optimized for *J* = 200 Hz) *δ*/ppm = 5.08/−9.8.
HRMS (APCI) calculated for $C_{43}H_{39}Cl_2N_2Si^+$ $[(M+H)]^+$: 681.2254; found: 681.2251.

Analytical data for amine (S_a)-**4j**:
The NMR spectroscopic data are in agreement with those reported in section 4 for the racemic amine.
Optical Rotation $[\alpha]_D^{RT}$ = −50.5 (c 0.5, $CHCl_3$, 84% *ee*). The enantiomeric excess of (S_a)-**4j** was determined by HPLC analysis on a chiral stationary phase (*Daicel Chiralpak*® AD-H column, column temperature 20°C, mobile phase *n*-heptane:isopropanol = 90:10, flow rate: 0.6 mL/min, λ = 254 nm): t_R =23.4 min (major), t_R = 26.8 min (minor).

(*R*a)-*N*²-benzhydryl-6,6'-dibromo-[1,1'-binaphthalene]-2,2'-diamine [(*R*a)-4k], (*S*a)-*N*²-benzhydryl-6,6'-dibromo-[1,1'-binaphthalene]-2,2'-diamine [(*S*a)-4k] and (*S*a,^{Si}*S)-*N*²-benzhydryl-6,6'-dibromo-*N*²'-(*tert*-butyl(phenyl)silyl)-[1,1'-binaphthalene]-2,2'-diamine [(*S*a,^{Si}*S**)-5ka]**

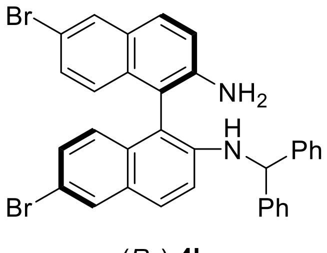

(R_a)-**4k**
$C_{33}H_{24}Br_2N_2$
M = 608.38 g/mol

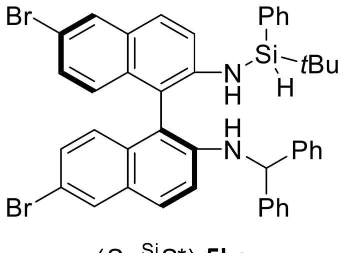

(S_a,^{Si}S*)-**5ka**
$C_{43}H_{38}Br_2N_2Si$
M = 770.68 g/mol

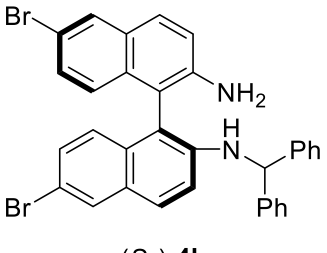

(S_a)-**4k**
$C_{33}H_{24}Br_2N_2$
M = 608.38 g/mol

Prepared according to **GP 8** from amine *rac*-**4k** (122 mg, 0.20 mmol, 1.00 equiv.) and *tert*-butyl(phenyl)silane (23.0 mg, 0.14 mmol, 0.70 equiv.). The reaction was stirred for 5 d. Purification by flash column chromatography on neutral alumina (cyclohexane:triethylamine = 20:1 → cyclohexane:dichloromethane = 3:1) afforded amine (R_a)-**4k** (47.8 mg. 0.079 mmol, 39%, 85% *ee*) and crude silylamine (S_a,^{Si}S*)-**5ka** (73.4 mg, d.r. = 92:8) as off-white solids.

Silylamine (S_a,^{Si}S*)-**5ka** (73.4 mg, d.r. = 92:8) was deprotected according to **GP 9** to afford amine (S_a)-**4k** (52.6 mg. 0.086 mmol, 43%, 77% *ee*) as an off-white solid.

Analytical data for amine (R_a)-**4k**:
The NMR spectroscopic data are in agreement with those reported in section 4 for the racemic amine.
Optical Rotation $[\alpha]_D^{RT}$ = +36.8 (c 0.5, $CHCl_3$, 85% *ee*). The enantiomeric excess of (R_a)-**4k** was determined by HPLC analysis on a chiral stationary phase (*Daicel Chiralpak*® AD-H column, column temperature 20°C, mobile phase *n*-heptane:isopropanol = 90:10, flow rate: 0.6 mL/min, λ = 254 nm): t_R =16.7 min (minor) , t_R = 19.4 min (major).

Analytical data for silylamine (S_a,^{Si}S*)-**5ka**:
^{1}H NMR selected signals for the major diastereomer (700 MHz, C_6D_6, 298 K) δ/ppm = 7.79 (d, J = 2.1 Hz, 1H), 7.58 (d, J = 2.1 Hz, 1H), 7.51–7.47 (m, 2H), 7.32 (d, J = 9.1 Hz, 1H), 7.27 (dd, J = 9.0, 2.1 Hz, 1H), 7.22 (dd, J = 9.0, 2.0 Hz, 1H), 7.15 (m_c, 5H, partly overlapped by the solvent signal), 7.13 (d, J = 8.9 Hz, 1H), 7.10 (d, J = 9.1 Hz, 1H), 7.05 (m_c, 2H), 7.02–6.99 (m, 3H), 6.97–6.94 (m, 3H), 6.94– 6.91 (m, 2H), 6.88 (m_c, 1H) 5.58 (d, J = 5.9 Hz, 1H), 5.07 (d, J = 2.9 Hz, 1H), 4.50 (d, J = 5.9 Hz, 1H), 4.05 (d, J = 3.0 Hz, 1H), 0.69 (s, 9H).
^{1}H NMR selected signals for the minor diastereomer (700 MHz, C_6D_6, 298 K) δ/ppm = 5.60 (d, J = 5.9 Hz, 1H), 5.04 (d, J = 2.6 Hz, 1H), 4.56 (d, J = 5.9 Hz, 1H), 4.06 (d, J = 2.6 Hz, 1H), 0.64 (s, 9H).
^{29}Si{^{1}H} DEPT NMR (99 MHz, C_6D_6, 298 K) δ/ppm = −9.8.
HRMS (APCI) calculated for $C_{43}H_{39}Br_2N_2Si^+$ [(M+H)]$^+$: 769.1244; found: 769.1248.

Analytical data for amine (S_a)-**4k**:
The NMR spectroscopic data are in agreement with those reported in section 4 for the racemic amine.
Optical Rotation $[\alpha]_D^{RT}$ = −34.4 (c 0.5, $CHCl_3$, 76% *ee*). The enantiomeric excess of (S_a)-**4k** was determined by HPLC analysis on a chiral stationary phase (*Daicel Chiralpak*® AD-H column, column temperature 20°C, mobile phase *n*-heptane:isopropanol = 90:10, flow rate: 0.6 mL/min, λ = 254 nm): t_R =16.7 min (major), t_R = 19.4 min (minor).

(R_a)-N^2-benzhydryl-6,6'-dimethyl-[1,1'-binaphthalene]-2,2'-diamine [(R_a)-4l], (S_a)-N^2-benzhydryl-6,6'-dimethyl-[1,1'-binaphthalene]-2,2'-diamine [(S_a)-4l] and (S_a,$^{Si}S^*$)-N^2-benzhydryl-6,6'-dimethyl-$N^{2'}$-(*tert*-butyl(phenyl)silyl)-[1,1'-binaphthalene]-2,2'-diamine [(S_a,$^{Si}S^*$)-5la]

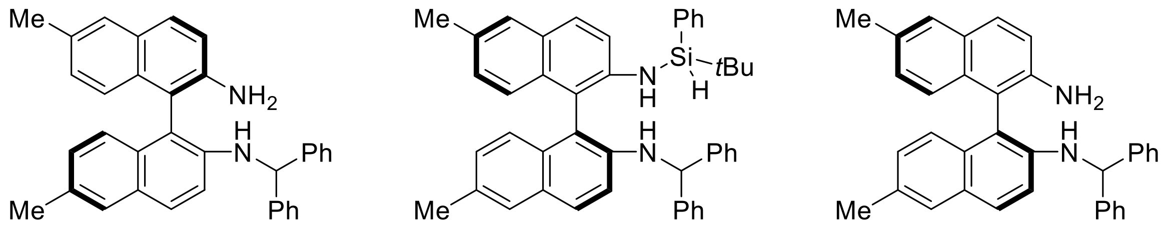

(R_a)-**4l** $C_{35}H_{30}N_2$ M = 478.64 g/mol

(S_a,$^{Si}S^*$)-**5la** $C_{45}H_{44}N_2Si$ M = 640.95 g/mol

(S_a)-**4l** $C_{35}H_{30}N_2$ M = 478.64 g/mol

Prepared according to **GP 8** from amine *rac*-**4l** (95.7 mg, 0.20 mmol, 1.00 equiv.) and *tert*-butyl(phenyl)silane (23.0 mg, 0.14 mmol, 0.70 equiv.). The reaction was stirred for 4 d. Purification by flash column chromatography on neutral alumina (cyclohexane:triethylamine = 20:1 → cyclohexane:dichloromethane = 3:1) afforded amine (R_a)-**4l** (56.9 mg. 0.12 mmol, 59%, 47% *ee*) and crude silylamine (S_a,$^{Si}S^*$)-**5la** (49.4 mg, d.r. = 93:7) as off-white solids.

Silylamine (S_a,$^{Si}S^*$)-**5la** (49.4 mg, d.r. = 93:7) was deprotected according to **GP 9** to afford amine (S_a)-**4l** (28.9 mg. 0.06 mmol, 30%, 83% *ee*) as an off-white solid.

Analytical data for amine (R_a)-**4l**:
The NMR spectroscopic data are in agreement with those reported in section 4 for the racemic amine.
Optical Rotation $[\alpha]_D^{RT}$ = +15.6 (c 0.5, $CHCl_3$, 47% *ee*). The enantiomeric excess of (R_a)-**4l** was determined by HPLC analysis on a chiral stationary phase (*Daicel Chiralpak*® IA column, column temperature 20°C, mobile phase *n*-heptane:isopropanol = 97:3, flow rate: 0.6 mL/min, λ = 254 nm): t_R =21.2 min (major), t_R = 23.8 min (minor).

Analytical data for silylamine (S_a,$^{Si}S^*$)-**5la**:
^{1}H NMR selected signals for the major diastereomer (500 MHz, C_6D_6, 298 K) δ/ppm = 5.70 (d, J = 5.8 Hz, 1H), 5.17 (d, J = 2.9 Hz, 1H), 4.64 (d, J = 5.8 Hz, 1H), 4.18 (d, J = 2.9 Hz, 1H), 2.21 (s, 3H), 2.18 (s, 3H), 0.73 (s, 9H).
^{1}H NMR selected signals for the minor diastereomer (500 MHz, C_6D_6, 298 K) δ/ppm = 4.71 (d, J = 5.6 Hz, 1H), 4.21 (d, J = 2.4 Hz, 1H), 0.70 (s, 9H).
^{1}H/^{29}Si HMQC NMR (500/99 MHz, C_6D_6, 298 K, optimized for J = 200 Hz) δ/ppm = 5.17/−10.0.
HRMS (APCI) calculated for $C_{45}H_{45}N_2Si^+$ $[(M+H)]^+$: 641.3347; found: 641.3353.

Analytical data for amine (S_a)-**4l**:

The NMR spectroscopic data are in agreement with those reported in section 4 for the racemic amine.

Optical Rotation $[\alpha]_D^{RT}$ = −29.5 (c 0.5, $CHCl_3$, 83% *ee*). The enantiomeric excess of (S_a)-**4l** was determined by HPLC analysis on a chiral stationary phase (*Daicel Chiralpak*® IA column, column temperature 20°C, mobile phase *n*-heptane:isopropanol = 97:3, flow rate: 0.6 mL/min, λ = 254 nm): t_R =21.3 min (minor), t_R = 23.8 min (major).

(*R*a)-*N*2-benzhydryl-6,6'-dimethyl-[1,1'-binaphthalene]-2,2'-diamine [(*R*a)-4l], (*S*a)-*N*2-benzhydryl-6,6'-dimethyl-[1,1'-binaphthalene]-2,2'-diamine [(*S*a)-4l] and (*S*a,Si*S)-*N*2-benzhydryl-6,6'-dimethyl-*N*2'-(*tert*-butyl(2-tolyl)silyl)-[1,1'-binaphthalene]-2,2'-diamine [(*S*a,Si*S**)-5li]**

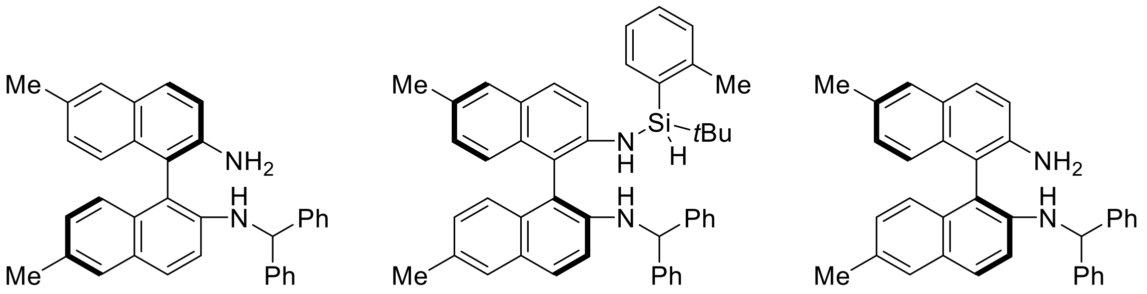

(R_a)-**4l** $C_{35}H_{30}N_2$ M = 478.64 g/mol

(S_a,^{Si}S*)-**5li** $C_{46}H_{46}N_2Si$ M = 654.97 g/mol

(S_a)-**4l** $C_{35}H_{30}N_2$ M = 478.64 g/mol

Prepared according to **GP 8** from amine *rac*-**4l** (95.7 mg, 0.20 mmol, 1.00 equiv.) and *tert*-butyl(2-tolyl)silane (25.0 mg, 0.14 mmol, 0.70 equiv.). The reaction was stirred for 8 d. Purification by flash column chromatography on neutral alumina (cyclohexane:triethylamine = 20:1 → cyclohexane:dichloromethane = 3:1) afforded amine (R_a)-**4l** (51.4 mg. 0.11 mmol, 54%, 57% *ee*) and crude silylamine (S_a,^{Si}S*)-**5li** (47.9 mg, d.r. = 97:3) as off-white solids.

Silylamine (S_a,^{Si}S*)-**5li** (47.9 mg, d.r. = 97:3) was deprotected according to **GP 9** to afford amine (S_a)-**4l** (30.1 mg. 0.06 mmol, 31%, 88% *ee*) as an off-white solid.

Analytical data for amine (R_a)-**4l**:

The NMR spectroscopic data are in agreement with those reported in section 4 for the racemic amine.

Optical Rotation $[\alpha]_D^{RT}$ = +22.2 (c 0.55, $CHCl_3$, 57% *ee*). The enantiomeric excess of (R_a)-**4l** was determined by HPLC analysis on a chiral stationary phase (*Daicel Chiralpak*® IA column, column temperature 20°C, mobile phase *n*-heptane:isopropanol = 97:3, flow rate: 0.6 mL/min, λ = 254 nm): t_R =21.4 min (major), t_R = 24.0 min (minor).

Analytical data for silylamine (S_a,^{Si}S*)-**5li**:

^{1}H NMR selected signals for the major diastereomer (500 MHz, C_6D_6, 298 K) δ/ppm = ^{1}H NMR (500 MHz, Benzene-d_6) δ 5.71 (d, J = 5.7 Hz, 1H), 5.33 (d, J = 2.7 Hz, 1H), 4.64 (d, J = 5.7 Hz, 1H), 4.18 (d, J = 2.8 Hz, 1H), 2.38 (s, 3H), 2.21 (s, 3H), 2.18 (s, 3H), 0.76 (s, 9H).

^{1}H NMR selected signals for the minor diastereomer (500 MHz, C_6D_6, 298 K) δ/ppm = 0.71 (s, 9H).

^{1}H/^{29}Si HMQC NMR (500/99 MHz, C_6D_6, 298 K, optimized for J = 200 Hz) δ/ppm = 5.33/−12.1.

HRMS (APCI) calculated for $C_{46}H_{47}N_2Si^+$ [(M+H)]$^+$: 655.3503; found: 655.3511.

Analytical data for amine (S_a)-**4l**:

The NMR spectroscopic data are in agreement with those reported in section 4 for the racemic amine.

Optical Rotation $[\alpha]_D^{RT}$ = −29.7 (c 0.42, $CHCl_3$, 88% *ee*). The enantiomeric excess of (S_a)-**4l** was determined by HPLC analysis on a chiral stationary phase (*Daicel Chiralpak*® IA column, column temperature 20°C, mobile phase *n*-heptane:isopropanol = 97:3, flow rate: 0.6 mL/min, λ = 254 nm): t_R =21.5 min (minor), t_R = 24.0 min (major).

(*R*a)-*N*²-benzhydryl-6,6'-diisopropyl-[1,1'-binaphthalene]-2,2'-diamine [(*R*a)-4m], (*S*a)-*N*²-benzhydryl-6,6'-diisopropyl-[1,1'-binaphthalene]-2,2'-diamine (*S*a)-4m and (*S*a,^{Si}*S)-*N*²-benzhydryl-6,6'-diisopropyl-*N*²'-(*tert*-butyl(phenyl)silyl)-[1,1'-binaphthalene]-2,2'-diamine (*S*a,^{Si}*S**)-5ma**

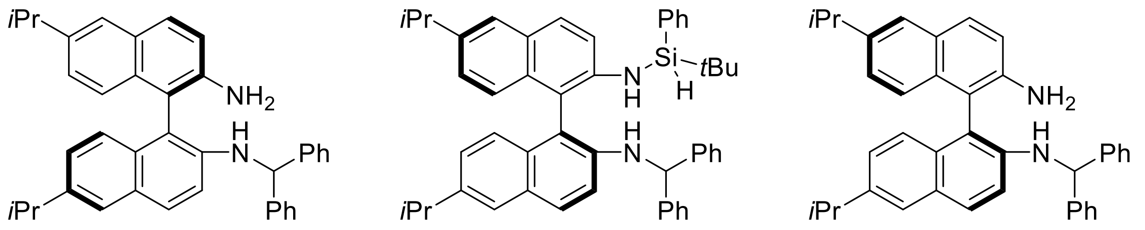

(R_a)-**4m** $C_{39}H_{38}N_2$ M = 534.75 g/mol

(S_a,^{Si}S*)-**5ma** $C_{49}H_{52}N_2Si$ M = 697.05 g/mol

(S_a)-**4m** $C_{39}H_{38}N_2$ M = 534.75 g/mol

Prepared according to **GP 8** from amine *rac*-**4m** (106.7 mg, 0.20 mmol, 1.00 equiv.) and *tert*-butyl(phenyl)silane (23.0 mg, 0.14 mmol, 0.70 equiv.). The reaction was stirred for 7 d. Purification by flash column chromatography on neutral alumina (cyclohexane:triethylamine = 20:1 → cyclohexane:dichloromethane = 3:1) afforded amine (R_a)-**4m** (62.5 mg. 0.12 mmol, 58%, 32% *ee*) and crude silylamine (S_a,^{Si}S*)-**5ma** (41.6 mg, d.r. = 89:11) as off-white solids.

Silylamine (S_a,^{Si}S*)-**5ma** (49.4 mg, d.r. = 89:11) was deprotected according to **GP 9** to afford amine (S_a)-**4m** (24.3 mg. 0.05 mmol, 23%, 76% *ee*) as an off-white solid.

Analytical data for amine (R_a)-**4m**:

The NMR spectroscopic data are in agreement with those reported in section 4 for the racemic amine.

Optical Rotation $[\alpha]_D^{RT}$ = +25.0 (c 0.54, $CHCl_3$, 32% *ee*). The enantiomeric excess of (R_a)-**4m** was determined by HPLC analysis on a chiral stationary phase (*Daicel Chiralpak*® IA column, column temperature 20°C, mobile phase *n*-heptane:isopropanol = 98:2, flow rate: 0.6 mL/min, λ = 254 nm): t_R =14.1 min (major), t_R = 20.6 min (minor).

Analytical data for silylamine (S_a,^{Si}S*)-**5ma**:

^{1}H NMR selected signals for the major diastereomer (500 MHz, C_6D_6, 298 K) 5.70 (d, *J* = 6.1 Hz, 1H), 5.16 (d, *J* = 2.6 Hz, 1H), 4.65 (d, *J* = 6.0 Hz, 1H), 4.17 (s, 1H), 2.80 (m, 2H), 1.22–1.13 (m, 6H), 0.71 (s, 9H).

^{1}H NMR selected signals for the minor diastereomer (500 MHz, C_6D_6, 298 K) *δ*/ppm = 4.70 (d, *J* = 6.4 Hz, 1H), 0.68 (s, 9H).

^{1}H/^{29}Si HMQC NMR (500/99 MHz, C_6D_6, 298 K, optimized for *J* = 200 Hz) *δ*/ppm = 5.16/−9.8.

HRMS (APCI) calculated for $C_{49}H_{53}N_2Si^+$ $[(M+H)]^+$: 697.3973; found: 697.3975.

Analytical data for amine (S_a)-**4m**:

The NMR spectroscopic data are in agreement with those reported in section 4 for the racemic amine.

Optical Rotation $[\alpha]_D^{RT}$ = −60.7 (c 0.28, $CHCl_3$, 76% *ee*). The enantiomeric excess of (S_a)-**4m** was determined by HPLC analysis on a chiral stationary phase (*Daicel Chiralpak*® IA column, column temperature 20°C, mobile phase *n*-heptane:isopropanol = 98:2, flow rate: 0.6 mL/min, λ = 254 nm): t_R =14.0 min (minor), t_R = 20.6 min (major).

(R_a)-*N*^{2}-benzhydryl-6,6'-diphenyl-[1,1'-binaphthalene]-2,2'-diamine [(R_a)-4n], (S_a)-*N*^{2}-benzhydryl-6,6'-diphenyl-[1,1'-binaphthalene]-2,2'-diamine [(S_a)-4n] and (S_a,^{Si}S*)-*N*^{2}-benzhydryl-6,6'-diphenyl-*N*$^{2'}$-(*tert*-butyl(phenyl)silyl)-[1,1'-binaphthalene]-2,2'-diamine [(S_a,^{Si}S*)-5na]

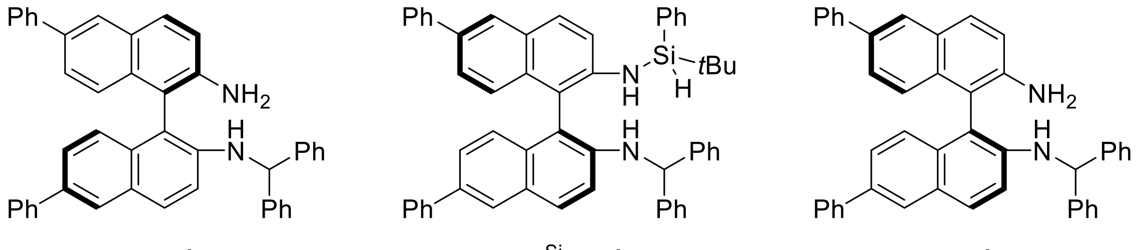

(R_a)-**4n** $C_{45}H_{34}N_2$ M = 602.78 g/mol | (S_a,^{Si}S*)-**4n** $C_{55}H_{48}N_2Si$ M = 765.09 g/mol | (S_a)-**4na** $C_{45}H_{34}N_2$ M = 602.78 g/mol

Prepared according to **GP 8** from amine *rac*-**4n** (120.6 mg, 0.20 mmol, 1.00 equiv.) and *tert*-butyl(phenyl)silane (23.0 mg, 0.14 mmol, 0.70 equiv.). The reaction was stirred for 9 d. Purification by flash column chromatography on neutral alumina (cyclohexane:triethylamine = 20:1 → cyclohexane:dichloromethane = 3:1) afforded amine (R_a)-**4n** (75.2 mg. 0.12 mmol, 62%, 30% *ee*) and crude silylamine (S_a,^{Si}S*)-**5na** (38.4 mg, d.r. = 95:5) as off-white solids.

Silylamine (S_a,^{Si}S*)-**5na** (38.4 mg, d.r. = 95:5) was deprotected according to **GP 9** to afford amine (S_a)-**4n** (21.5 mg. 0.036 mmol, 18%, 89% *ee*) as an off-white solid.

Analytical data for amine (R_a)-**4n**:
The NMR spectroscopic data are in agreement with those reported in section 4 for the racemic amine.
Optical Rotation $[\alpha]_D^{RT}$ = −6.2 (c 0.5, $CHCl_3$, 30% *ee*). The enantiomeric excess of (R_a)-**4n** was determined by HPLC analysis on a chiral stationary phase (*Daicel Chiralpak*® AD-H column, column temperature 20°C, mobile phase *n*-heptane:isopropanol = 90:10, flow rate: 0.6 mL/min, λ = 254 nm): t_R =29.8 min (minor), t_R = 49.9 min (minor).

Analytical data for silylamine (S_a,^{Si}S*)-**5na**:
^{1}H NMR selected signals for the major diastereomer (500 MHz, C_6D_6, 298 K) *δ*/ppm = 5.74 (d, *J* = 6.0 Hz, 1H), 5.19 (d, *J* = 2.9 Hz, 1H), 4.75 (d, *J* = 6.0 Hz, 1H), 4.30 (d, *J* = 3.0 Hz, 1H), 0.75 (s, 9H).
^{1}H/^{29}Si HMQC NMR (500/99 MHz, C_6D_6, 298 K, optimized for *J* = 200 Hz) *δ*/ppm = 5.17/−9.7.
HRMS (APCI) calculated for $C_{55}H_{49}N_2Si^+$ [(M+H)]$^+$: 765.3660; found: 765.3668.

Analytical data for amine (S_a)-**4n**:
The NMR spectroscopic data are in agreement with those reported in section 4 for the racemic amine.

Optical Rotation $[\alpha]_D^{RT}$ = +22.6 (c 0.33, $CHCl_3$, 89% *ee*). The enantiomeric excess of (S_a)-**4n** was determined by HPLC analysis on a chiral stationary phase (*Daicel Chiralpak*® AD-H column, column temperature 20°C, mobile phase *n*-heptane:isopropanol = 90:10, flow rate: 0.6 mL/min, λ = 254 nm): t_R =29.8 min (major), t_R = 49.7 min (major).

(*R*a)-*N*²-benzhydryl-6,6’-bis(trimethylsilyl)-[1,1'-binaphthalene]-2,2'-diamine [(*R*a)-4o], (*S*a)-*N*²-benzhydryl-6,6’- bis(trimethylsilyl)-[1,1'-binaphthalene]-2,2'-diamine [(*S*a)-4o] and (*S*a,SiS*)-*N*²-benzhydryl-6,6'- bis(trimethylsilyl)-*N*²'-(*tert*-butyl(phenyl)silyl)-[1,1'-binaphthalene]-2,2'-diamine [(*S*a,SiS*)-5oa]

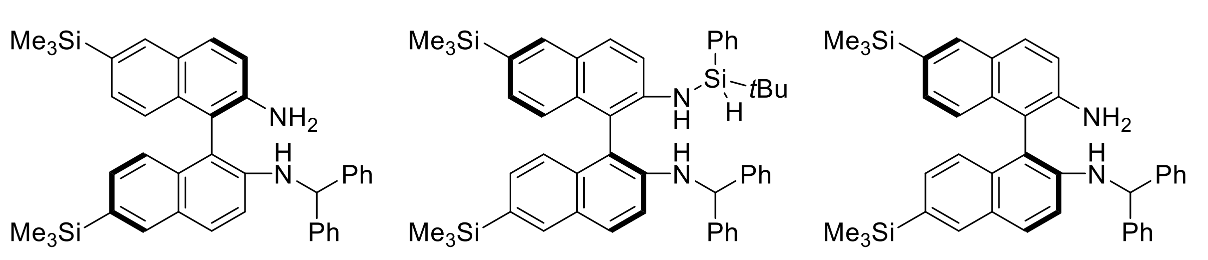

(R_a)-**4o** $C_{39}H_{42}N_2Si_2$ M = 594.95 g/mol

(S_a,^{Si}S*)-**5oa** $C_{49}H_{56}N_2Si_3$ M = 757.26 g/mol

(S_a)-**4o** $C_{39}H_{42}N_2Si_2$ M = 594.95 g/mol

Prepared according to **GP 8** from amine *rac*-**4o** (119.0 mg, 0.20 mmol, 1.00 equiv.) and *tert*-butyl(phenyl)silane (23.0 mg, 0.14 mmol, 0.70 equiv.). The reaction was stirred for 8 d. Purification by flash column chromatography on neutral alumina (cyclohexane:triethylamine = 20:1 → cyclohexane:dichloromethane = 3:1) afforded amine (R_a)-**4o** (52.4 mg. 0.088 mmol, 44%, 72% *ee*) and crude silylamine (S_a,^{Si}S*)-**5oa** (73.1 mg, d.r. = 91:9) as off-white solids.

Silylamine (S_a,^{Si}S*)-**5oa** (73.1 mg, d.r. = 91:9) was deprotected according to **GP 9** to afford amine (S_a)-**4o** (52.6 mg. 0.088 mmol, 44%, 76% *ee*) as an off-white solid.

Analytical data for amine (R_a)-**4o**:
The NMR spectroscopic data are in agreement with those reported in section 4 for the racemic amine.
Optical Rotation $[\alpha]_D^{RT}$ = 38.7 (c 0.5, $CHCl_3$, 72% *ee*). The enantiomeric excess of (R_a)-**4o** was determined by HPLC analysis on a chiral stationary phase (*Daicel Chiralpak*® AD-H column, column temperature 20°C, mobile phase *n*-heptane:isopropanol = 97:3, flow rate: 0.6 mL/min, λ = 254 nm): t_R =7.4 min (major), t_R = 8.9 min (minor).

Analytical data for silylamine (S_a,^{Si}S*)-**5oa**:
^{1}H NMR selected signals for the major diastereomer (500 MHz, C_6D_6, 298 K) *δ*/ppm = 5.72 (d, *J* = 6.1 Hz, 1H), 5.16 (d, *J* = 3.1 Hz, 1H), 4.74 (d, *J* = 6.0 Hz, 1H), 4.28 (d, *J* = 3.1 Hz, 1H), 0.72 (s, 9H), 0.26 (s, 9H), 0.25 (s, 9H).
^{1}H NMR selected signals for the minor diastereomer (500 MHz, C_6D_6, 298 K) *δ*/ppm = 4.79 (d, *J* = 6.0 Hz, 1H), 0.68 (s, 9H).
^{1}H/^{29}Si HMQC NMR (500/99 MHz, C_6D_6, 298 K, optimized for *J* = 7 Hz) *δ*/ppm = 5.16/−9.8, 0.26/−4.8.
HRMS (APCI) calculated for $C_{49}H_{57}N_2Si_3^+$ [(M+H)]$^+$: 757.3824; found: 757.3833.

Analytical data for amine (S_a)-**4o**:
The NMR spectroscopic data are in agreement with those reported in section 4 for the racemic amine.
Optical Rotation $[\alpha]_D^{RT}$ = −41.1 (c 0.5, $CHCl_3$, 76% *ee*). The enantiomeric excess of (S_a)-**4o** was determined by HPLC analysis on a chiral stationary phase (*Daicel Chiralpak*® AD-H column, column temperature 20°C, mobile phase *n*-heptane:isopropanol = 97:3, flow rate: 0.6 mL/min, λ = 254 nm): t_R =7.4 min (minor), t_R = 8.9 min (major).

(R_a)-N^2-benzhydryl-6,6'-diimethoxy-[1,1'-binaphthalene]-2,2'-diamine [(R_a)-4p], (S_a)-N^2-benzhydryl-6,6'-dimethoxy-[1,1'-binaphthalene]-2,2'-diamine [(S_a)-4p] and (S_a,^{Si}S*)-N^2-benzhydryl-6,6'-dimethoxy-$N^{2'}$-(*tert*-butyl(phenyl)silyl)-[1,1'-binaphthalene]-2,2'-diamine [(S_a,^{Si}S*)-5pa]

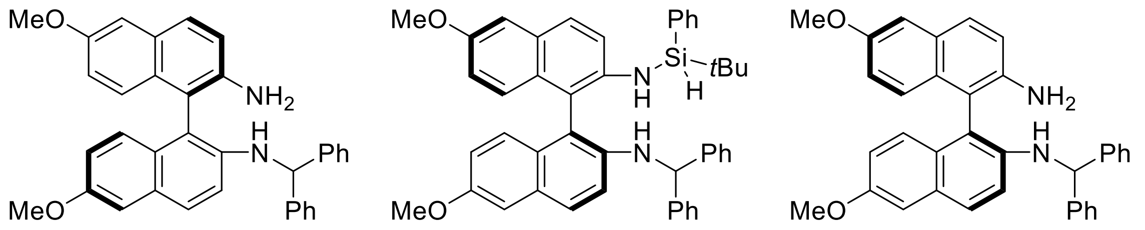

(R_a)-**4p** $C_{35}H_{30}N_2O_2$ M = 510.64 g/mol

(S_a,^{Si}S*)-**5pa** $C_{45}H_{44}N_2O_2Si$ M = 672.94 g/mol

(S_a)-**4p** $C_{35}H_{30}N_2O_2$ M = 510.64 g/mol

Prepared according to **GP 8** from amine *rac*-**4p** (89.8 mg, 0.18 mmol, 1.00 equiv.) and *tert*-butyl(phenyl)silane (20.2 mg, 0.12 mmol, 0.70 equiv.). The reaction was stirred for 24 h. Purification by flash column chromatography on neutral alumina (cyclohexane:triethylamine = 15:1 → dichloromethane) afforded amine (R_a)-**4p** (46.1 mg. 0.09 mmol, 49%, 74% *ee*) and crude silylamine (S_a,^{Si}S*)-**5pa** (46.6 mg, d.r. = 89:11) as off-white solids.

Silylamine (S_a,^{Si}S*)-**5pa** (49.4 mg, dr = 89:11) was deprotected according to **GP 9** to afford amine (S_a)-**4p** (32.7 mg. 0.06 mmol, 36%, 74% *ee*) as an off-white solid.

Analytical data for amine (R_a)-**4p**:
The NMR spectroscopic data are in agreement with those reported in section 4 for the racemic amine.
Optical Rotation $[\alpha]_D^{RT}$ = +40.3 (c 0.59, $CHCl_3$, 73% *ee*). The enantiomeric excess of (R_a)-**4p** was determined by HPLC analysis on a chiral stationary phase (*Daicel Chiralpak*® AD-H column, column temperature 20°C, mobile phase *n*-heptane:isopropanol = 95:5, flow rate: 0.6 mL/min, λ = 254 nm): t_R =56.6 min (major), t_R = 61.2 min (minor).

Analytical data for silylamine (S_a,^{Si}S*)-**5pa**:
^{1}H NMR selected signals for the major diastereomer (500 MHz, C_6D_6, 298 K) 5.71 (d, J = 5.8 Hz, 1H), 5.20 (d, J = 3.0 Hz, 1H), 4.56 (d, J = 5.7 Hz, 1H), 4.12 (d, J = 3.0 Hz, 1H), 3.39 (s, 3H), 3.34 (s, 3H), 0.76 (s, 9H).
^{1}H NMR selected signals for the minor diastereomer (500 MHz, C_6D_6, 298 K) δ/ppm 5.16 (d, J = 2.8 Hz, 1H), 4.63 (d, J = 5.6 Hz, 1H), 4.14 (d, J = 2.8 Hz, 1H), 0.73 (s, 9H).
^{1}H/^{29}Si HMQC NMR (500/99 MHz, C_6D_6, 298 K, optimized for J = 200 Hz) δ/ppm = 5.20/−10.0.
HRMS (APCI) calculated for $C_{45}H_{45}N_2O_2Si^+$ $[(M+H)]^+$: 673.3245; found: 673.3245.

Analytical data for amine (S_a)-**4p**:

The NMR spectroscopic data are in agreement with those reported in section 4 for the racemic amine.

Optical Rotation $[\alpha]_D^{RT}$ = −41.2 (c 0.49, $CHCl_3$, 74% *ee*). The enantiomeric excess of (S_a)-**4p** was determined by HPLC analysis on a chiral stationary phase (*Daicel Chiralpak*® AD-H column, column temperature 20°C, mobile phase *n*-heptane:isopropanol = 95:5, flow rate: 0.6 mL/min, λ = 254 nm): t_R =56.6 min (minor), t_R = 61.1 min (major).

(*R*a)-*N*2-benzhydryl-7,7’-dimethyl-[1,1'-binaphthalene]-2,2'-diamine [(*R*a)-4q], (*S*a)-*N*2-benzhydryl-7,7’-dibromo-[1,1'-binaphthalene]-2,2'-diamine and (*S*a,SiS*)-*N*2-benzhydryl-7,7'-dibromo-*N*2'-(*tert*-butyl(phenyl)silyl)-[1,1'-binaphthalene]-2,2'-diamine [(*S*a,SiS*)-5qa]

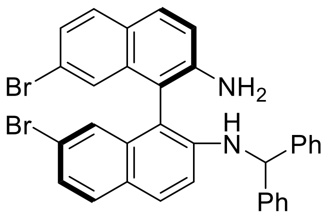

(R_a)-**4q**
$C_{33}H_{24}Br_2N_2$
M = 608.38 g/mol

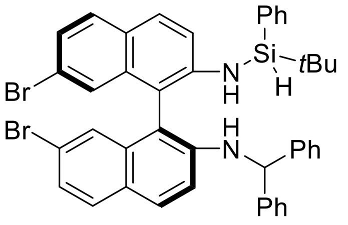

(S_a,${}^{Si}S$*)-**5qa**
$C_{43}H_{38}Br_2N_2Si$
M = 770.68 g/mol

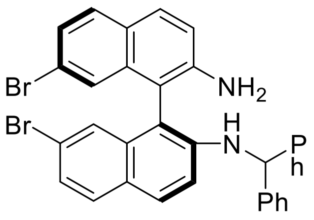

(S_a)-**4q**
$C_{33}H_{24}Br_2N_2$
M = 608.38 g/mol

Prepared according to **GP 8** from amine *rac*-**4q** (121.7 mg, 0.20 mmol, 1.00 equiv.) and *tert*-butyl(phenyl)silane (23.0 mg, 0.14 mmol, 0.70 equiv.). The reaction was stirred for 3 d. Purification by flash column chromatography on neutral alumina (cyclohexane:triethylamine = 20:1 → cyclohexane:dichloromethane = 3:1 afforded amine (R_a)-**4q** (70.5 mg. 0.12 mmol, 58%, 41% *ee*) and crude silylamine (S_a,${}^{Si}S$*)-**5qa** (50.3 mg, d.r. = 88:12) as off-white solids.

Silylamine (S_a,${}^{Si}S$*)-**5qa** (33.1 mg, d.r. = 88:12) was deprotected according to **GP 9** to afford amine (S_a)-**4q** (32.1. 0.05 mmol, 26%, 76% *ee*) as an off-white solid.

Analytical data for amine (R_a)-**4q**:

The NMR spectroscopic data are in agreement with those reported in section 4 for the racemic amine.

Optical Rotation $[\alpha]_D^{RT}$ = +37.6 (c 0.51, $CHCl_3$, 41% *ee*). The enantiomeric excess of (R_a)-**4q** was determined by HPLC analysis on a chiral stationary phase (*Daicel Chiralpak*® AD-H column, column temperature 20°C, mobile phase *n*-heptane:isopropanol = 95:5, flow rate: 0.6 mL/min, λ = 254 nm): t_R =26.7 min (major), t_R = 30.0 min (minor).

Analytical data for silylamine (S_a,^{Si}S*)-**5qa**:

^{1}H NMR selected signals for the major diastereomer (700 MHz, C_6D_6, 298 K) *δ*/ppm = 5.58 (d, *J* = 5.7 Hz, 1H), 5.10 (d, *J* = 2.9 Hz, 1H), 4.53 (d, *J* = 5.7 Hz, 1H), 4.03 (d, *J* = 2.9 Hz, 1H), 0.62 (s, 9H).
^{1}H NMR selected signals for the minor diastereomer (700 MHz, C_6D_6, 298 K) *δ*/ppm = 5.61 (d, *J* = 5.9 Hz, 1H), 5.06 (d, *J* = 2.8 Hz, 1H), 4.59 (d, *J* = 5.8 Hz, 1H), 4.11 (d, *J* = 2.9 Hz, 1H), 0.66 (s, 9H).
^{1}H/^{29}Si HMQC NMR (500/99 MHz, C_6D_6, 298 K, optimized for *J* = 200 Hz) *δ*/ppm = 5.10/−10.0.
HRMS (APCI) calculated for $C_{43}H_{39}Br_2N_2Si^+$ [(M+H)]$^+$: 769.1244; found: 769.1243.

Analytical data for amine (S_a)-**4q**:
The NMR spectroscopic data are in agreement with those reported in section 4 for the racemic amine.
Optical Rotation $[\alpha]_D^{RT}$ = −74.3 (c 0.56, $CHCl_3$, 76% *ee*). The enantiomeric excess of (S_a)-**4q** was determined by HPLC analysis on a chiral stationary phase (*Daicel Chiralpak*® AD-H column, column temperature 20°C, mobile phase *n*-heptane:isopropanol = 95:5, flow rate: 0.6 mL/min, λ = 254 nm): t_R =26.7 min (minor), t_R = 30.1 min (major).

(*R*a)-*N*²-benzhydryl-7,7'-dimethyl-[1,1'-binaphthalene]-2,2'-diamine [(*R*a)-4r], (*S*a)-*N*²-benzhydryl-7,7'-dimethyl-[1,1'-binaphthalene]-2,2'-diamine [(*S*a)-4r] and (*S*a,^{Si}*S)-*N*²-benzhydryl-7,7'-dimethyl-*N*²'-(*tert*-butyl(phenyl)silyl)-[1,1'-binaphthalene]-2,2'-diamine [(*S*a,^{Si}*S**)-5ra]**

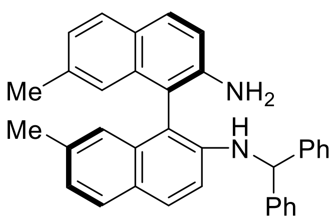

(R_a)-**4r**
$C_{35}H_{30}N_2$
M = 478.64 g/mol

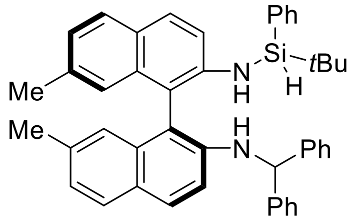

(S_a,^{Si}S*)-**5ra**
$C_{45}H_{44}N_2Si$
M = 640.95 g/mol

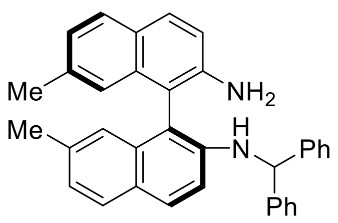

(S_a)-**4r**
$C_{35}H_{30}N_2$
M = 478.64 g/mol

Prepared according to **GP 8** from amine *rac*-**4r** (95.7 mg, 0.20 mmol, 1.00 equiv.) and *tert*-butyl(phenyl)silane (23.0 mg, 0.14 mmol, 0.70 equiv.). The reaction was stirred for 6 d. Purification by flash column chromatography on neutral alumina (cyclohexane:triethylamine = 20:1 → cyclohexane:dichloromethane = 3:1) afforded amine (R_a)-**4r** (64.0 mg. 0.13 mmol, 67%, 23% *ee*) and crude silylamine (S_a,^{Si}S*)-**5ra** (33.1 mg, d.r. = 88:12) as off-white solids.

Silylamine (S_a,^{Si}S*)-**5ra** (33.1 mg, d.r. = 88:12) was deprotected according to **GP 9** to afford amine (S_a)-**4r** (18.5. 0.04 mmol, 19%, 80% *ee*) as an off-white solid.

Analytical data for amine (R_a)-**4r**:
The NMR spectroscopic data are in agreement with those reported in section 4 for the racemic amine.

Optical Rotation $[\alpha]_D^{RT}$ = +23.5 (c 0.62, $CHCl_3$, 23% *ee*). The enantiomeric excess of (R_a)-**4r** was determined by HPLC analysis on a chiral stationary phase (*Daicel Chiralpak*® IA column, column temperature 20°C, mobile phase *n*-heptane:isopropanol = 97:3, flow rate: 0.6 mL/min, λ = 250 nm): t_R =20.4 min (major), t_R = 22.7 min (minor).

Analytical data for silylamine (S_a,^{Si}S*)-**5ra**:

^{1}H NMR selected signals for the major diastereomer (500 MHz, C_6D_6, 298 K) *δ*/ppm = 5.70 (d, *J* = 5.9 Hz, 1H), 5.17 (d, *J* = 3.0 Hz, 1H), 4.68 (d, *J* = 6.0 Hz, 1H), 4.17 (d, *J* = 3.1 Hz, 1H), 2.06 (s, 3H), 2.04 (s, 3H), 0.68 (s, 9H).

^{1}H NMR selected signals for the minor diastereomer (500 MHz, C_6D_6, 298 K) *δ*/ppm = 5.73 (d, *J* = 6.3 Hz, 1H), 4.74 (d, *J* = 6.1 Hz, 1H), 4.24 (d, *J* = 2.7 Hz, 1H), 2.03 (s, 3H), 0.69 (s, 9H).

^{1}H/^{29}Si HMQC NMR (500/99 MHz, C_6D_6, 298 K, optimized for *J* = 200 Hz) *δ*/ppm = 5.17/−10.0.

HRMS (APCI) calculated for $C_{45}H_{45}N_2Si^+$ $[(M+H)]^+$: 641.3347; found: 641.3352.

Analytical data for amine (S_a)-**4r**:

The NMR spectroscopic data are in agreement with those reported in section 4 for the racemic amine.

Optical Rotation $[\alpha]_D^{RT}$ = −81.4 (c 0.29, $CHCl_3$, 80% *ee*). The enantiomeric excess of (S_a)-**4r** was determined by HPLC analysis on a chiral stationary phase (*Daicel Chiralpak*® IA column, column temperature 20°C, mobile phase *n*-heptane:isopropanol = 97:3, flow rate: 0.6 mL/min, λ = 254 nm): t_R =21.0 min (minor), t_R = 23.7 min (major).

(*R*a)-*N*²-benzhydryl-7,7'-diphenyl-[1,1'-binaphthalene]-2,2'-diamine [(*R*a)-4s], (*S*a)-*N*²-benzhydryl-7,7'-diphenyl-[1,1'-binaphthalene]-2,2'-diamine [(*S*a)-4s] and (*S*a,^Si^*S)-*N*²-benzhydryl-7,7'-diphenyl-*N*²'-(*tert*-butyl(phenyl)silyl)-[1,1'-binaphthalene]-2,2'-diamine [(*S*a,^Si^*S**)-5sa]**

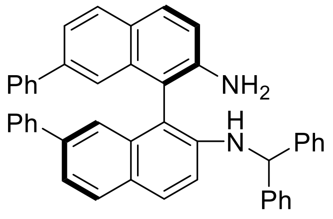

(R_a)-**4s**
$C_{45}H_{34}N_2$
M = 602.78 g/mol

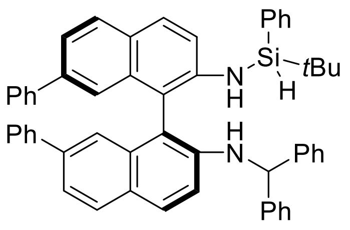

(S_a,^{Si}S*)-**5sa**
$C_{55}H_{48}N_2Si$
M = 765.09 g/mol

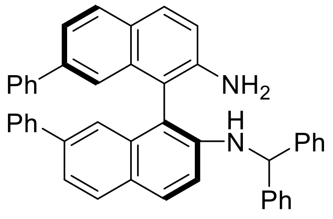

(S_a)-**4s**
$C_{45}H_{34}N_2$
M = 602.78 g/mol

Prepared according to **GP 8** from amine *rac*-**4s** (120.6 mg, 0.20 mmol, 1.00 equiv.) and *tert*-butyl(phenyl)silane (23.0 mg, 0.14 mmol, 0.70 equiv.). The reaction was stirred for 12 d. Purification by flash column chromatography on neutral alumina (cyclohexane:triethylamine = 20:1 → cyclohexane:dichloromethane = 3:1) afforded amine (R_a)-**4s** (73.5 mg. 0.12 mmol, 61%, 18% *ee*) and crude silylamine (S_a,^{Si}S*)-**5sa** (22.3 mg, d.r. = 87:13) as off-white solids.

Silylamine (S_a,^{Si}S*)-**5sa** (22.3 mg, d.r. = 87:13) was deprotected according to **GP 9** to afford amine (S_a)-**4s** (11.2 mg. 0.02 mmol, 9%, 73% *ee*) as an off-white solid.

Analytical data for amine (R_a)-**4s**:
The NMR spectroscopic data are in agreement with those reported in section 4 for the racemic amine.
Optical Rotation $[\alpha]_D^{RT}$ = −11.7 (c 0.64, $CHCl_3$, 18% *ee*). The enantiomeric excess of (R_a)-**4s** was determined by HPLC analysis on a chiral stationary phase (*Daicel Chiralpak*® AD-H column, column temperature 20°C, mobile phase *n*-heptane:isopropanol = 90:10, flow rate: 0.6 mL/min, λ = 280 nm): t_R =33.4 min (major), t_R = 39.7 min (minor).

Analytical data for silylamine (S_a,^{Si}S*)-**5sa**:
^{1}H NMR selected signals for the major diastereomer (500 MHz, C_6D_6, 298 K) *δ*/ppm = 5.63 (d, *J* = 5.8 Hz, 1H), 5.14 (d, *J* = 2.8 Hz, 1H), 4.73 (d, *J* = 5.9 Hz, 1H), 4.20 (d, *J* = 2.9 Hz, 1H), 0.63 (s, 9H).
^{1}H NMR selected signals for the minor diastereomer (500 MHz, C_6D_6, 298 K) *δ*/ppm = 5.67 (d, *J* = 6.0 Hz, 1H), 4.78 (d, *J* = 6.0 Hz, 1H), 4.28 (d, *J* = 2.7 Hz, 1H).
^{1}H/^{29}Si HMQC NMR (500/99 MHz, C_6D_6, 298 K, optimized for *J* = 200 Hz) *δ*/ppm = 5.14/−9.9.
HRMS (APCI) calculated for $C_{55}H_{49}N_2Si^+$ $[(M+H)]^+$: 765.3660; found: 765.3669.

Analytical data for amine (S_a)-**4s**:
The NMR spectroscopic data are in agreement with those reported in section 4 for the racemic amine.

Optical Rotation $[\alpha]_D^{RT}$ = +42.6 (c 0.19, $CHCl_3$, 73% *ee*). The enantiomeric excess of (S_a)-**4s** was determined by HPLC analysis on a chiral stationary phase (*Daicel Chiralpak*® AD-H column, column temperature 20°C, mobile phase *n*-heptane:isopropanol = 90:10, flow rate: 0.6 mL/min, λ = 280 nm): t_R =33.4 min (minor), t_R = 39.7 min (major).

(*R*a)-*N*2-benzhydryl-7,7'-diimethoxy-[1,1'-binaphthalene]-2,2'-diamine [(*R*a)-4t], (*S*a)-*N*2-benzhydryl-7,7'-dimethoxy-[1,1'-binaphthalene]-2,2'-diamine [(*S*a)-4t] and (*S*a,SiS*)-*N*2-benzhydryl-7,7'-dimethoxy-*N*2'-(*tert*-butyl(phenyl)silyl)-[1,1'-binaphthalene]-2,2'-diamine [(*S*a,SiS*)-5ta]

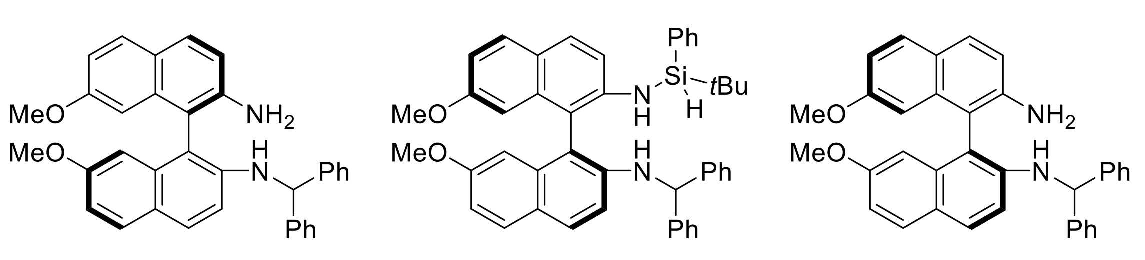

(R_a)-**4t** $C_{35}H_{30}N_2O_2$ M = 510.64 g/mol

(S_a,^{Si}S*)-**5ta** $C_{45}H_{44}N_2O_2Si$ M = 672.94 g/mol

(S_a)-**4t** $C_{35}H_{30}N_2O_2$ M = 510.64 g/mol

Prepared according to **GP 8** from amine *rac*-**4t** (102.1 mg, 0.20 mmol, 1.00 equiv.) and *tert*-butyl(phenyl)silane (23.0 mg, 0.14 mmol, 0.70 equiv.). The reaction was stirred for 10 d. Purification by flash column chromatography on neutral alumina (cyclohexane:triethylamine = 20:1 → dichloromethane) afforded amine (R_a)-**4t** (63.8 mg. 0.12 mmol, 62%, 19% *ee*) and crude silylamine (S_a,${}^{Si}S^*$)-**5ta** (20.1 mg, d.r. = 91:9) as off-white solids.

Silylamine (S_a,${}^{Si}S^*$)-**5ta** (20.1 mg, dr = 91:9) was deprotected according to **GP 9** to afford amine (S_a)-**4t** (11.3 mg. 0.02 mmol, 11%, 88% *ee*) as an off-white solid.

Analytical data for amine (R_a)-**4t**:
The NMR spectroscopic data are in agreement with those reported in section 4 for the racemic amine.
Optical Rotation $[\alpha]_D^{RT}$ = +10.3 (c 0.53, $CHCl_3$, 19% *ee*). The enantiomeric excess of (R_a)-**4t** was determined by HPLC analysis on a chiral stationary phase (*Daicel Chiralpak*® AD-H column, column temperature 20°C, mobile phase *n*-heptane:isopropanol = 90:10, flow rate: 0.6 mL/min, λ = 254 nm): t_R =19.4 min (major), t_R = 25.0 min (minor).

Analytical data for silylamine (S_a,${}^{Si}S^*$)-**5ta**:
^{1}H NMR selected signals for the major diastereomer (500 MHz, C_6D_6, 298 K) 5.71 (d, J = 5.8 Hz, 1H), 5.20 (d, J = 3.0 Hz, 1H), 4.56 (d, J = 5.7 Hz, 1H), 4.12 (d, J = 3.0 Hz, 1H), 3.39 (s, 3H), 3.34 (s, 3H), 0.76 (s, 9H).
^{1}H NMR selected signals for the minor diastereomer (500 MHz, C_6D_6, 298 K) δ/ppm 5.72 (d, J = 6.2 Hz, 1H), 5.17 (d, J = 3.2 Hz, 1H), 4.73 (d, J = 6.1 Hz, 1H), 4.26 (d, J = 3.1 Hz, 1H), 3.16 (s, 3H), 0.74 (s, 9H).
^{1}H/^{29}Si HMQC NMR (500/99 MHz, C_6D_6, 298 K, optimized for J = 200 Hz) δ/ppm = 5.17/−9.9.
HRMS was not obtained due to almost complete decomposition.

Analytical data for amine (S_a)-**4t**:
The NMR spectroscopic data are in agreement with those reported in section 4 for the racemic amine.
Optical Rotation $[\alpha]_D^{RT}$ = −55.6 (c 0.16, $CHCl_3$, 88% *ee*). The enantiomeric excess of (S_a)-**4t** was determined by HPLC analysis on a chiral stationary phase (*Daicel Chiralpak*® AD-H column, column temperature 20°C, mobile phase *n*-heptane:isopropanol = 90:10, flow rate: 0.6 mL/min, λ = 254 nm): t_R =19.4 min (minor), t_R = 24.9 min (major).

(*R*a)-*N*[2]-benzhydryl-4,4'-dimethyl-[1,1'-binaphthalene]-2,2'-diamine [(*R*a)-4u], (*S*a)-*N*[2]-benzhydryl-4,4'-dimethyl-[1,1'-binaphthalene]-2,2'-diamine [(*S*a)-4u] and (*S*a,SiS*)-*N*[2]-benzhydryl-4,4'-dimethyl-*N*[2']-(*tert*-butyl(phenyl)silyl)-[1,1'-binaphthalene]-2,2'-diamine [(*S*a,SiS*)-5ua]

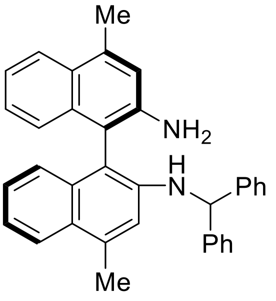

(*R*a)-**4u**
$C_{35}H_{30}N_2$
M = 478.64 g/mol

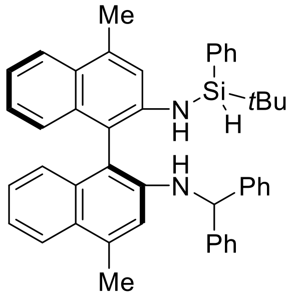

(*S*a,Si*S**)-**5ua**
$C_{45}H_{44}N_2Si$
M = 640.95 g/mol

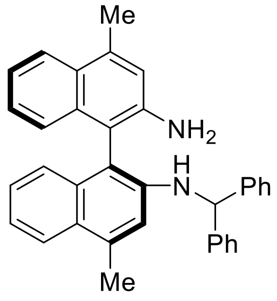

(*S*a)-**4u**
$C_{35}H_{30}N_2$
M = 478.64 g/mol

Prepared according to **GP 8** from amine *rac*-**4u** (95.7 mg, 0.20 mmol, 1.00 equiv.) and *tert*-butyl(phenyl)silane (23.0 mg, 0.14 mmol, 0.70 equiv.). The reaction was stirred for 7 d. Purification by flash column chromatography on neutral alumina (cyclohexane:triethylamine = 20:1 → cyclohexane:dichloromethane = 3:1) afforded amine (*R*a)-**4u** (64.2 mg. 0.13 mmol, 67%, 15% *ee*) and crude silylamine (*S*a,Si*S**)-**5ua** (28.7 mg, d.r. = 87:13) as off-white solids.

Silylamine (*S*a,Si*S**)-**5ua** (28.7 mg, d.r. = 87:13) was deprotected according to **GP 9** to afford amine (*S*a)-**4u** (14.2 mg, 0.030 mmol, 15%, 55% *ee*) as an off-white solid.

Analytical data for amine (*R*a)-**4t**:
The NMR spectroscopic data are in agreement with those reported in section 4 for the racemic amine.
Optical Rotation $[\alpha]_D^{RT}$ = +10.0 (c 0.2, $CHCl_3$, 15% *ee*). The enantiomeric excess of (*R*a)-**4t** was determined by HPLC analysis on a chiral stationary phase (*Daicel Chiralpak*® IA column, column temperature 20°C, mobile phase *n*-heptane:isopropanol = 97:3, flow rate: 0.6 mL/min, λ = 250 nm): t_R =23.5 min (major), t_R = 29.3 min (minor).

Analytical data for silylamine (*S*a,Si*S**)-**5ua**:
^{1}H NMR selected signals for the major diastereomer (500 MHz, C_6D_6, 298 K) *δ*/ppm = 5.77 (d, *J* = 6.2 Hz, 1H), 5.22 (d, *J* = 3.1 Hz, 1H), 4.70 (d, *J* = 6.2 Hz, 1H), 4.24 (d, *J* = 3.2 Hz, 1H), 2.45 (s, 3H), 2.20 (s, 3H), 0.74 (s, 9H).
^{1}H NMR selected signals for the minor diastereomer (500 MHz, C_6D_6, 298 K) *δ*/ppm = 5.80 (d, *J* = 6.3 Hz, 1H), 5.18 (d, *J* = 2.7 Hz, 1H), 4.77 (d, *J* = 6.1 Hz, 1H), 4.27 (s, 1H), 0.70 (s, 9H).
^{1}H/^{29}Si HMQC NMR (500/99 MHz, C_6D_6, 298 K, optimized for *J* = 7 Hz) *δ*/ppm = 5.22/−10.2.
HRMS (APCI) calculated for $C_{45}H_{45}N_2Si^+$ $[(M+H)]^+$: 641.3347; found: 641.3353.

Analytical data for amine (S_a)-**4u**:

The NMR spectroscopic data are in agreement with those reported in section 4 for the racemic amine.

Optical Rotation $[\alpha]_D^{RT}$ = −24.0 (c 0.3, $CHCl_3$, 55% *ee*). The enantiomeric excess of (S_a)-**4u** was determined by HPLC analysis on a chiral stationary phase (*Daicel Chiralpak*® IA column, column temperature 20°C, mobile phase *n*-heptane:isopropanol = 97:3, flow rate: 0.6 mL/min, λ = 250 nm): t_R =23.5 min (minor), t_R = 29.2 min (major).

6 Scale-Up Experiment

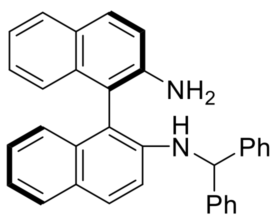

(R_a)-**4f**
$C_{33}H_{26}N_2$
M = 450.59 g/mol

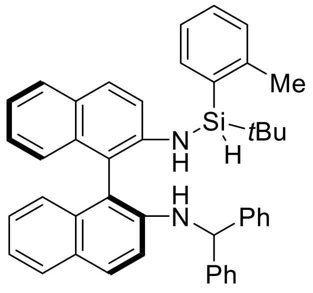

(S_a,${}^{Si}S$*)-**5fi**
$C_{44}H_{42}N_2Si$
M = 626.92 g/mol

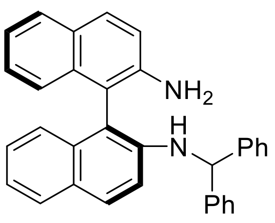

(S_a)-**5f**
$C_{33}H_{26}N_2$
M = 450.59 g/mol

In an argon-filled glovebox, an oven-dried dram vial is charged with MesCu (9.0 mg, 50 µmol, 5.0 mol%), (*R*),(*R*)-Ph-BPE (30 mg, 60 µmol, 6.0 mol%) and C_6D_6 (0.5 mL). The mixture is stirred at room temperature for 5 min, to give a clear, yellow solution. A solution of amine *rac*-**4f** (451 mg, 1.00 mmol, 1.00 equiv.) and *tert*-butyl(2-tolyl)silane (125 mg, 0.70 mmol, 0.70 equiv.) in C_6D_6 (2.5 mL) is added and the reaction mixture is stirred in the glovebox at ambient temperature for 10 d. Purification by flash column chromatography on neutral alumina (cyclohexane:triethylamine = 20:1 → cyclohexane:dichloromethane = 1:1) afforded amine (R_a)-**4f** (323 mg. 0.72 mmol, 72%, 29% *ee*) as an off-white solid and crude silylamine (S_a,${}^{Si}S$*)-**5fi** (176 mg, d.r. = 96:4, isolated with remaining *tert*-butyl(2-tolyl)silane) as a yellow resin.

The spectroscopic data of (R_a)-**4f** and (S_a,${}^{Si}S$*)-**5fi** are consistent with those reported for the 0.20-mmol scale. A small amount of crude silylamine (S_a,${}^{Si}S$*)-**5fi** was deprotected according to **GP 9** and afforded amine (S_a)-**4f** (90% *ee*).

7 Deprotection of the Benzhydryl Group

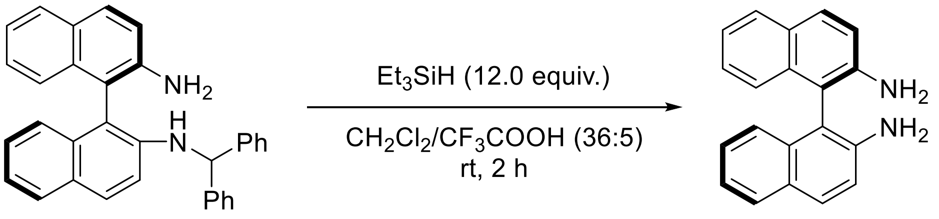

(R_a)-**4f** (R_a)-**4a**

$C_{20}H_{16}N_2$

M = 284.36 g/mol

According to a modified literature procedure,[S30] amine (R_a)-**4f** (100 mg, 222 µmol, 1.00 equiv.) was dissolved in dichloromethane (22 mL). To the solution triethylsilane (310 mg, 0.43 mL, 2.66 mmol, 12.0 equiv.) and trifluoroacetic acid (4.56 g, 3.1 mL, 40.0 mmol, 180 equiv.) was added and the reaction mixture was stirred at ambient temperature for 2 h. Then, the reaction mixture was poured into an aqueous, saturated sodium carbonate solution (100 mL) and the phases were separated. The aqueous layer was extracted with ethyl acetate (20 mL) and the combined organic phases were washed with brine (20 mL), dried over anhydrous Na_2SO_4, and filtered. Volatiles were removed under reduced pressure and the residue was purified by flash column chromatography on silica gel (dichloromethane) to afford diamine (R_a)-**4a** (56.3 mg. 198 µmol, 89%, 31% *ee*) as an off-white solid.

R_f = 0.37.

^{1}H NMR (400 MHz, $CDCl_3$, 298 K) δ/ppm = 7.86–7.76 (m, 4H), 7.28–7.19 (m, 4H), 7.15 (d, J = 8.7 Hz, 2H), 7.12–7.08 (m, 2H), 3.64 (s, 4H).

$^{13}C\{^1H\}$ NMR (100 MHz, $CDCl_3$, 298 K) δ/ppm = 142.8, 133.8, 129.6, 128.6, 128.3, 127.0, 124.1, 122.6, 118.5, 112.7.

Optical Rotation $[\alpha]_D^{RT}$ = +36.2 (c 0.66, $CHCl_3$, 31% *ee*). The enantiomeric excess of (R_a)-**4a** was determined by HPLC analysis on a chiral stationary phase (*Daicel Chiralpak*® AD-H column, column temperature 20°C, mobile phase *n*-heptane:isopropanol = 85:15, flow rate: 0.6 mL/min, λ = 250 nm): t_R =29.0 min (major), t_R = 111.5 min (minor).

The spectroscopic data are in accordance with those reported in the literature.[S28]

8 Determination of Absolute Configuration

The absolute configuration of the enantioenriched amine **4f** was determined by synthesis of enantiopure (*R*)-**4f** and (*S*)-**4f** by employing commercially obtained (*R*)- and (*S*)-BINAM. and comparing the retention times on the chiral HPLC and optical rotation values with the samples obtained in the kinetic resolution. Additionally, the benzhydryl group of **4f** was cleaved and the optical rotation value is consistent with those reported in the literature for (*R*)-BINAM. These analyses point to the enrichment of the (*R*)-amine. Based on these findings, the absolute configurations of the other compounds were assigned by analogy, assuming that the (*S*)-enantiomer undergoes silylation more rapidly in all cases.

The absolute configuration of the silyl amines was not determined.

9 Optimization of Reaction

9.1 Screening of the *N*-Protecting Group

Table S1. Screening of the N-Protecting Group

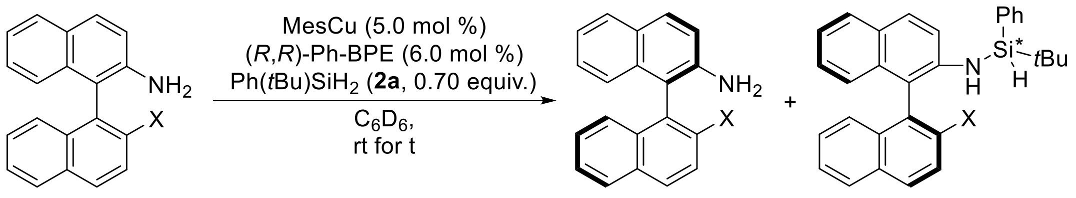

rac-**4a–h**
rac-**S30**

(R_a)-**4a–h**
(R_a)-**S30**

(S_a,^{Si}S*)-**5aa–ha**
(S_a,^{Si}S*)-**S31**

**entry**	**X**	**t**	**conv (%)**[b]	***ee* of (R_a)-4 (%)**[c]	***ee* of (S_a)-4 (%)**[d]	**d.r. of 5**[e]	***s***[f]
1	NH_2 (**4a**)	48 h	n.r.	–	–	–	–
2	NMe_2 (**4b**)	13 days	40	22	30	64:26	2
3	NHMe (**4c**)	60 h	50	52	52	77:23	5
4	NH*i*Pr (**4d**)	60 h	58	68	48	75:25	6
5	NHBn (**4e**)	40 h	48	60	64	83:17	8
6[h]	NH[(CH)Ph_2] (**4f**)	4 days	41	55	79	91:9	15
7	NHTr (**4g**)	48 h	n.r.	–	–	–	–
8	O[(CH)Ph_2] (**4h**)	6 days	42	42	59	n.d.	6
9	Me (**S30**)	60 h	50	54	53	75:25	6

[a]Reactions were performed on a 0.2 mmol scale. The configuration at the silicon atom was not determined. [b]Conversion was monitored by ^{1}H NMR spectroscopy and calculated according to conversion = $ee_{\text{unreacted amine}}/(ee_{\text{unreacted amine}} + ee_{\text{silylamine}}) \times 100$. [c]Determined by HPLC analysis on chiral stationary phases. [d]Determined by HPLC analysis on chiral stationary phases after deprotection of the silylated aniline group. [e]Determined by ^{1}H NMR spectroscopic analysis. [f]$s = \ln[(1 - C)(1 - ee)]/\ln[(1 - C)(1 + ee)]/$, where $ee = ee_{\text{unreacted amine}}/100$ and C = conversion/100. [g]Reaction run in CD_2Cl_2. [h]Reaction run in toluene. n.r. = no reaction. n.d. = not determined

9.2 Screening of Monohydrosilanes

Table S2. Screening of Monohydrosiilanes

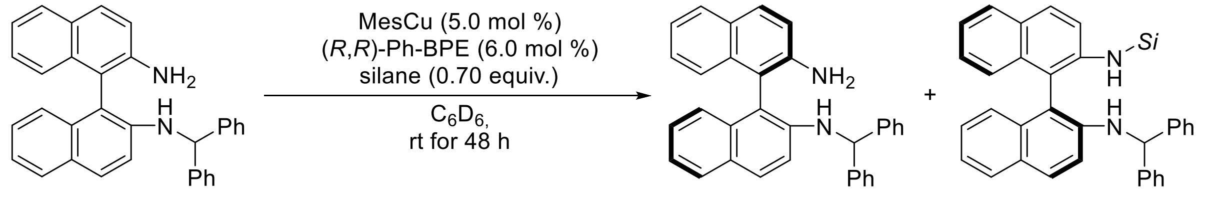

rac-**4f** (R_a)-**4f** (S_a)-**5f**

entry	silane	conv (%)	*ee* of (R_a)-4f (%)	*ee* of (S_a)-4f (%)	d.r. of 5f
1	Ph_3SiH	–	–	–	–
2	Ph_2MeSiH	–	–	–	–
3	$PhMe_2SiH$	–	–	–	–
4	(*t*Bu)Me_2SiH	–	–	–	–
5	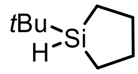	–	–	–	–
6	(*t*Bu)$_2SiH_2$	–	–	–	–

9.3 Screening of Dihydrosilanes

Table S3. Screening of Dihydrosilanes

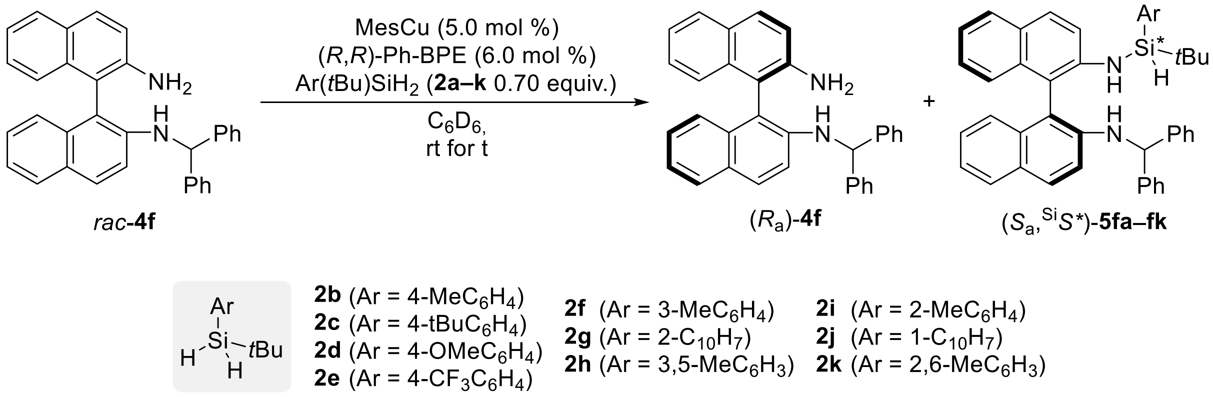

entry	Ar	t	conv (%)	ee of (R_a)-4f (%)	ee of (S_a)-4f (%)	d.r. of 5fa–fk	*s*
1[b]	Ph (**2a**)	4 days	41	55	79	91:9	15
2	4-MeC_6H_4 (**2b**)	6 days	51	76	73	88:12	14
3	4-*t*BuC_6H_4 (**2c**)	8 days	57	90	68	87:13	16
4	4-$OMeC_6H_4$ (**2d**)	6 days	49	70	72	87:13	13
5	4-$CF_3C_6H_4$ (**2e**)	5 days	46	62	73	83:17	12
6	3-MeC_6H_4 (**2f**)	6 days	50	76	75	90:10	16
7	2-$C_{10}H_7$ (**2g**)	4 days	44	62	78	89:11	15
8	3,5-MeC_6H_3 (**2h**)	8 days	50	78	78	90:10	19
9	2-MeC_6H_4 (**2i**)	7 days	36	50	87	96:4	24
10	1-$C_{10}H_7$ (**2j**)	7 days	42	60	84	93:7	21
11	2,6-MeC_6H_3 (**2k**)	4 days	n.r.	–	–	–	–

[a]For details see footnotes of Table S1. [b]Reaction run in toluene

With silane **S2**: Conversion obtained by 1H NMR spectroscopic analysis of the reaction mixture does not match conversion calculated according to the Kagan equation, decomposition occurs during purification (column chromatography). *s* value in parentheses calculated using the enantiomeric excess of the silyamine and conversion according to NMR.

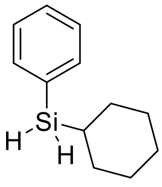

S2

42% conversion (4 d, NMR)[c]
(R_a)-NH_2: 30% *ee*
(S_a)-NH_2: 70% *ee*
d.r. (NHSi) = 80:20
***s* = 7.6 (9.3)**

10 References

[S1] Zhu, J, Chen, S.; He, C. Catalytic Enantioselective Dehydrogenative Si–O Coupling to Access Chiroptical Silicon-Stereogenic Siloxanes and Alkoxysilanes, *J. Am. Chem. Soc.* **2021**, *143*, 5301–5307. DOI: 10.1021/jacs.1c01106.

[S2] Chang, X.; Zhang, Q.; Guo, C. Switchable Smiles Rearrangement for Enantioselective *O*-Aryl Amination, *Org. Lett.* **2019**, *21*, 4915–4918. DOI: 10.1021/acs.orglett.9b01848

[S3] Wu, M; Chen, Y.-W.; Lu, Q.; Wang, Y.-B.; Cheng, J. K.; Yu, P.; Tan, B. *Organocatalytic* Si–C_{Aryl} Bond Functionalization-Enabled Atroposelective Synthesis of Axially Chiral Biaryl Siloxanes *J. Am. Chem. Soc.* **2023**, *145*, 20646–20654. DOI: 10.1021/jacs.3c07839.

[S4] Vyskočil, S.; Smrčina, M.; Lorenc M.; Tišlerová, I.; Brooks, R. D.; Kulagowski, J. J.; Langer, V.; Farrugia, L. J.; Kočovský, P. Copper(II)-Mediated Oxidative Coupling of 2-Aminonaphthalene Homologues. Competition between the Straight Dimerization and the Formation of Carbazoles *J. Org. Chem.* **2001**, *66*, 1359–1365. DOI: 10.1021/jo005691w.

[S5] Pape, F.; Thiel, N.O.; Teichert, J. F. *Z*-Selective Copper(I)-Catalyzed Alkyne Semihydrogenation with Tethered Cu–Alkoxide Complexes *Chem. Eur. J.* **2015**, *21*, 15934–15938. DOI: 10.1002/chem.201501739.

[S6] Rendler, S.; Plefka, O.; Karatas, B.; Auer, G., Fröhlich, R.; Mück-Lichtenfeld, C.; Grimme, S.; Oestreich, M. Stereoselective Alcohol Silylation by Dehydrogenative Si–O Coupling: Scope, Limitations, and Mechanism of the Cu–H-Catalyzed Non-Enzymatic Kinetic Resolution with Silicon-Stereogenic Silanes *Chem. Eur. J.* **2008**, *14*, 11512–11528. DOI: 10.1002/chem.200801377.

[S7] Hurkes, N.; Spirk, S.; Belaj, F.; Pietschnig, R. At the Edge of Stability – Preparation of Methyl-substituted Arylsilanetriols and Investigation of their Condensation Behavior *Z. Anorg. Allg. Chem.* **2013**, *639*, 2631–2636. DOI: 10.1002/zaac.201300349.

[S8] Homsi, F.; Hosoi, K.; Nozaki, K.; Hiyama, T. Solid Phase Cross-Coupling Reaction of Aryl(Halo)Silanes with 4-Iodobenzoic Acid. *J. Organomet. Chem.* **2001**, *624*, 208–216. DOI: 10.1016/S0022-328X(01)00664-7.

[S9] Wang, K.; Zhou, J.; Jiang, Y.; Zhang, M.; Wang, C.; Xue, D.; Tang, W.; Sun, H.; Xiao, J.; Li, C. Selective Manganese-Catalyzed Oxidation of Hydrosilanes to Silanols under Neutral Reaction Conditions. *Angew. Chem. Int. Ed.* **2019**, *58*, 6380–6384. DOI: 10.1002/anie.201900342. *Angew. Chem.* **2019**, *131*, 6446–6450.

[S10] Igawa, K.; Yoshihiro, D.; Ichikawa, N.; Kokan, N.; Tomooka, K. Catalytic Enantioselective Synthesis of Alkenylhydrosilanes. *Angew. Chem., Int. Ed.* **2012**, *51*, 12745–12748. DOI: 10.1002/anie.201207361. *Angew. Chem.* **2012**, *124*, 12917–12920.

[S11] Yang, W.; Liu, L.; Guo, J.; Wang, S.-G.; Zhang, J.-Y.; Fan, L.-W.; Tian, Y.; Wang, L.-L.; Luan, C.; Li, Z.-L.; et al. Enantioselective Hydroxylation of Dihydrosilanes to Si-Chiral Silanols Catalyzed by In Situ Generated Copper(II) Species. *Angew. Chem., Int. Ed.* **2022**, *61*, e202205743. DOI: 10.1002/anie.202205743. *Angew. Chem.* **2022**, *134*, e202205743.

[S12] Čapková, K.; Yoneda, Y.; Dickerson, T. J.; Janda, K. D. Synthesis and Structure–Activity Relationships of Second-Generation Hydroxamate Botulinum Neurotoxin A Protease Inhibitors. *Bioorg. Med. Chem. Lett.* **2007**, *17*, 6463–6466. DOI: 10.1016/j.bmcl.2007.09.103.

[S13] Miró, J.; Gensch, T.; Ellwart, M.; Han, S.-J.; Lin, H.-H.; Sigman, M. S.; Toste, F. D. Enantioselective Allenoate-Claisen Rearrangement Using Chiral Phosphate Catalysts. *J. Am. Chem. Soc.* **2020**, *142*, 6390–6399. DOI: 10.1021/jacs.0c01637.

[S14] Uiterweerd, M. T.; Minnaard, A. J. Racemic Total Synthesis of Elmonin and Pratenone A, from Streptomyces, Using a Common Intermediate Prepared by Peri-Directed C–H Functionalization. *Org. Lett.* **2022**, *24*, 9361–9365. DOI: 10.1021/acs.orglett.2c03449.

[S15] Ghosh, M. K.; Rzymkowski, J.; Kalek, M. Transition-Metal-Free Aryl–Aryl Cross-Coupling: C−H Arylation of 2-Naphthols with Diaryliodonium Salts. *Chem. Eur. J.* **2019**, *25*, 9619–9623. DOI: 10.1002/chem.201902204

[S16] Qiu, H.; Shuai, B.; Wang, Y.-Z.; Liu, D.; Chen, Y.-G.; Gao, P.-S.; Ma, H.-X.; Chen, S.; Mei, T.-S. Enantioselective Ni-Catalyzed Electrochemical Synthesis of Biaryl Atropisomers. *J. Am. Chem. Soc.* **2020**, *142*, 9872–9878. DOI: 10.1021/jacs.9b13117.

[S17] He, Z.; Pulis, A. P.; Procter, D. J. The Interrupted Pummerer Reaction in a Sulfoxide-Catalyzed Oxidative Coupling of 2-Naphthols. *Angew. Chem., Int. Ed.* **2019**, *58*, 7813–7817. DOI: 10.1002/anie.201903492. *Angew. Chem.* **2019**, *131*, 7895–7899.

[S18] Chen, Y.-H.; Qi, L.-W.; Fang, F.; Tan, B. Organocatalytic Atroposelective Arylation of 2-Naphthylamines as a Practical Approach to Axially Chiral Biaryl Amino Alcohols. *Angew. Chem., Int. Ed.* **2017**, *56*, 16308–16312. DOI: 10.1002/anie.201710537. *Angew. Chem.* **2017**, *129*, 16526–16530.

[S19] Liu, W.; Jiang, Q.; Yang, X. A Versatile Method for Kinetic Resolution of Protecting-Group-Free BINAMs and NOBINs through Chiral Phosphoric Acid Catalyzed Triazane Formation. *Angew. Chem., Int. Ed.* **2020**, *59*, 23598–23602. DOI: 10.1002/anie.202009395. *Angew. Chem.* **2020**, *132*, 23804–23808.

[S20] Wang, J.; Li, H.; Duan, W.; Zu, L.; Wang, W. Organocatalytic Asymmetric Michael Addition of 2,4-Pentandione to Nitroolefins. *Org. Lett.* **2005**, *7*, 4713–4716. DOI: 10.1021/ol0519137.

[S21] Pupo, G.; Ibba, F.; Ascough, D. M. H.; Vicini, A. C.; Ricci, P.; Christensen, K. E.; Pfeifer, L.; Morphy, J. R.; Brown, J. M.; Paton, R. S.; et al. Asymmetric Nucleophilic Fluorination under Hydrogen Bonding Phase-Transfer Catalysis. *Science* **2018**, *360*, 638–642. DOI: 10.1126/science.aar7941.

[S22] Gao, B.; Li, D.; Li, X.; Duan, R.; Pang, X.; Cui, Y.; Duan, Q.; Chen, X. Preparation of Biocompatible, Biodegradable and Sustainable Polylactides Catalyzed by Aluminum Complexes Bearing Unsymmetrical Dinaphthalene-Imine Derivatives via Ring-Opening Polymerization of Lactides. *Catal. Sci. Technol.* **2015**, *5*, 4644–4652. DOI:10.1039/C5CY00982K.

[S23] Rohde, V. H. G.; Müller, M. F.; Oestreich, M. Intramolecularly Sulfur-Stabilized Silicon Cations with Chiral Binaphthyl Backbones: Synthesis of Three Different Motifs and Their Application in

Enantioselective Diels–Alder Reactions. *Organometallics* **2015**, *34*, 3358–3373. DOI: 10.1021/acs.organomet.5b00351.

[S24] De, C. K.; Pesciaioli, F.; List, B. Catalytic Asymmetric Benzidine Rearrangement. *Angew. Chem., Int. Ed.* **2013**, *52*, 9293–9295. DOI: 10.1002/anie.201304039. *Angew. Chem.* **2013**, *125*, 9463–9465.

[S25] Zhu, B.Y.; Jia, Z. J.; Huang, W.; Song, Y.; Kanter, J.; Scarborough, R. M. Preparation of pyrazolecarboxamides as inhibitors of factor Xa. US Patent US 6,632,815 B2, October 14, 2003.

[S26] Pettersson, L. Tricyclic compounds as 5-HT2 antagonists and their preparation, pharmaceutical compositions and use in the treatment of diseases. European Patent EP 3 753 924 A1, December 23, 2020.

[S27] Cheng, D.-J.; Yan, L.; Tian, S.-K.; Wu, M.-Y.; Wang, L.-X.; Fan, Z.-L.; Zheng, S.-C.; Liu, X.-Y.; Tan, B. Highly Enantioselective Kinetic Resolution of Axially Chiral BINAM Derivatives Catalyzed by a Brønsted Acid. *Angew. Chem., Int. Ed.* **2014**, *53*, 3684–3687. DOI: 10.1002/anie.201310562. *Angew. Chem.* **2014**, *126*, 3758–3761.

[S28] Uchikura, T.; Kanno, Y.; Fukuda, Y.; Sato, M.; Akiyama, T. Kinetic Resolution of 1,1′-Binaphthyl-2,2′-Diamine Derivatives by Chiral Calcium Phosphate-Catalyzed Acylation. *Org. Biomol. Chem.* **2024**, *22*, 3444–3447. DOI: 10.1039/D4OB00355A.

[S29] Van Es, J. J. G. S.; Biemans, H. A. M.; Meijer, E. W. Synthesis and Characterization of Optically Active Cyclic 6,6′-Dinitro-1,1′-Binaphthyl-2,2′-Diethers. *Tetrahedron: Asymmetry* **1997**, *8*, 1825–1831. DOI: 10.1016/S0957-4166(97)00185-7.

[S30] Li, T.; Pobanz, M. A.; Shih, C.; Wu, Z.; Yang, W. J.; Z., Boyu. Preparation of amidophenoxyindazoles as inhibitors of c-Met, US Patent US 8,030,302 B2, October 4, 2011.

11 HPLC traces

(R_a)-N^2-benzhydryl-[1,1'-binaphthalene]-2,2'-diamine [(R_a)-**4f**]

and (S_a)-N^2-benzhydryl-[1,1'-binaphthalene]-2,2'-diamine [(S_a)-**4f**]

Figure S1. *rac*-N^2-benzhydryl-[1,1'-binaphthalene]-2,2'-diamine (*rac*-**4f**)

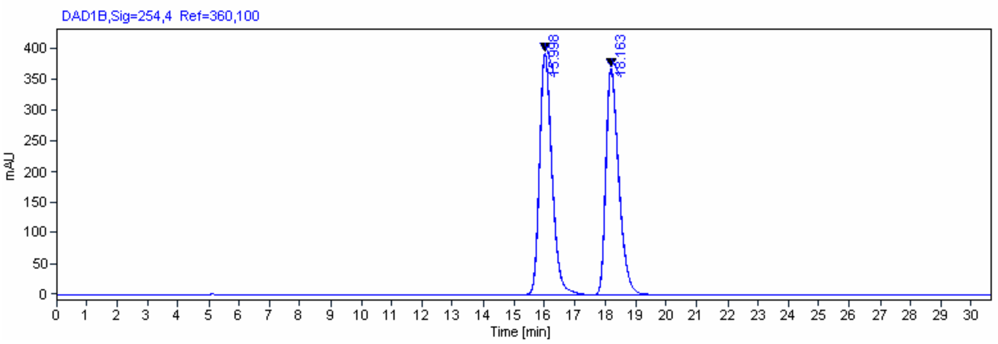

Signal: DAD1B,Sig=254,4 Ref=360,100

RT [min]	Width [min]	Area	Height	Area%
15.998	2.18	11084.39	392.43	50.69
18.163	2.54	10782.59	368.26	49.31

Figure S2. (R_a)-N^2-benzhydryl-[1,1'-binaphthalene]-2,2'-diamine [(R_a)-**4f**, 99% *ee*]

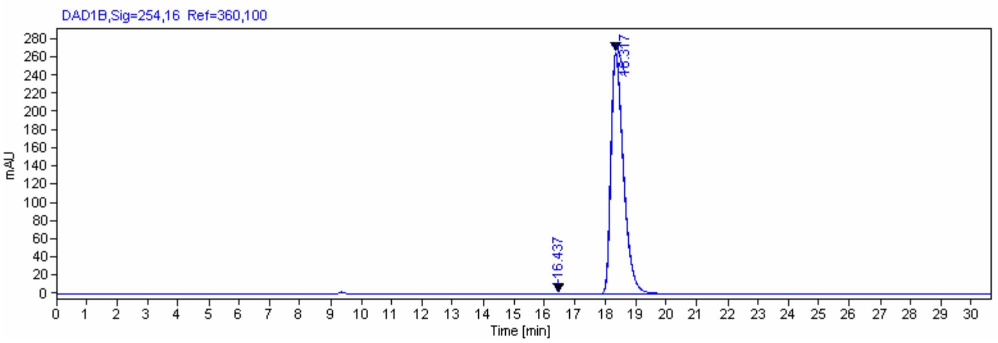

Signal: DAD1B,Sig=254,16 Ref=360,100

RT [min]	Width [min]	Area	Height	Area%
16.437	1.38	9.50	0.30	0.13
18.317	2.97	7576.57	265.05	99.87

Figure S3. (S_a)-N^2-benzhydryl-[1,1'-binaphthalene]-2,2'-diamine [(S_a)-**4f**, 99% *ee*]

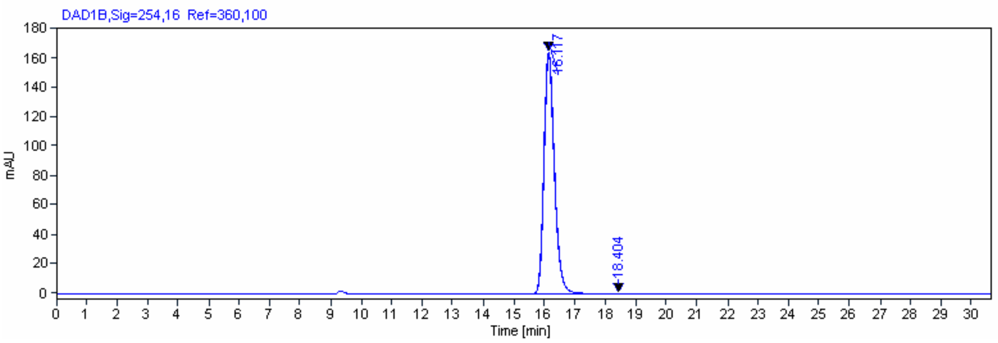

Signal: DAD1B,Sig=254,16 Ref=360,100

RT [min]	Width [min]	Area	Height	Area%
16.117	2.17	3880.26	164.10	99.81
18.404	1.17	7.21	0.29	0.19

Figure S4. (R_a)-N^2-benzhydryl-[1,1'-binaphthalene]-2,2'-diamine [(R_a)-**4f**, 50% *ee*]

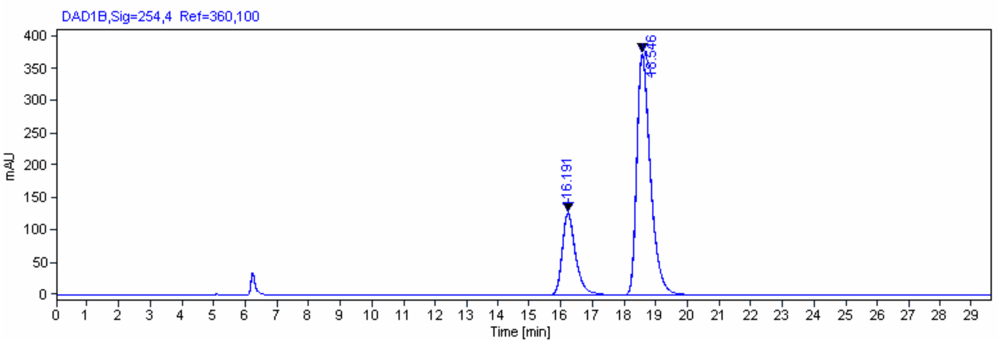

Signal: DAD1B,Sig=254,4 Ref=360,100

RT [min]	Width [min]	Area	Height	Area%
16.191	1.95	3704.02	126.69	24.95
18.546	2.35	11144.54	372.60	75.05

Figure S5. (S_a)-N^2-benzhydryl-[1,1'-binaphthalene]-2,2'-diamine [(S_a)-**4f**, 87% *ee*]

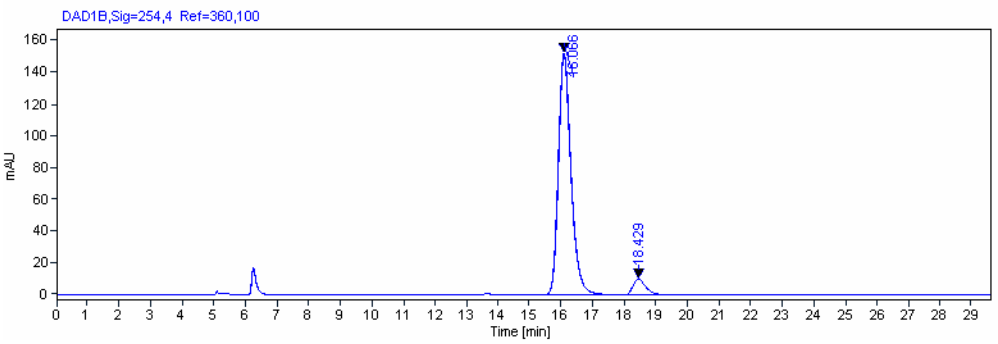

Signal: DAD1B,Sig=254,4 Ref=360,100

RT [min]	Width [min]	Area	Height	Area%
16.066	2.27	4206.98	152.16	93.69
18.429	1.69	283.43	9.94	6.31

[(R_a)-N^2-benzhydryl-6,6'-difluoro-[1,1'-binaphthalene]-2,2'-diamine [(R_a)-**4i**]

and (S_a)-N^2-benzhydryl-6,6'-difluoro-[1,1'-binaphthalene]-2,2'-diamine [(S_a)-**4i**]

Figure S6. *rac*-N^2-benzhydryl-6,6'-difluoro-[1,1'-binaphthalene]-2,2'-diamine (*rac*-**4i**)

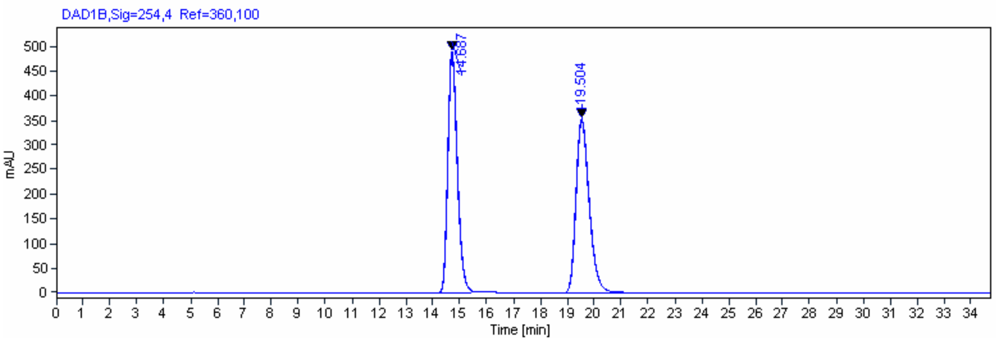

Signal:	DAD1B,Sig=254,4 Ref=360,100			
**RT [min]**	**Width [min]**	**Area**	**Height**	**Area%**
14.687	2.22	11958.16	489.62	50.04
19.504	2.52	11941.25	352.48	49.96

Figure S7. (R_a)-**4i** N^2-benzhydryl-6,6'-difluoro-[1,1'-binaphthalene]-2,2'-diamine [(R_a)-**4i**, 55% *ee*]

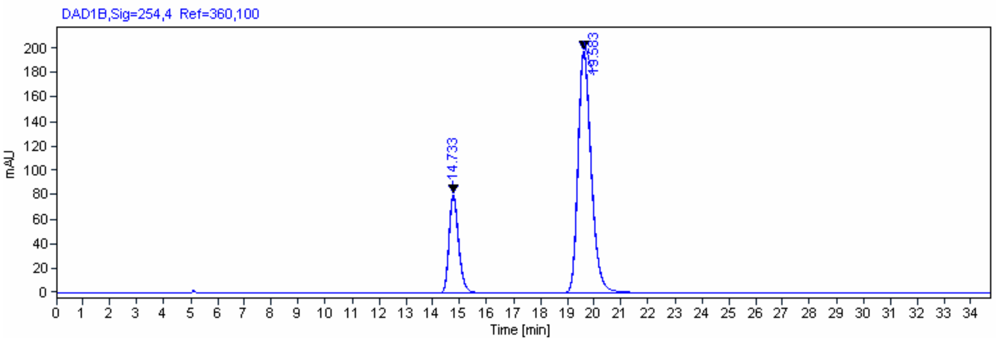

Signal:	DAD1B,Sig=254,4 Ref=360,100			
**RT [min]**	**Width [min]**	**Area**	**Height**	**Area%**
14.733	1.85	1957.63	80.13	22.56
19.583	3.01	6718.90	197.30	77.44

Figure S8. (S_a)-N^2-benzhydryl-6,6'-difluoro-[1,1'-binaphthalene]-2,2'-diamine [(S_a)-**4i**, 77% *ee*]

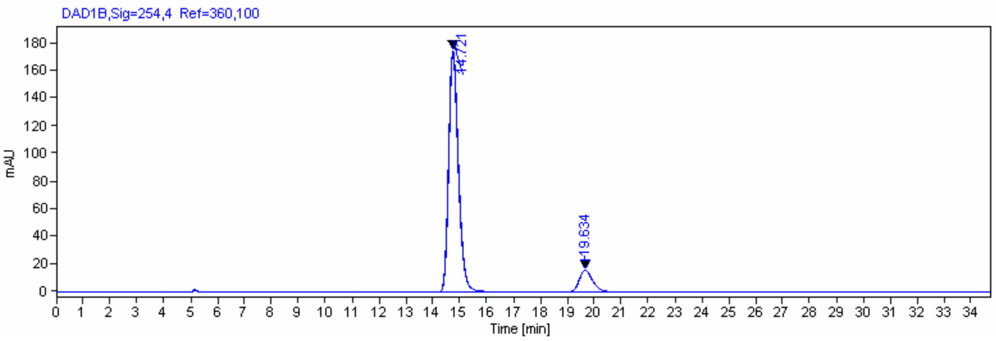

Signal:	DAD1B,Sig=254,4 Ref=360,100			
**RT [min]**	**Width [min]**	**Area**	**Height**	**Area%**
14.721	2.14	4383.05	174.65	88.87
19.634	1.95	549.15	15.88	11.13

(*R*a)-*N*2-benzhydryl-6,6’-dichloro-[1,1'-binaphthalene]-2,2'-diamine [(*R*a)-**4j**]

and (*S*a)-*N*2-benzhydryl-6,6‘-dichloro-[1,1'-binaphthalene]-2,2'-diamine [(*S*a)-**4j**]

Figure S9. *rac*-*N*2-benzhydryl-6,6‘-dichloro-[1,1'-binaphthalene]-2,2'-diamine (*rac*-**4j**)

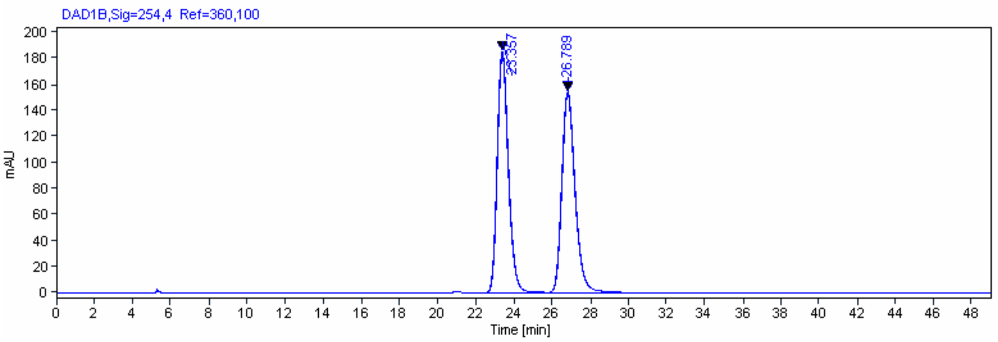

**Signal:**	DAD1B,Sig=254,4 Ref=360,100			
**RT [min]**	**Width [min]**	**Area**	**Height**	**Area%**
23.357	3.69	7486.85	185.10	50.13
26.789	5.38	7449.33	153.95	49.87

Figure S10. (*R*a)-*N*2-benzhydryl-6,6‘-dichloro-[1,1'-binaphthalene]-2,2'-diamine [(*R*a)-**4j**, 57% *ee*]

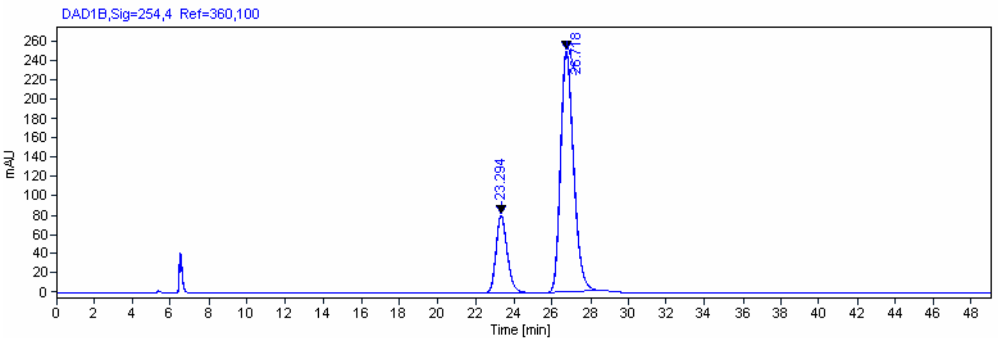

**Signal:**	DAD1B,Sig=254,4 Ref=360,100			
**RT [min]**	**Width [min]**	**Area**	**Height**	**Area%**
23.294	2.91	3260.91	80.18	21.63
26.718	2.86	11811.78	249.05	78.37

Figure S11. (*S*a)-*N*2-benzhydryl-6,6’-dichloro-[1,1'-binaphthalene]-2,2'-diamine [(*S*a)-**4j**, 84% *ee*]

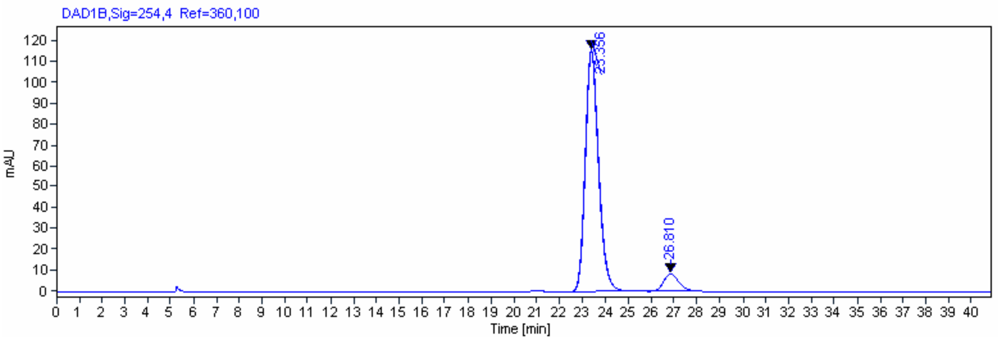

**Signal:**	DAD1B,Sig=254,4 Ref=360,100			
**RT [min]**	**Width [min]**	**Area**	**Height**	**Area%**
23.356	3.13	4636.71	115.17	92.28
26.810	2.39	387.95	8.33	7.72

(R_a)-N^2-benzhydryl-6,6'-dibromo-[1,1'-binaphthalene]-2,2'-diamine [(R_a)-**4k**]
and (S_a)-N^2-benzhydryl-6,6'-dibromo-[1,1'-binaphthalene]-2,2'-diamine [(S_a)-**4k**]

Figure S12. *rac*-N^2-benzhydryl-6,6'-dibromo-[1,1'-binaphthalene]-2,2'-diamine (*rac*-**4k**)

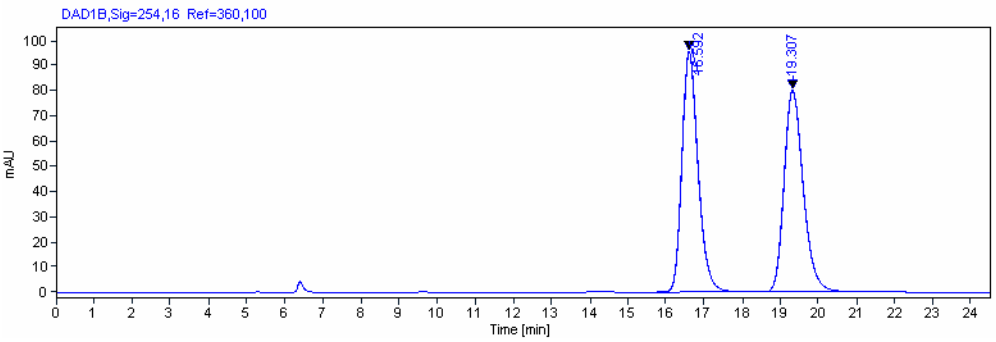

Signal:	DAD1B,Sig=254,16 Ref=360,100			
**RT [min]**	**Width [min]**	**Area**	**Height**	**Area%**
16.592	2.43	2807.05	95.62	50.13
19.307	2.48	2792.47	80.17	49.87

Figure S13. (R_a)-N^2-benzhydryl-6,6'-dibromo-[1,1'-binaphthalene]-2,2'-diamine [(R_a)-**4k**, 85% *ee*]

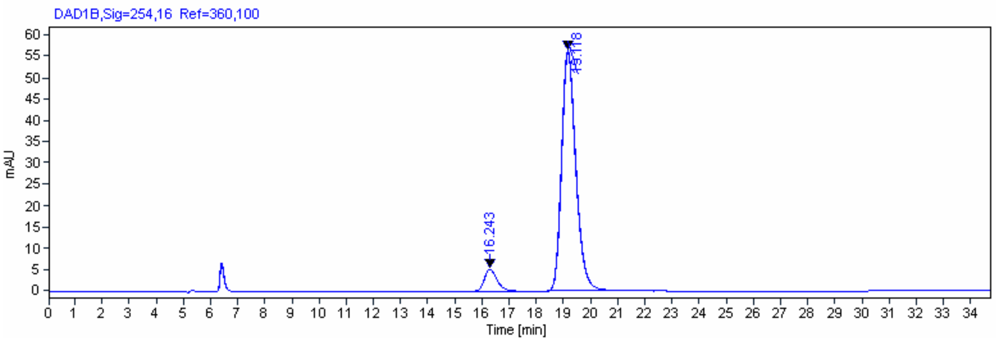

Signal:	DAD1B,Sig=254,16 Ref=360,100			
**RT [min]**	**Width [min]**	**Area**	**Height**	**Area%**
16.243	1.93	166.43	5.15	7.57
19.118	2.37	2030.89	56.02	92.43

Figure S14. (S_a)-N^2-benzhydryl-6,6'-dibromo-[1,1'-binaphthalene]-2,2'-diamine [(S_a)-**4k**, 77% *ee*]

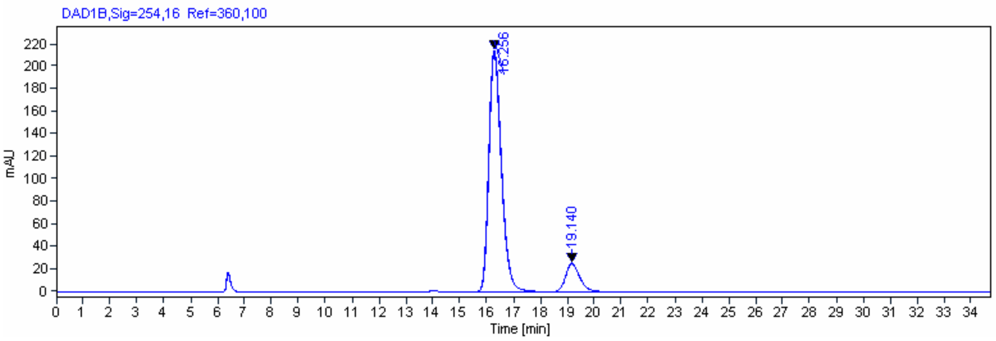

Signal:	DAD1B,Sig=254,16 Ref=360,100			
**RT [min]**	**Width [min]**	**Area**	**Height**	**Area%**
16.256	2.88	6923.85	214.42	88.41
19.140	2.06	907.62	25.02	11.59

(R_a)-N^2-benzhydryl-6,6'-dimethyl-[1,1'-binaphthalene]-2,2'-diamine [(R_a)-**4l**]

and (S_a)-N^2-benzhydryl-6,6'-dimethyl-[1,1'-binaphthalene]-2,2'-diamine [(S_a)-**4l**]

Figure S15. *rac*-N^2-benzhydryl-6,6'-dimethyl-[1,1'-binaphthalene]-2,2'-diamine (*rac*-**4l**)

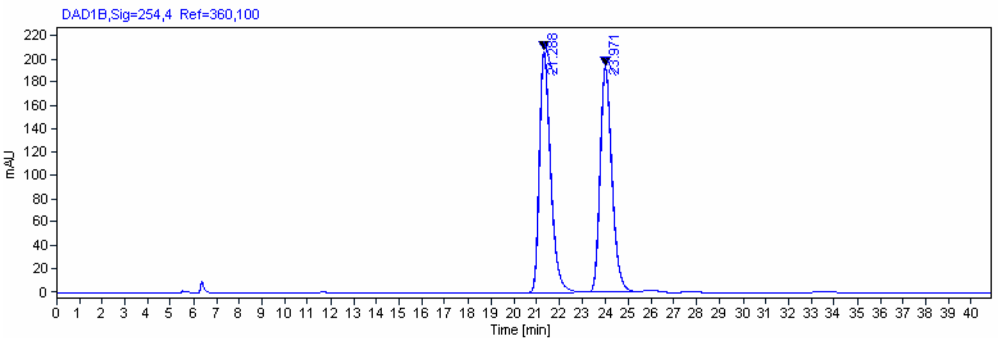

**Signal:**	DAD1B,Sig=254,4 Ref=360,100			
**RT [min]**	**Width [min]**	**Area**	**Height**	**Area%**
21.288	2.64	7021.96	206.54	50.43
23.971	2.29	6902.15	192.62	49.57

Figure S16. (R_a)-N^2-benzhydryl-6,6'-dimethyl-[1,1'-binaphthalene]-2,2'-diamine [(R_a)-**4l**, 47% *ee*]

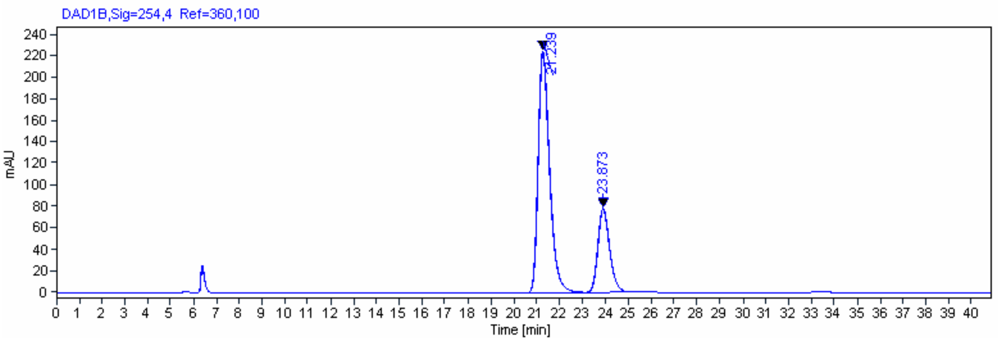

**Signal:**	DAD1B,Sig=254,4 Ref=360,100			
**RT [min]**	**Width [min]**	**Area**	**Height**	**Area%**
21.239	2.61	7768.73	224.72	73.63
23.873	2.10	2782.07	77.99	26.37

Figure S17. (S_a)-N^2-benzhydryl-6,6'-dimethyl-[1,1'-binaphthalene]-2,2'-diamine [(S_a)-**4l**, 83% *ee*]

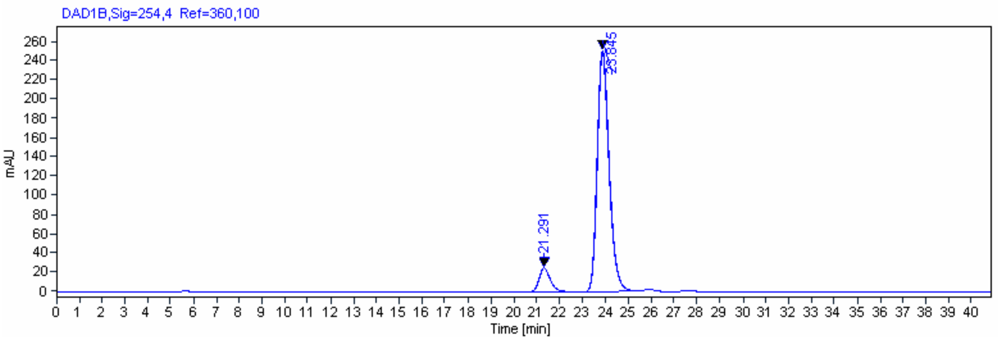

**Signal:**	DAD1B,Sig=254,4 Ref=360,100			
**RT [min]**	**Width [min]**	**Area**	**Height**	**Area%**
21.291	2.05	839.99	24.82	8.53
23.845	2.35	9003.16	250.32	91.47

Figure S18. (*R*a)-*N*[2]-benzhydryl-6,6'-dimethyl-[1,1'-binaphthalene]-2,2'-diamine [(*R*a)-**4l**, 57% *ee*]

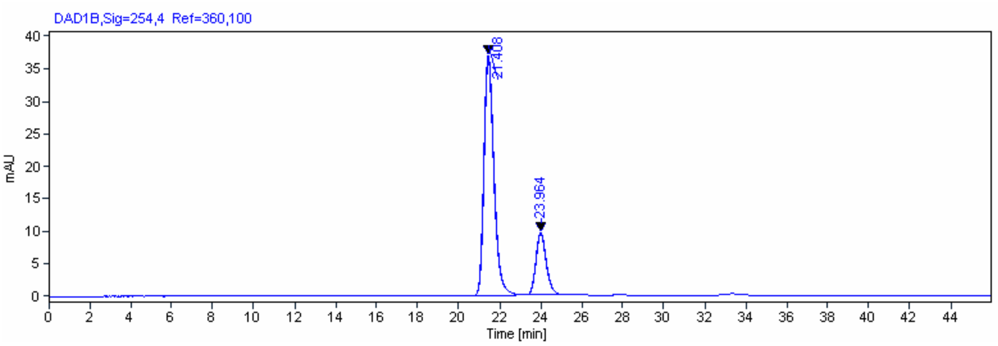

Signal:	DAD1B,Sig=254,4 Ref=360,100			
**RT [min]**	**Width [min]**	**Area**	**Height**	**Area%**
21.408	2.51	1241.95	36.99	78.57
23.964	1.99	338.66	9.61	21.43

Figure S19. (*S*a)-*N*[2]-benzhydryl-6,6'-dimethyl-[1,1'-binaphthalene]-2,2'-diamine [(*S*a)-**4l**, 88% *ee*]

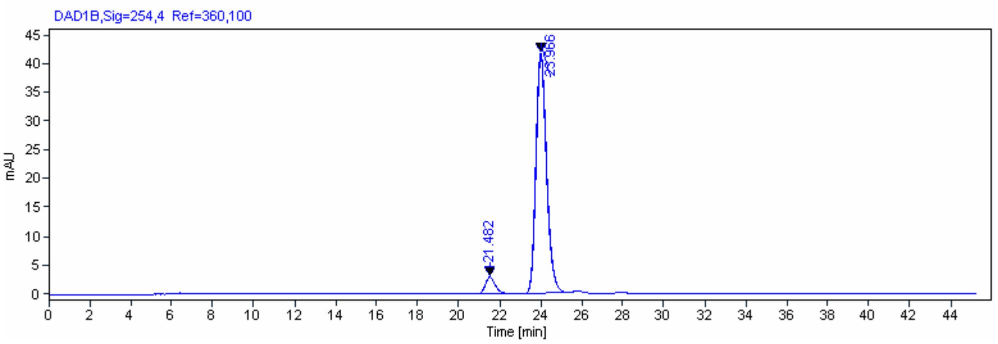

Signal:	DAD1B,Sig=254,4 Ref=360,100			
**RT [min]**	**Width [min]**	**Area**	**Height**	**Area%**
21.482	1.73	95.15	2.86	6.00
23.966	2.20	1490.11	41.67	94.00

(R_a)-N^2-benzhydryl-6,6’-diisopropyl-[1,1'-binaphthalene]-2,2'-diamine [(R_a)-**4m**]

and (S_a)-N^2-benzhydryl-6,6‘-diisopropyl-[1,1'-binaphthalene]-2,2'-diamine [(S_a)-**4m**]

Figure S20. *rac*-N^2-benzhydryl-6,6‘-diisopropyl-[1,1'-binaphthalene]-2,2'-diamine (*rac*-**4m**)

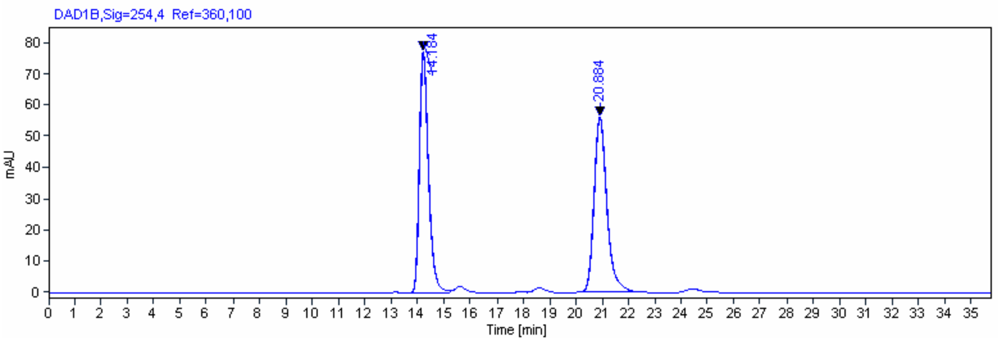

Signal:	DAD1B,Sig=254,4 Ref=360,100			
**RT [min]**	**Width [min]**	**Area**	**Height**	**Area%**
14.184	1.59	1846.92	77.20	49.30
20.884	2.42	1899.29	56.22	50.70

Figure S21. (R_a)-N^2-benzhydryl-6,6‘-diisopropyl-[1,1'-binaphthalene]-2,2'-diamine [(R_a)-**4m**, 32% *ee*]

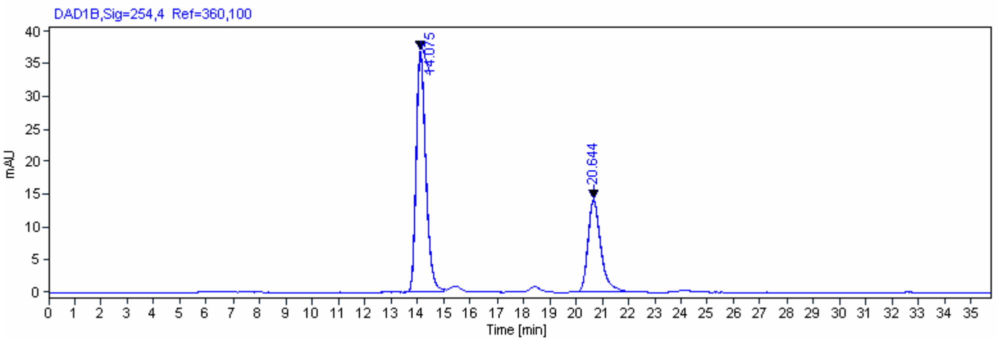

Signal:	DAD1B,Sig=254,4 Ref=360,100			
**RT [min]**	**Width [min]**	**Area**	**Height**	**Area%**
14.075	1.55	943.42	37.00	66.05
20.644	2.63	485.02	14.18	33.95

Figure S22. (S_a)-N^2-benzhydryl-6,6’-diisopropyl-[1,1'-binaphthalene]-2,2'-diamine [(S_a)-**4m**, 76% *ee*]

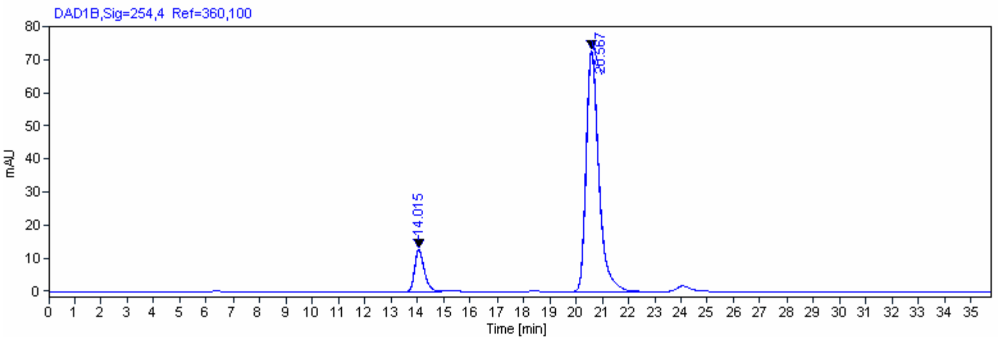

Signal:	DAD1B,Sig=254,4 Ref=360,100			
**RT [min]**	**Width [min]**	**Area**	**Height**	**Area%**
14.015	1.34	327.57	12.78	11.86
20.567	2.93	2435.35	72.82	88.14

(R_a)-N^2-benzhydryl-6,6'-diphenyl-[1,1'-binaphthalene]-2,2'-diamine [(R_a)-**4n**]

and (S_a)-N^2-benzhydryl-6,6'-diphenyl-[1,1'-binaphthalene]-2,2'-diamine [(S_a)-**4n**]

Figure S23. *rac*-N^2-benzhydryl-6,6'-diphenyl-[1,1'-binaphthalene]-2,2'-diamine (*rac*-**4n**)

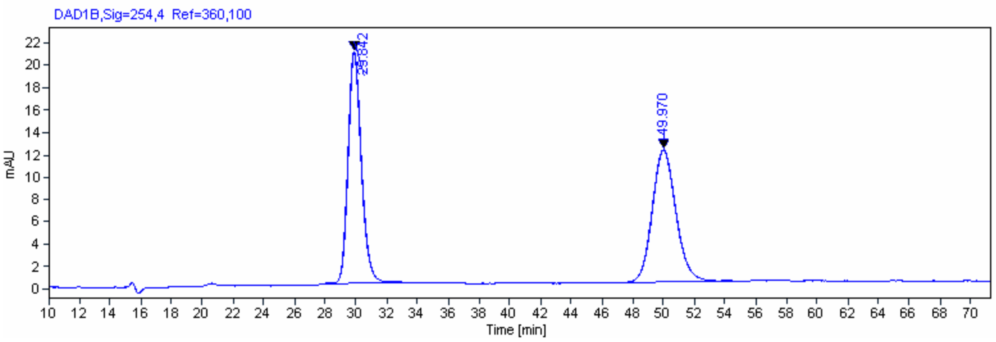

Signal:	DAD1B,Sig=254,4 Ref=360,100			
**RT [min]**	**Width [min]**	**Area**	**Height**	**Area%**
29.842	5.56	1245.73	20.64	49.71
49.970	7.85	1260.41	11.78	50.29

Figure S24. (R_a)-N^2-benzhydryl-6,6'-diphenyl-[1,1'-binaphthalene]-2,2'-diamine [(R_a)-**4n**, 30% *ee*]

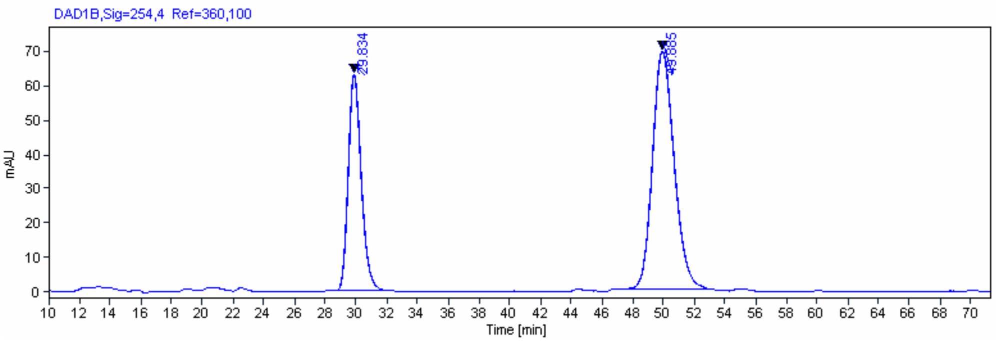

Signal:	DAD1B,Sig=254,4 Ref=360,100			
**RT [min]**	**Width [min]**	**Area**	**Height**	**Area%**
29.834	4.13	3705.01	63.30	35.14
49.885	6.02	6839.27	69.56	64.86

Figure S25. (S_a)-N^2-benzhydryl-6,6'-diphenyl-[1,1'-binaphthalene]-2,2'-diamine [(S_a)-**4n**, 89% *ee*]

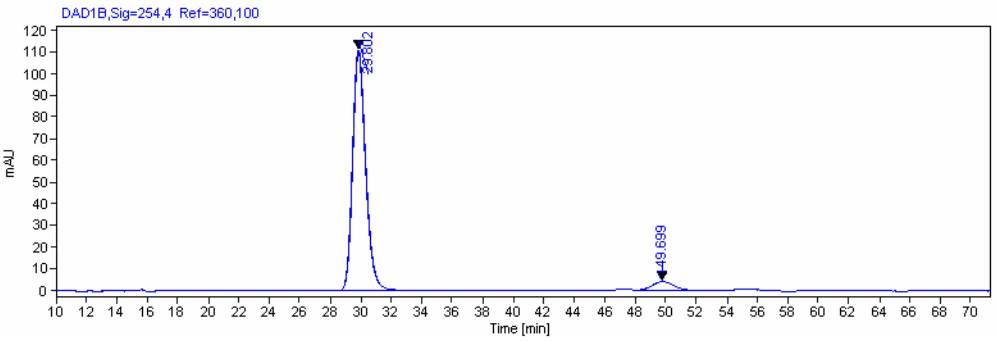

Signal:	DAD1B,Sig=254,4 Ref=360,100			
**RT [min]**	**Width [min]**	**Area**	**Height**	**Area%**
29.802	5.10	6564.49	110.80	94.53
49.699	4.25	380.18	3.91	5.47

(*R*a)-*N*2-benzhydryl-6,6’-bis(trimethylsilyl)-[1,1'-binaphthalene]-2,2'-diamine [(*R*a)-**4o**] and (*S*a)-*N*2-benzhydryl-6,6‘-bis(trimethylsilyl)-[1,1'-binaphthalene]-2,2'-diamine [(*S*a)-**4o**]

Figure S26. *rac*-*N*2-benzhydryl-6,6‘-bis(trimethylsilyl)-[1,1'-binaphthalene]-2,2'-diamine (*rac*-**4o**)

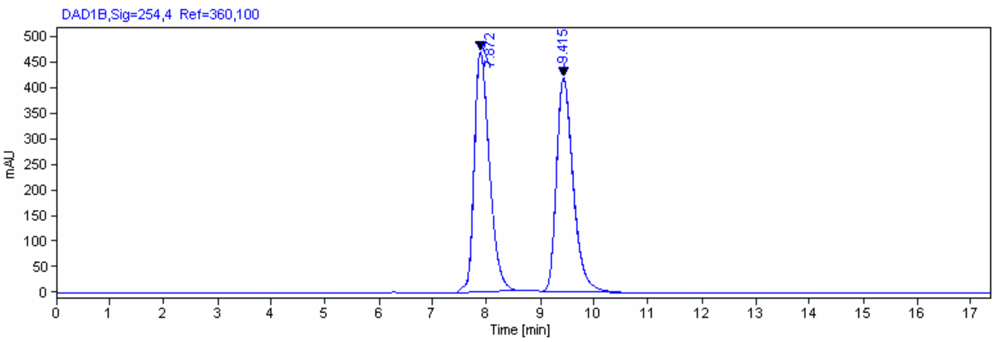

**Signal:**	DAD1B,Sig=254,4 Ref=360,100			
**RT [min]**	**Width [min]**	**Area**	**Height**	**Area%**
7.872	1.21	9159.01	468.18	50.58
9.415	1.74	8948.45	416.76	49.42

Figure S27. (*R*a)-*N*2-benzhydryl-6,6‘-bis(trimethylsilyl)-[1,1'-binaphthalene]-2,2'-diamine [(*R*a)-**4o**, 72% *ee*]

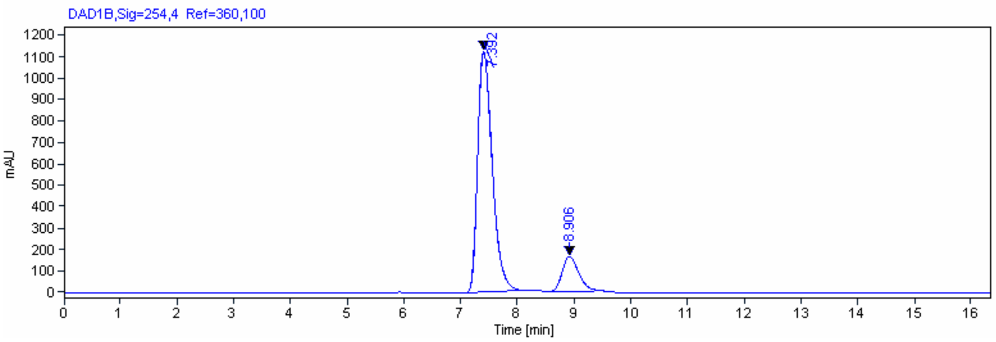

**Signal:**	DAD1B,Sig=254,4 Ref=360,100			
**RT [min]**	**Width [min]**	**Area**	**Height**	**Area%**
7.392	1.04	20121.89	1124.12	85.99
8.906	0.99	3278.13	161.64	14.01

Figure S28. (*S*a)-*N*2-benzhydryl-6,6’-bis(trimethylsilyl)-[1,1'-binaphthalene]-2,2'-diamine [(*S*a)-**4o**, 76% *ee*]

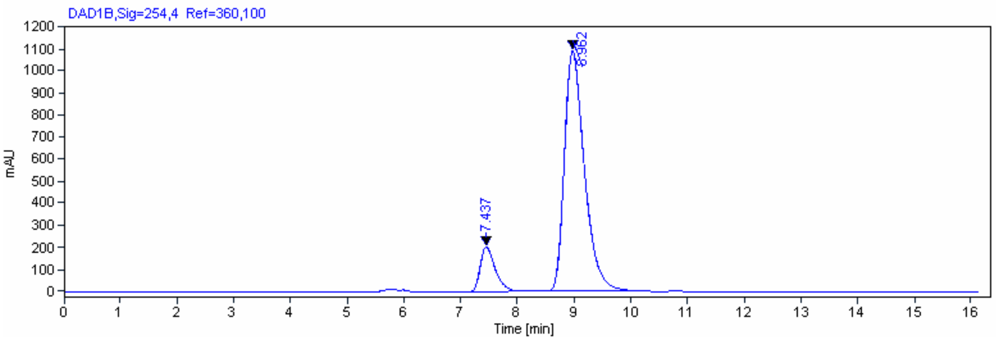

**Signal:**	DAD1B,Sig=254,4 Ref=360,100			
**RT [min]**	**Width [min]**	**Area**	**Height**	**Area%**
7.437	0.96	3692.67	202.61	12.25
8.962	1.51	26447.90	1086.32	87.75

(R_a)-N^2-benzhydryl-6,6'-dimethoxy-[1,1'-binaphthalene]-2,2'-diamine [(R_a)-**4p**]
and (S_a)-N^2-benzhydryl-6,6'-dimethoxy-[1,1'-binaphthalene]-2,2'-diamine [(S_a)-**4p**]

Figure S29. *rac*-N^2-benzhydryl-6,6'-dimethoxy-[1,1'-binaphthalene]-2,2'-diamine (*rac*-**4p**)

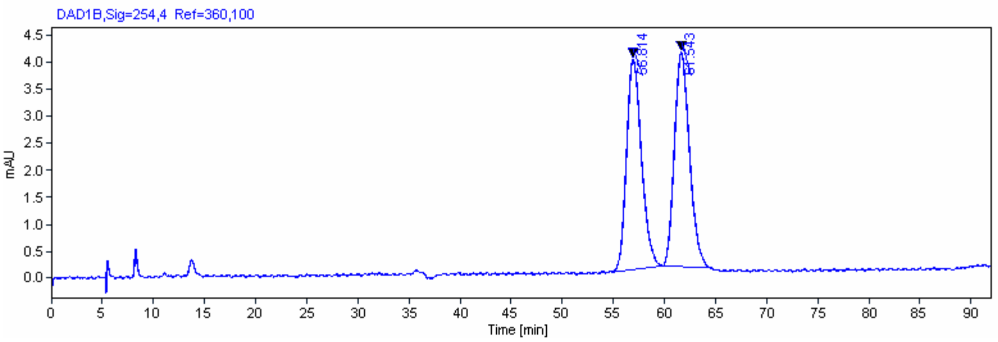

Signal:	DAD1B,Sig=254,4 Ref=360,100			
**RT [min]**	**Width [min]**	**Area**	**Height**	**Area%**
56.814	4.90	397.79	3.91	49.37
61.543	4.82	407.94	3.99	50.63

Figure S30. (R_a)-N^2-benzhydryl-6,6'-dimethoxy-[1,1'-binaphthalene]-2,2'-diamine [(R_a)-**4p**, 74% *ee*]

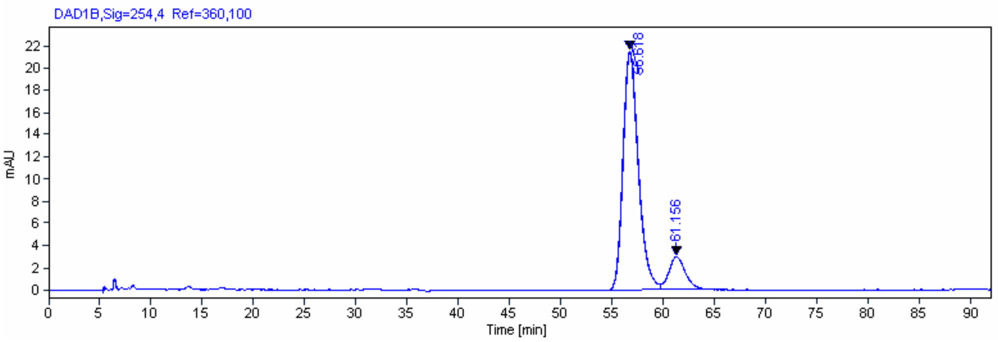

Signal:	DAD1B,Sig=254,4 Ref=360,100			
**RT [min]**	**Width [min]**	**Area**	**Height**	**Area%**
56.618	5.30	2232.48	21.53	87.10
61.156	6.31	330.50	2.96	12.90

Figure S31. (S_a)-N^2-benzhydryl-6,6'-dimethoxy-[1,1'-binaphthalene]-2,2'-diamine [(S_a)-**4p**, 74% *ee*]

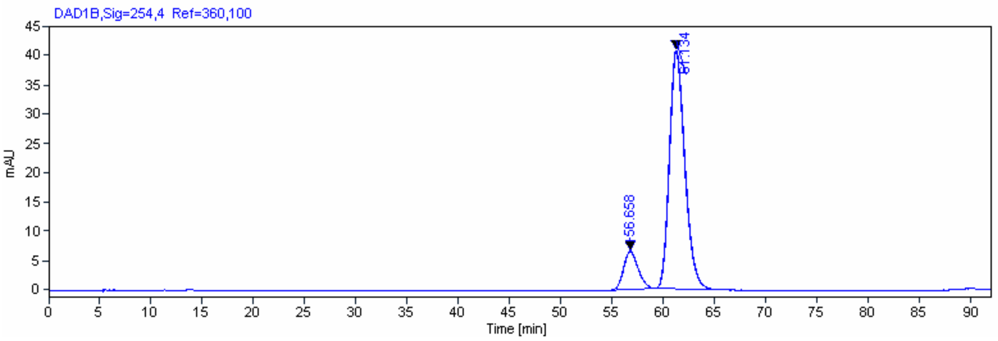

Signal:	DAD1B,Sig=254,4 Ref=360,100			
**RT [min]**	**Width [min]**	**Area**	**Height**	**Area%**
56.658	4.56	640.91	6.48	13.11
61.134	8.10	4247.84	40.63	86.89

(R_a)-N^2-benzhydryl-7,7'-dibromo-[1,1'-binaphthalene]-2,2'-diamine [(R_a)-**4q**]

and (S_a)-N^2-benzhydryl-7,7'-dibromo-[1,1'-binaphthalene]-2,2'-diamine [(S_a)-**4q**]

Figure S32. *rac*-N^2-benzhydryl-7,7'-dibromo-[1,1'-binaphthalene]-2,2'-diamine (*rac*-**4q**)

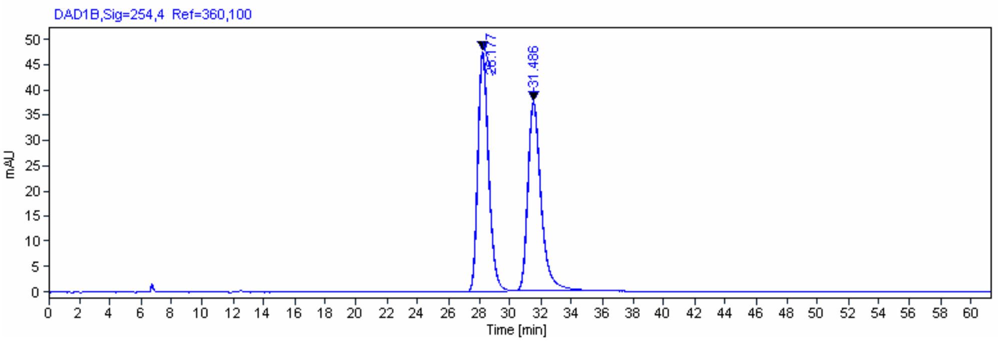

Signal:	DAD1B,Sig=254,4 Ref=360,100			
**RT [min]**	**Width [min]**	**Area**	**Height**	**Area%**
28.177	3.34	2315.82	47.67	50.62
31.486	6.87	2259.50	37.52	49.38

Figure S33. (R_a)-N^2-benzhydryl-7,7'-dibromo-[1,1'-binaphthalene]-2,2'-diamine [(R_a)-**4q**, 41% *ee*]

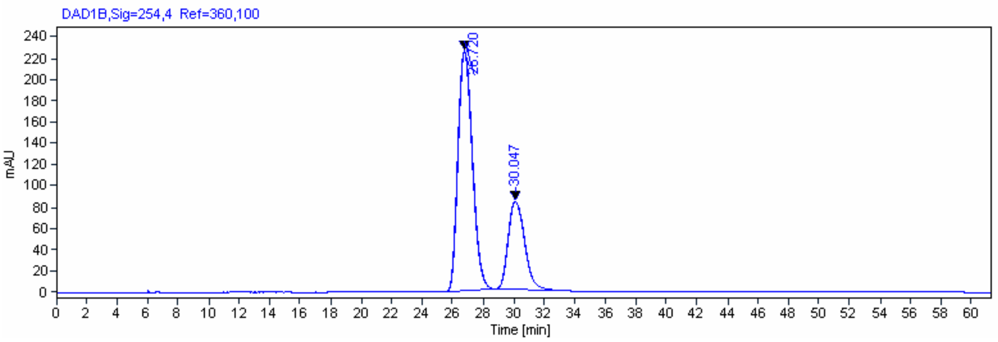

Signal:	DAD1B,Sig=254,4 Ref=360,100			
**RT [min]**	**Width [min]**	**Area**	**Height**	**Area%**
26.720	3.33	14794.29	224.71	70.43
30.047	5.56	6210.58	82.64	29.57

Figure S34. (S_a)-N^2-benzhydryl-7,7'-dibromo-[1,1'-binaphthalene]-2,2'-diamine [(S_a)-**4q**, 76% *ee*]

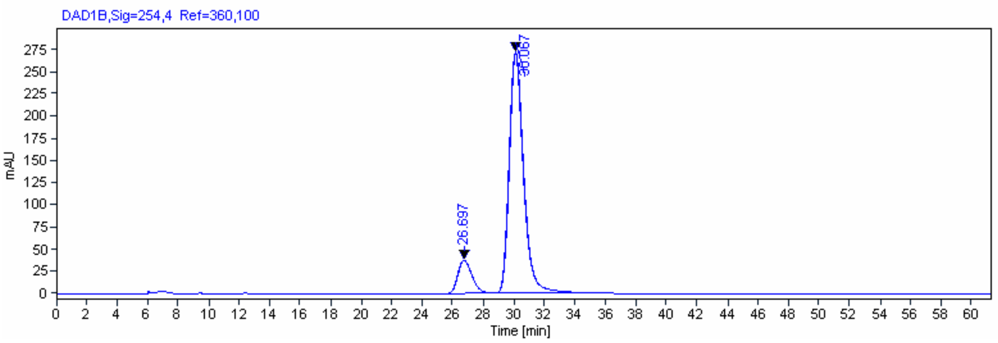

Signal:	DAD1B,Sig=254,4 Ref=360,100			
**RT [min]**	**Width [min]**	**Area**	**Height**	**Area%**
26.697	3.18	2389.77	37.25	11.92
30.067	6.21	17658.28	270.98	88.08

(R_a)-N^2-benzhydryl-7,7'-dimethyl-[1,1'-binaphthalene]-2,2'-diamine [(R_a)-**4r**] and (S_a)-N^2-benzhydryl-7,7'-dimethyl-[1,1'-binaphthalene]-2,2'-diamine [(S_a)-**4r**]

Figure S35. *rac*-N^2-benzhydryl-7,7'-dimethyl-[1,1'-binaphthalene]-2,2'-diamine (*rac*-**4r**)

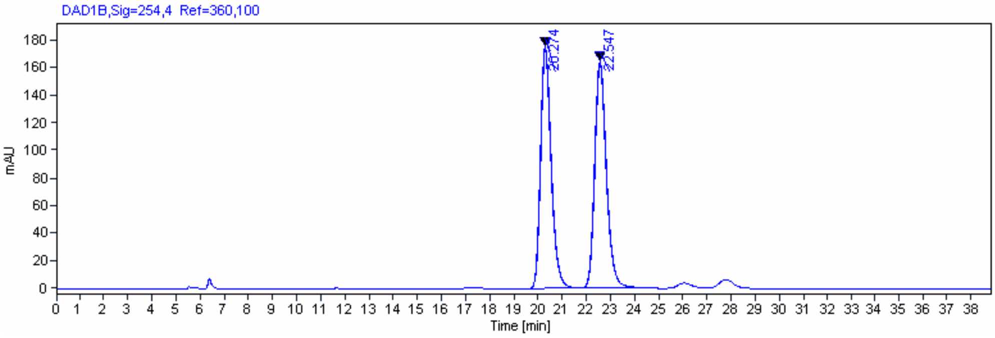

Signal: DAD1B,Sig=254,4 Ref=360,100

RT [min]	Width [min]	Area	Height	Area%
20.274	2.19	5354.33	174.86	49.43
22.547	2.28	5477.31	163.74	50.57

Figure S36. (R_a)-N^2-benzhydryl-7,7'-dimethyl-[1,1'-binaphthalene]-2,2'-diamine [(R_a)-**4r**, 23% *ee*]

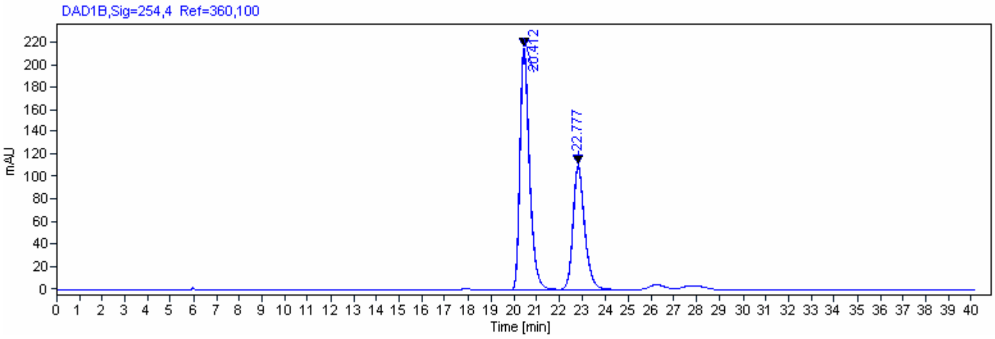

Signal: DAD1B,Sig=254,4 Ref=360,100

RT [min]	Width [min]	Area	Height	Area%
20.412	2.17	6221.32	214.94	61.69
22.777	2.53	3864.24	110.35	38.31

Figure S37. (S_a)-N^2-benzhydryl-7,7'-dimethyl-[1,1'-binaphthalene]-2,2'-diamine [(S_a)-**4r**, 80% *ee*]

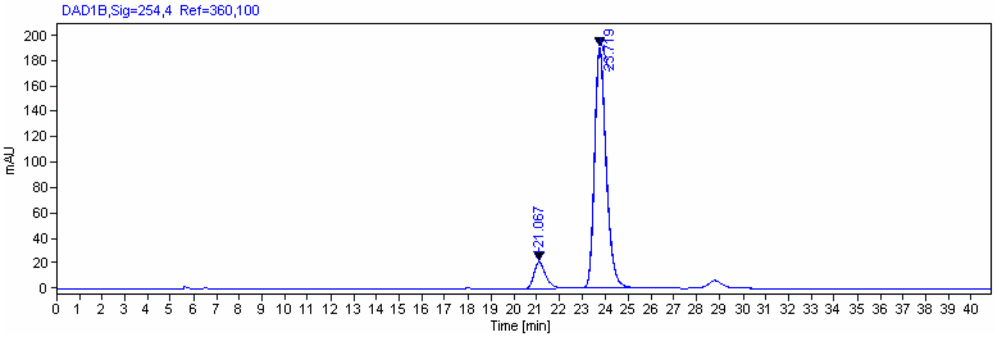

Signal: DAD1B,Sig=254,4 Ref=360,100

RT [min]	Width [min]	Area	Height	Area%
21.067	2.10	746.14	20.93	10.00
23.719	2.90	6713.88	190.13	90.00

(R_a)-N^2-benzhydryl-7,7'-diphenyl-[1,1'-binaphthalene]-2,2'-diamine [(R_a)-**4s**]

and (S_a)-N^2-benzhydryl-7,7'-diphenyl-[1,1'-binaphthalene]-2,2'-diamine [(S_a)-**4s**]

Figure S38. *rac*-N^2-benzhydryl-7,7'-diphenyl-[1,1'-binaphthalene]-2,2'-diamine (*rac*-**4s**)

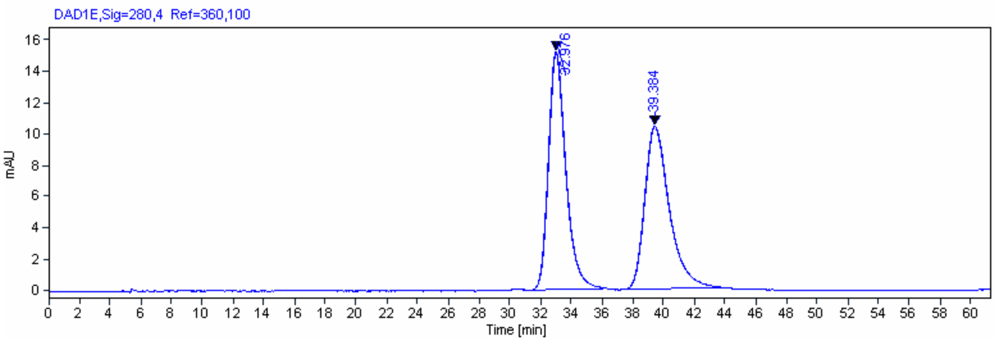

**Signal:**	DAD1B,Sig=280,4 Ref=360,100			
**RT [min]**	**Width [min]**	**Area**	**Height**	**Area%**
32.976	5.07	1189.95	15.20	50.89
39.384	6.15	1148.16	10.40	49.11

Figure S39. (R_a)-N^2-benzhydryl-7,7'-diphenyl-[1,1'-binaphthalene]-2,2'-diamine [(R_a)-**4s**, 18% *ee*]

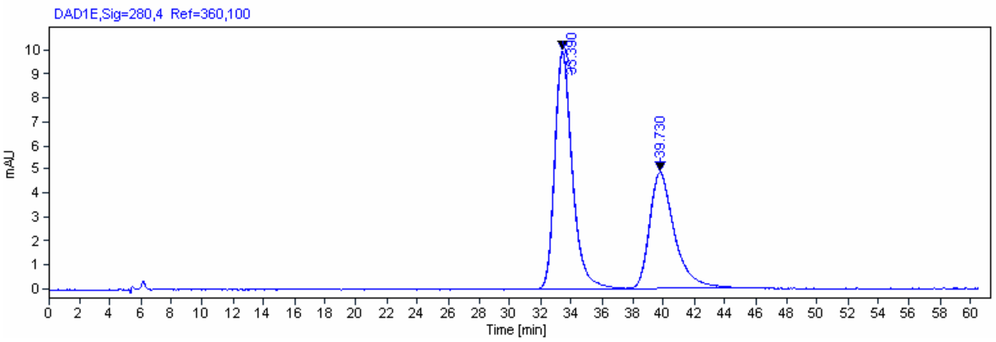

**Signal:**	DAD1B,Sig=280,4 Ref=360,100			
**RT [min]**	**Width [min]**	**Area**	**Height**	**Area%**
33.390	5.96	783.60	9.90	59.17
39.730	7.52	540.71	4.84	40.83

Figure S40. (S_a)-N^2-benzhydryl-7,7'-diphenyl-[1,1'-binaphthalene]-2,2'-diamine [(S_a)-**4s**, 73% *ee*]

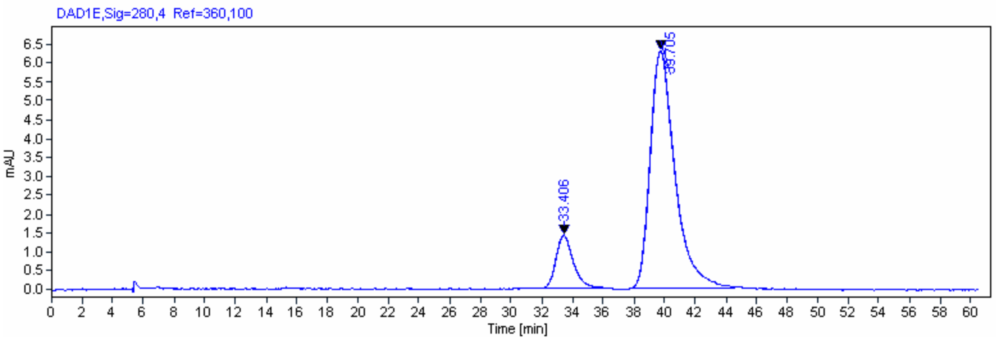

**Signal:**	DAD1B,Sig=280,4 Ref=360,100			
**RT [min]**	**Width [min]**	**Area**	**Height**	**Area%**
33.406	4.01	107.45	1.39	13.24
39.705	7.33	704.26	6.29	86.76

(R_a)-N^2-benzhydryl-7,7'-dimethoxy-[1,1'-binaphthalene]-2,2'-diamine [(R_a)-**4t**]

and (S_a)-N^2-benzhydryl-7,7'-dimethoxy-[1,1'-binaphthalene]-2,2'-diamine [(S_a)-**4t**]

Figure S41. *rac*-N^2-benzhydryl-7,7'-dimethoxy-[1,1'-binaphthalene]-2,2'-diamine (*rac*-**4t**)

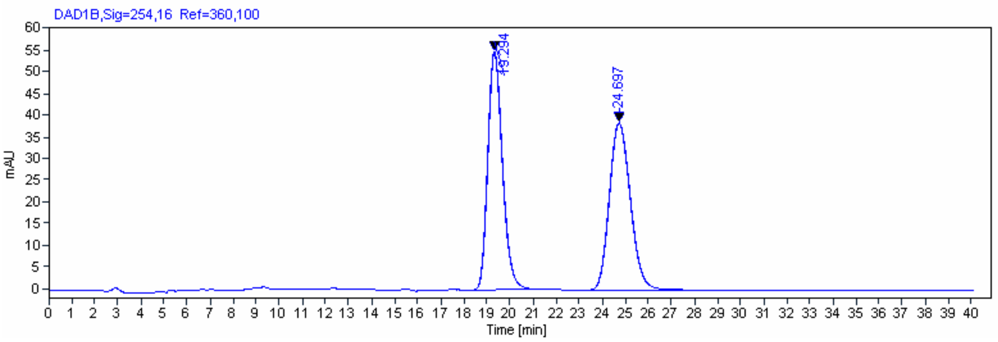

Signal:	DAD1B,Sig=254,16 Ref=360,100			
**RT [min]**	**Width [min]**	**Area**	**Height**	**Area%**
19.294	3.79	2417.75	54.73	49.96
24.697	4.27	2421.43	38.38	50.04

Figure S42. (R_a)-N^2-benzhydryl-7,7'-dimethoxy-[1,1'-binaphthalene]-2,2'-diamine [(R_a)-**4t**, 19% *ee*]

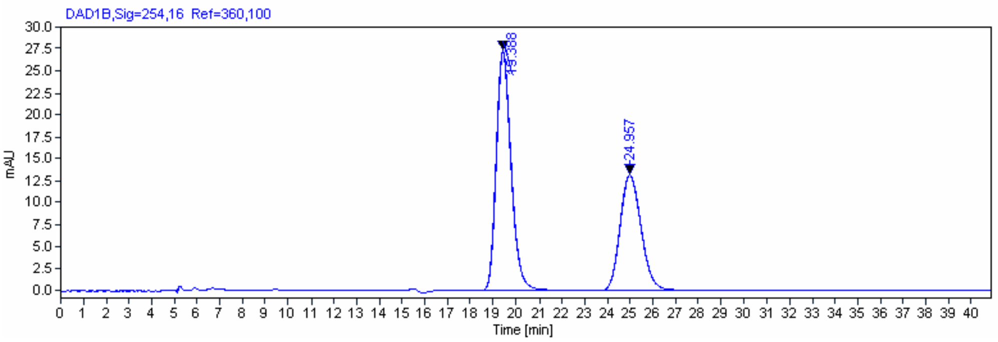

Signal:	DAD1B,Sig=254,16 Ref=360,100			
**RT [min]**	**Width [min]**	**Area**	**Height**	**Area%**
19.388	3.34	1227.87	27.22	59.45
24.957	3.79	837.66	13.12	40.55

Figure S43. (S_a)-N^2-benzhydryl-7,7'-dimethoxy-[1,1'-binaphthalene]-2,2'-diamine [(S_a)-**4t**, 88% *ee*]

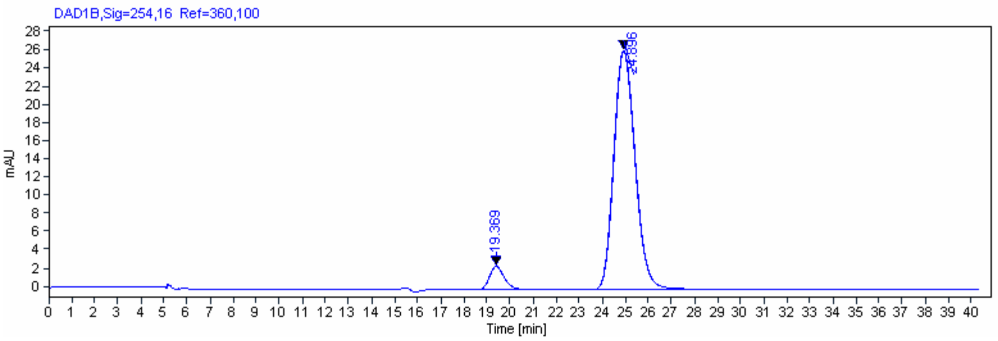

Signal:	DAD1B,Sig=254,16 Ref=360,100			
**RT [min]**	**Width [min]**	**Area**	**Height**	**Area%**
19.369	2.34	111.82	2.54	6.25
24.896	4.60	1676.28	26.25	93.75

(R_a)-N^2-benzhydryl-4,4‘-dimethyl-[1,1'-binaphthalene]-2,2'-diamine [(R_a)-**4u**]
and (S_a)-N^2-benzhydryl-4,4‘-dimethyl-[1,1'-binaphthalene]-2,2'-diamine [(S_a)-**4u**]

Figure S44. *rac*-N^2-benzhydryl-4,4‘-dimethyl-[1,1'-binaphthalene]-2,2'-diamine (*rac*-**4u**)

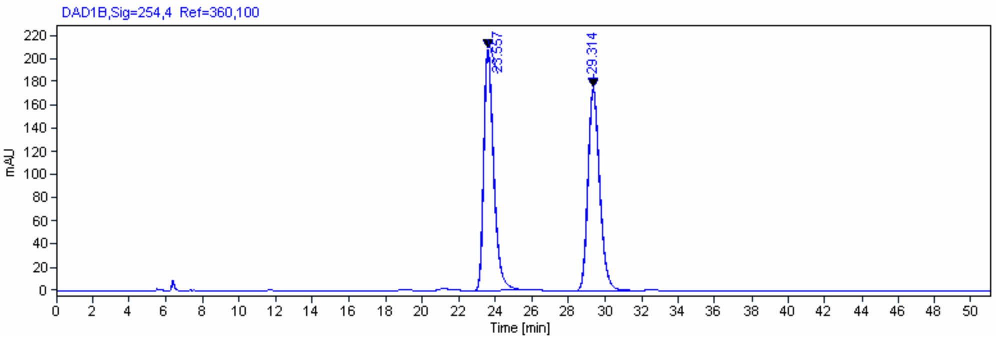

Signal: DAD1B,Sig=254,4 Ref=360,100

RT [min]	Width [min]	Area	Height	Area%
23.557	3.01	7870.05	208.09	50.27
29.314	3.67	7787.06	174.55	49.73

Figure S45. (R_a)-N^2-benzhydryl-4,4‘-dimethyl-[1,1'-binaphthalene]-2,2'-diamine [(R_a)-**4u**, 15% *ee*]

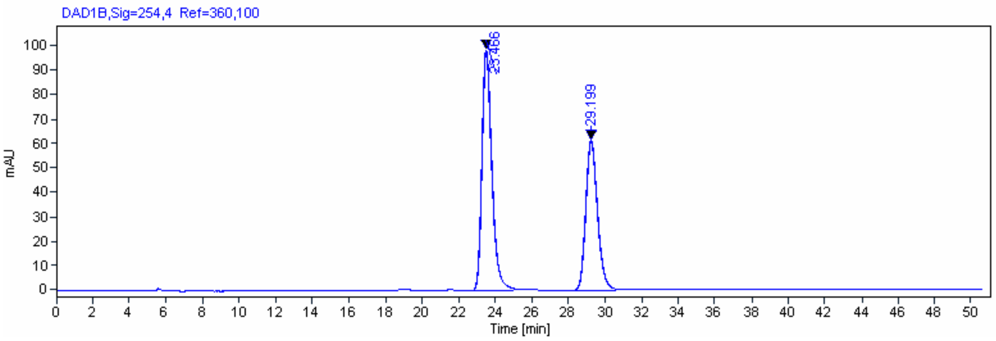

Signal: DAD1B,Sig=254,4 Ref=360,100

RT [min]	Width [min]	Area	Height	Area%
23.466	3.55	3683.04	98.05	57.59
29.199	3.07	2712.74	61.11	42.41

Figure S46. (S_a)-N^2-benzhydryl-4,4‘-dimethyl-[1,1'-binaphthalene]-2,2'-diamine [(S_a)-**4u**, 55% *ee*]

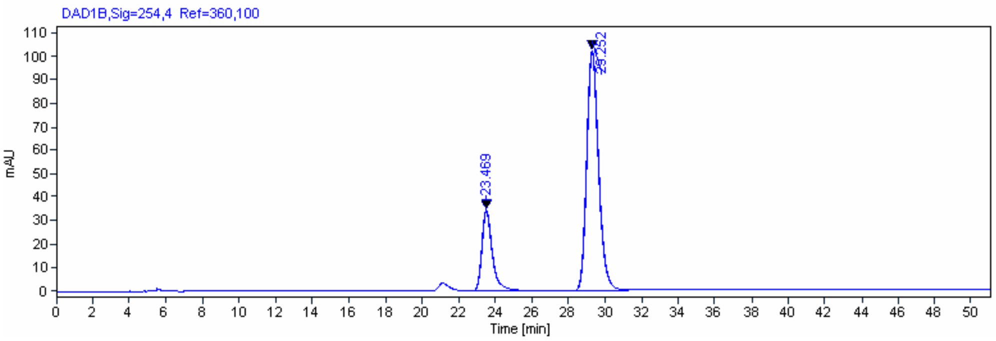

Signal: DAD1B,Sig=254,4 Ref=360,100

RT [min]	Width [min]	Area	Height	Area%
23.469	3.69	1344.42	34.33	22.64
29.252	4.15	4592.80	102.36	77.36

(R_a)-[1,1'-binaphthalene]-2,2'-diamine [(R_a)-**4a**] (Cleavage of Benzhydryl Group)

Figure S48. (R_a)-N^2-benzhydryl-[1,1'-binaphthalene]-2,2'-diamine [(R_a)-**4f**, 30% *ee*]

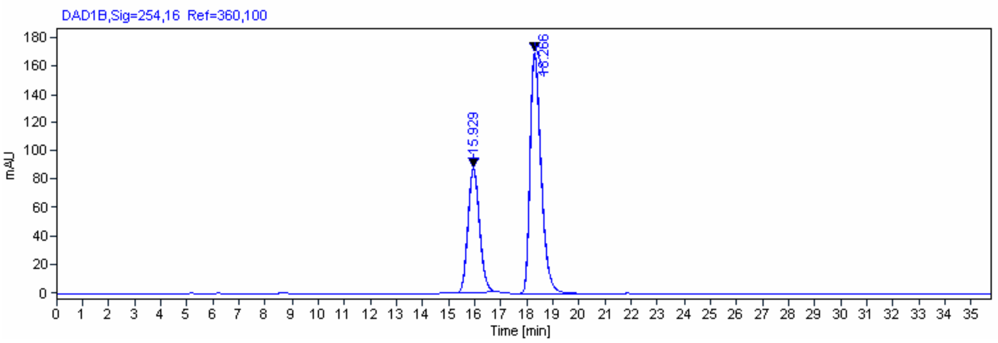

Signal:	DAD1B,Sig=254,16 Ref=360,100			
**RT [min]**	**Width [min]**	**Area**	**Height**	**Area%**
15.929	1.65	2690.70	87.20	35.18
18.266	2.39	4956.99	169.33	64.82

Figure S49. *rac*-[1,1'-binaphthalene]-2,2'-diamine (*rac*-**4a**)

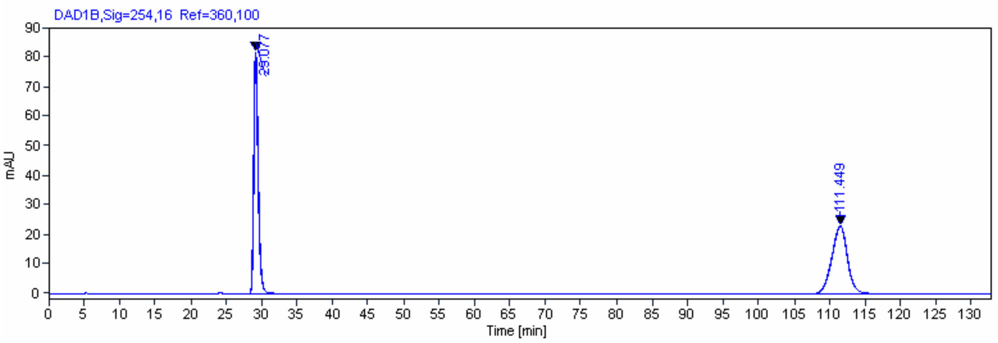

Signal:	DAD1B,Sig=254,16 Ref=360,100			
**RT [min]**	**Width [min]**	**Area**	**Height**	**Area%**
29.077	6.00	3462.43	81.85	49.87
111.449	12.49	3480.12	22.95	50.13

Figure S50. (R_a)-[1,1'-binaphthalene]-2,2'-diamine] [(R_a)-**4a**, 31% *ee*]

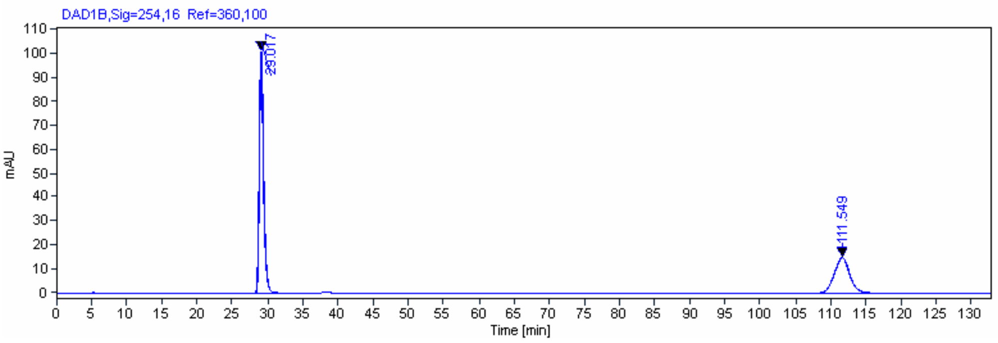

Signal:	DAD1B,Sig=254,16 Ref=360,100			
**RT [min]**	**Width [min]**	**Area**	**Height**	**Area%**
29.017	6.98	4255.27	100.95	65.43
111.549	12.36	2247.80	14.77	34.57

12 NMR Spectra

Figure S51. **^{1}H NMR** spectrum (500 MHz, $CDCl_3$, 298 K) of **2b**.

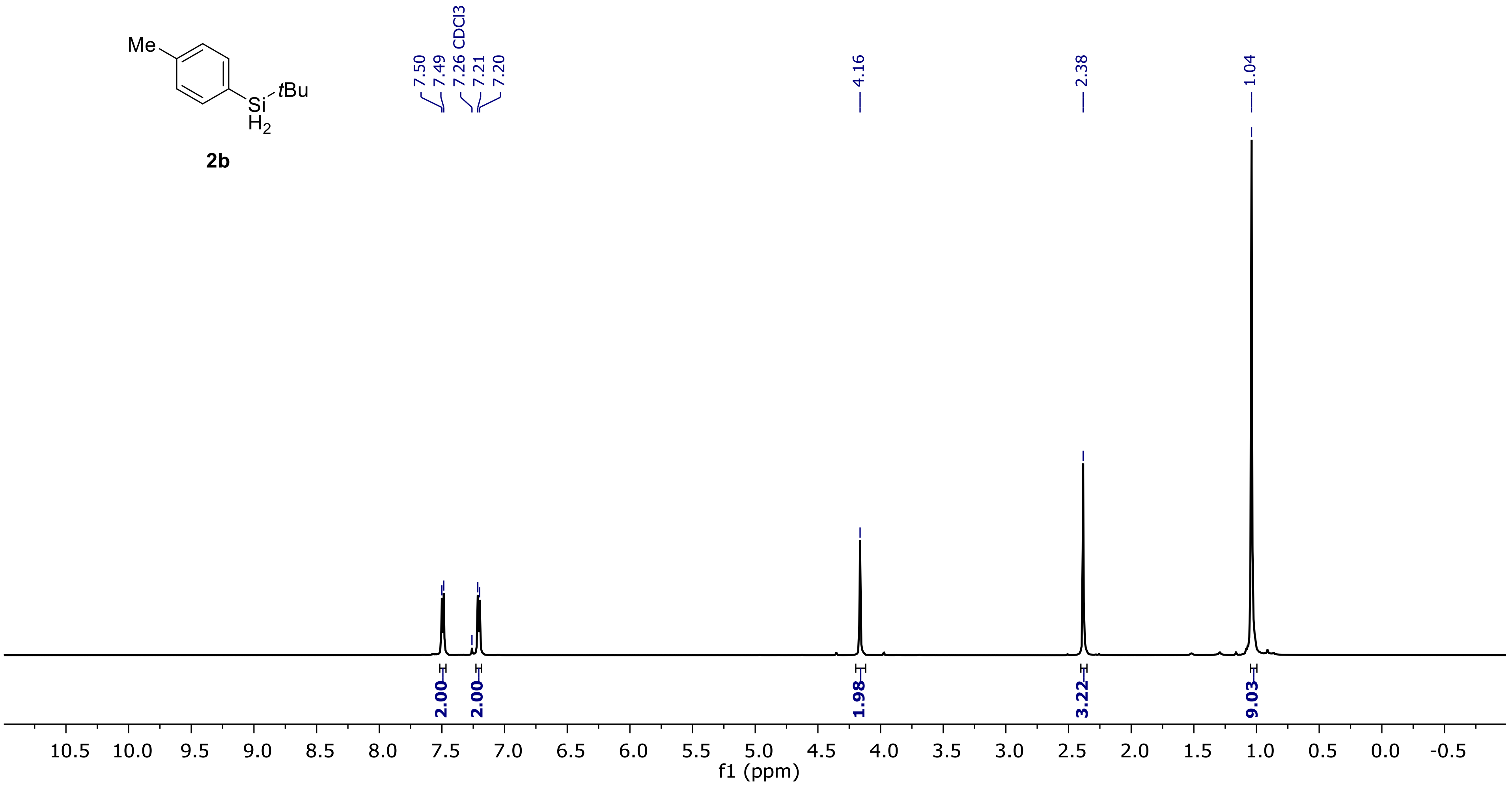

Figure S52. $^{13}C\{^{1}H\}$ **NMR** spectrum (126 MHz, $CDCl_3$, 298 K) of **2b**.

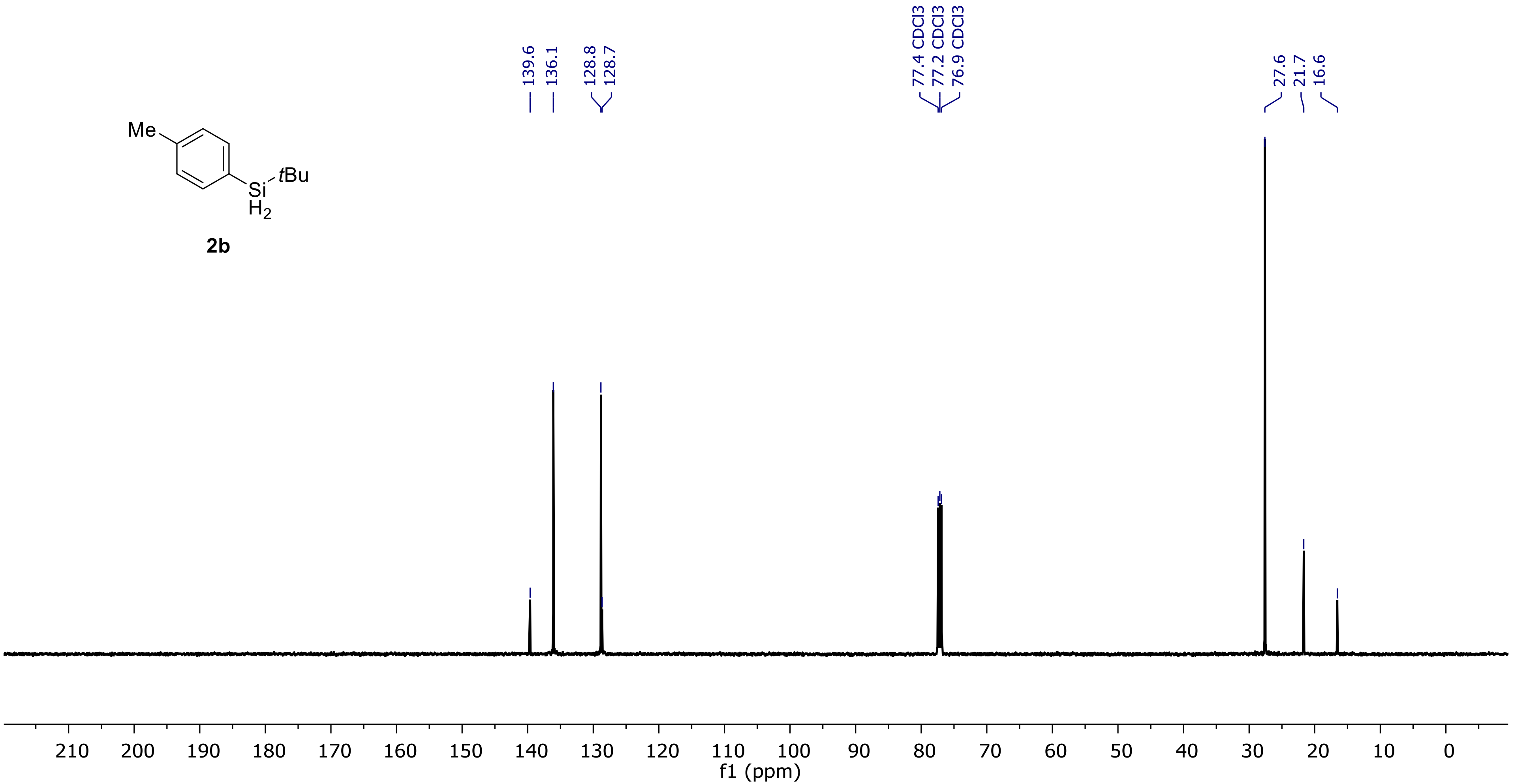

Figure S53. ^{29}**Si DEPT NMR** spectrum (99 MHz, $CDCl_3$, 298 K, optimized for *J* = 7.0 Hz) of **2b**.

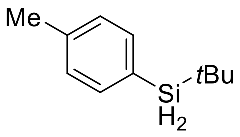

2b

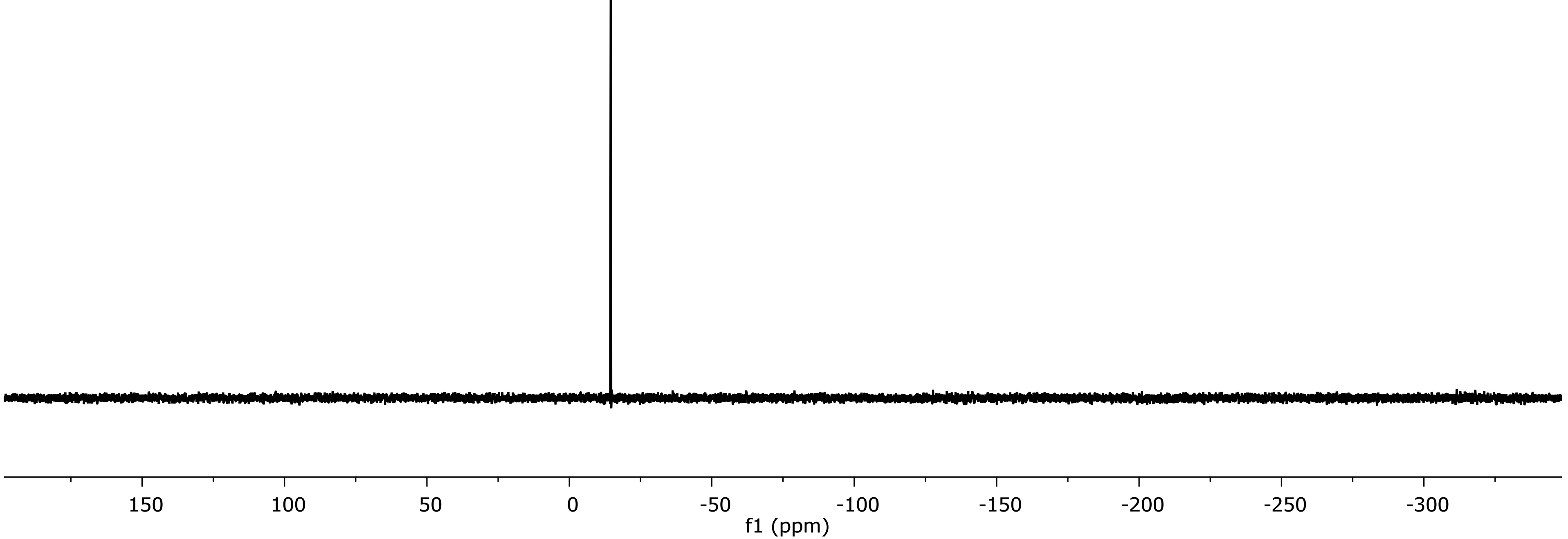

Figure S54. **^{1}H NMR** spectrum (500 MHz, $CDCl_3$, 298 K) of **2c**.

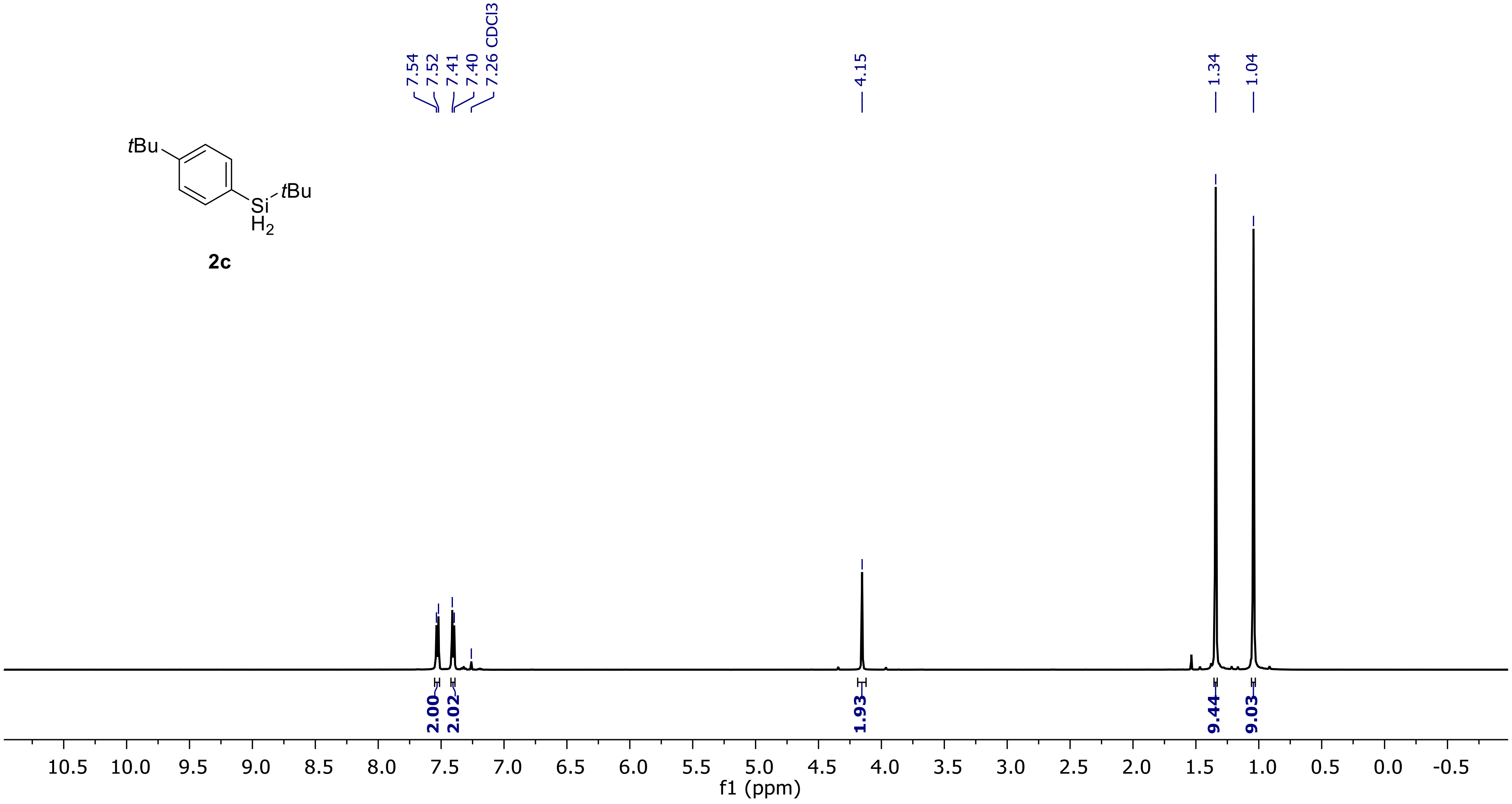

Figure S55. **$^{13}C\{^1H\}$ NMR** spectrum (126 MHz, $CDCl_3$, 298 K) of **2c**.

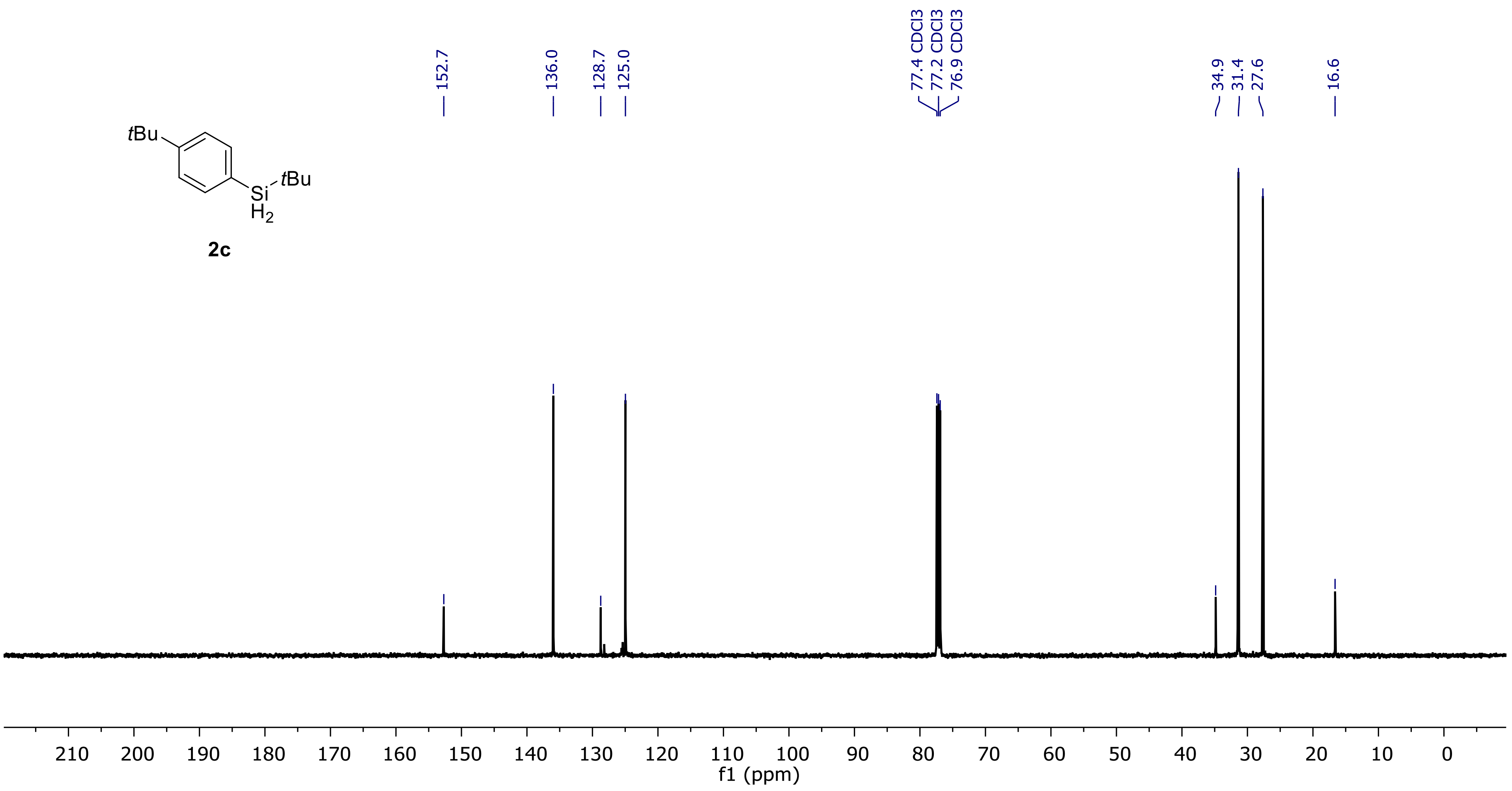

Figure S56. ^{29}Si DEPT NMR spectrum (99 MHz, $CDCl_3$, 298 K, optimized for *J* = 7.0 Hz) of **2c**.

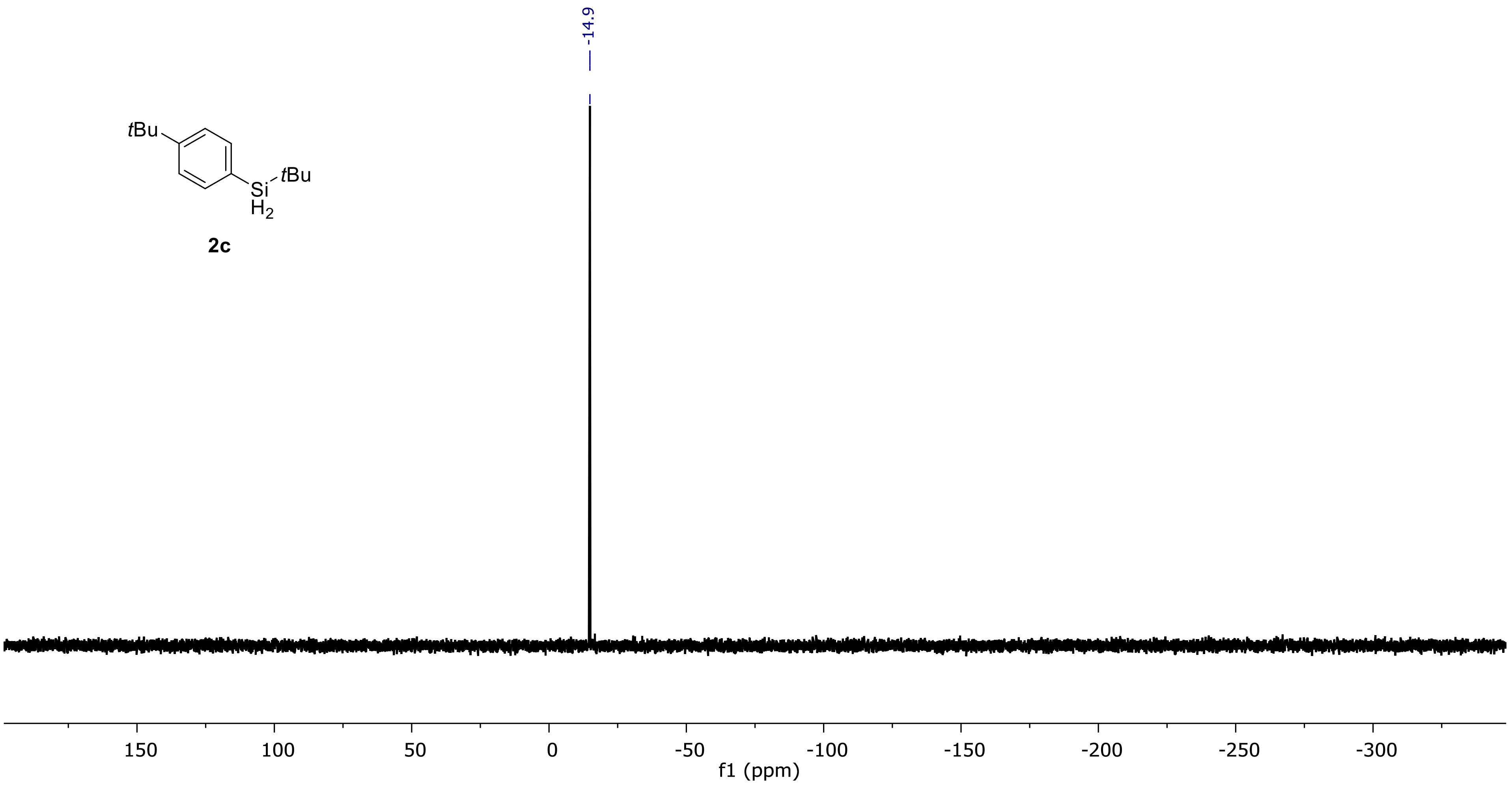

Figure S57. ^{1}**H NMR** spectrum (500 MHz, $CDCl_3$, 298 K) of **2f**.

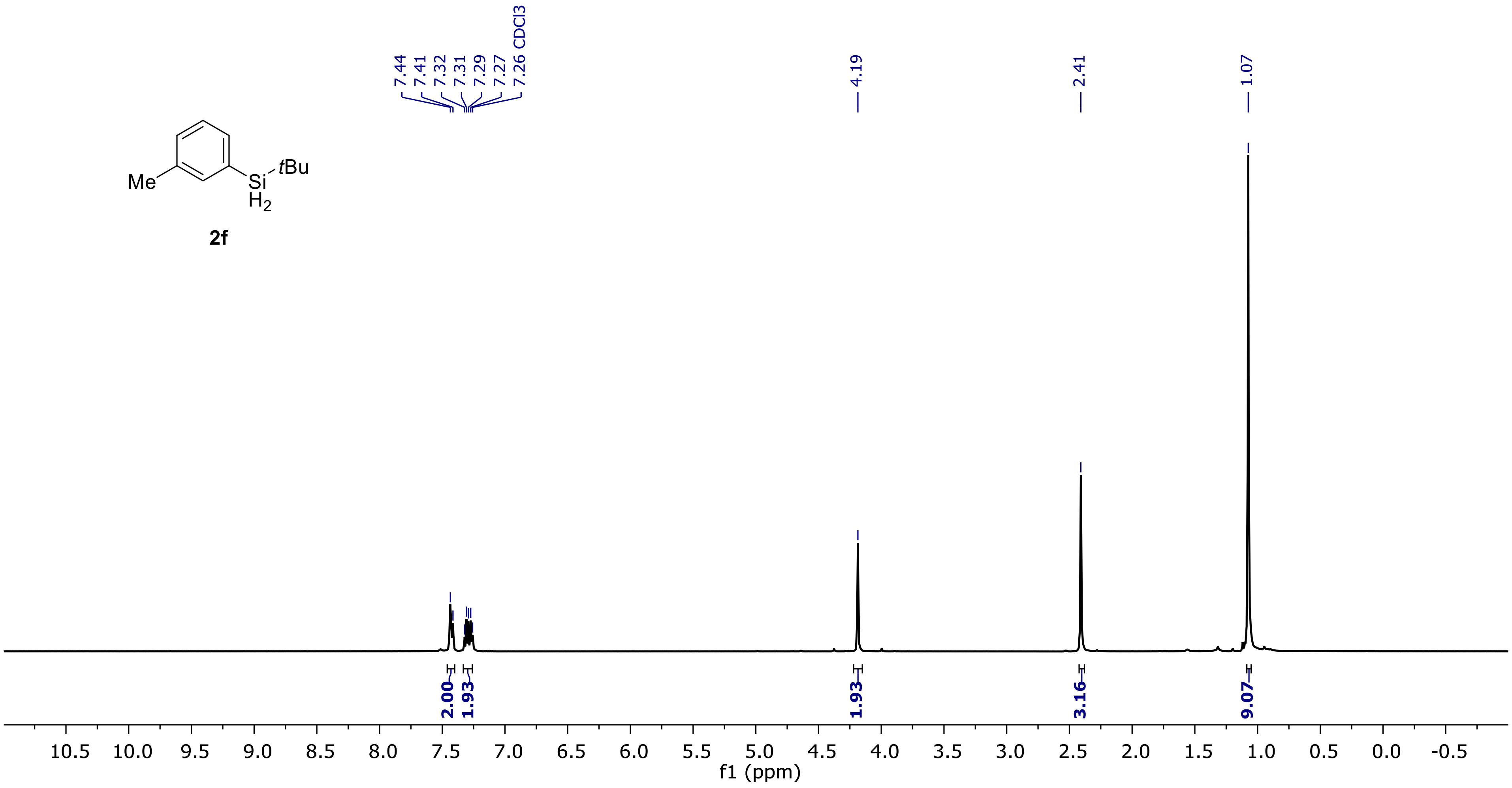

Figure S58. ^{13}**C{^{1}H} NMR** spectrum (126 MHz, $CDCl_3$, 298 K) of **2f**.

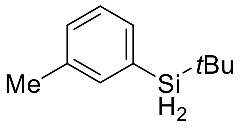

2f

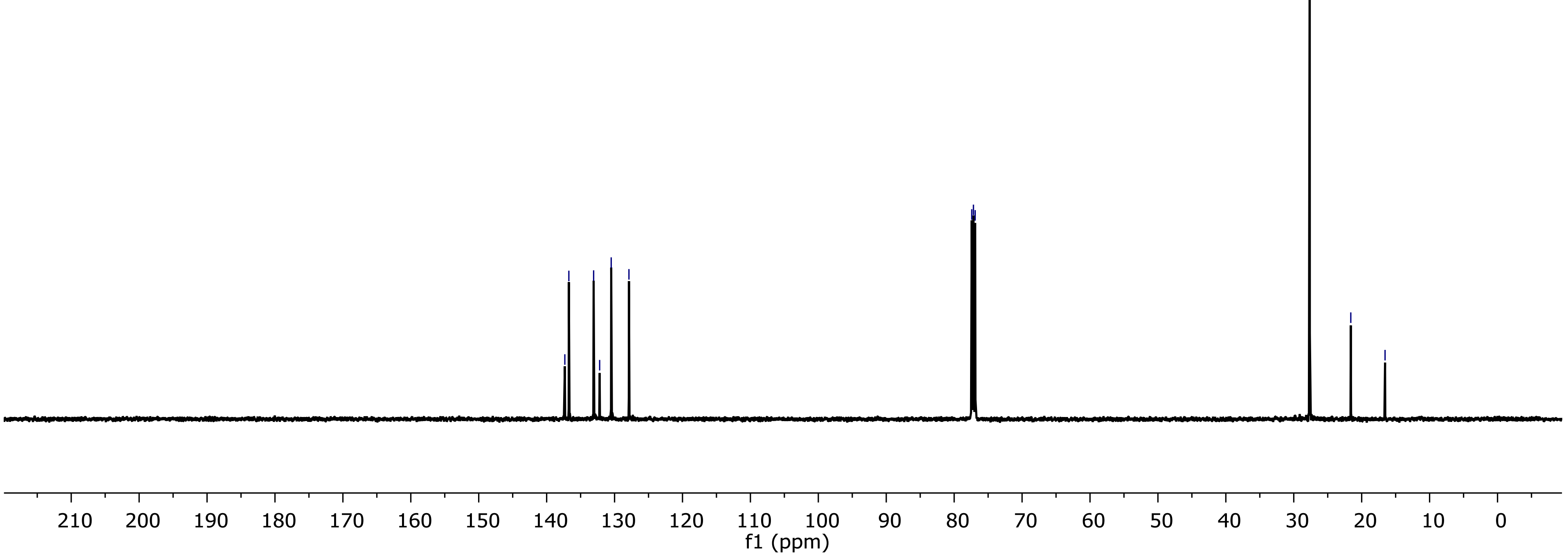

Figure S59. ^{29}**Si DEPT NMR** spectrum (99 MHz, $CDCl_3$, 298 K, optimized for *J* = 7.0 Hz) of **2f**.

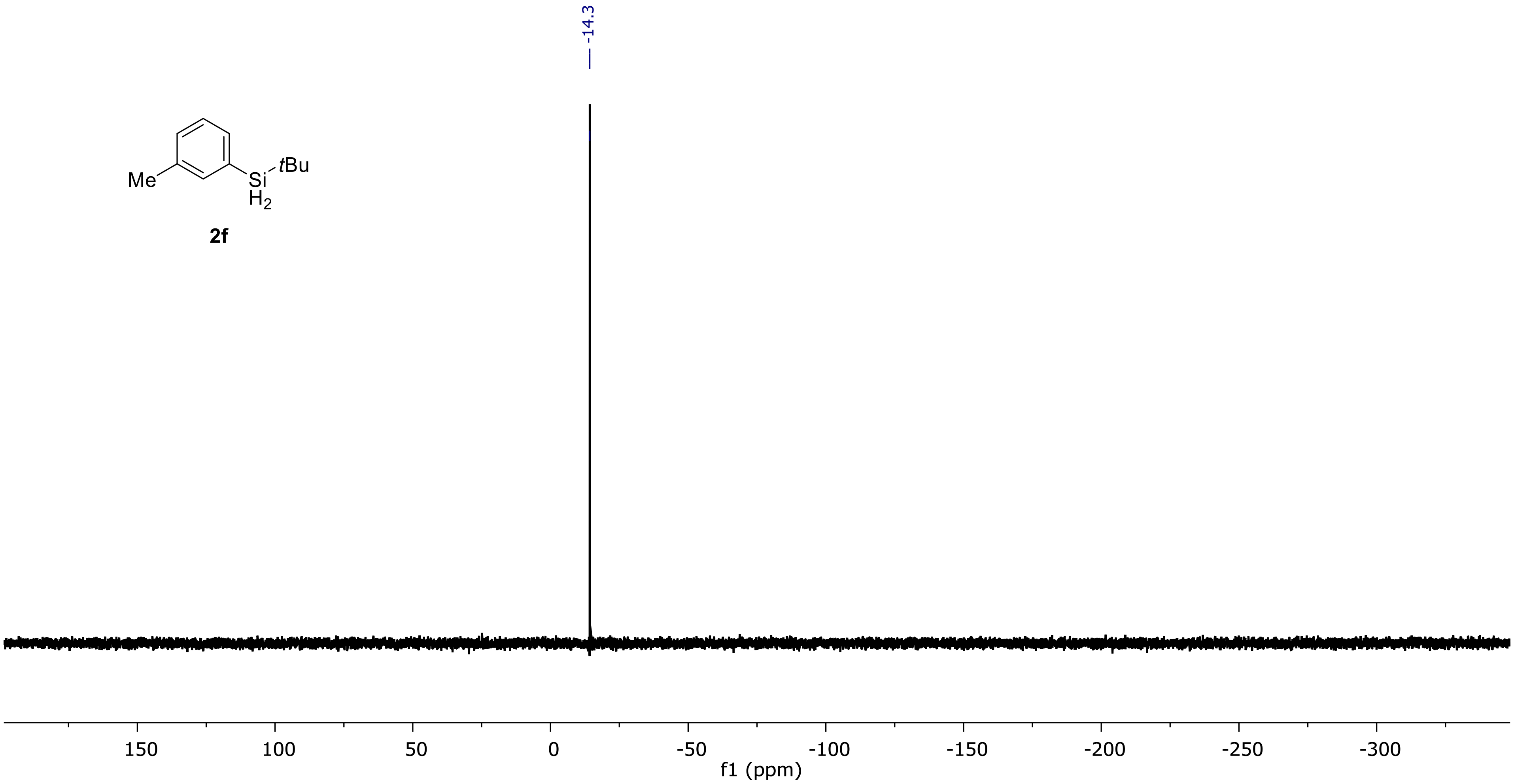

Figure S60. **¹H NMR** spectrum (500 MHz, $CDCl_3$, 298 K) of **2h**.

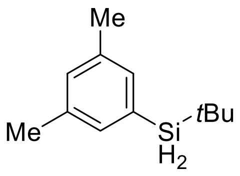

2h

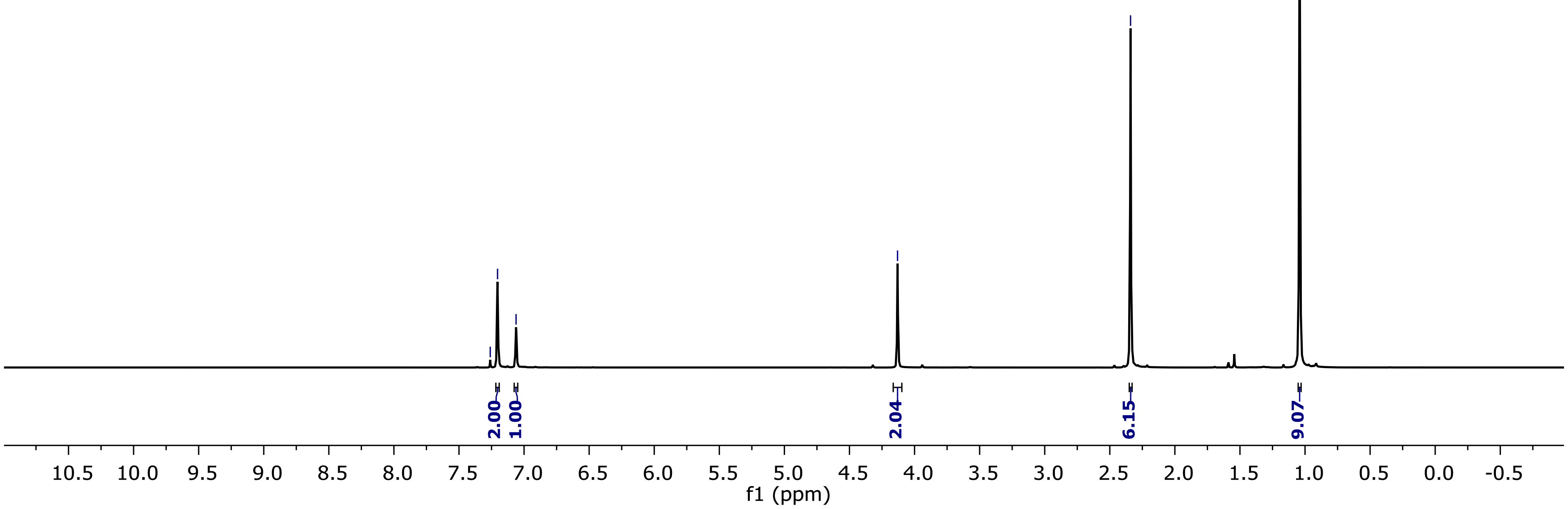

Figure S61. **$^{13}C\{^1H\}$ NMR** spectrum (126 MHz, $CDCl_3$, 298 K) of **2h**.

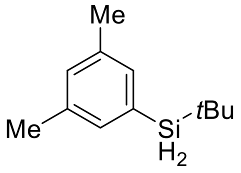

2h

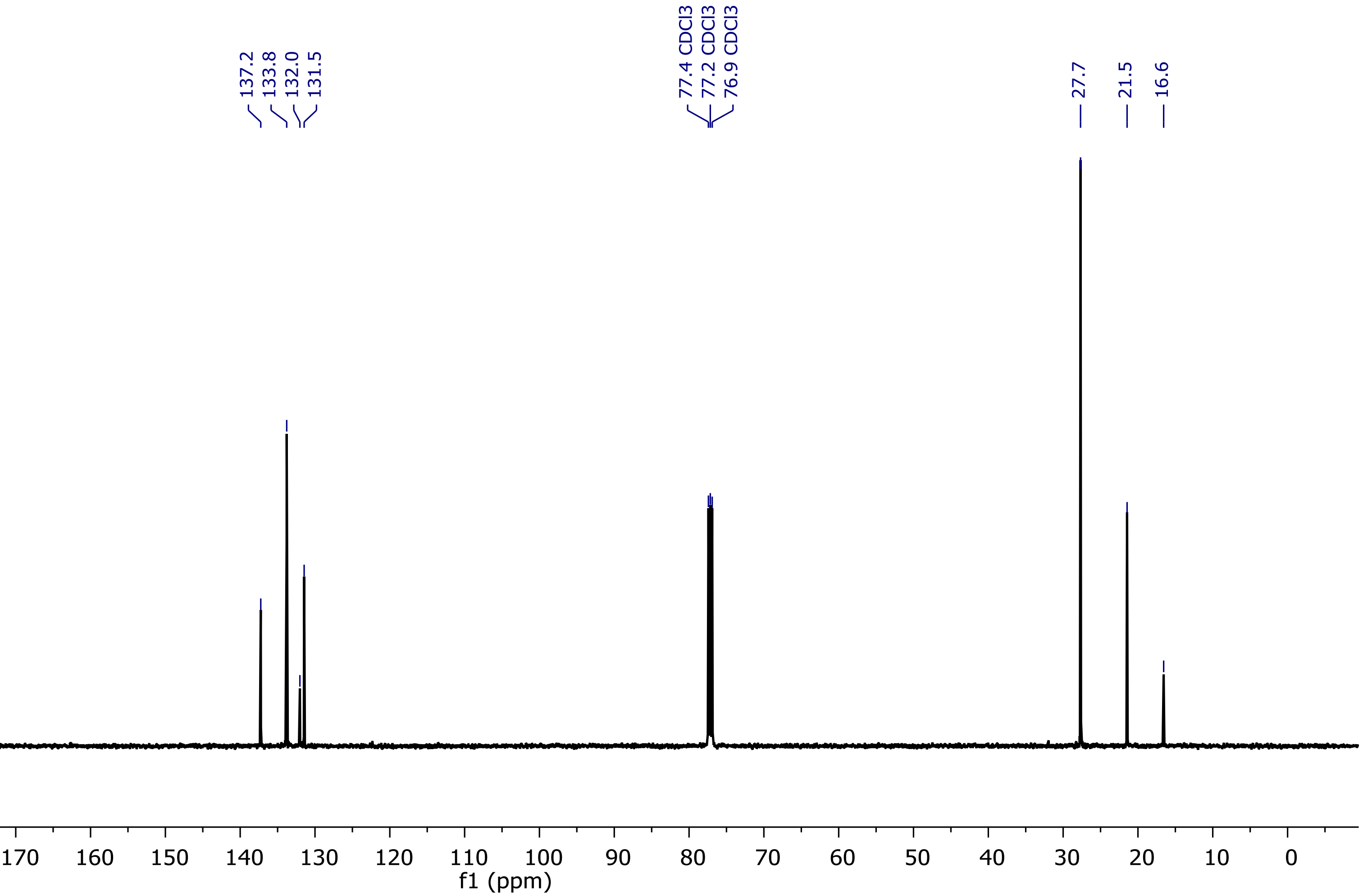

Figure S62. ^{29}**Si DEPT NMR** spectrum (99 MHz, $CDCl_3$, 298 K, optimized for J = 7.0 Hz) of **2h**.

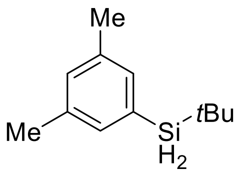

2h

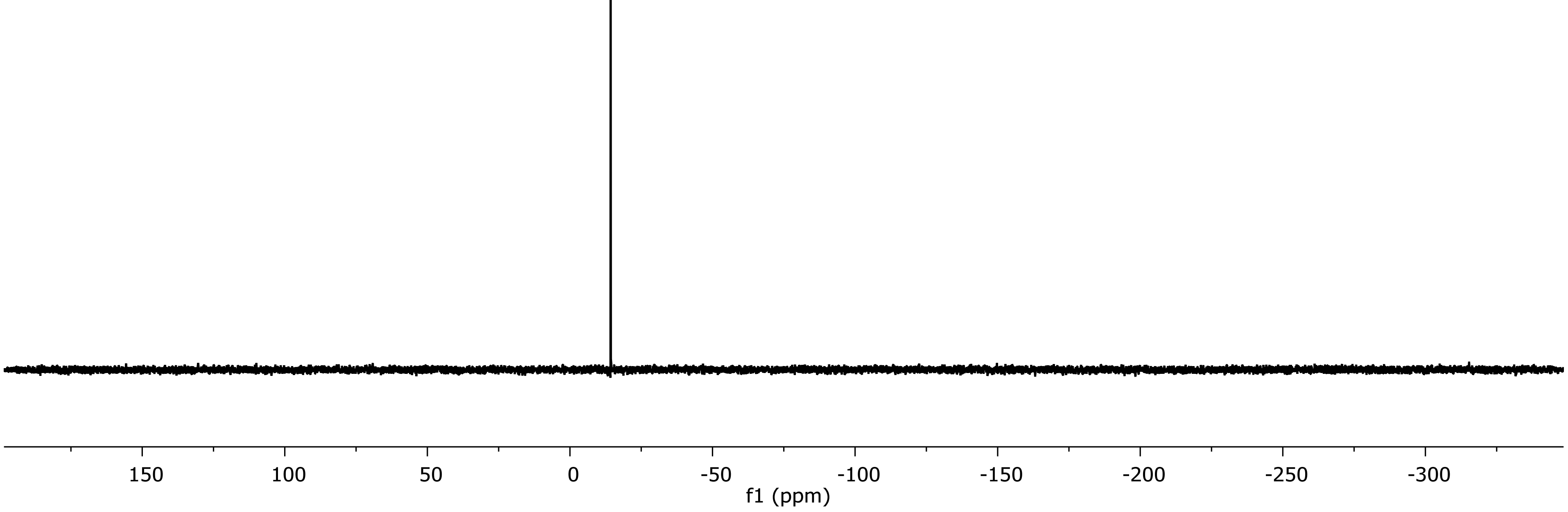

Figure S63. **^{1}H NMR** spectrum (500 MHz, $CDCl_3$, 298 K) of **2i**.

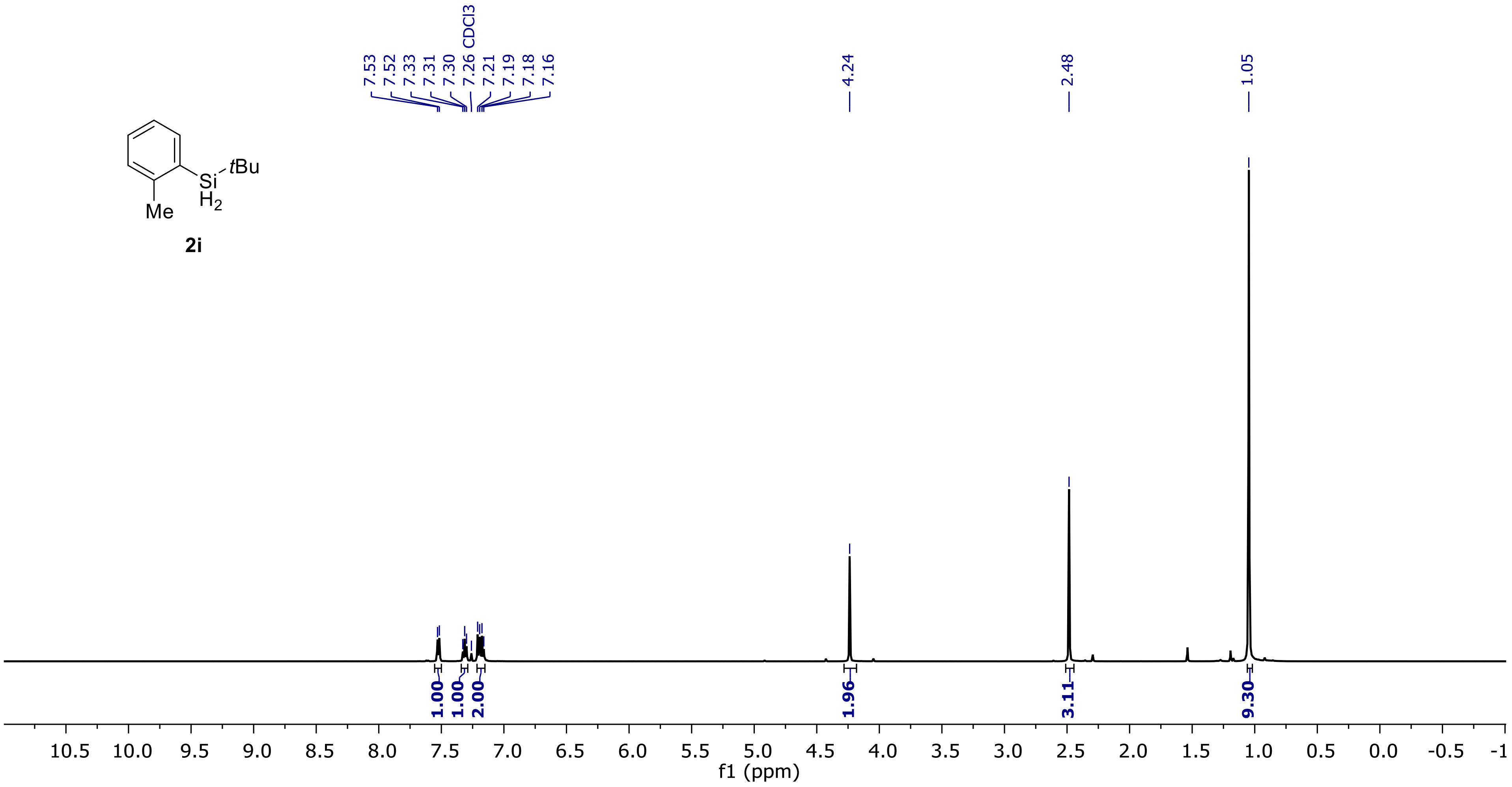

Figure S64. $^{13}C\{^1H\}$ **NMR** spectrum (126 MHz, $CDCl_3$, 298 K) of **2i**.

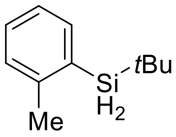

2i

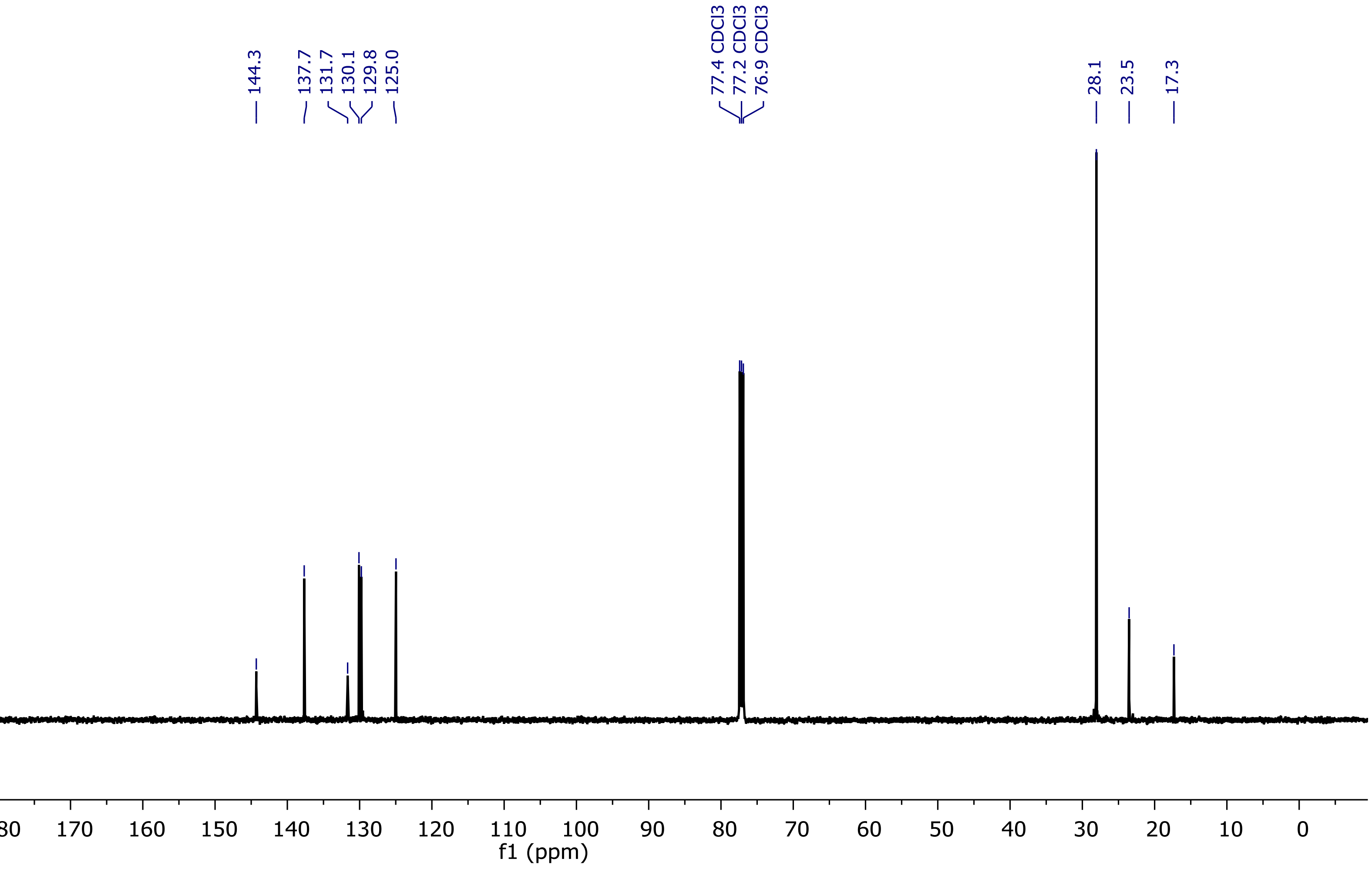

Figure S65. ^{29}**Si DEPT NMR** spectrum (99 MHz, $CDCl_3$, 298 K, optimized for *J* = 7.0 Hz) of **2i**.

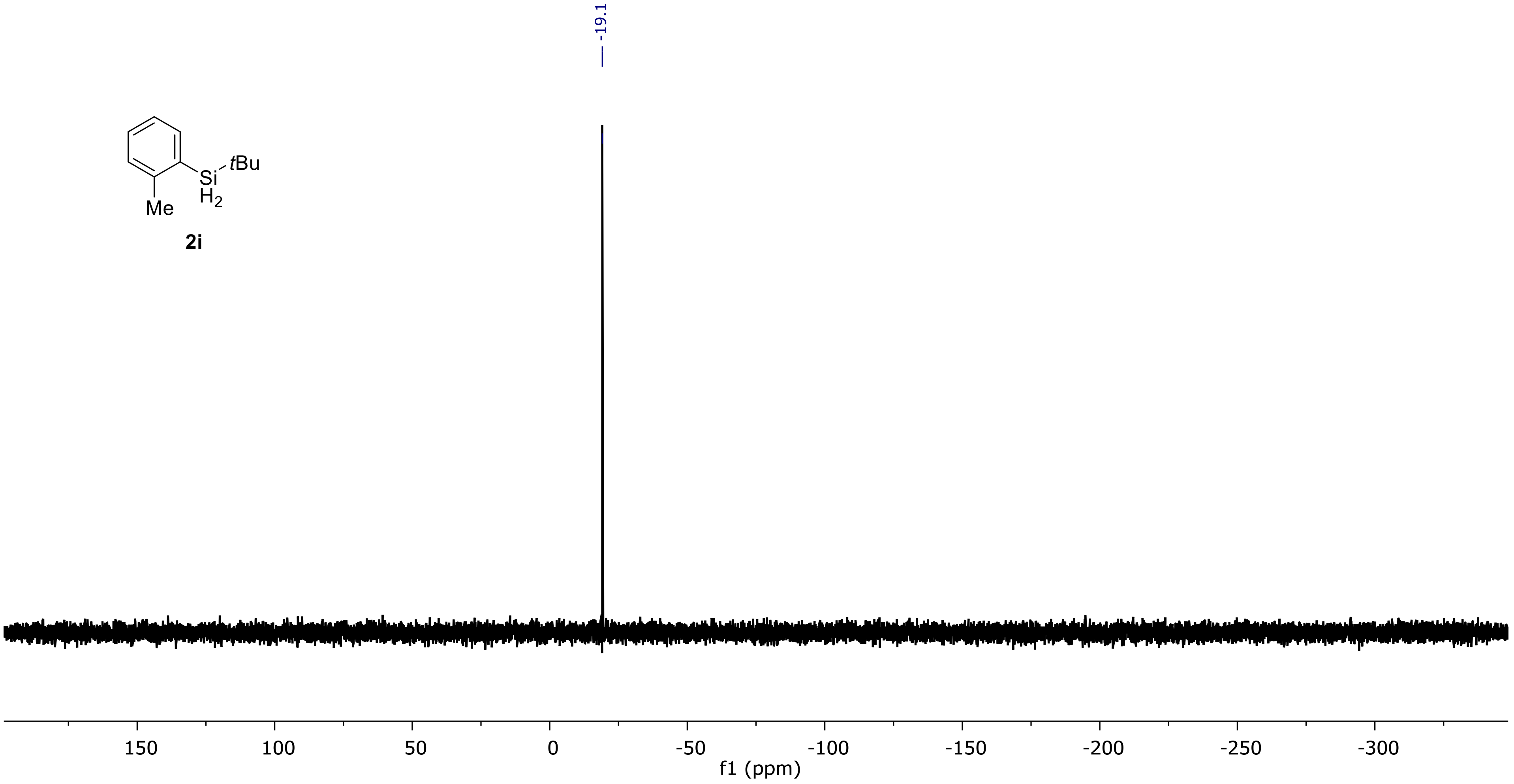

Figure S66. **^{1}H NMR** spectrum (500 MHz, $CDCl_3$, 298 K) of **2j**.

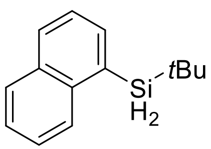

2j

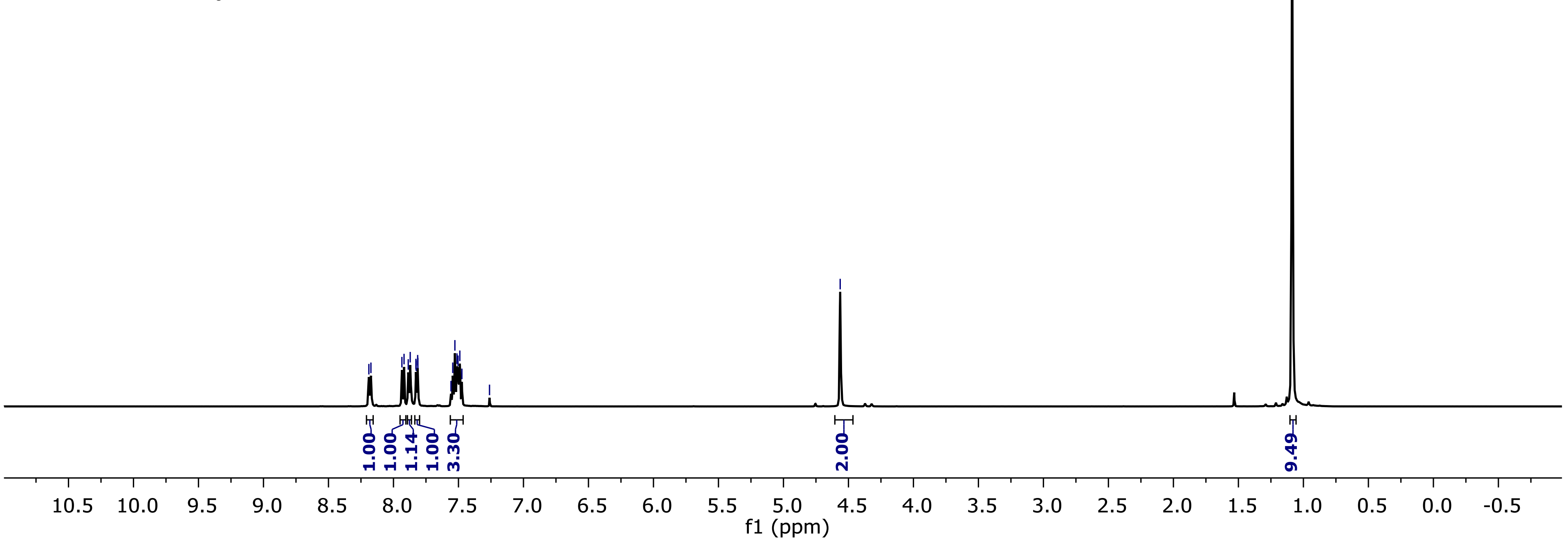

Figure S67. **$^{13}C\{^{1}H\}$ NMR** spectrum (126 MHz, $CDCl_3$, 298 K) of **2j**.

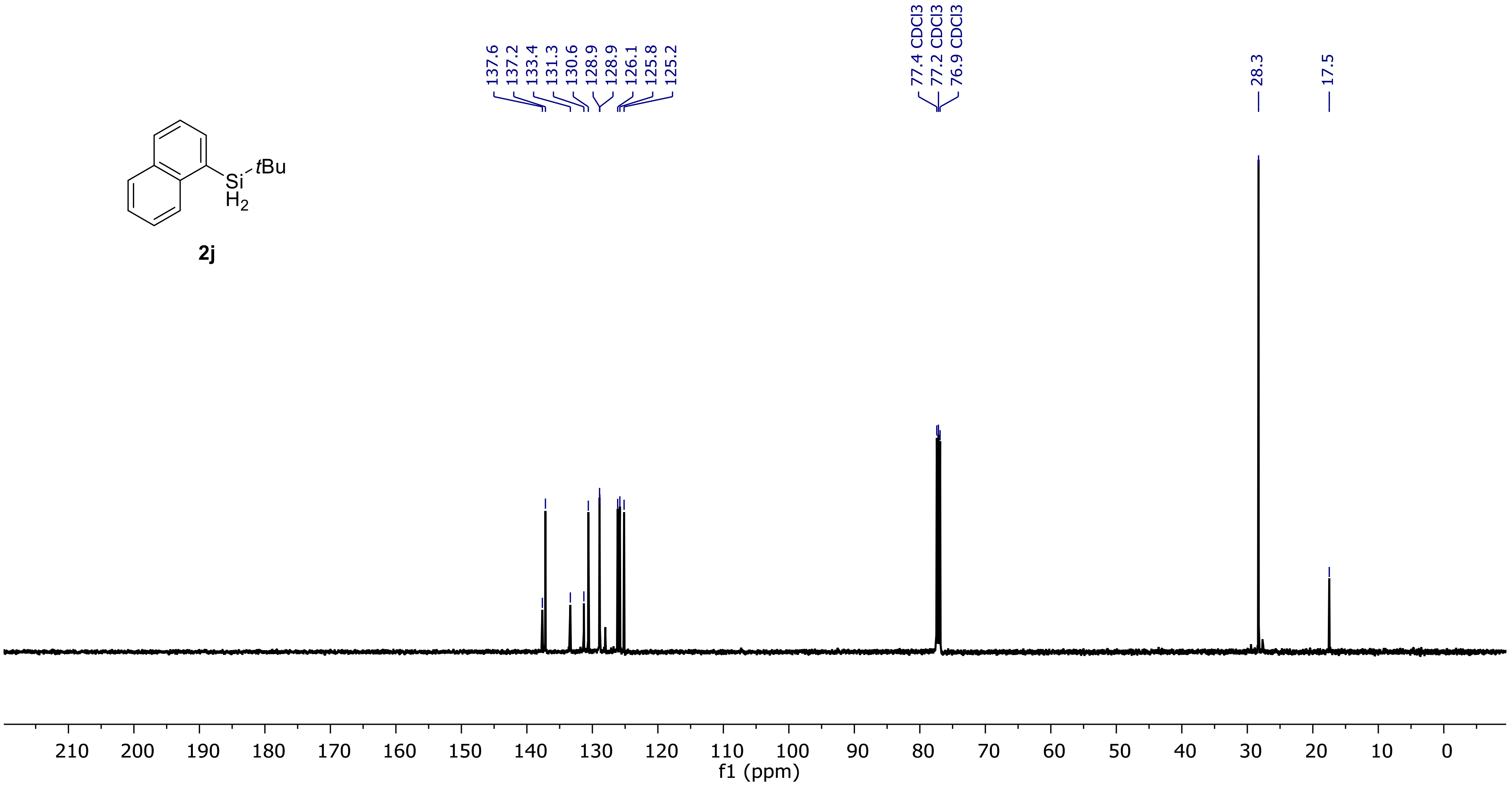

Figure S68. **^{29}Si DEPT NMR** spectrum (99 MHz, $CDCl_3$, 298 K, optimized for J = 7.0 Hz) of **2j**.

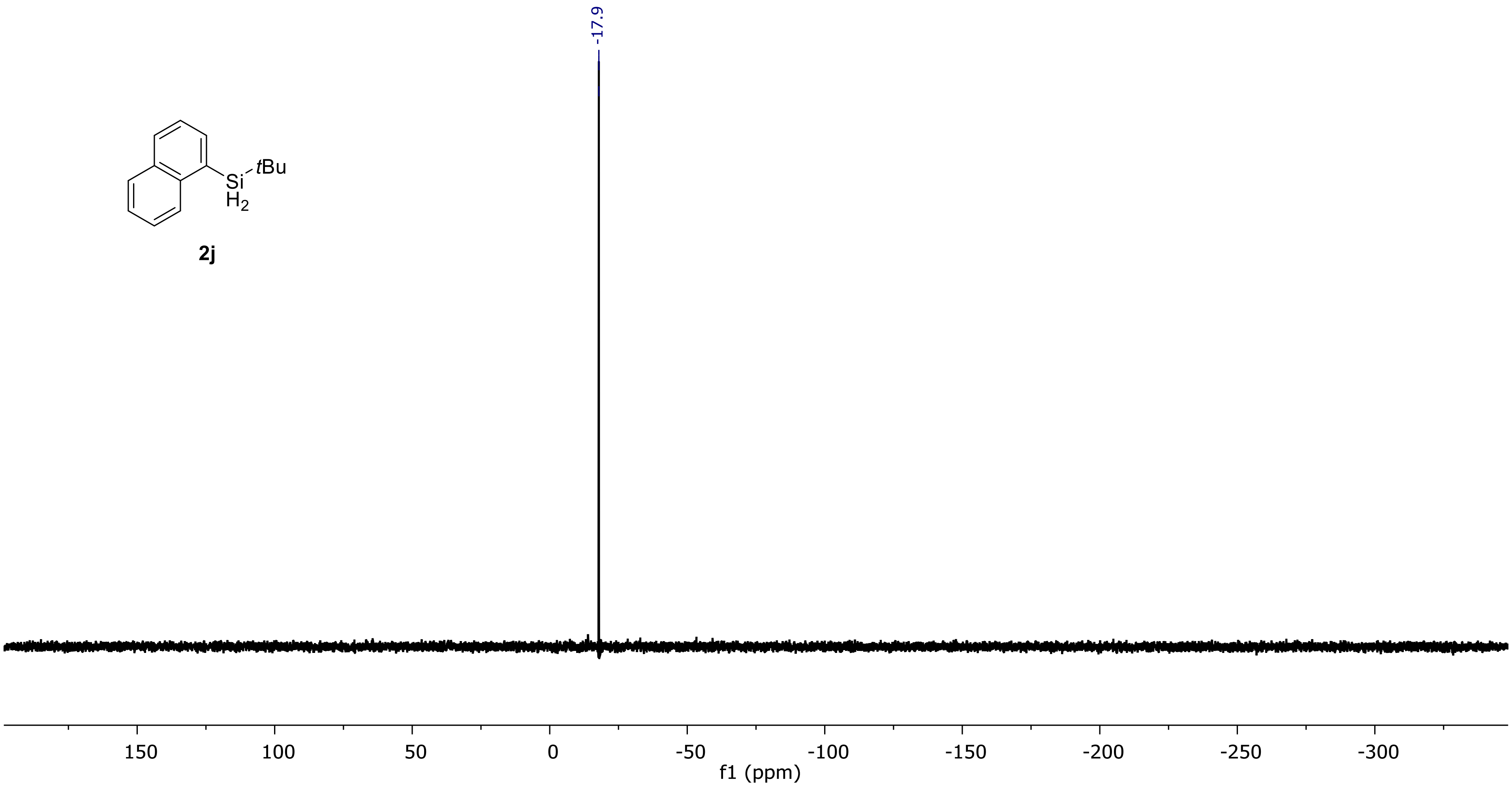

Figure S69. **^{1}H NMR** spectrum (500 MHz, $CDCl_3$, 298 K) of **2k**.

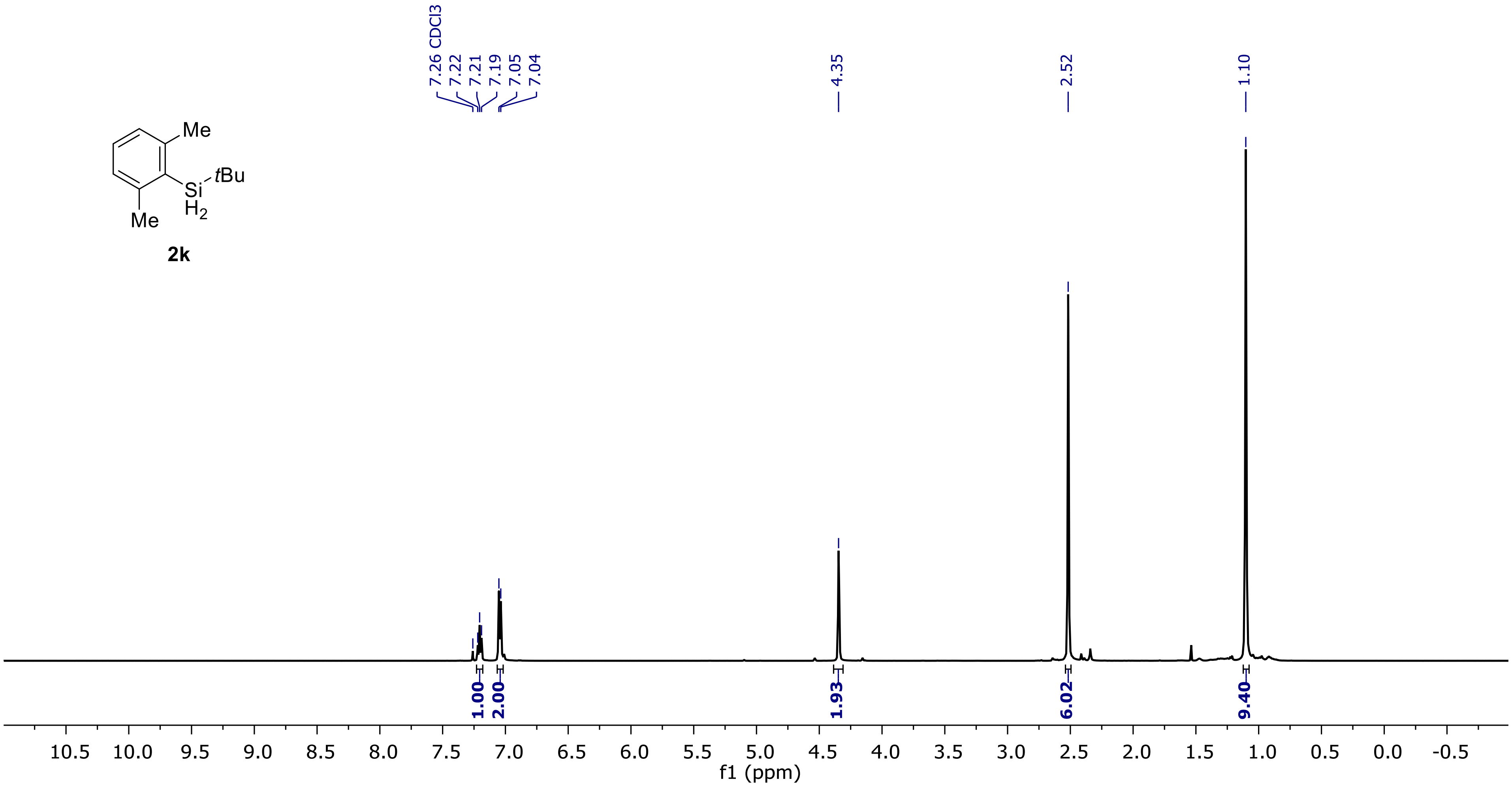

Figure S70. **$^{13}C\{^1H\}$ NMR** spectrum (126 MHz, $CDCl_3$, 298 K) of **2k**.

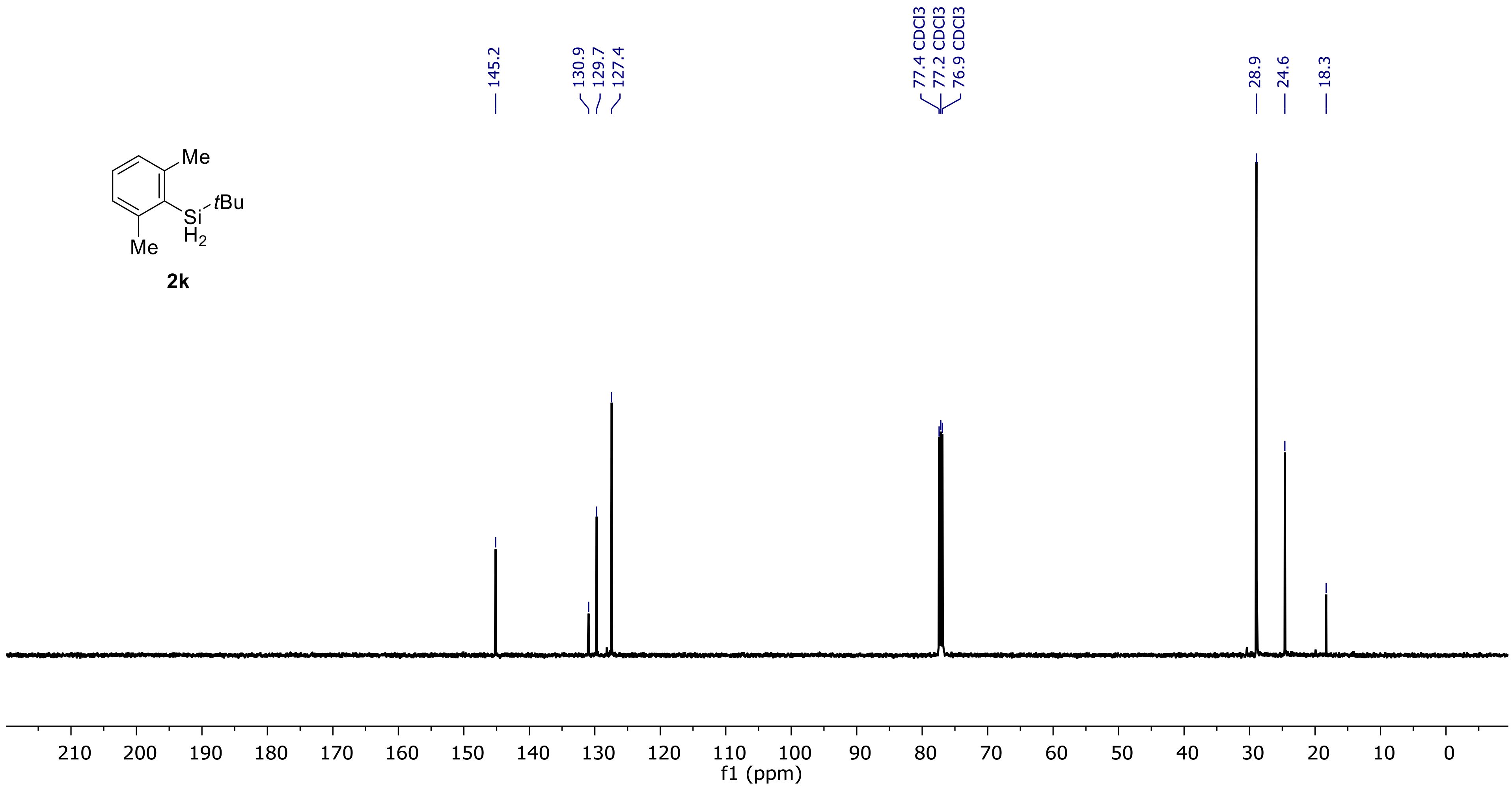

Figure S71. ^{29}Si DEPT NMR spectrum (99 MHz, $CDCl_3$, 298 K, optimized for J = 7.0 Hz) of **2k.**

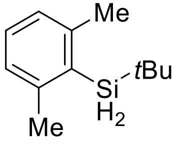

2k

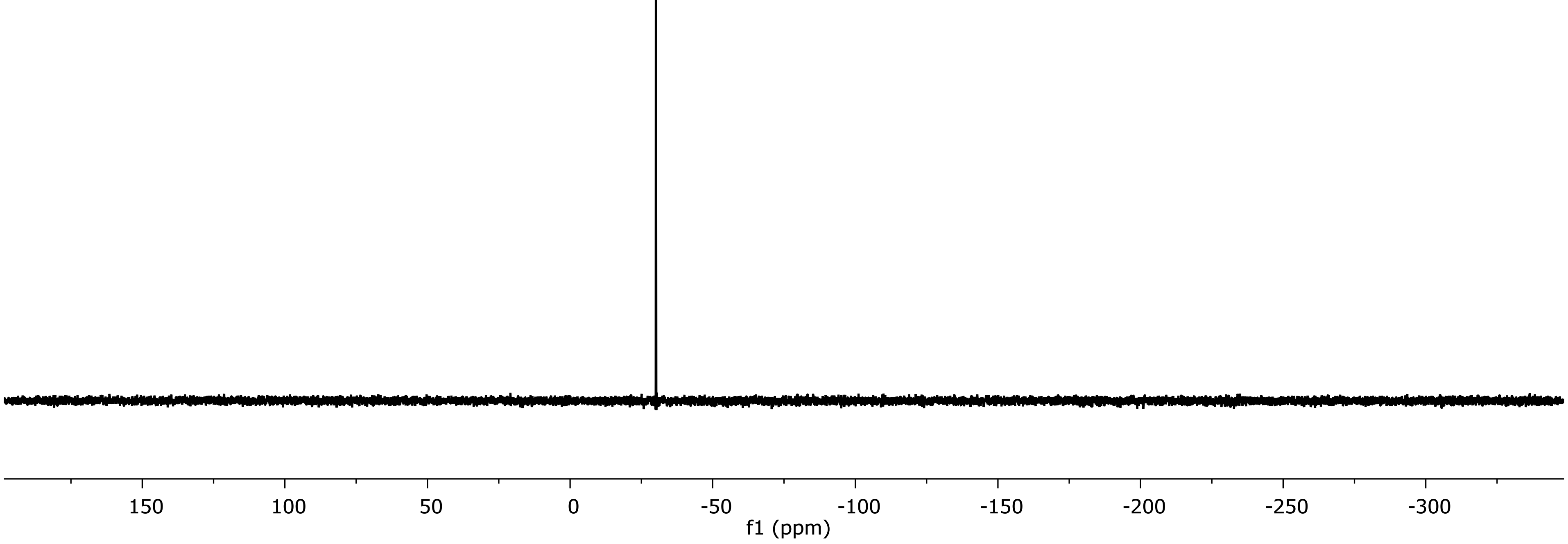

Figure S72. ^{29}**Si DEPT NMR** spectrum (99 MHz, $CDCl_3$, 298 K, optimized for J = 7.0 Hz) of **S1.**

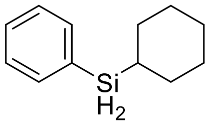

S1

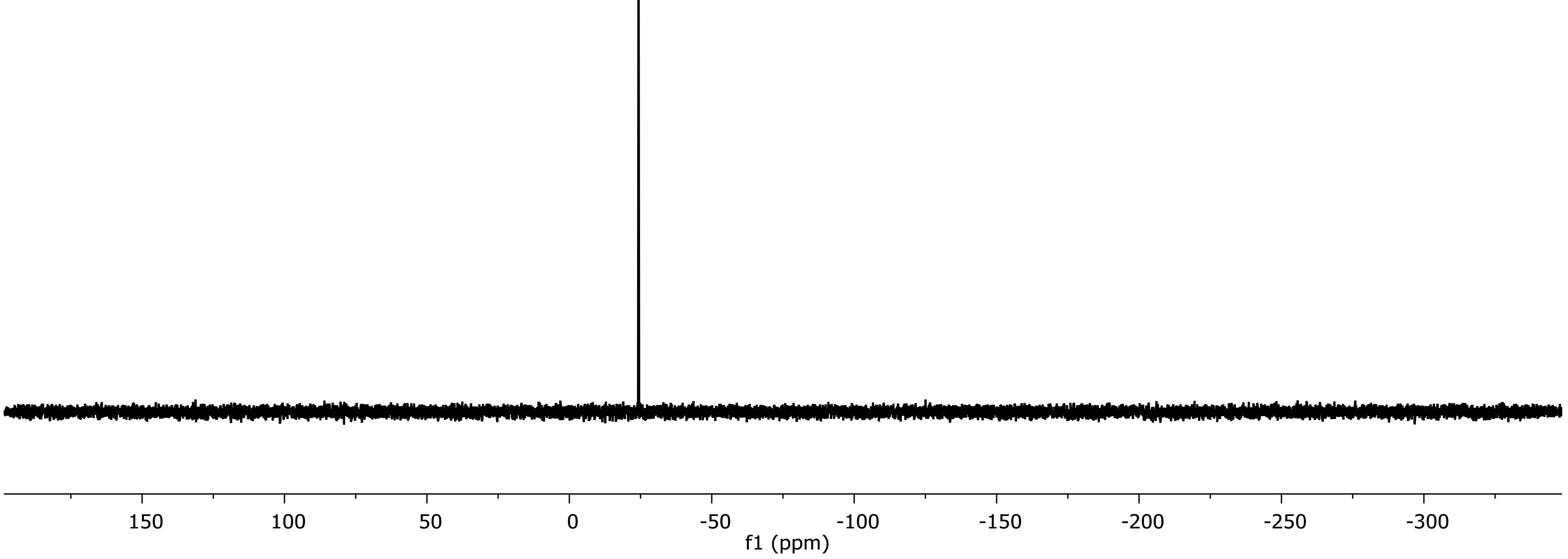

Figure S73. ^{1}**H NMR** spectrum (500 MHz, $CDCl_3$, 298 K) of **4f**.

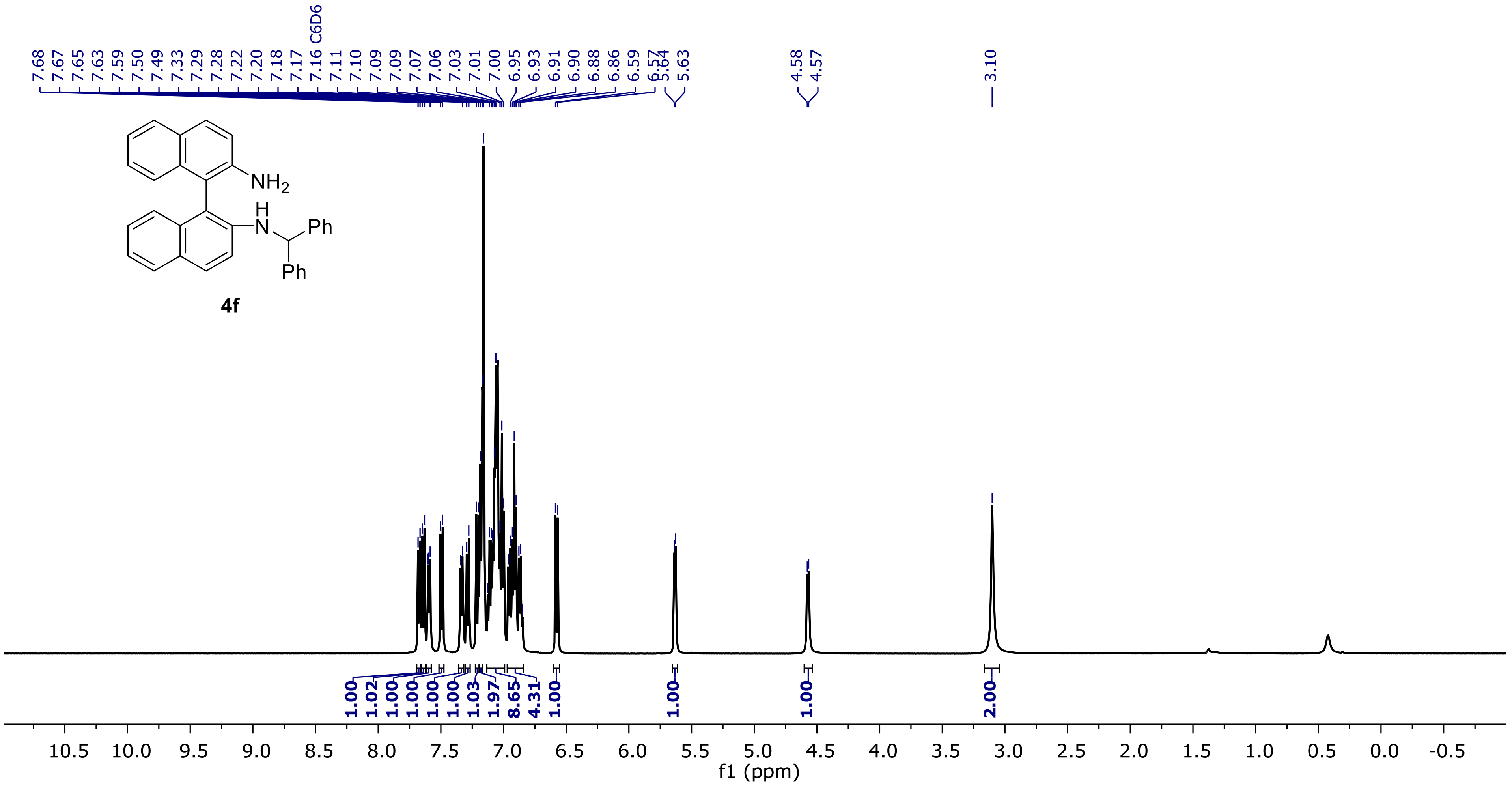

Figure S74. $^{13}C\{^{1}H\}$ NMR spectrum (126 MHz, $CDCl_3$, 298 K) of **4f**.

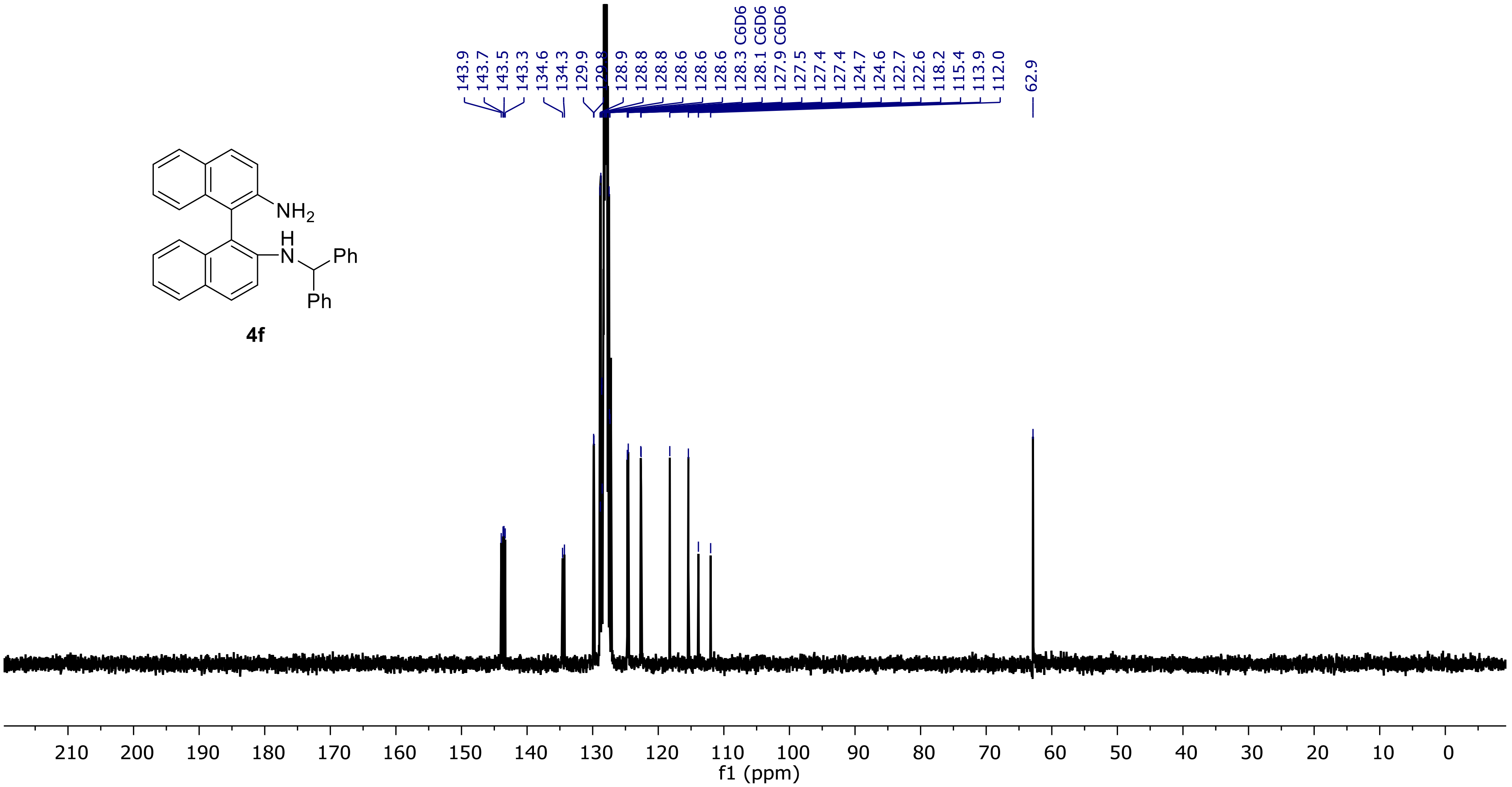

Figure S75. **^{1}H NMR** spectrum (500 MHz, C_6D_6, 298 K) of **4g**.

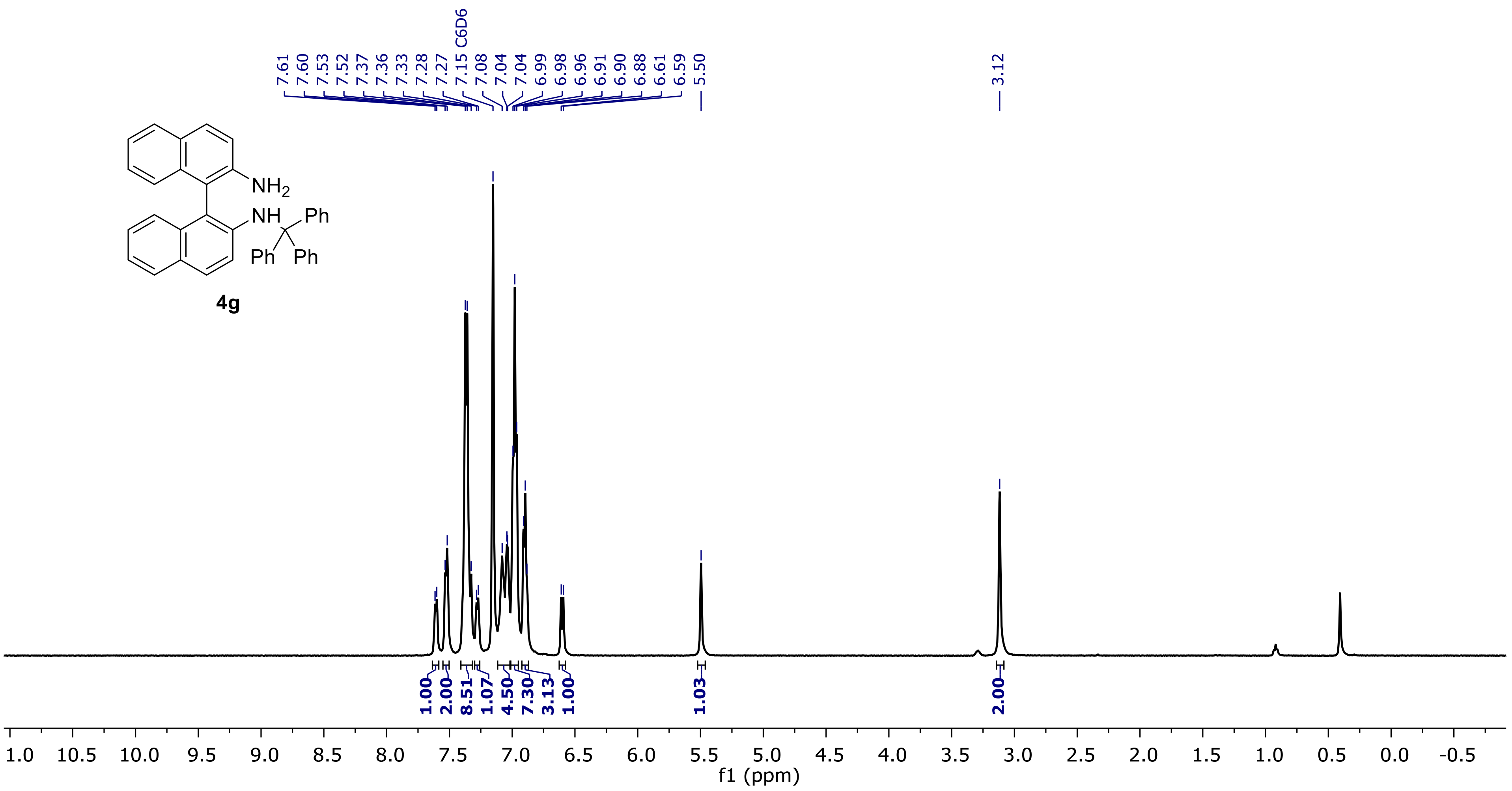

Figure S76. ^{13}**C{**^{1}**H} NMR** spectrum (101 MHz, C_6D_6, 298 K) of **4g**.

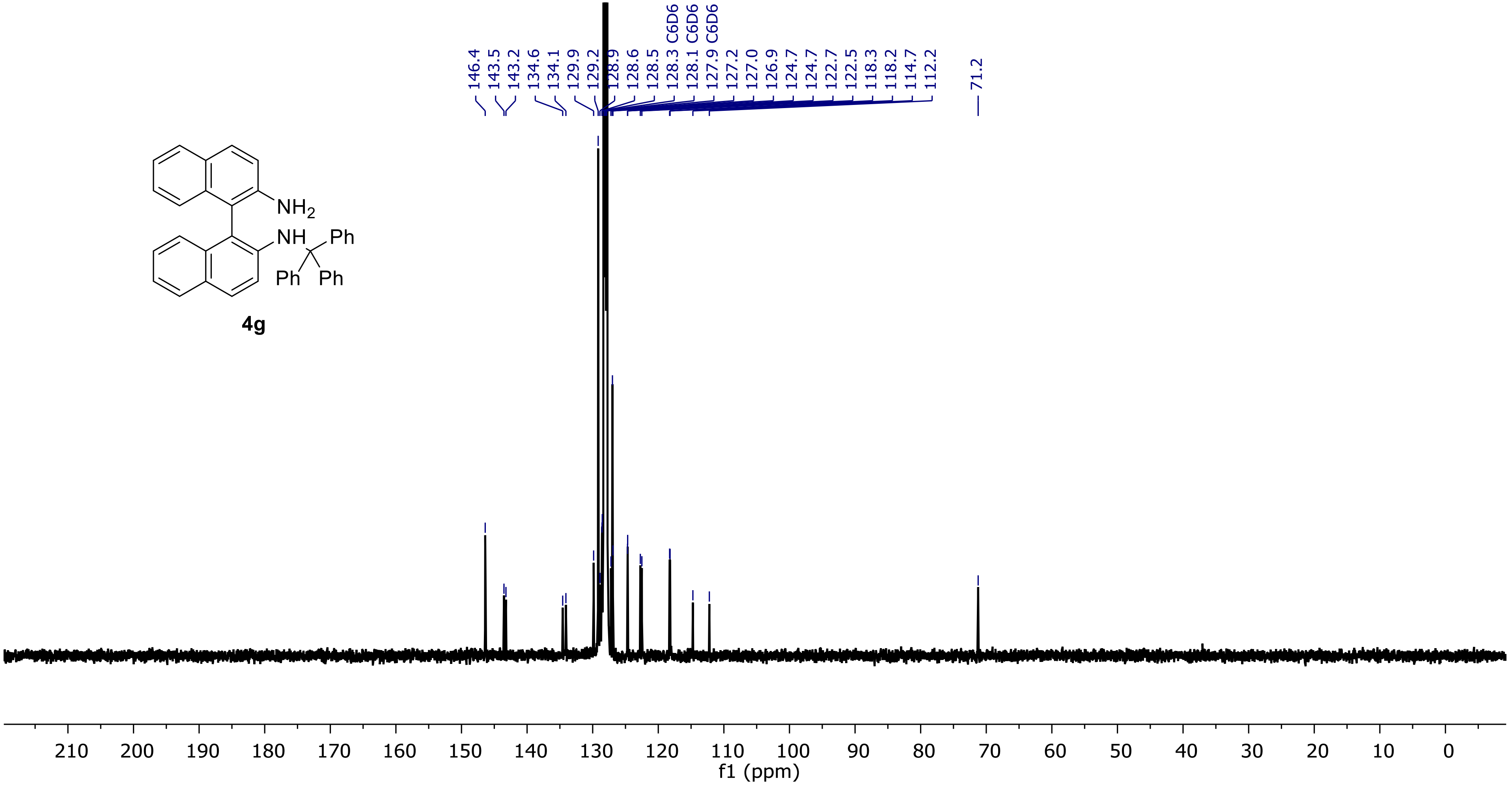

Figure S76. **^{1}H NMR** spectrum (500 MHz, C_6D_6, 298 K) of **S2**.

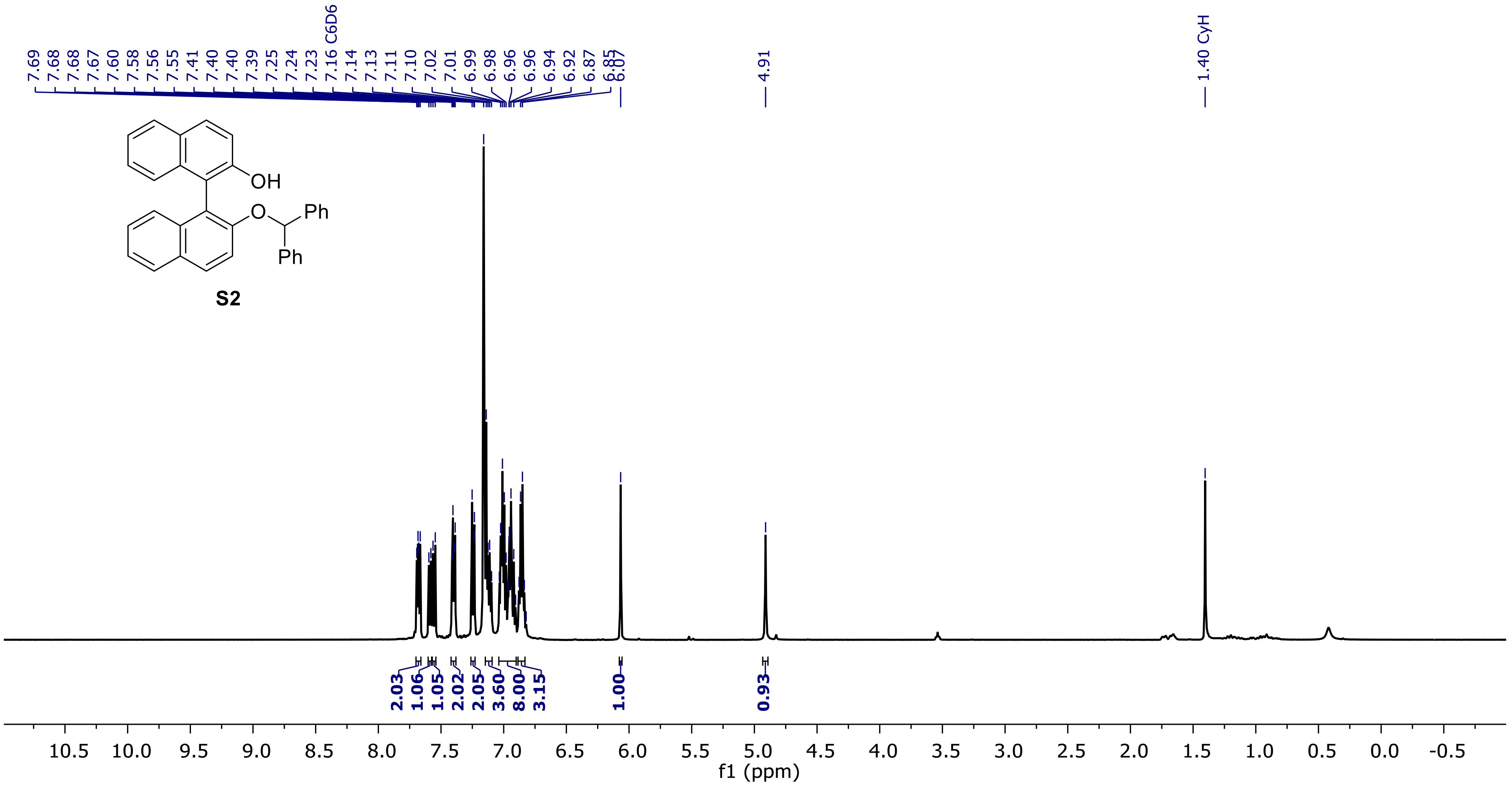

Figure S77. ^{13}C{^{1}H} NMR spectrum (126 MHz, C_6D_6, 298 K) of **S2**.

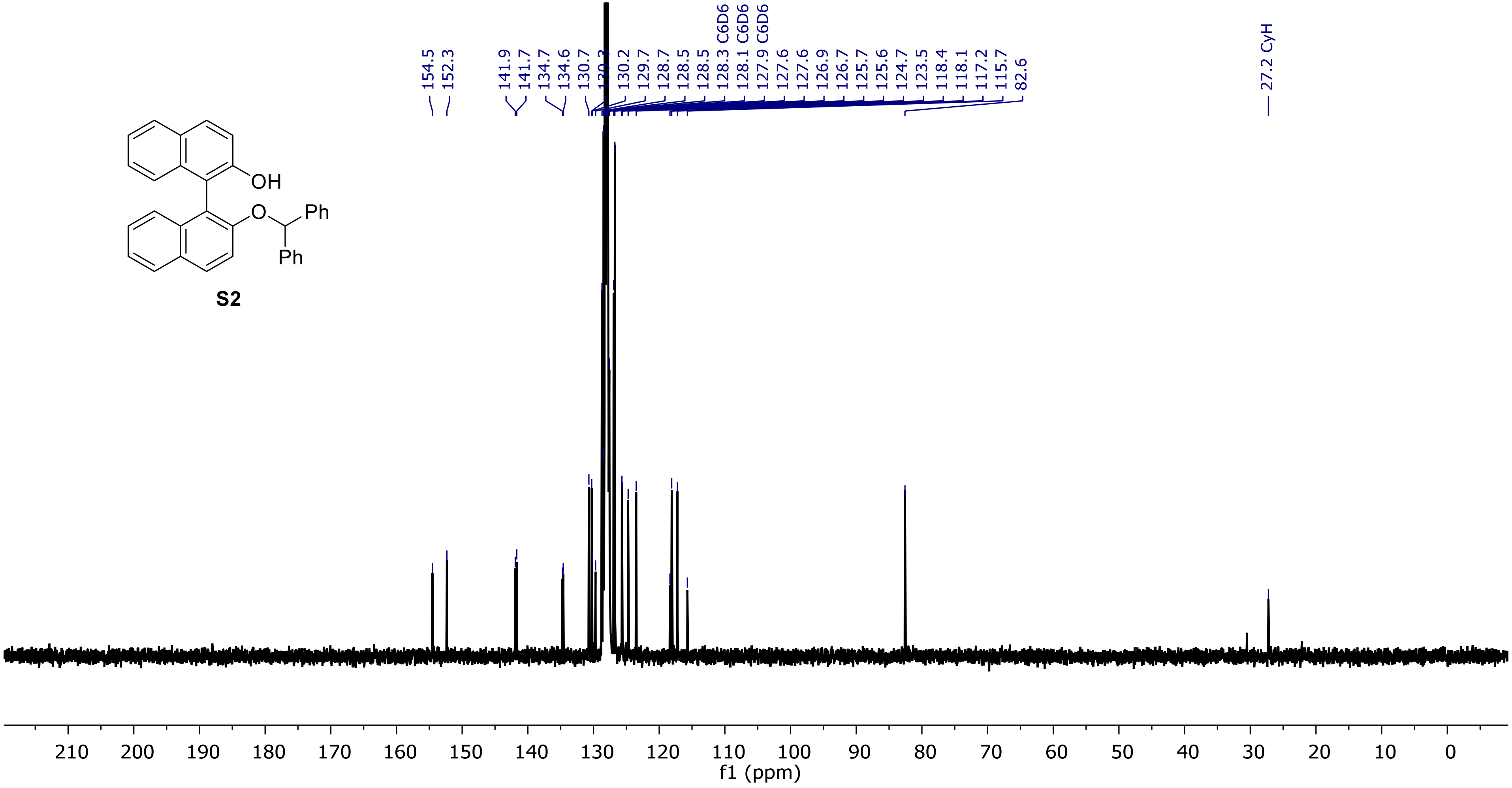

S2

Figure S78. **^{1}H NMR** spectrum (400 MHz, C_6D_6, 298 K) of **4h**.

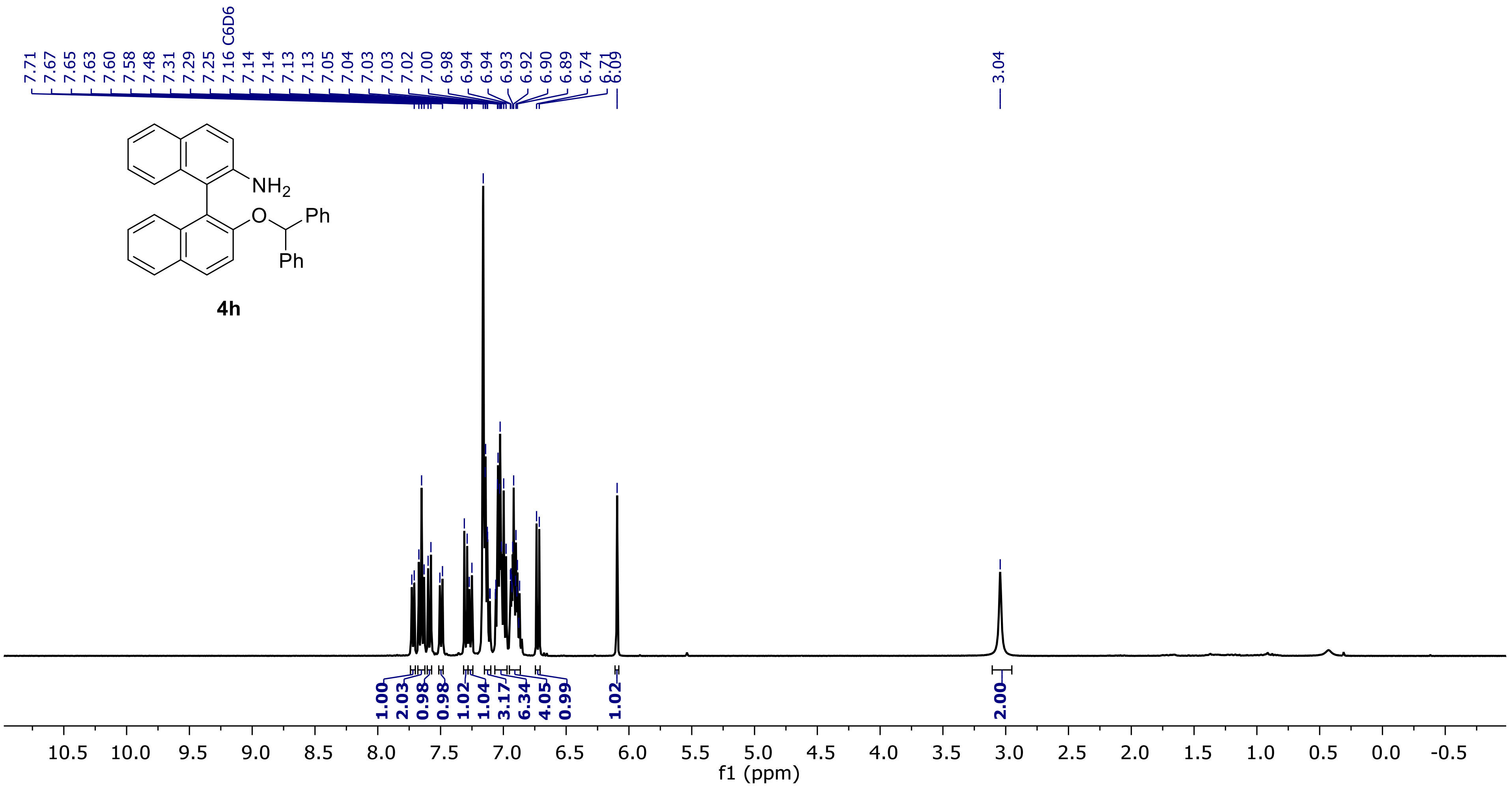

Figure S79. ^{13}**C{^{1}H} NMR** spectrum (101 MHz, C_6D_6, 298 K) of **4h**.

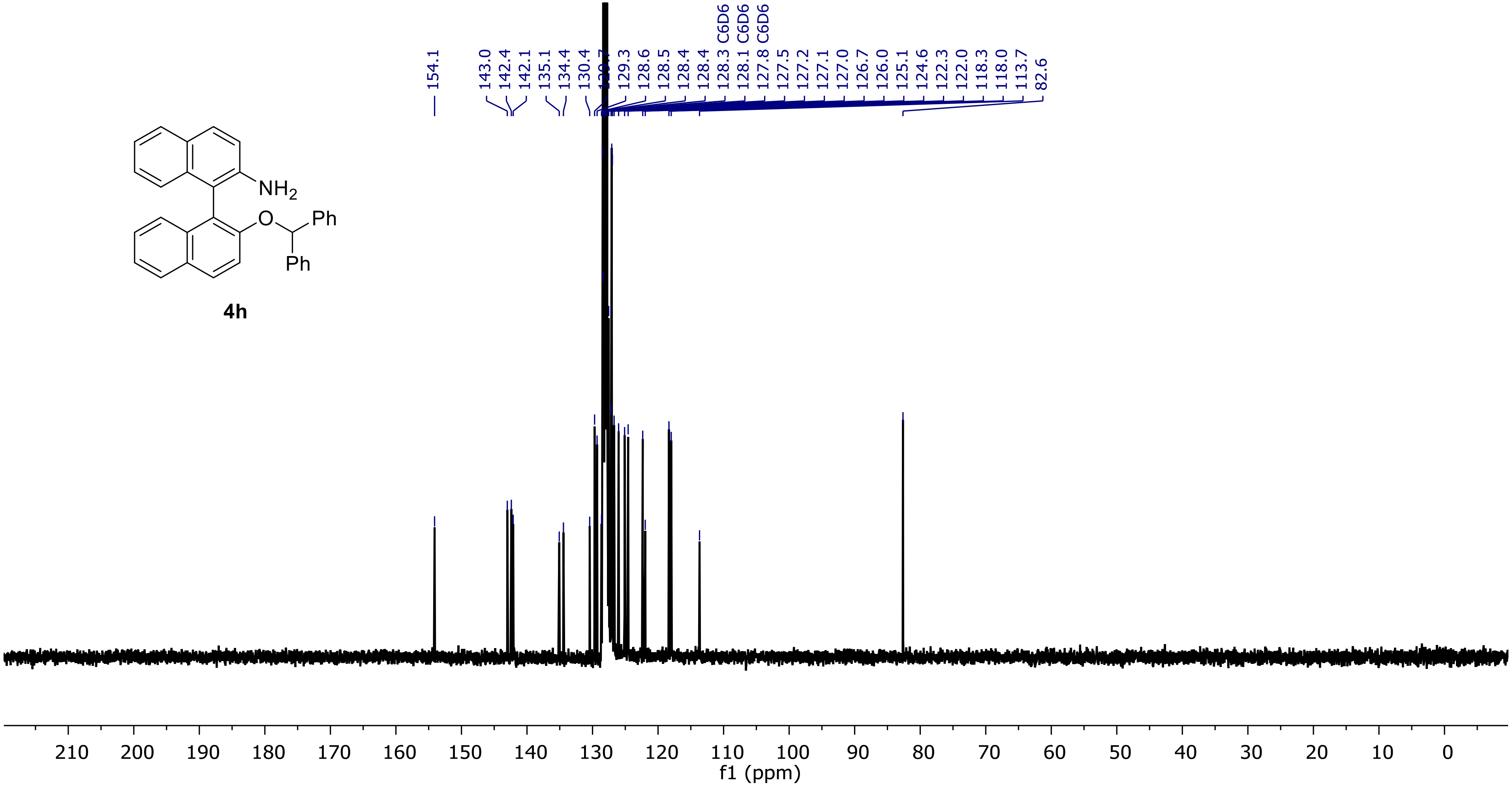

Figure S80. **^{1}H NMR** spectrum (500 MHz, DMSO-d_6, 298 K) of **S3**.

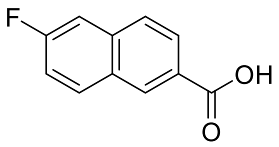

S3

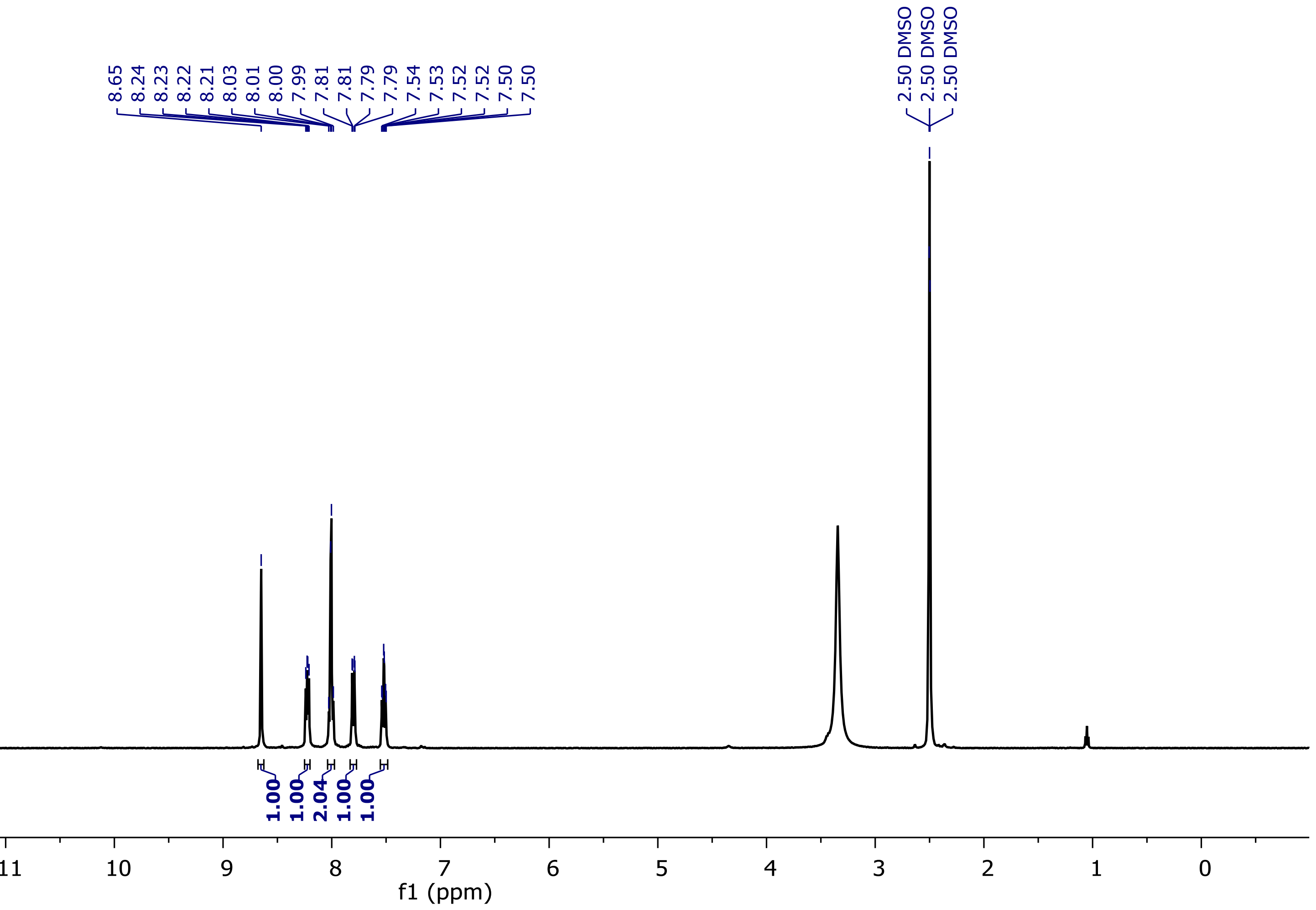

Figure S81. ^{19}**F NMR** spectrum (471 MHz, DMSO-d_6, 298 K) of **S3**.

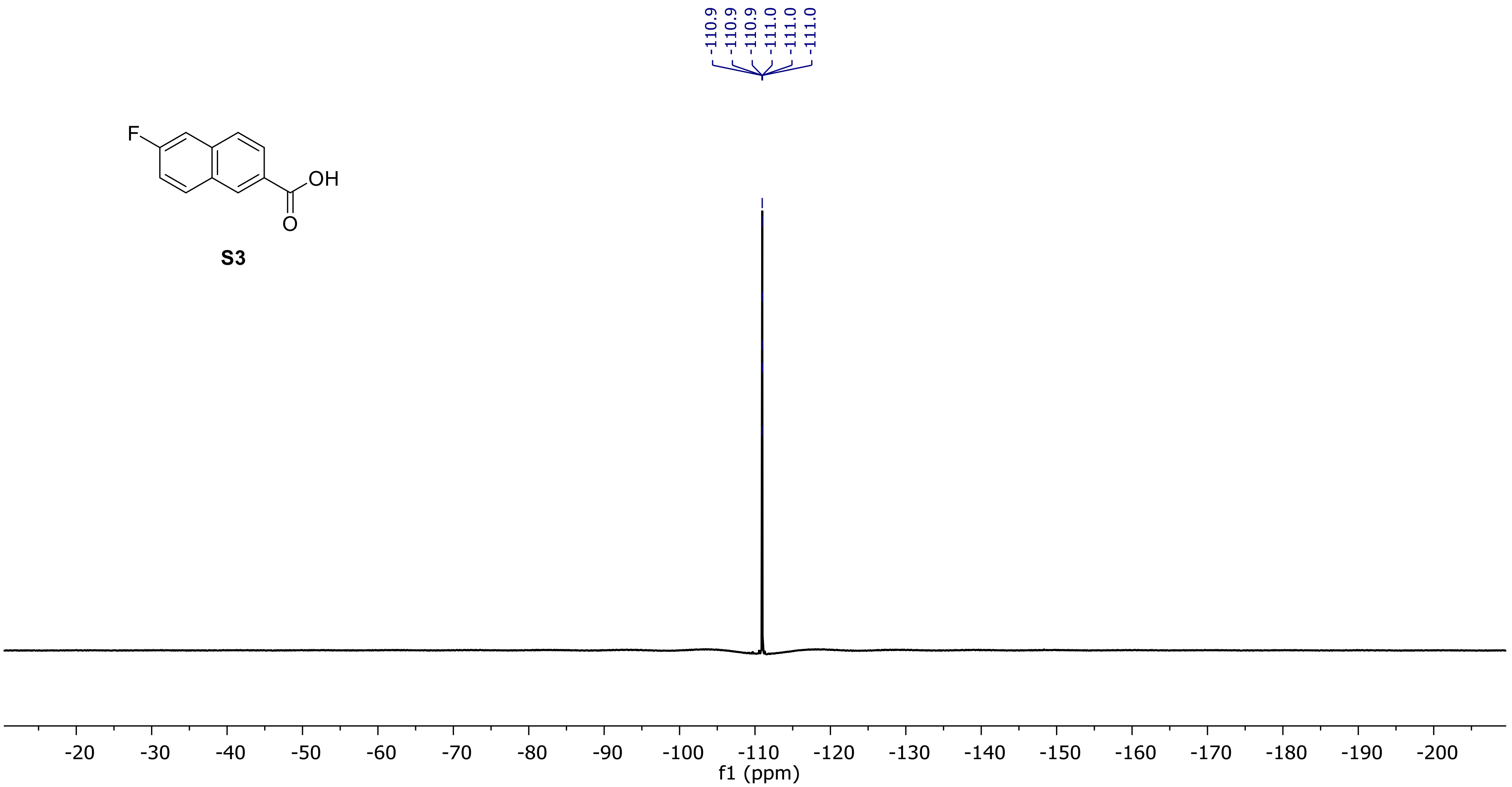

S3

Figure S82. **^{13}C{^{1}H} NMR** spectrum (101 MHz, DMSO-d_6, 298 K) of **S3**.

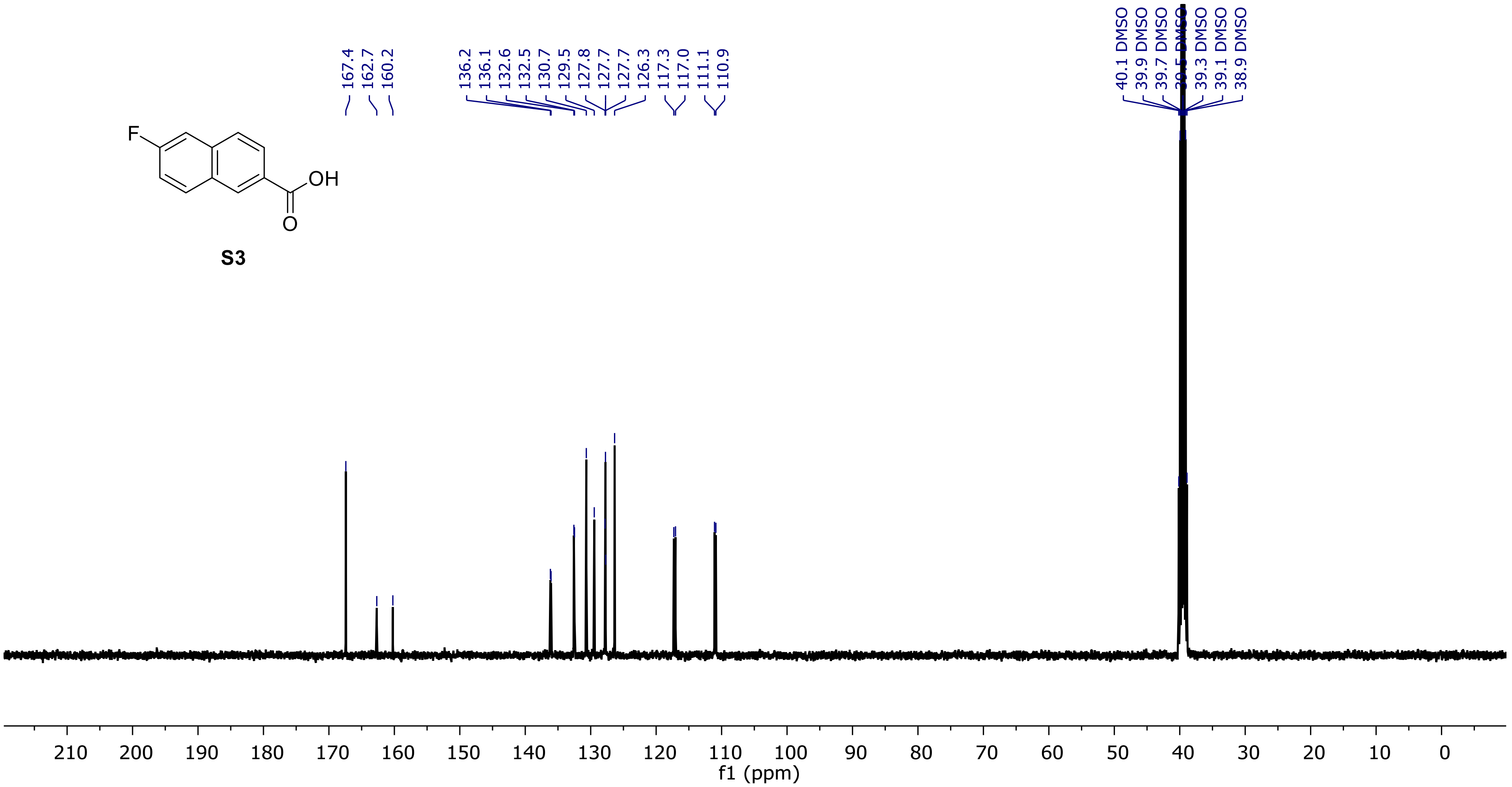

Figure S83. **^{1}H NMR** spectrum (500 MHz, $CDCl_3$, 298 K) of **S4**.

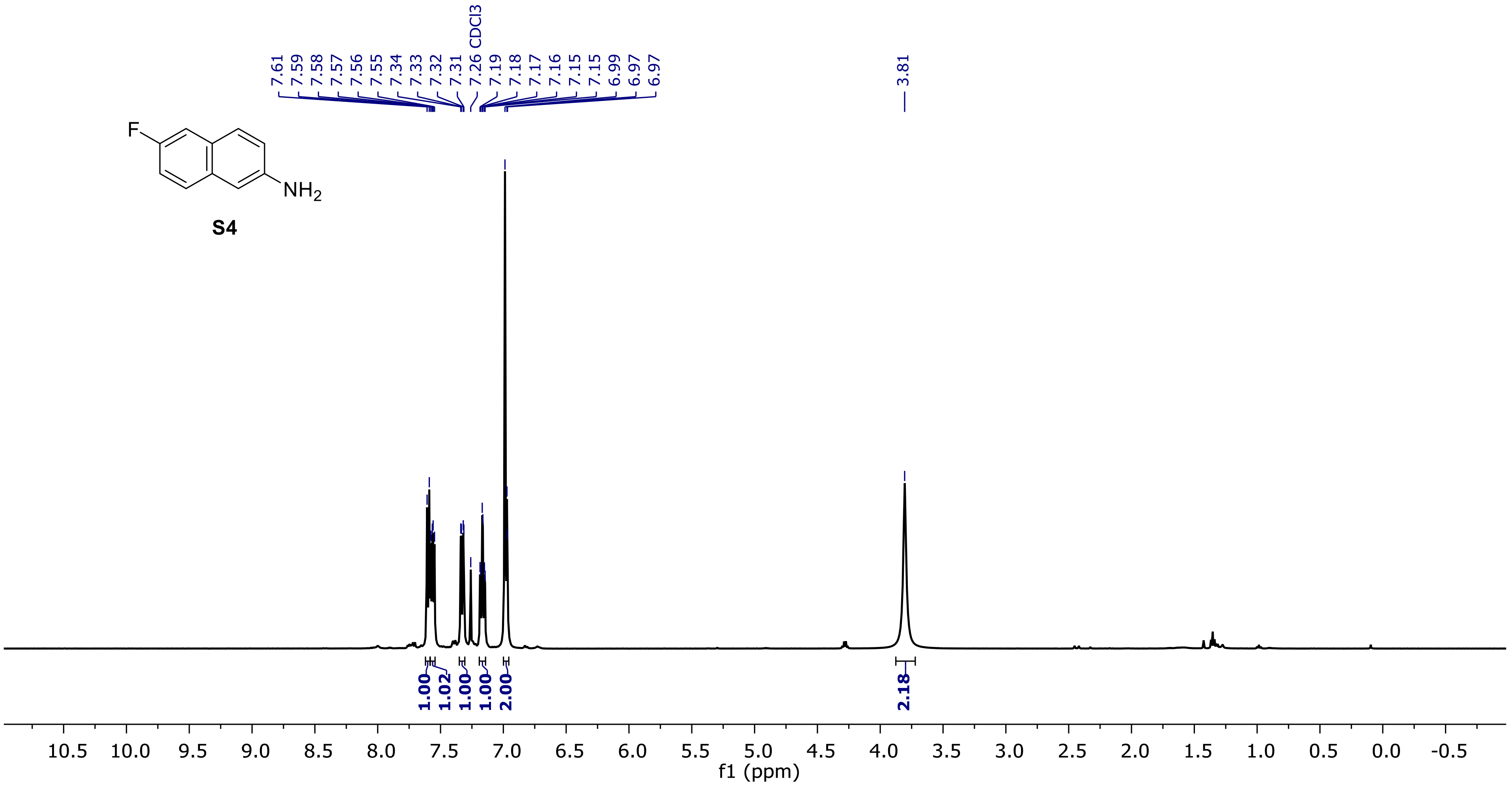

Figure S84. ^{19}**F NMR** spectrum (471 MHz, $CDCl_3$, 298 K) of **S4**.

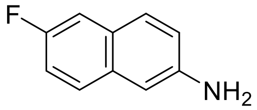

S4

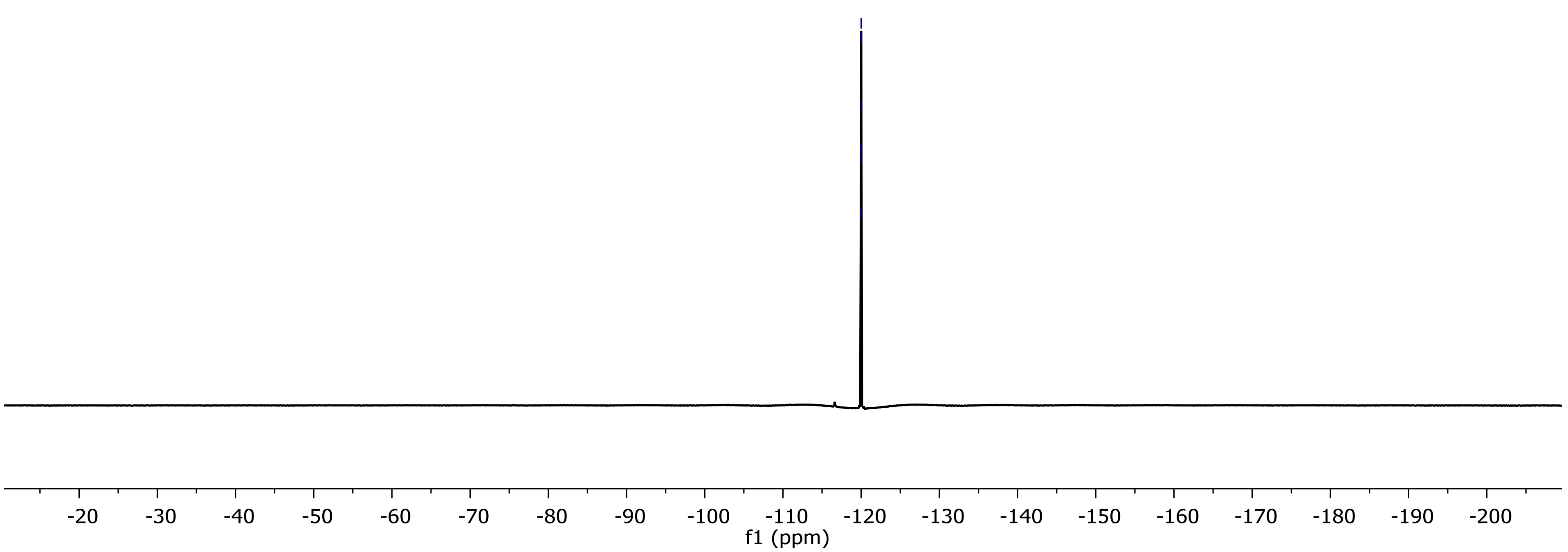

Figure S85. ^{13}**C{^{1}H} NMR** spectrum (126 MHz, $CDCl_3$, 298 K) of **S4**.

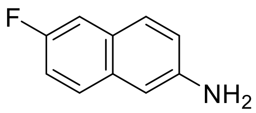

S4

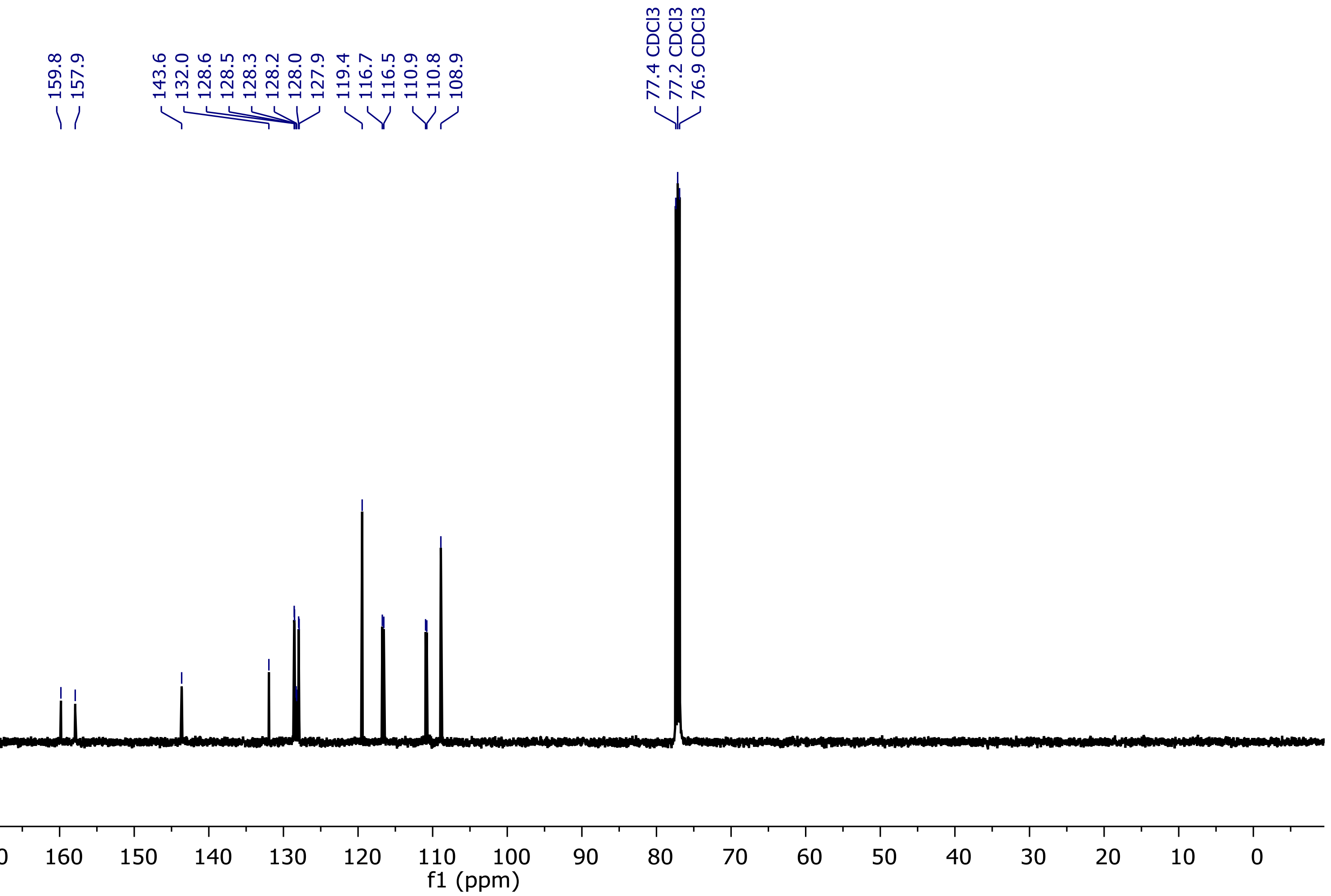

Figure S86. ^{1}**H NMR** spectrum (500 MHz, $CDCl_3$, 298 K) of **S5**.

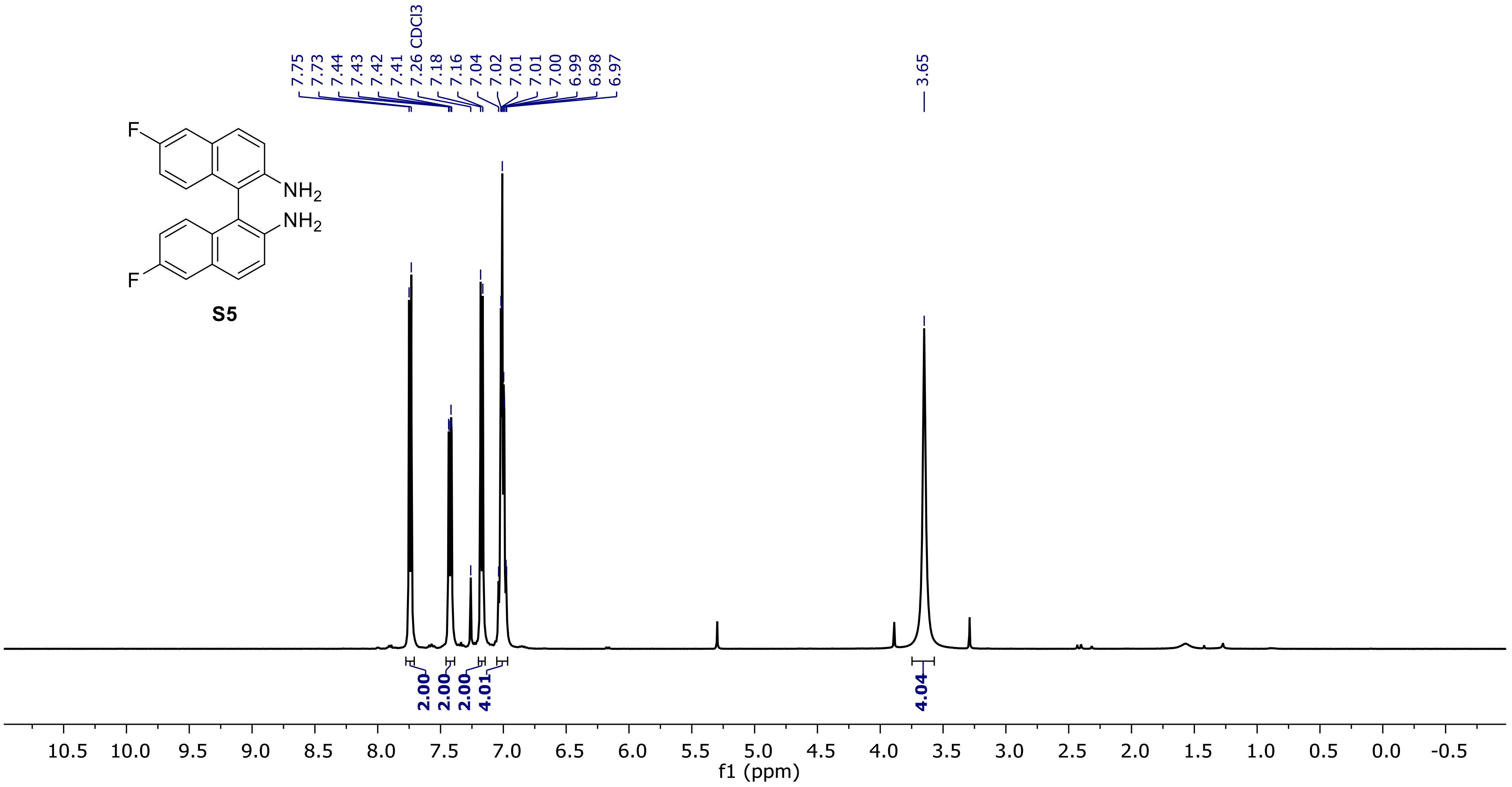

Figure S87. ^{19}**F NMR** spectrum (471 MHz, $CDCl_3$, 298 K) of **S5**.

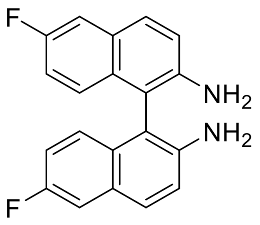

S5

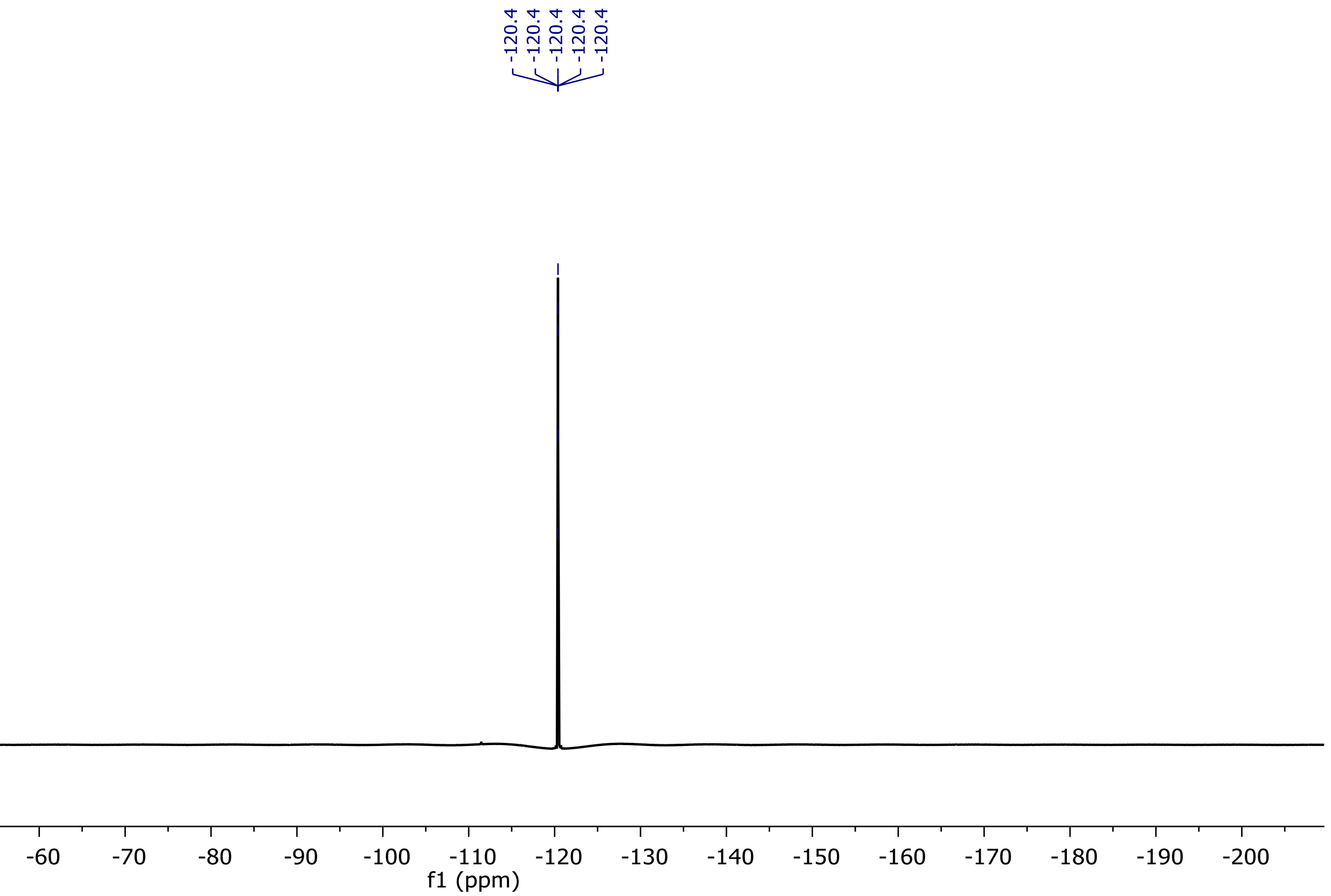

Figure S88. ^{13}**C{^{1}H} NMR** spectrum (126 MHz, $CDCl_3$, 298 K) of **S5**.

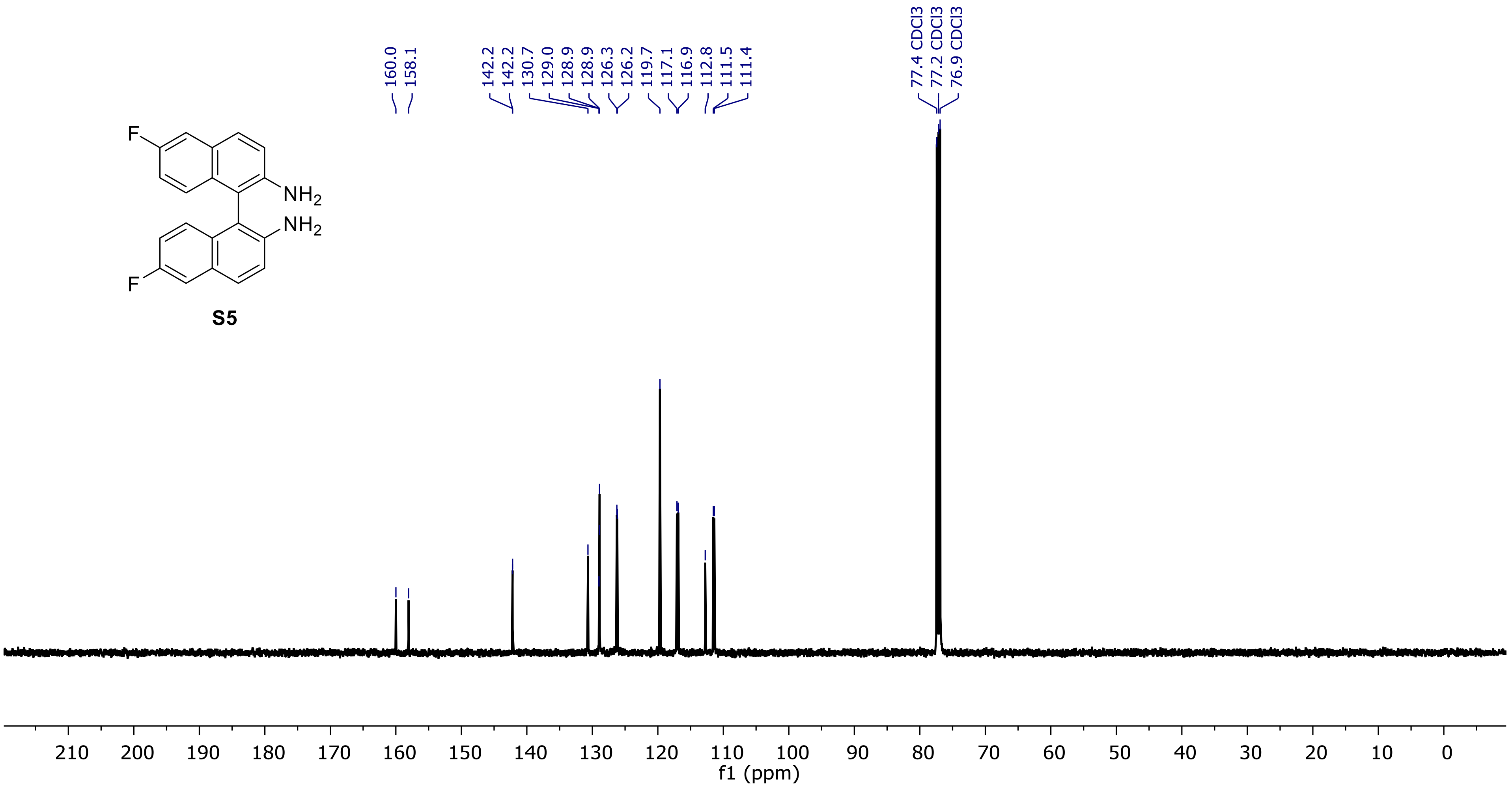

Figure S89. [1]**H NMR** spectrum (500 MHz, C_6D_6, 298 K) of **4i**.

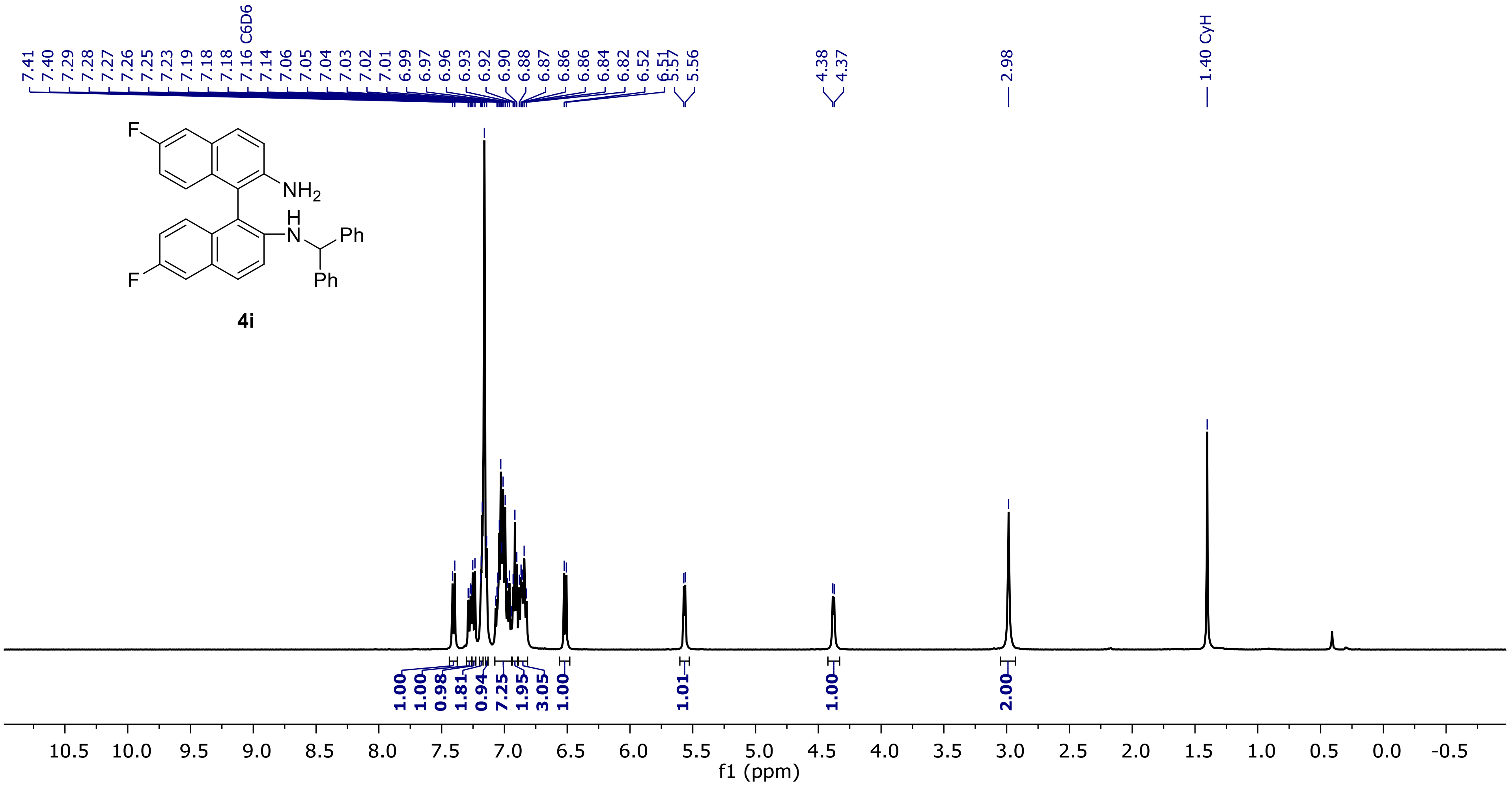

Figure S90. ^{19}F NMR spectrum (471 MHz, C_6D_6, 298 K) of **4i**.

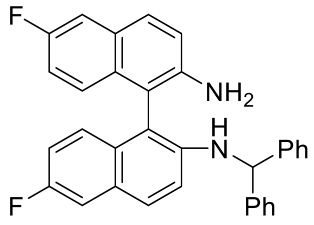

4i

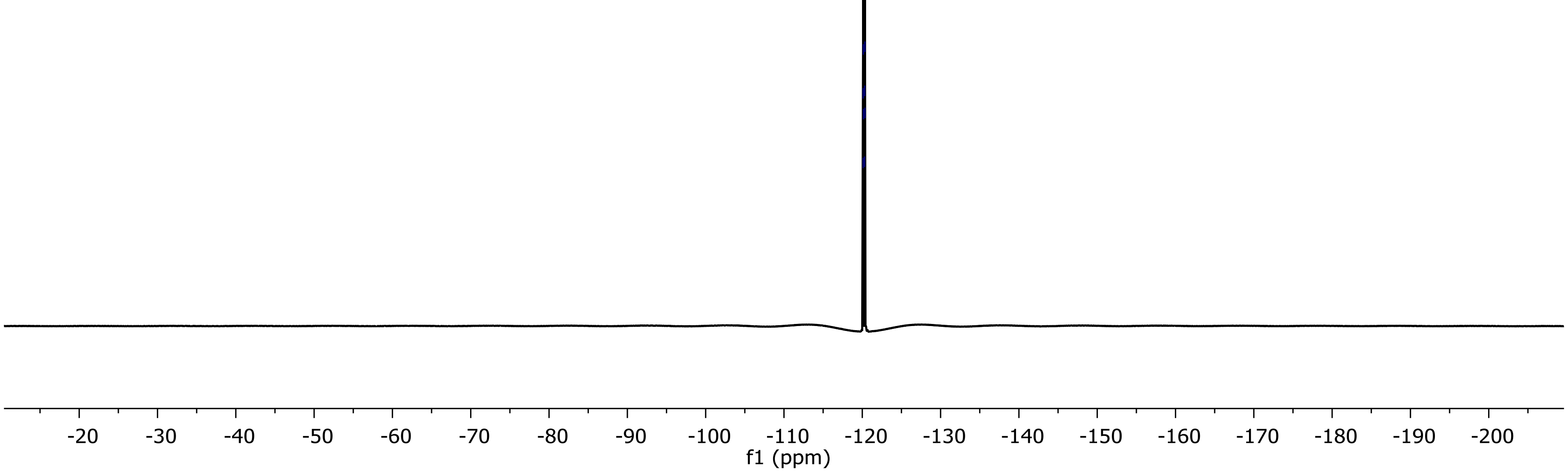

Figure S91. $^{13}C\{^1H\}$ NMR spectrum (126 MHz, C_6D_6, 298 K) of **4i**.

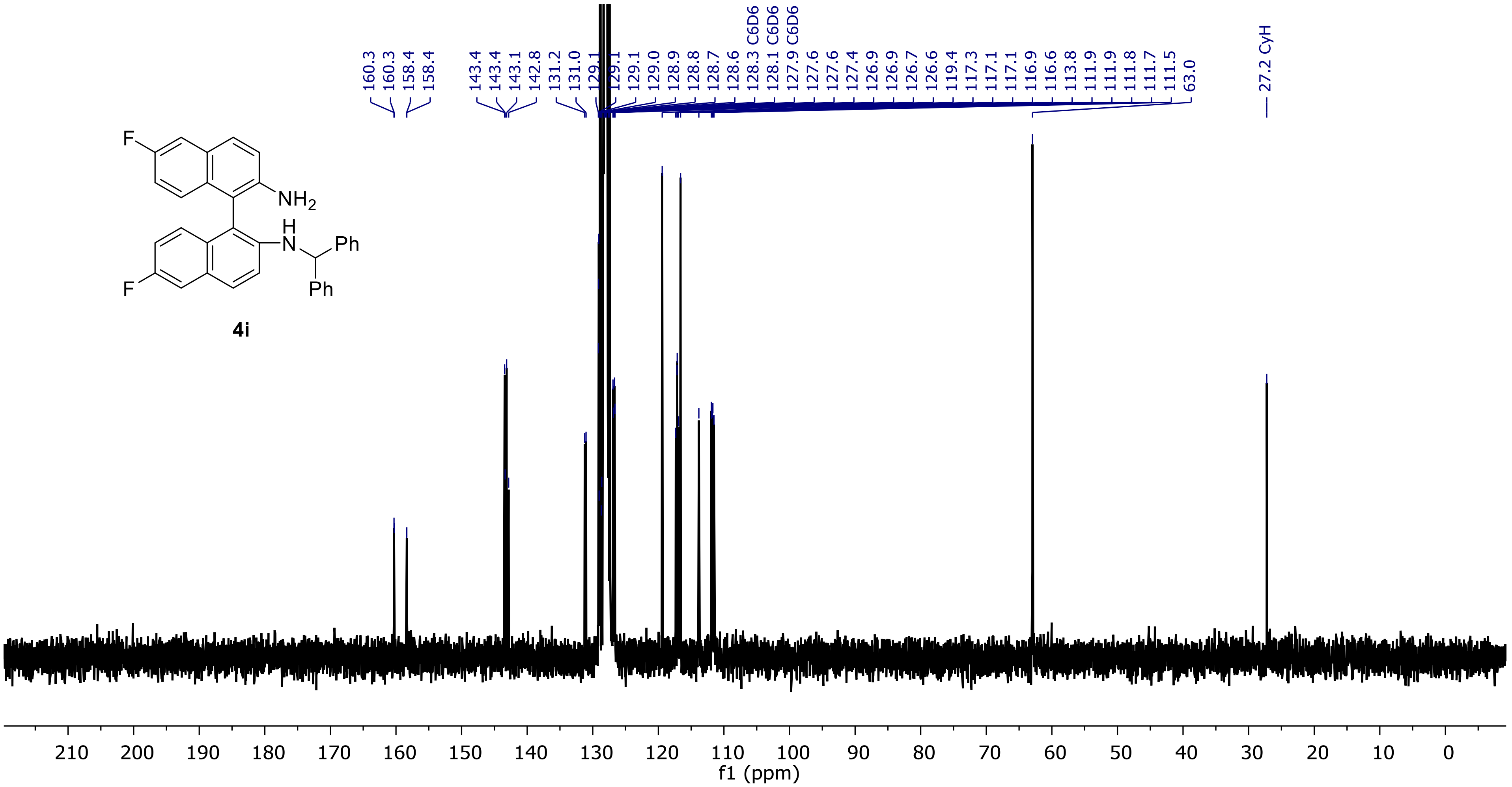

4i

Figure S92. ^{1}**H NMR** spectrum (400 MHz, $CDCl_3$, 298 K) of **S6**.

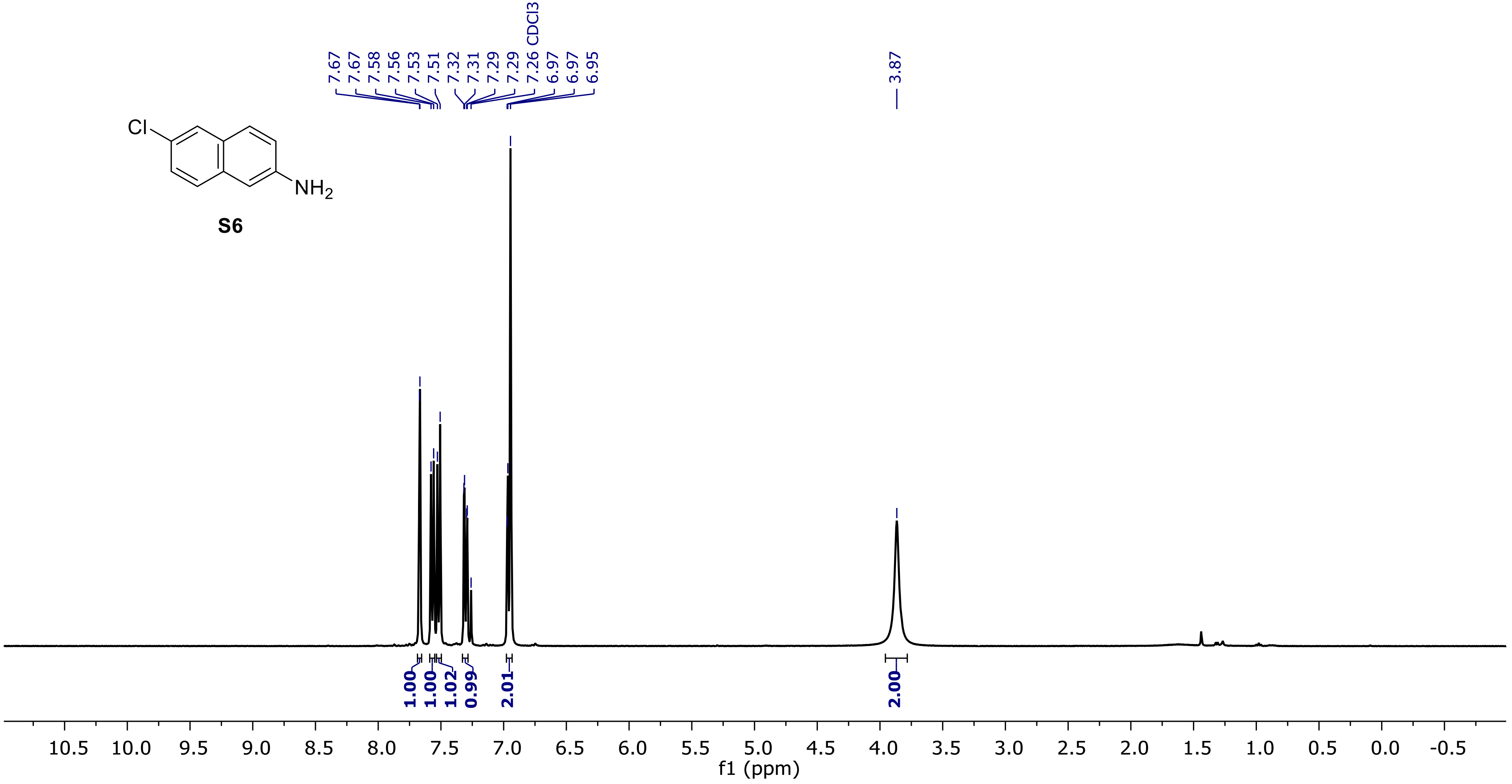

Figure S93. ^{13}**C{^{1}H} NMR** spectrum (126 MHz, $CDCl_3$, 298 K) of **S6**.

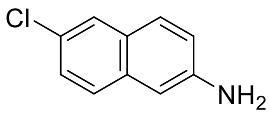

S6

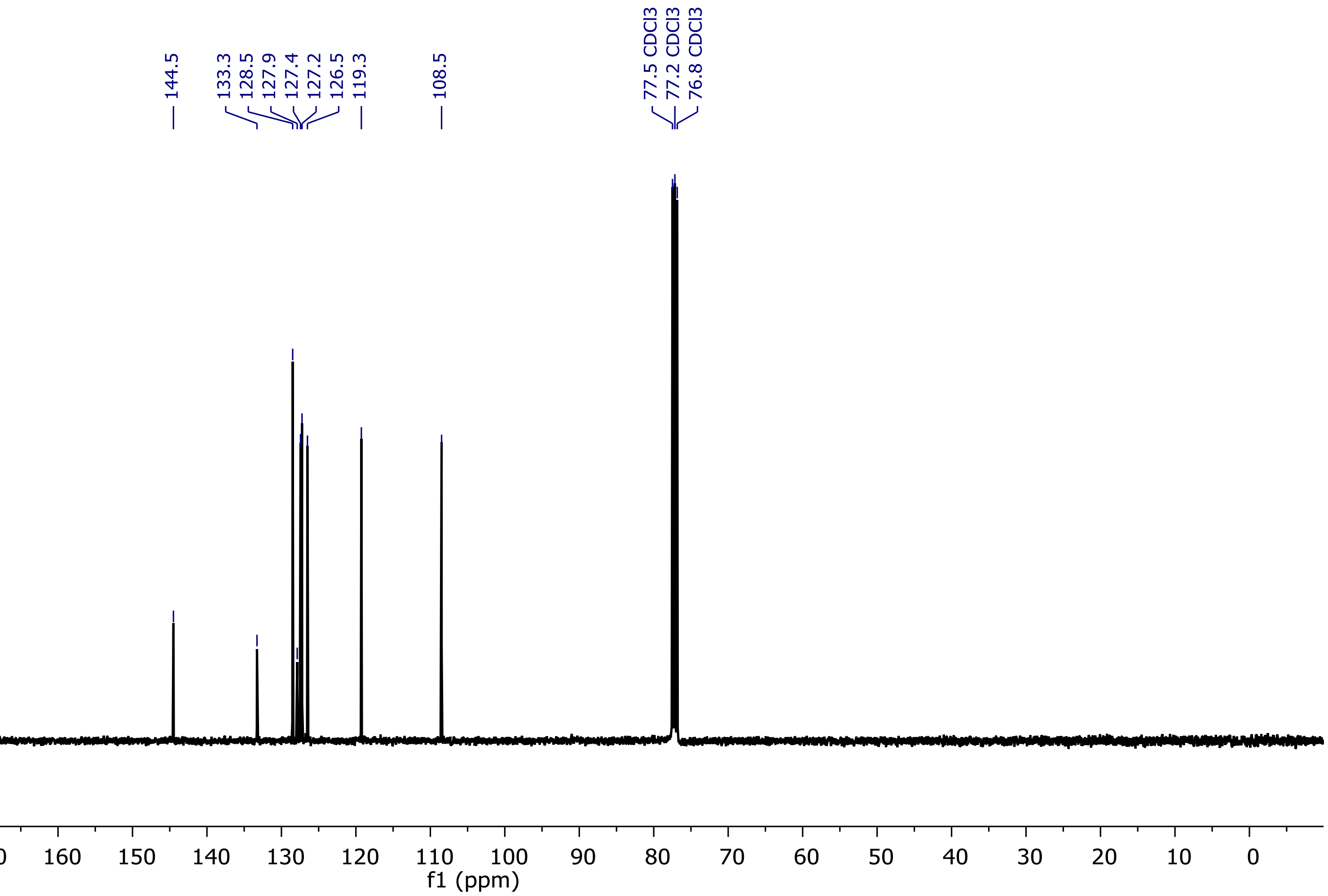

Figure S94. **¹H NMR** spectrum (400 MHz, $CDCl_3$, 298 K) of **S7**.

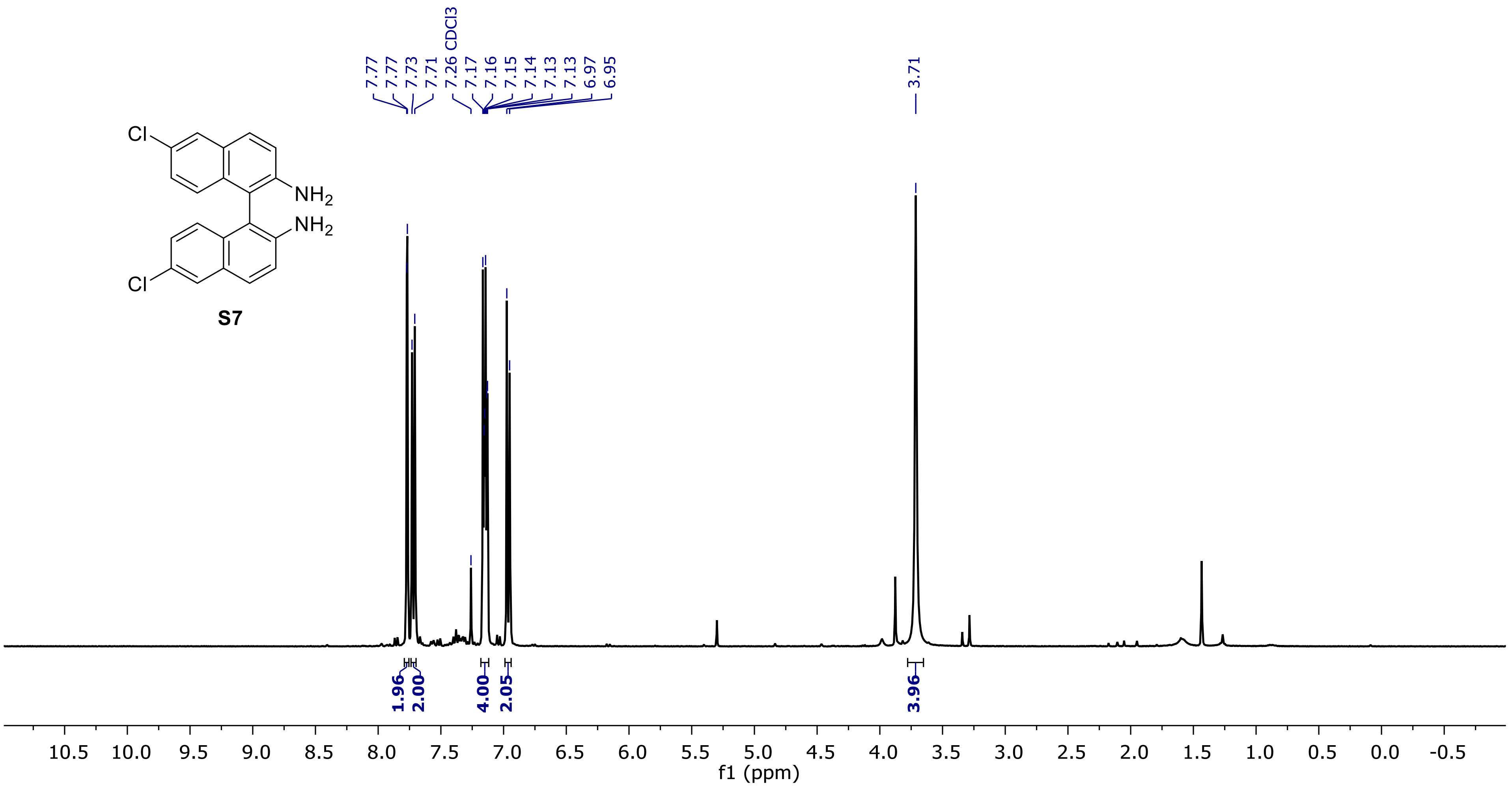

Figure S95. ^{13}**C{**^{1}**H} NMR** spectrum (101 MHz, $CDCl_3$, 298 K) of **S7**.

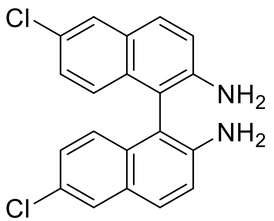

S7

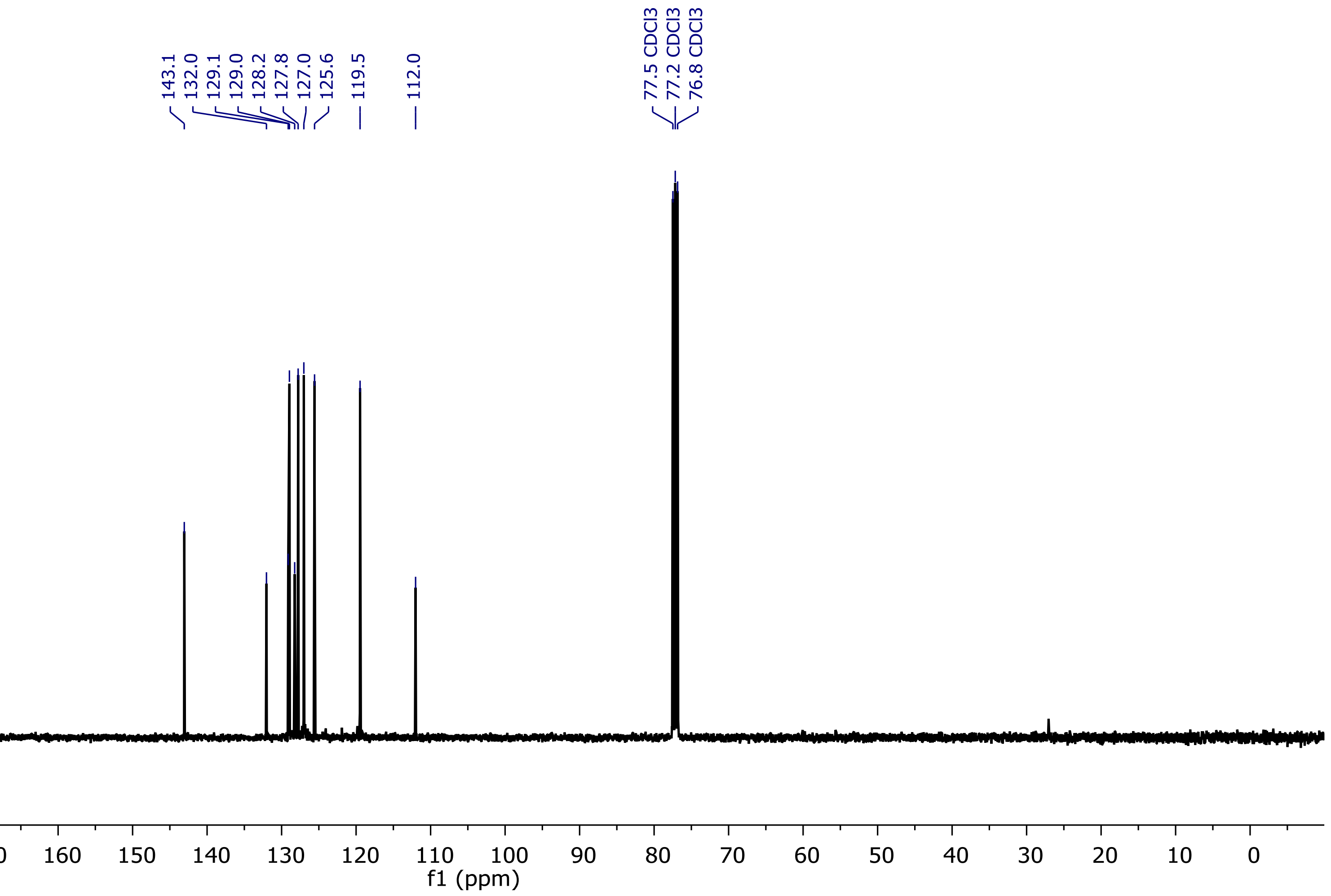

Figure S96. **^{1}H NMR** spectrum (500 MHz, C_6D_6, 298 K) of **4j**.

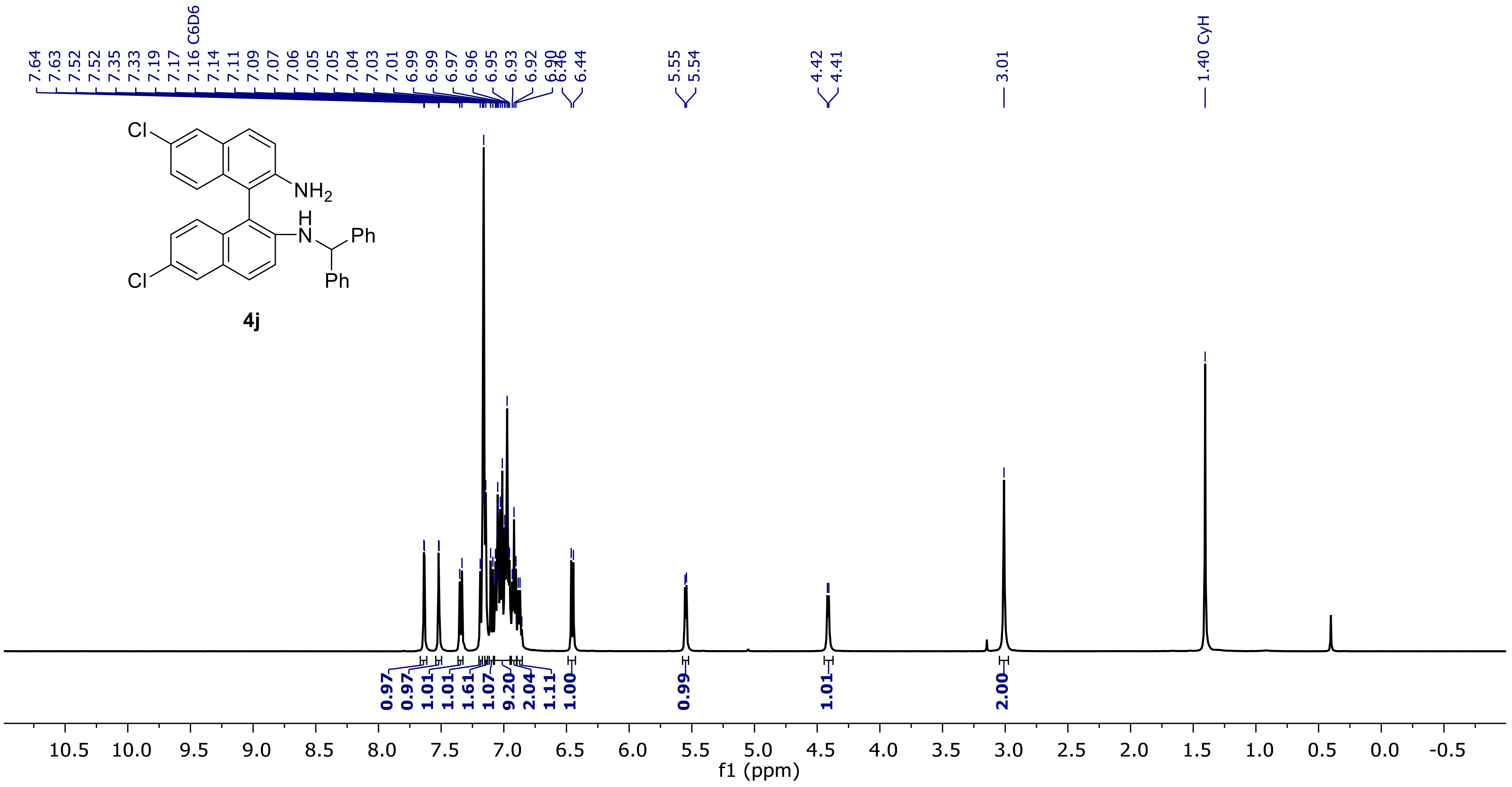

Figure S97. ^{13}**C{^{1}H} NMR** spectrum (126 MHz, C_6D_6, 298 K) of **4j**.

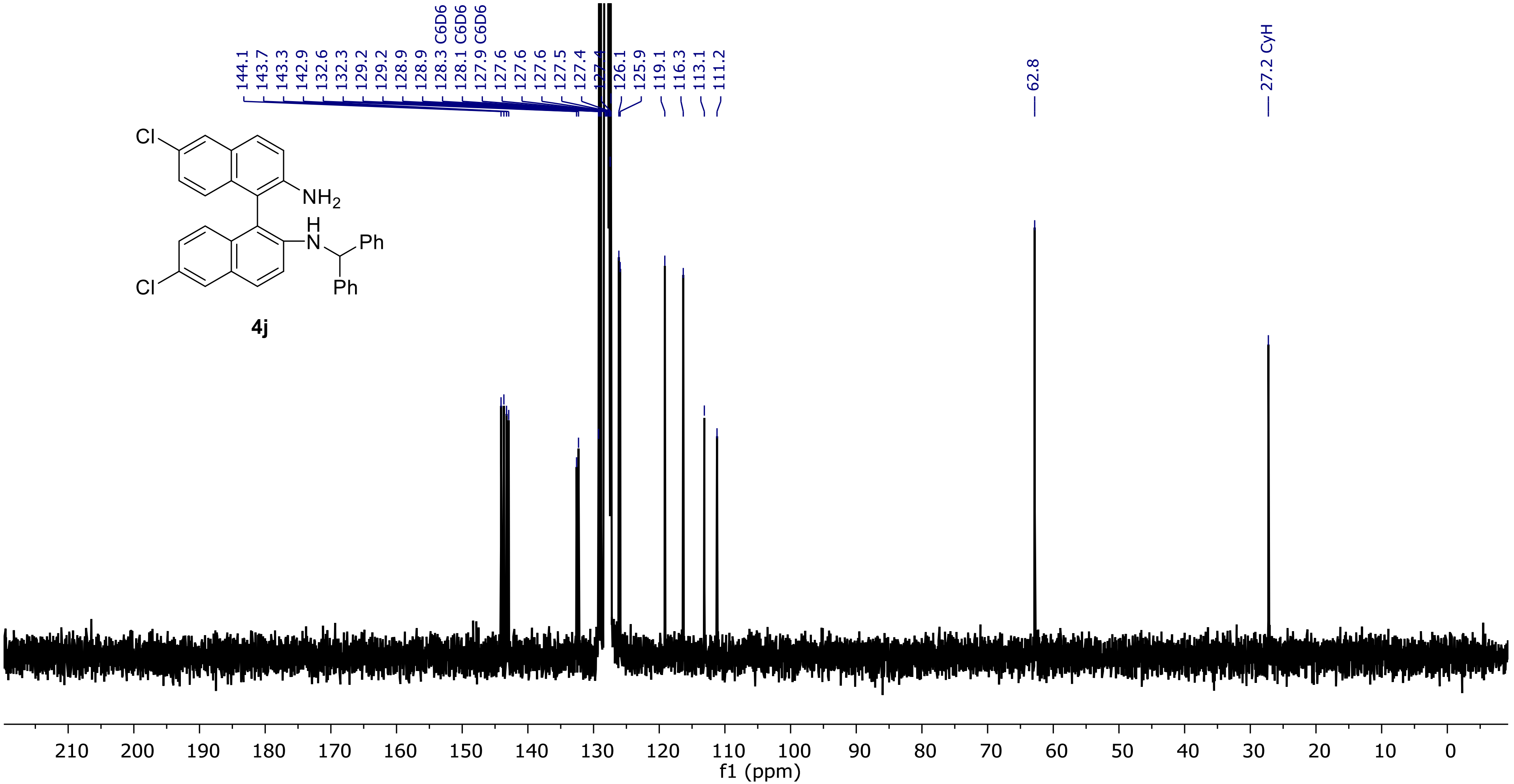

Figure S98. **^{1}H NMR** spectrum (500 MHz, C_6D_6, 298 K) of **4k**.

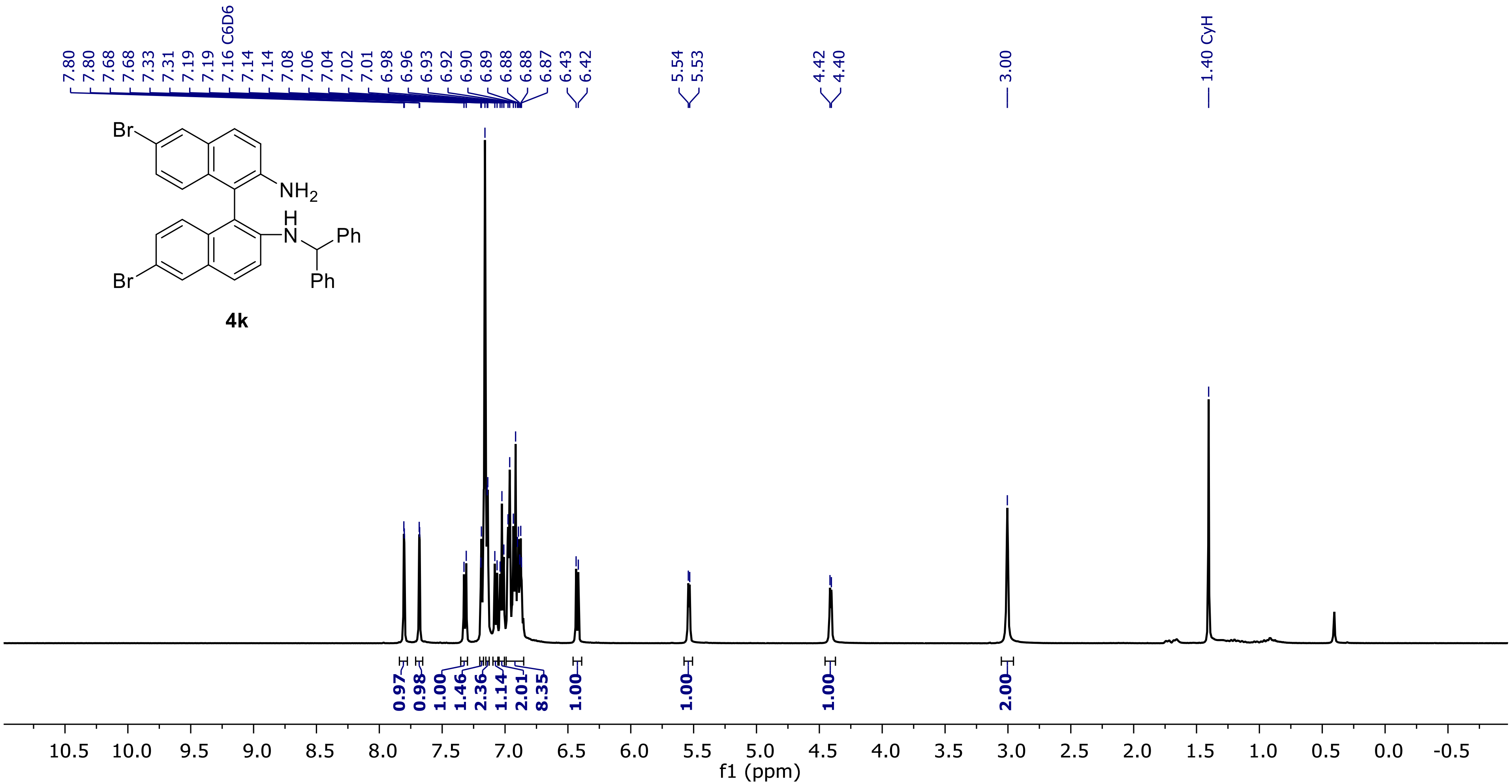

Figure S99. **$^{13}C\{^1H\}$ NMR** spectrum (126 MHz, C_6D_6, 298 K) of **4k**.

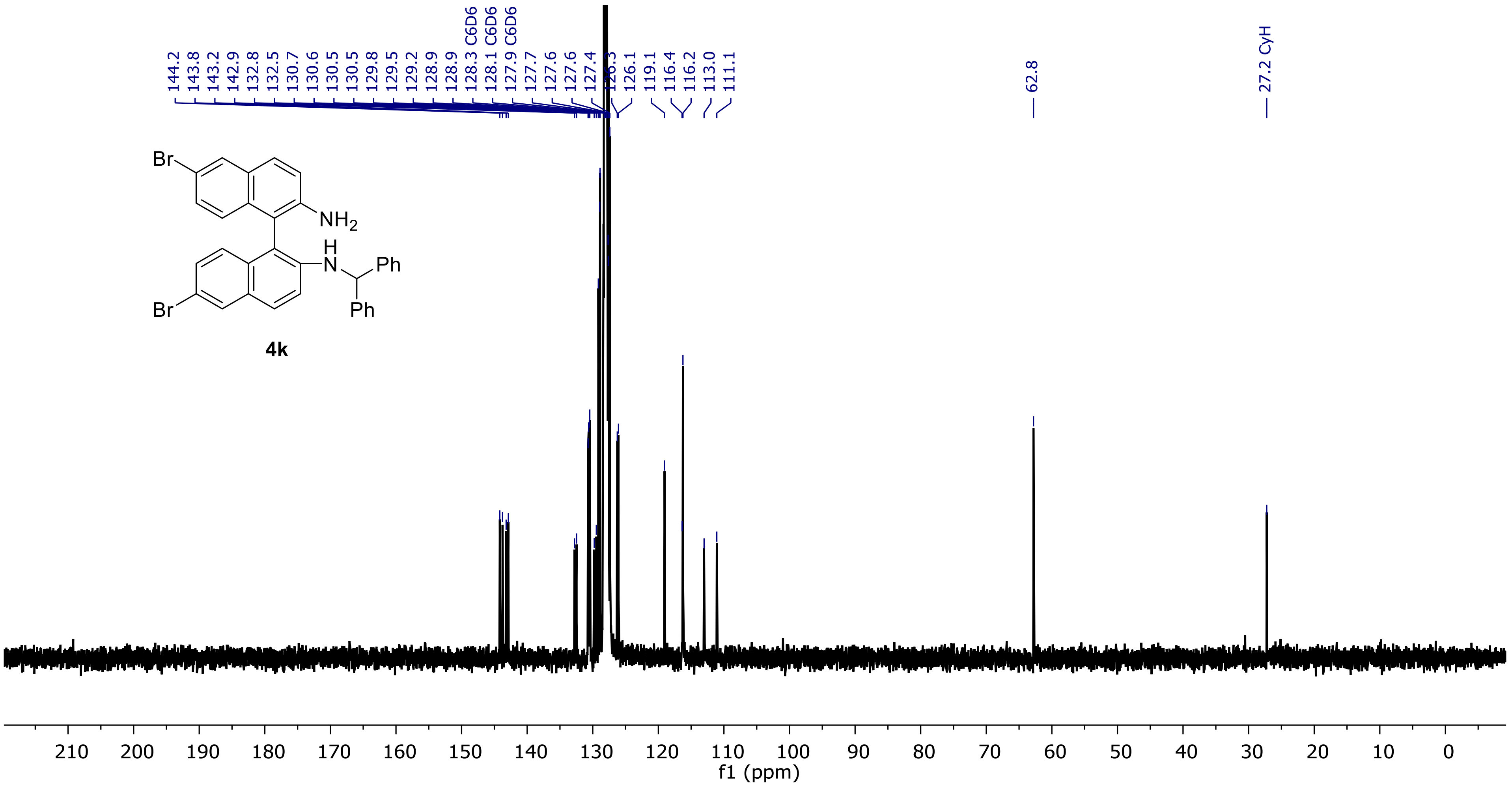

Figure S100. **¹H NMR** spectrum (500 MHz, C_6D_6, 298 K) of **4l**.

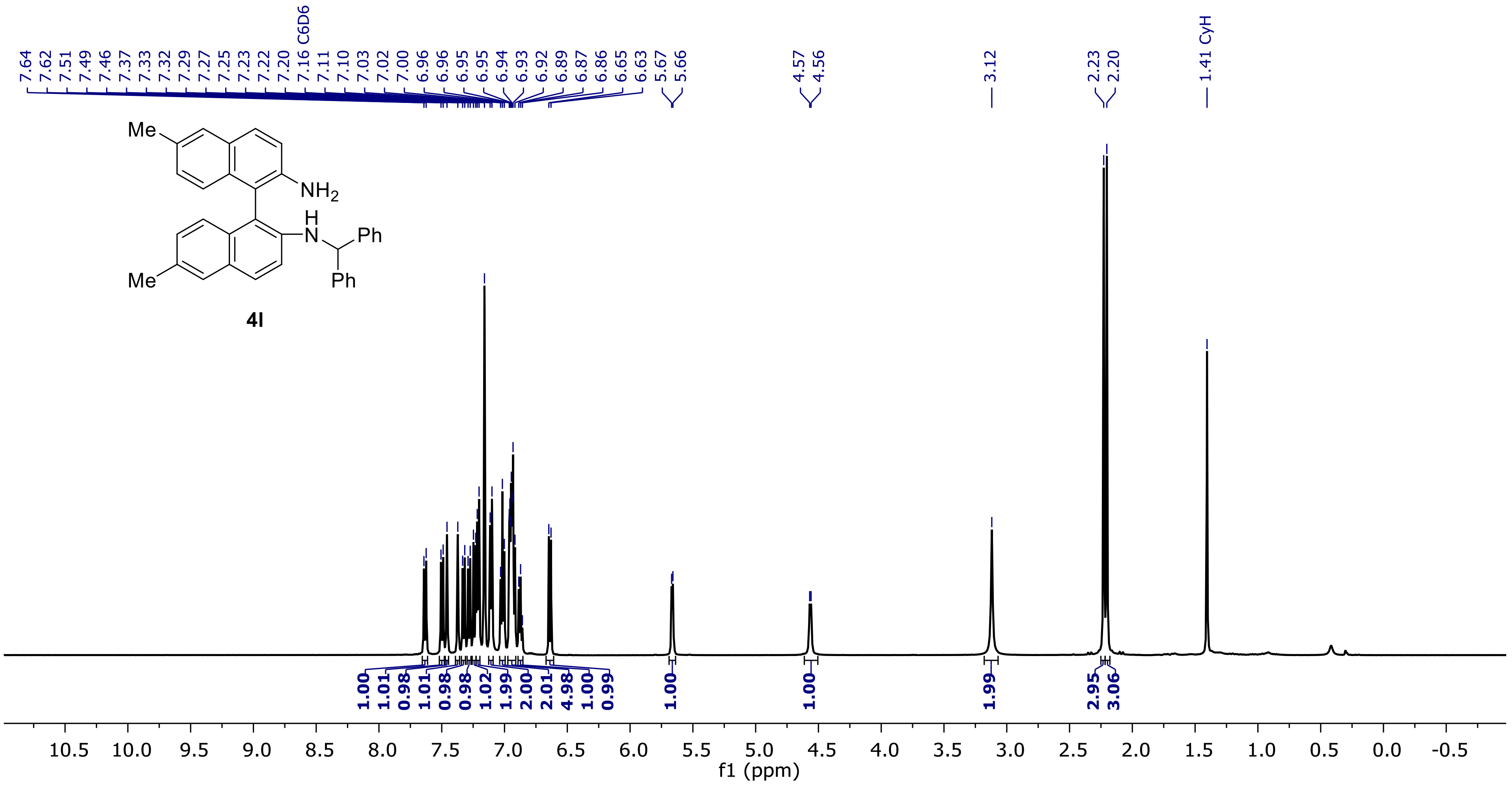

Figure S101. $^{13}C\{^1H\}$ **NMR** spectrum (126 MHz, C_6D_6, 298 K) of **4l**.

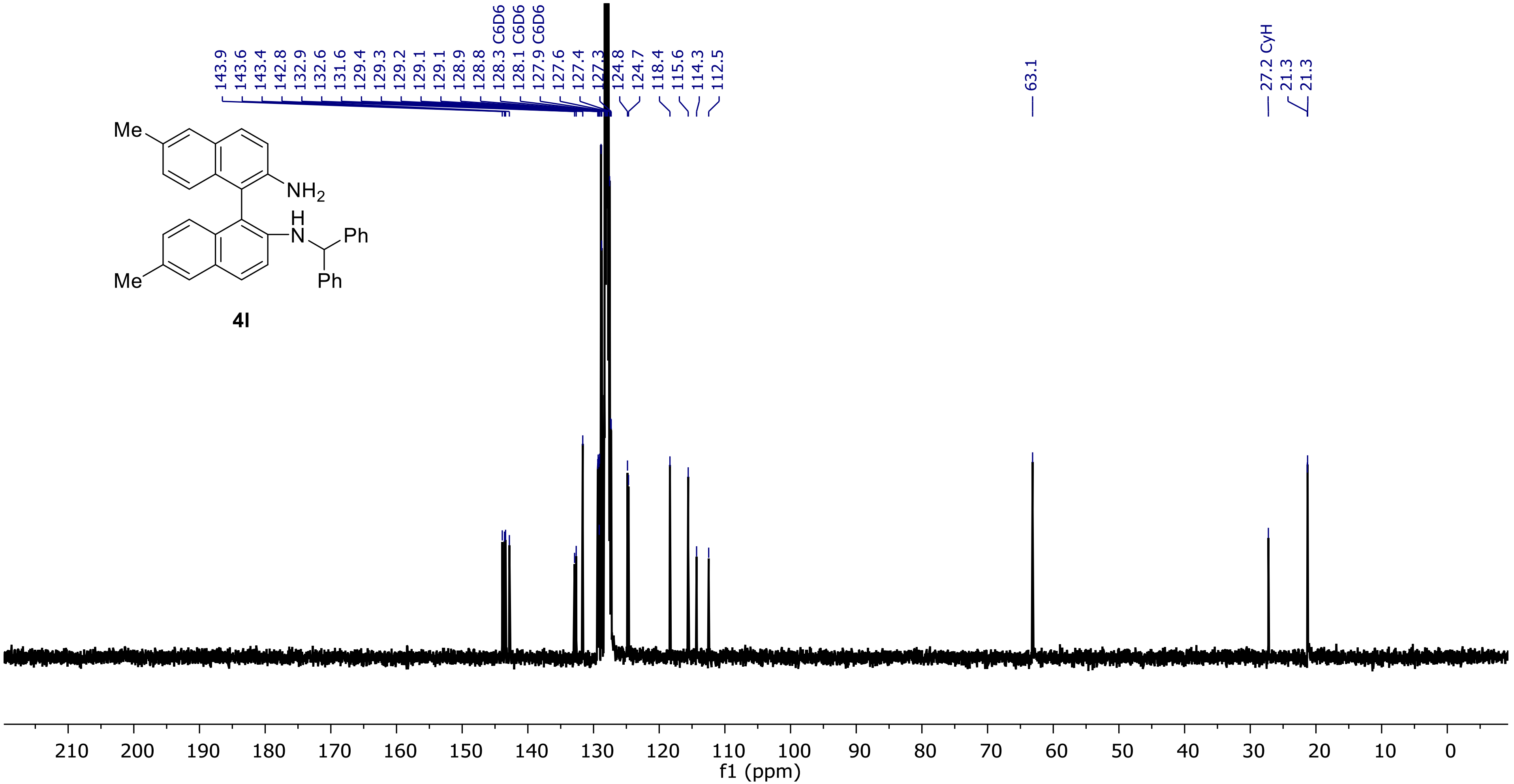

Figure S102. **^{1}H NMR** spectrum (400 MHz, $CDCl_3$, 298 K) of **S11**.

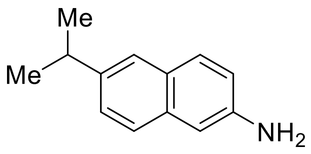

S11

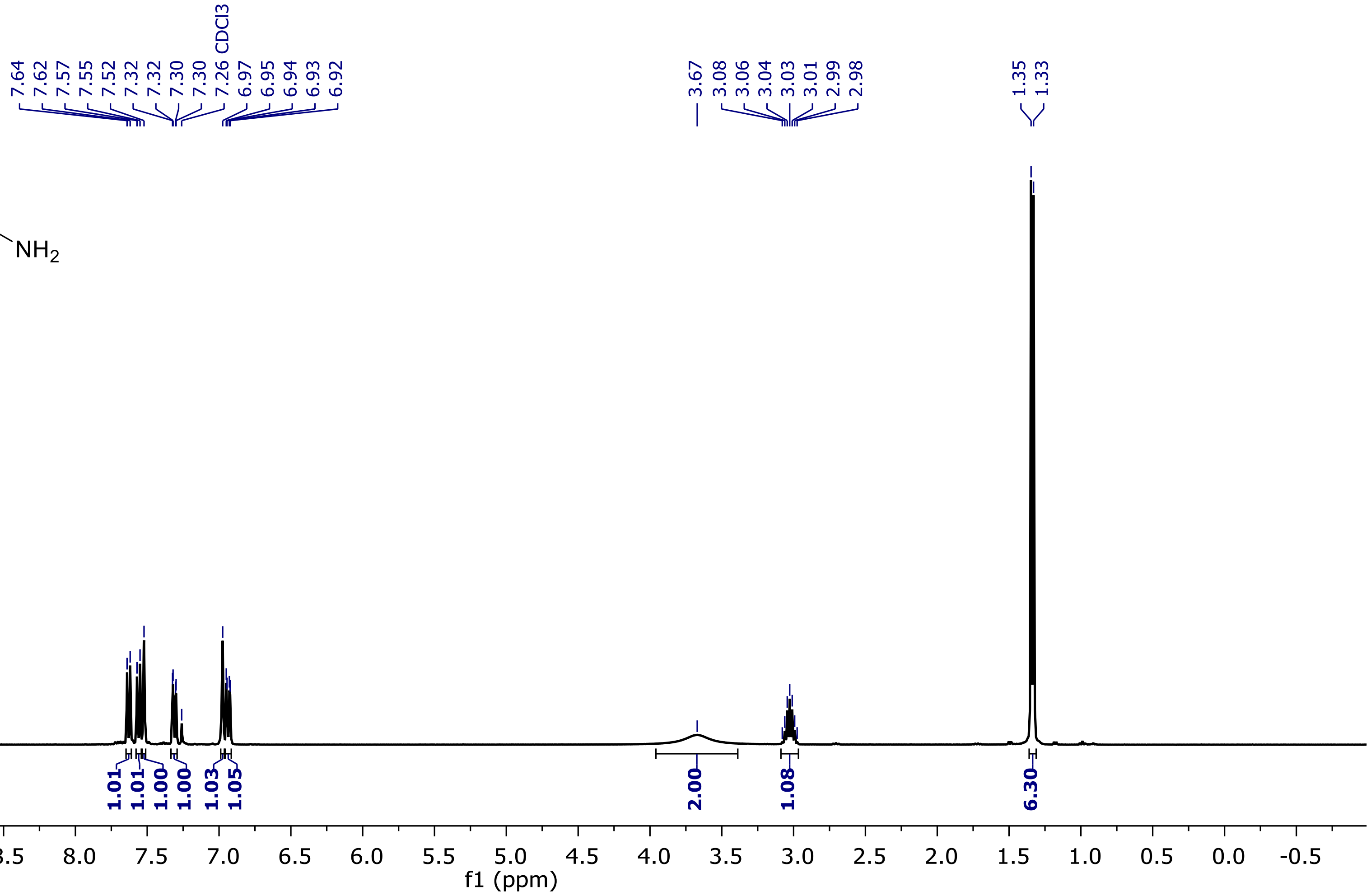

Figure S103. **$^{13}C\{^{1}H\}$ NMR** spectrum (101 MHz, $CDCl_3$, 298 K) of **S11**.

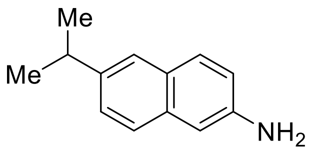

S11

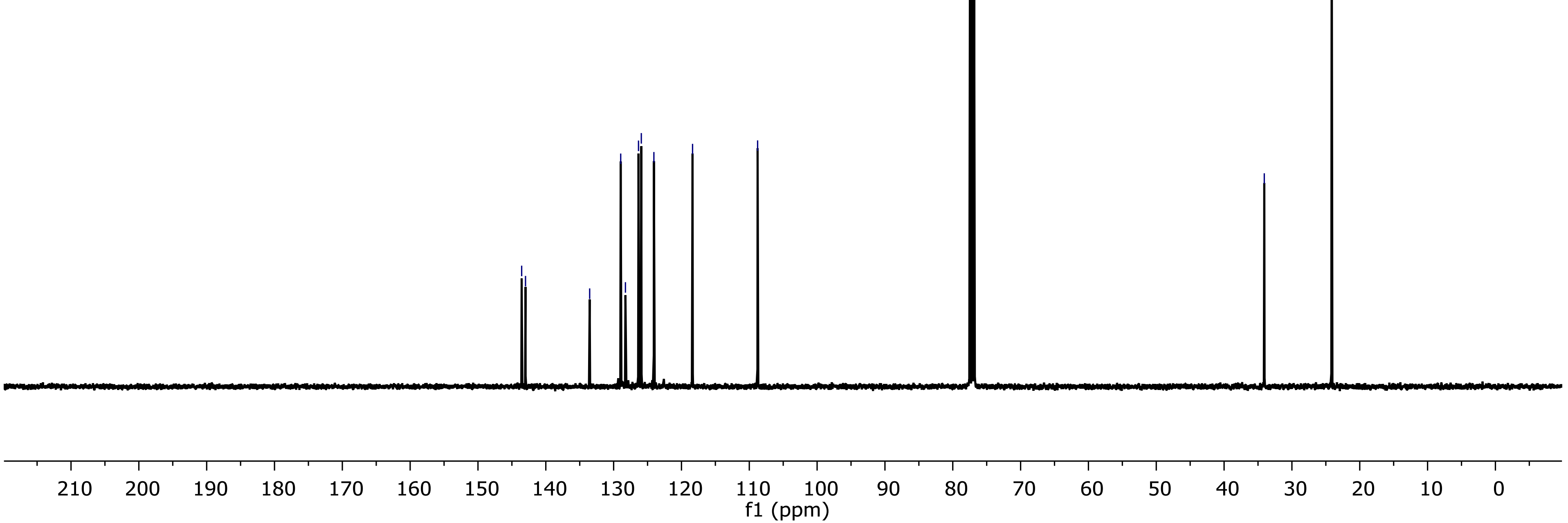

Figure S104. **¹H NMR** spectrum (500 MHz, $CDCl_3$, 298 K) of **S12**.

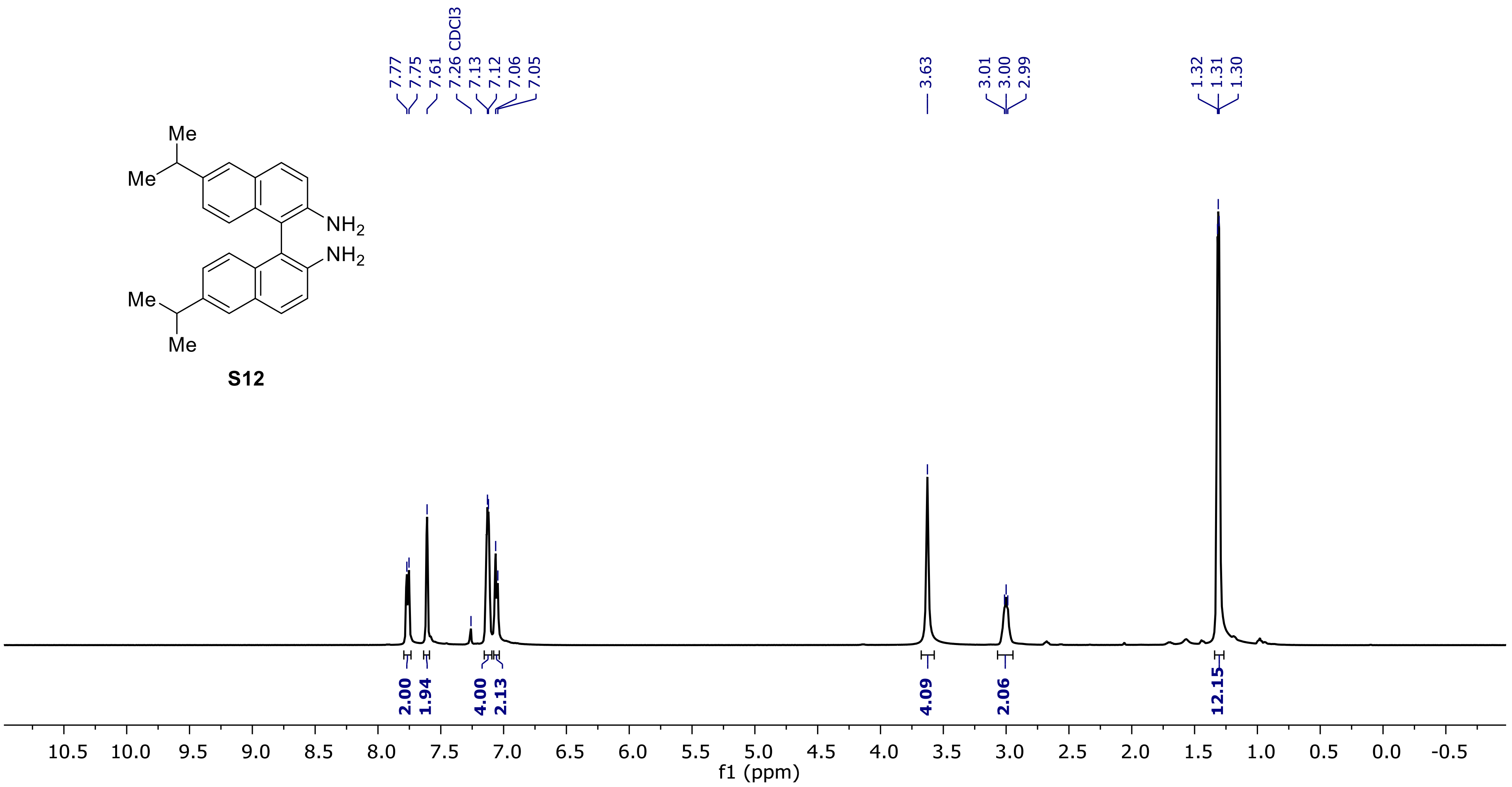

Figure S105. $^{13}C\{^1H\}$ **NMR** spectrum (126 MHz, $CDCl_3$, 298 K) of **S12**.

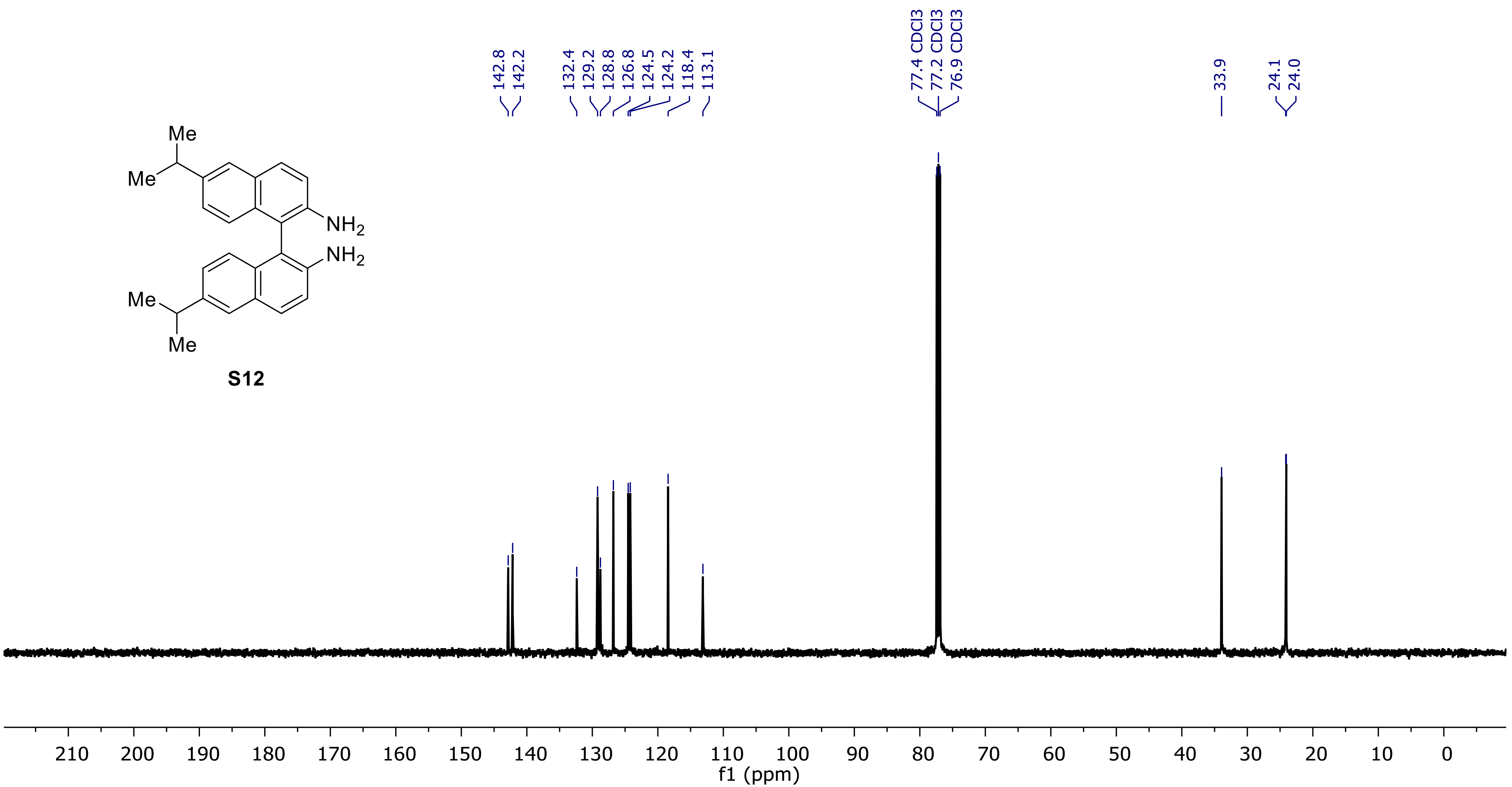

Figure S106. **^{1}H NMR** spectrum (500 MHz, C_6D_6, 298 K) of **4m**.

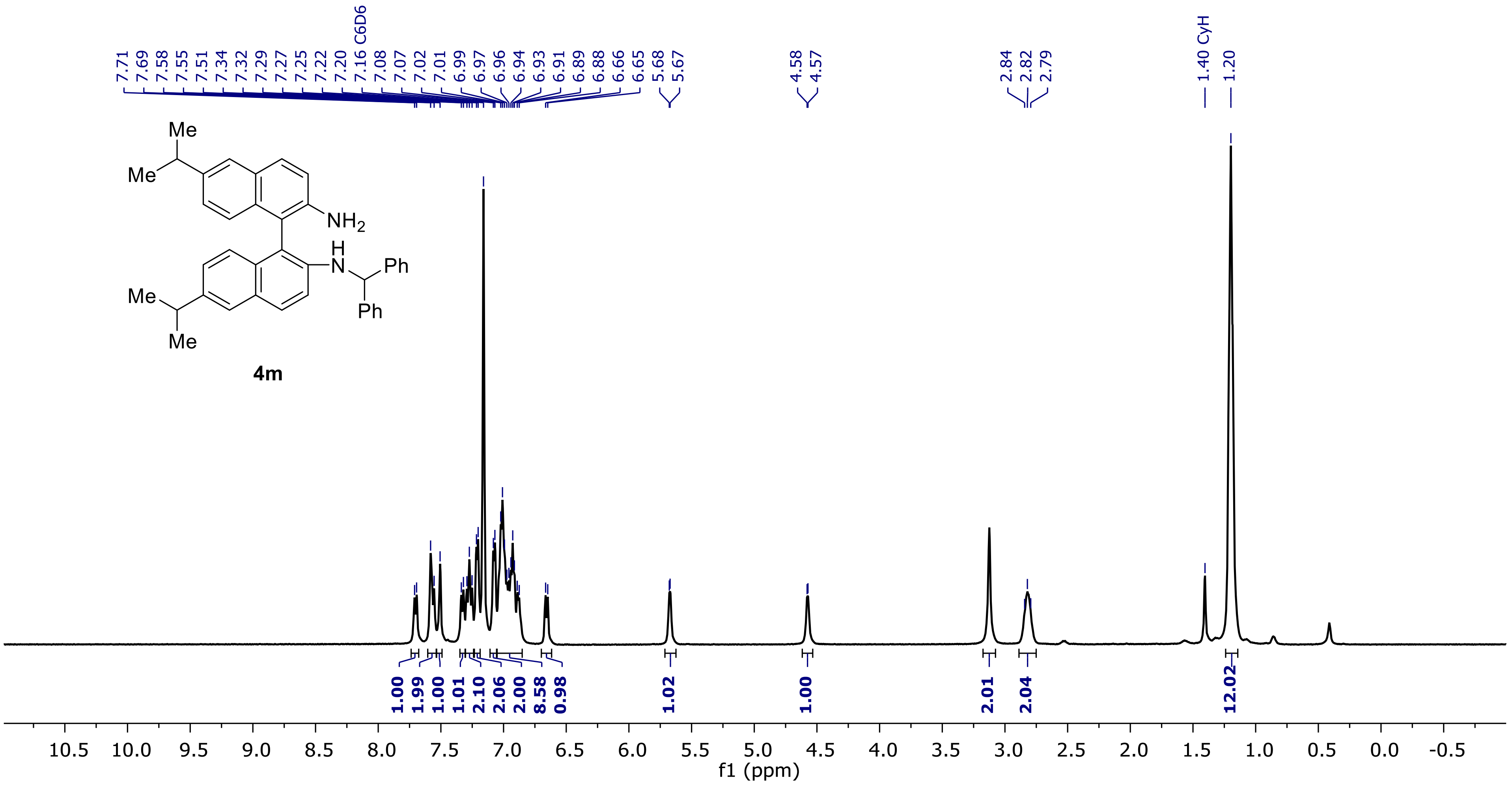

Figure S107. ^{13}**C{^{1}H} NMR** spectrum (126 MHz, C_6D_6, 298 K) of **4m**.

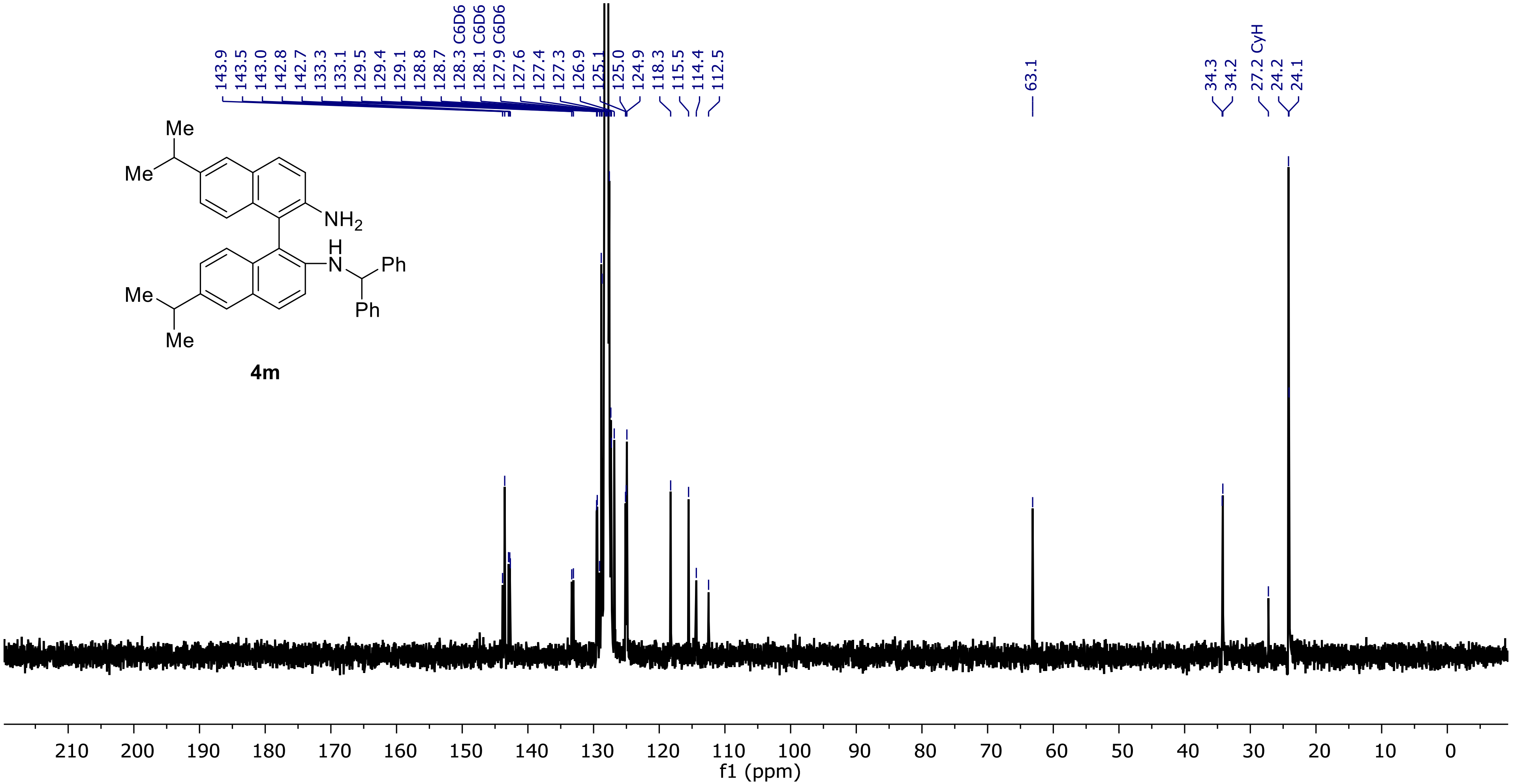

Figure S108. **^{1}H NMR** spectrum (500 MHz, C_6D_6, 298 K) of **4n**.

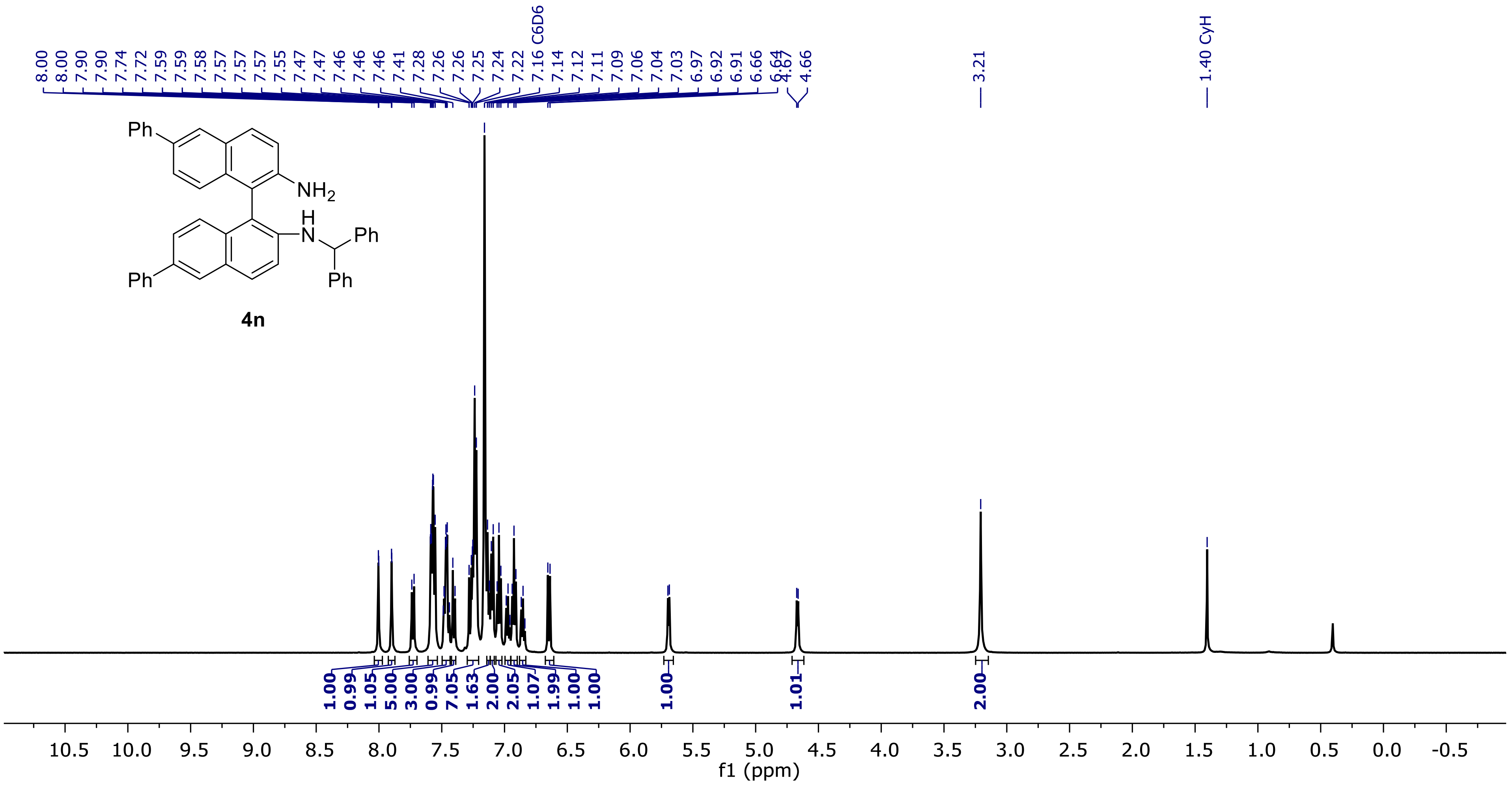

Figure S109. $^{13}C\{^1H\}$ **NMR** spectrum (126 MHz, C_6D_6, 298 K) of **4n**.

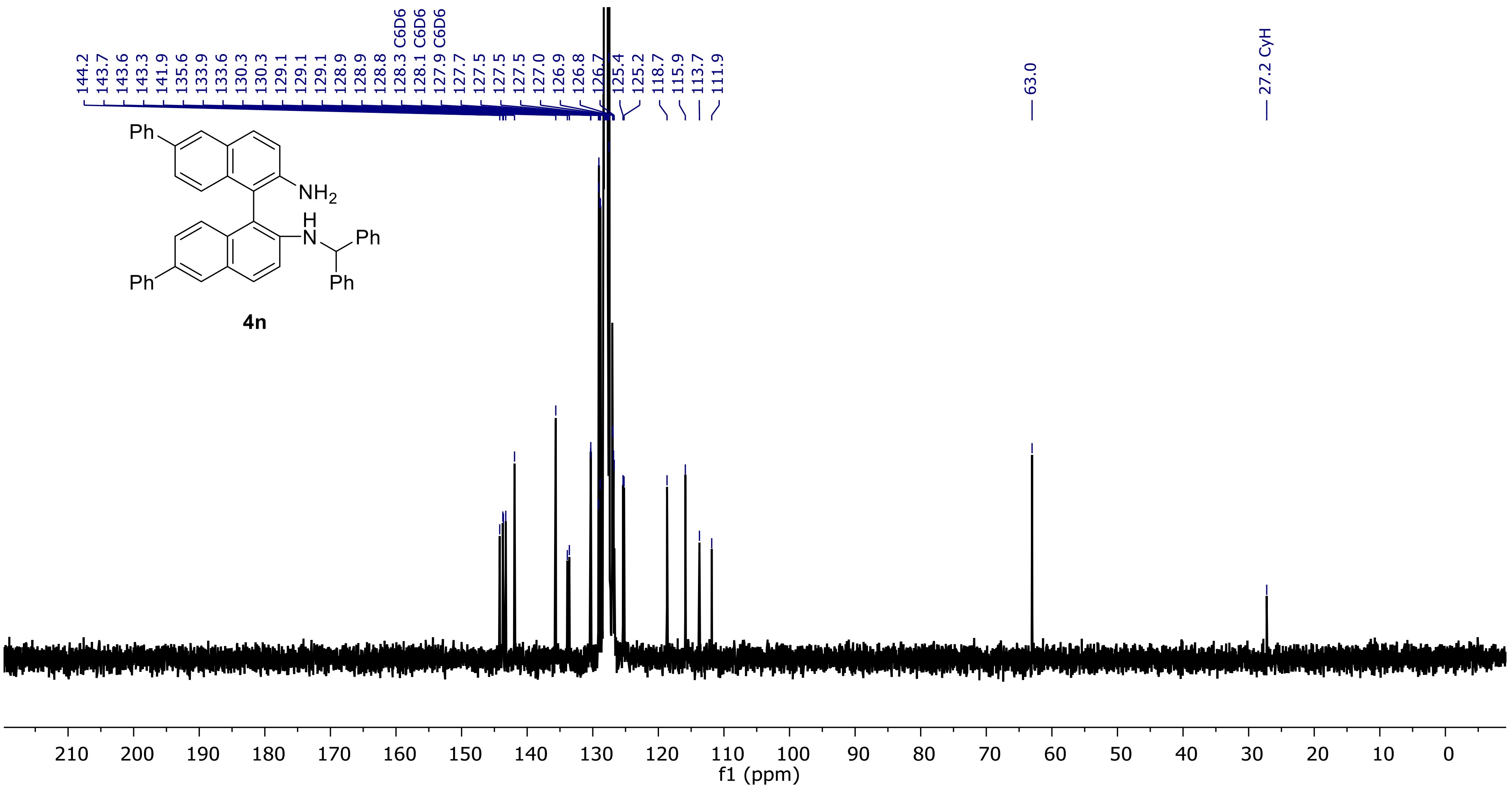

Figure S110. **^{1}H NMR** spectrum (500 MHz, C_6D_6, 298 K) of **4o**.

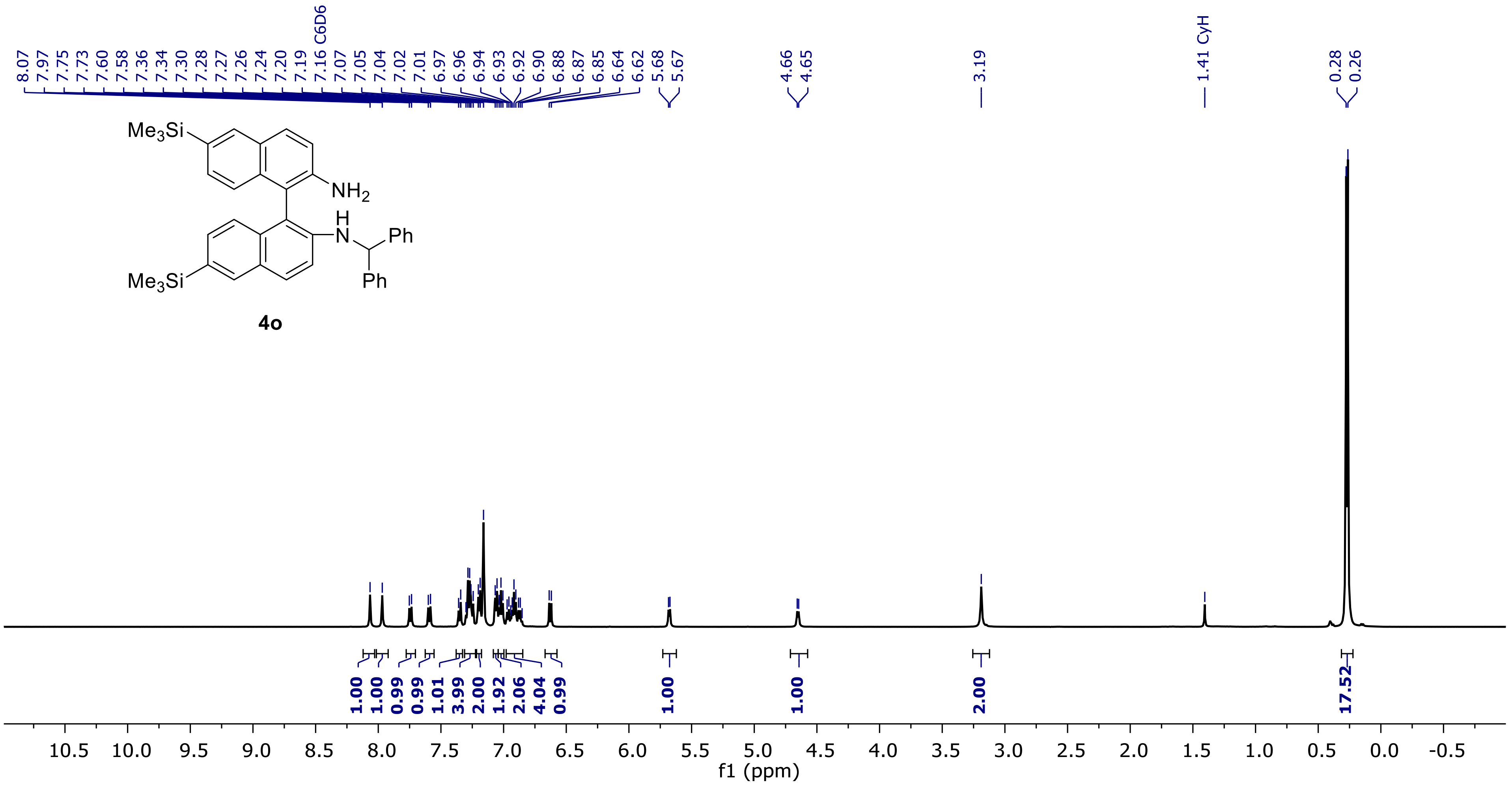

Figure S111. ^{13}**C{^{1}H} NMR** spectrum (126 MHz, C_6D_6, 298 K) of **4o**.

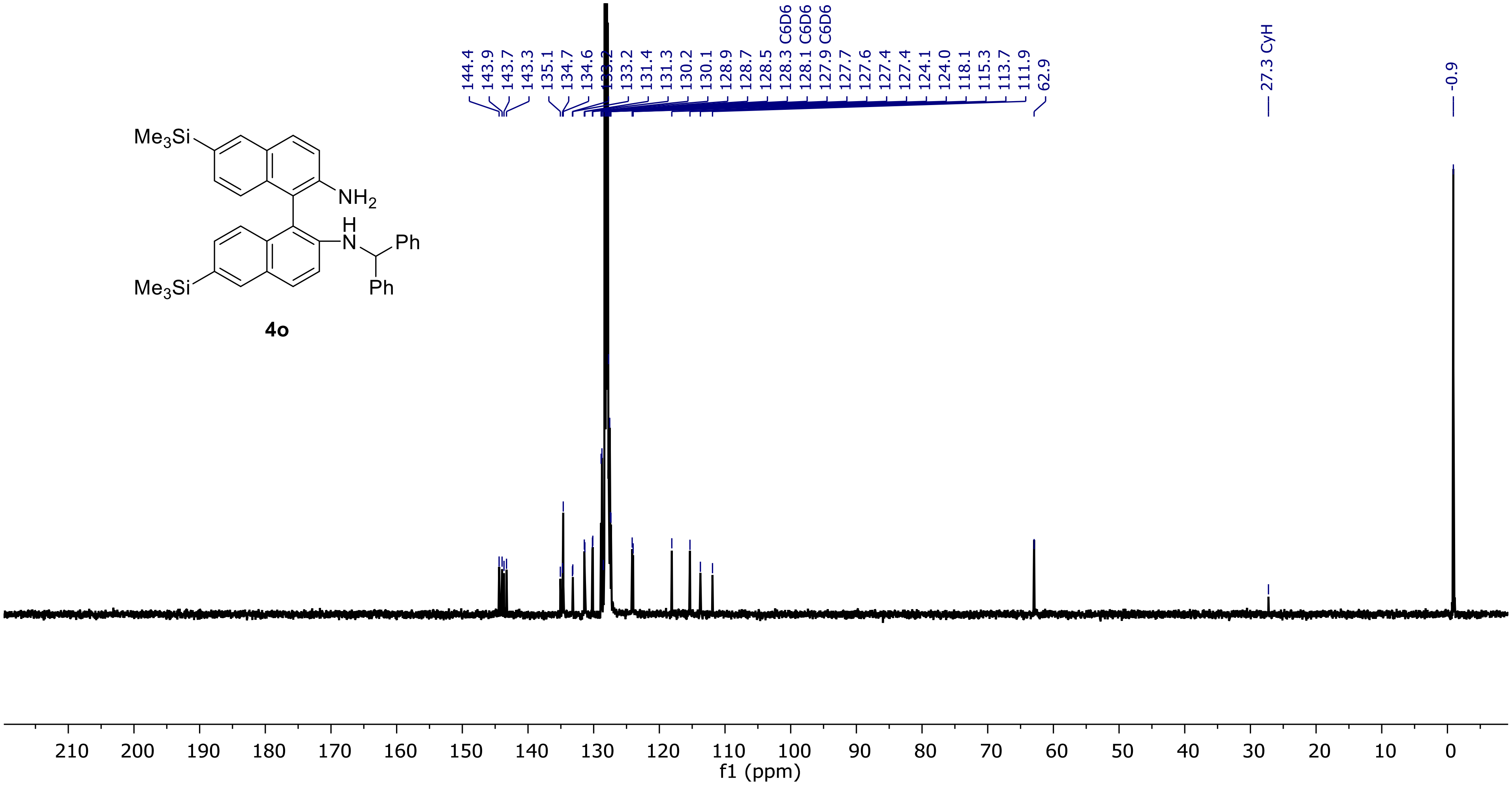

Figure S112. ^{29}**Si DEPT NMR** spectrum (99 MHz, C_6D_6, 298 K, optimized for J = 7.0 Hz) of **4o**.

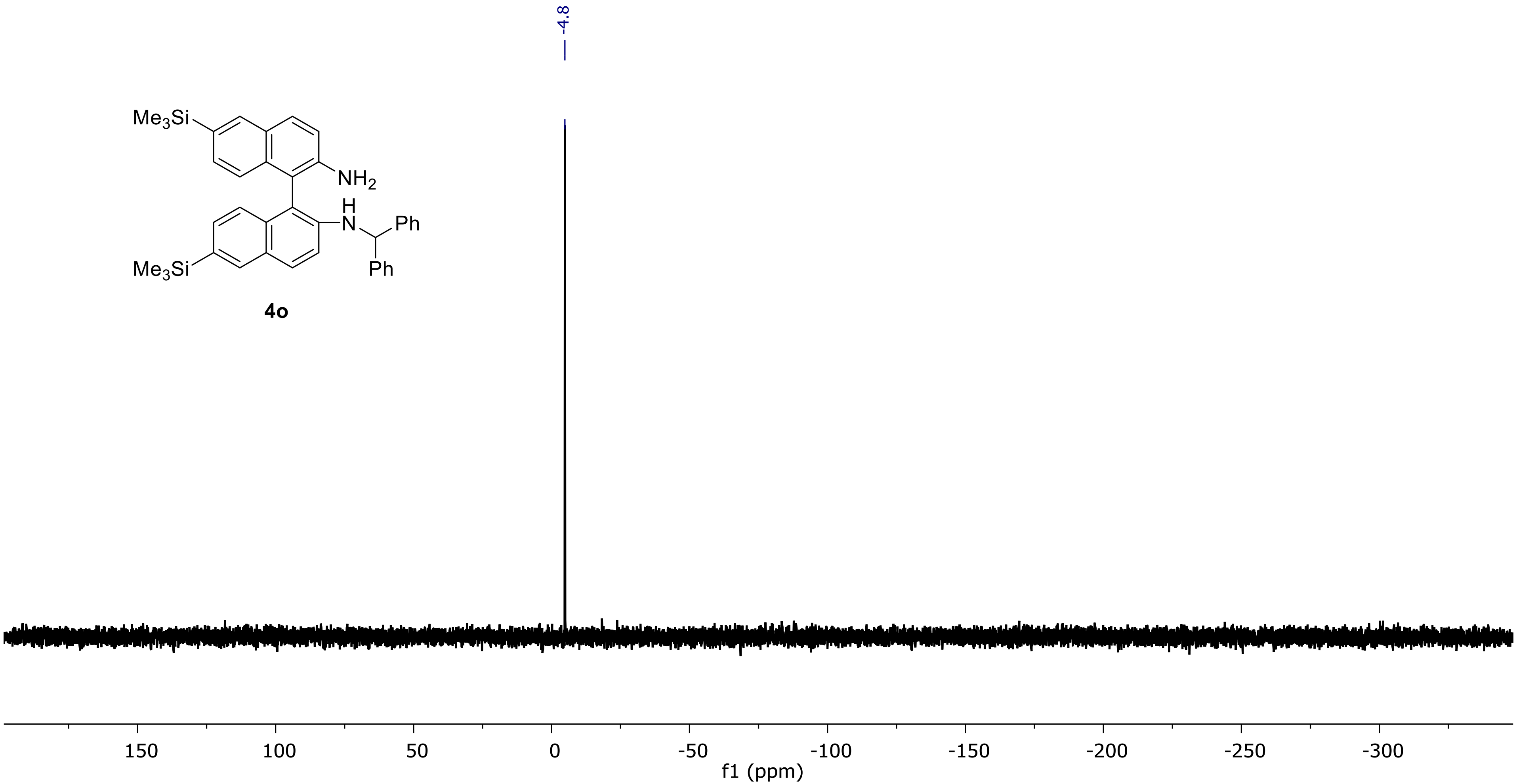

Figure S113. **^{1}H NMR** spectrum (500 MHz, C_6D_6, 298 K) of **4p**.

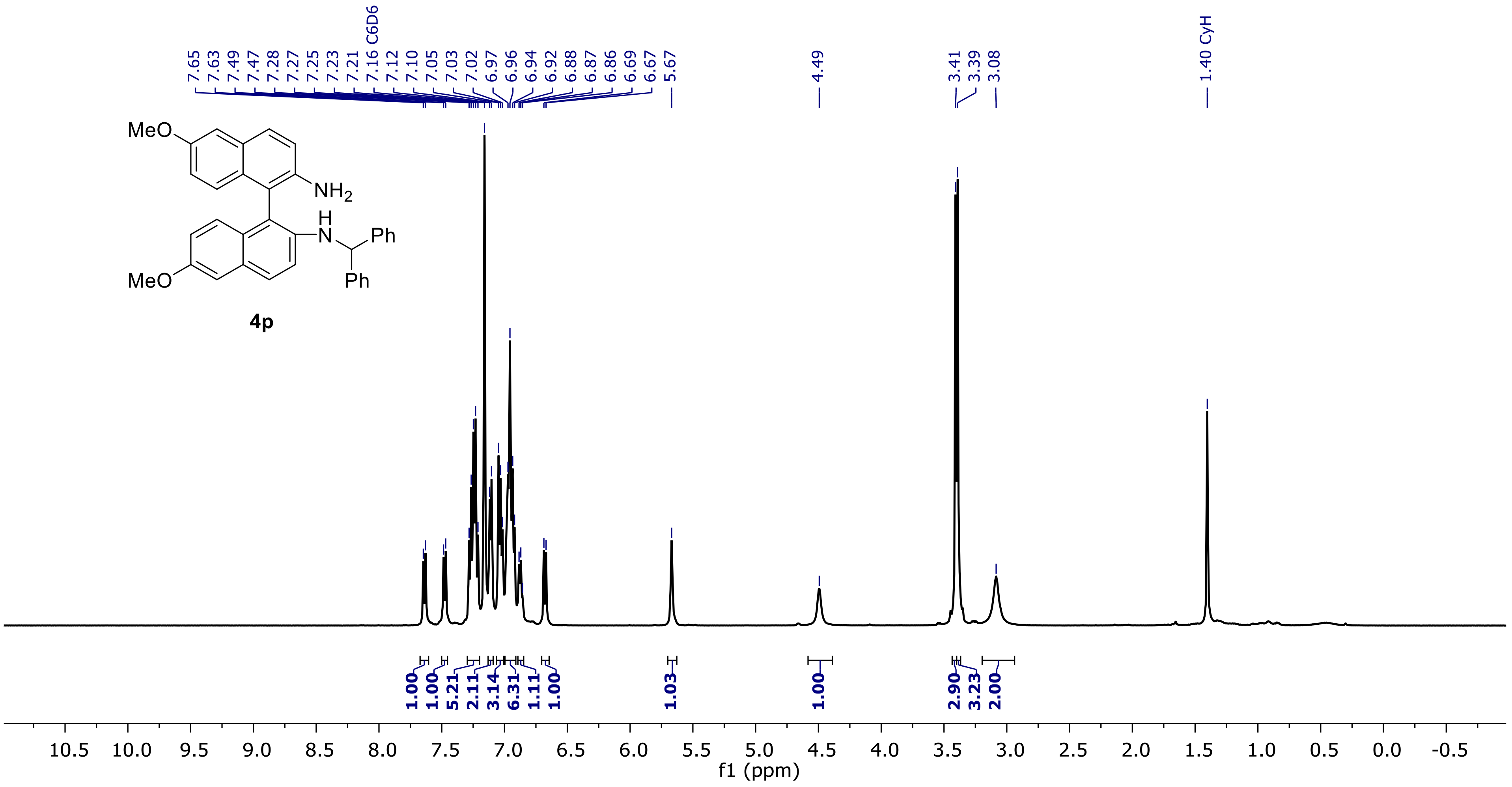

Figure S114. **^{13}C{^{1}H} NMR** spectrum (126 MHz, C_6D_6, 298 K) of **4p**.

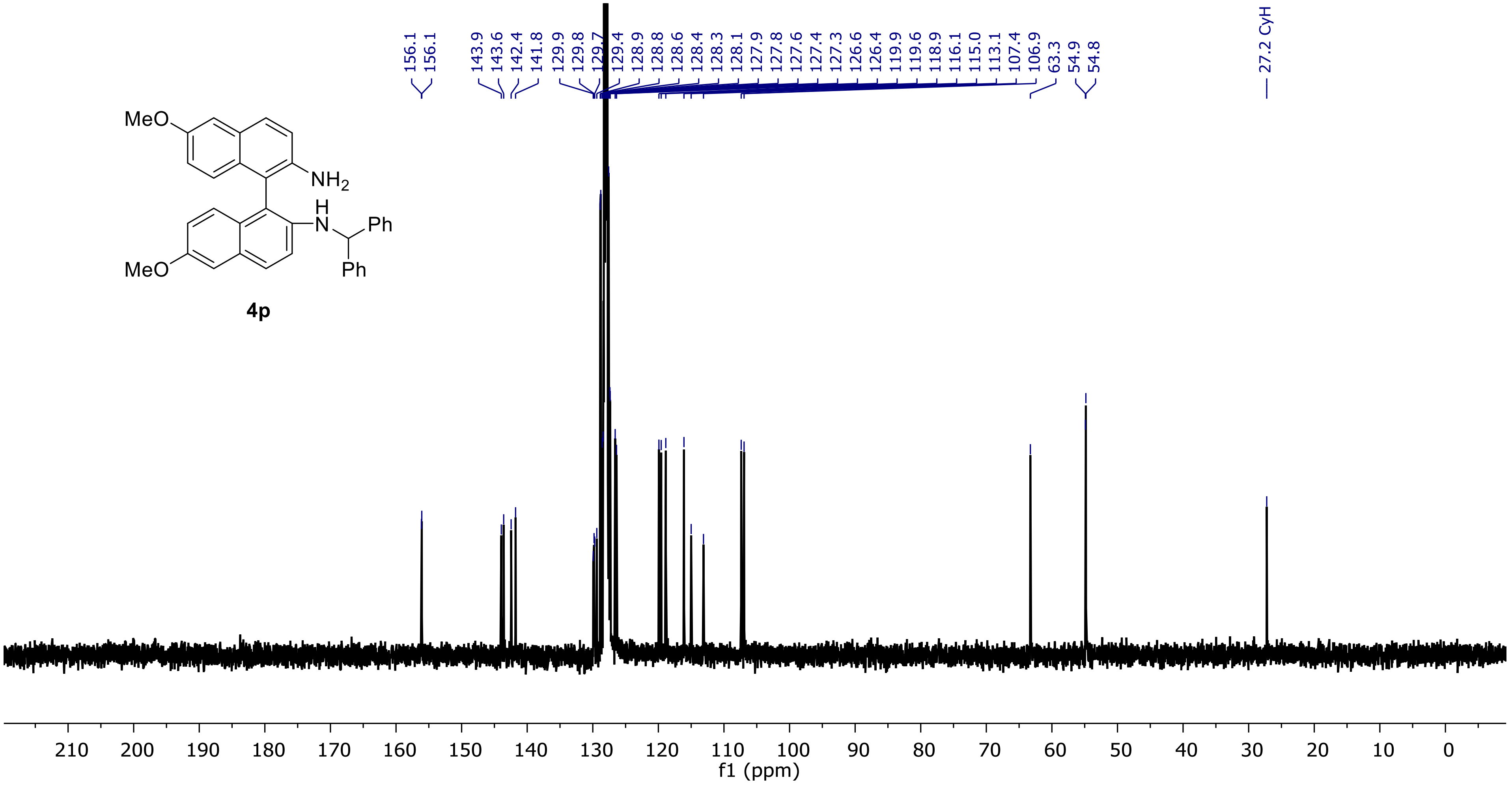

Figure S115. **^{1}H NMR** spectrum (500 MHz, $CDCl_3$, 298 K) of **S19**.

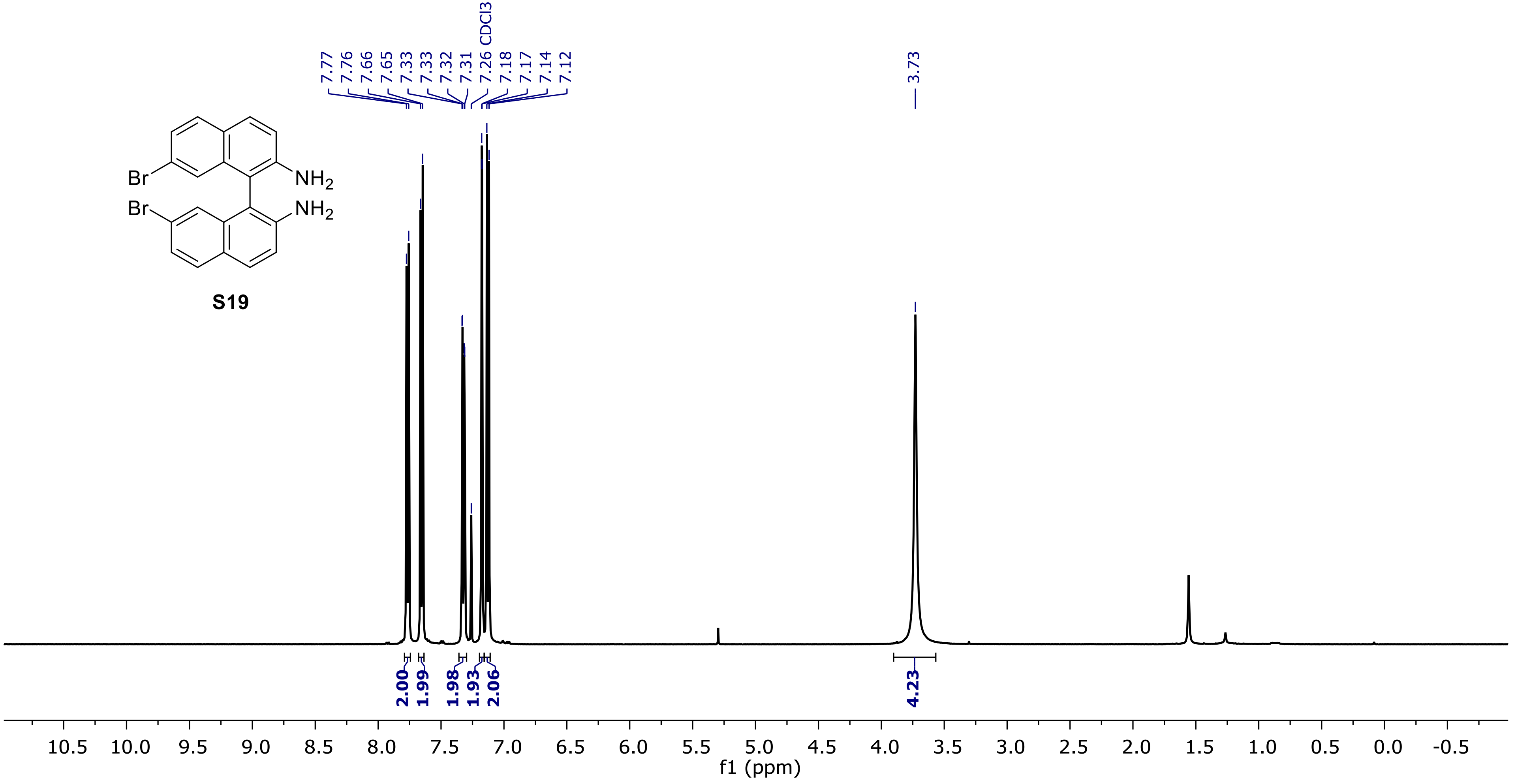

Figure S116. ^{13}**C{^{1}H} NMR** spectrum (126 MHz, $CDCl_3$, 298 K) of **S19**.

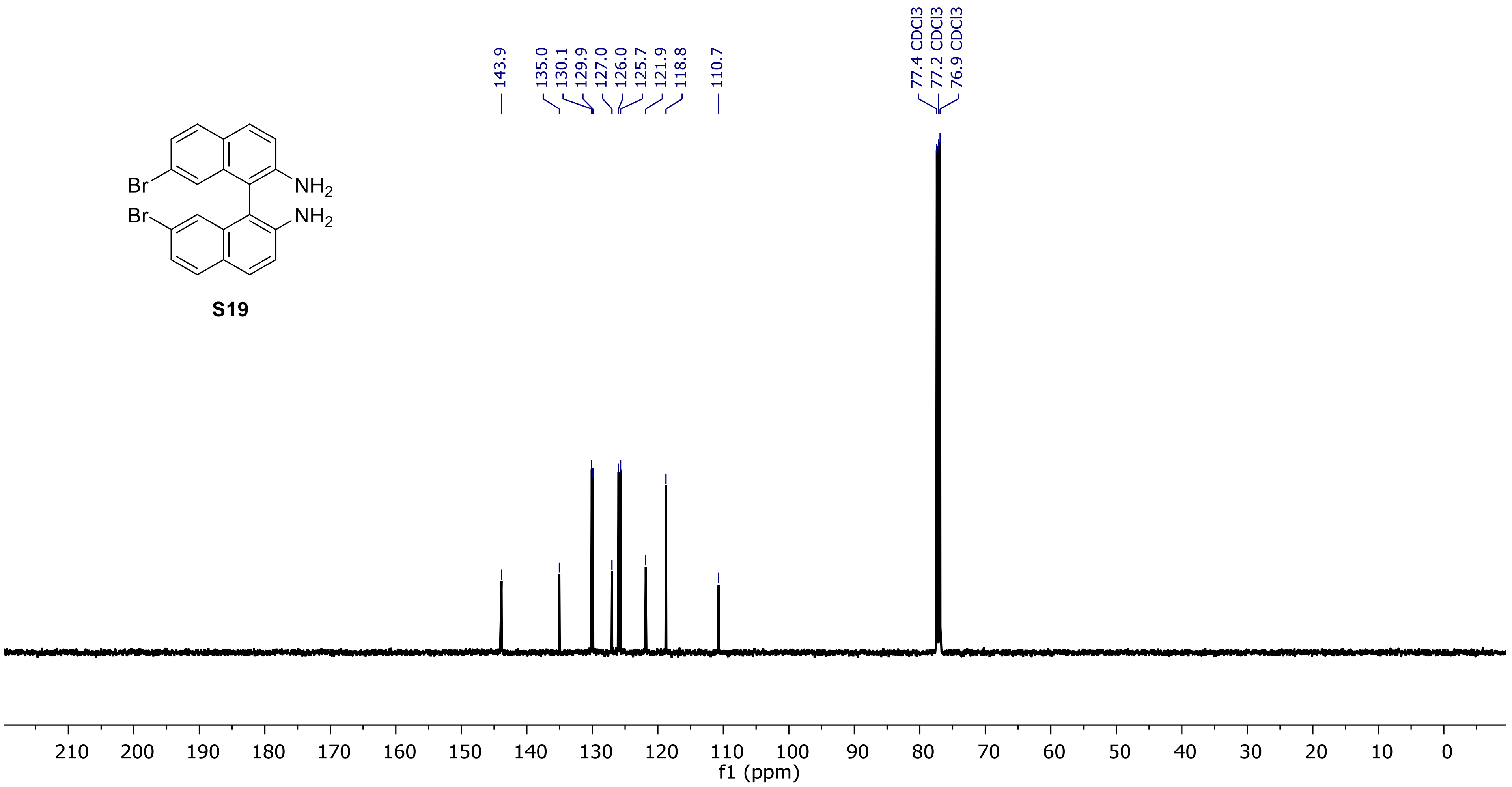

Figure S117. **^{1}H NMR** spectrum (500 MHz, C_6D_6, 298 K) of **4q**.

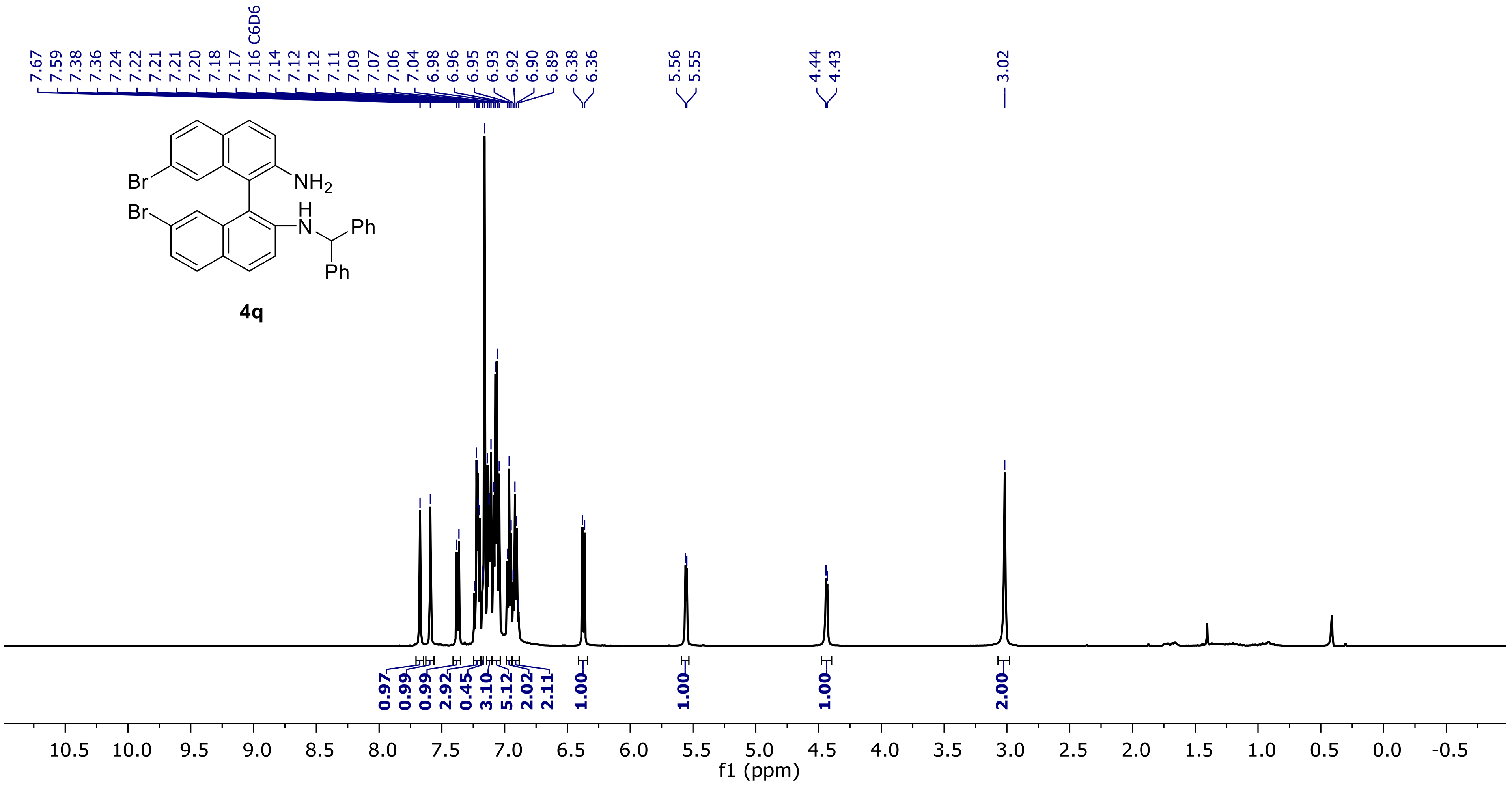

Figure S118. **$^{13}C\{^1H\}$ NMR** spectrum (126 MHz, C_6D_6, 298 K) of **4q**.

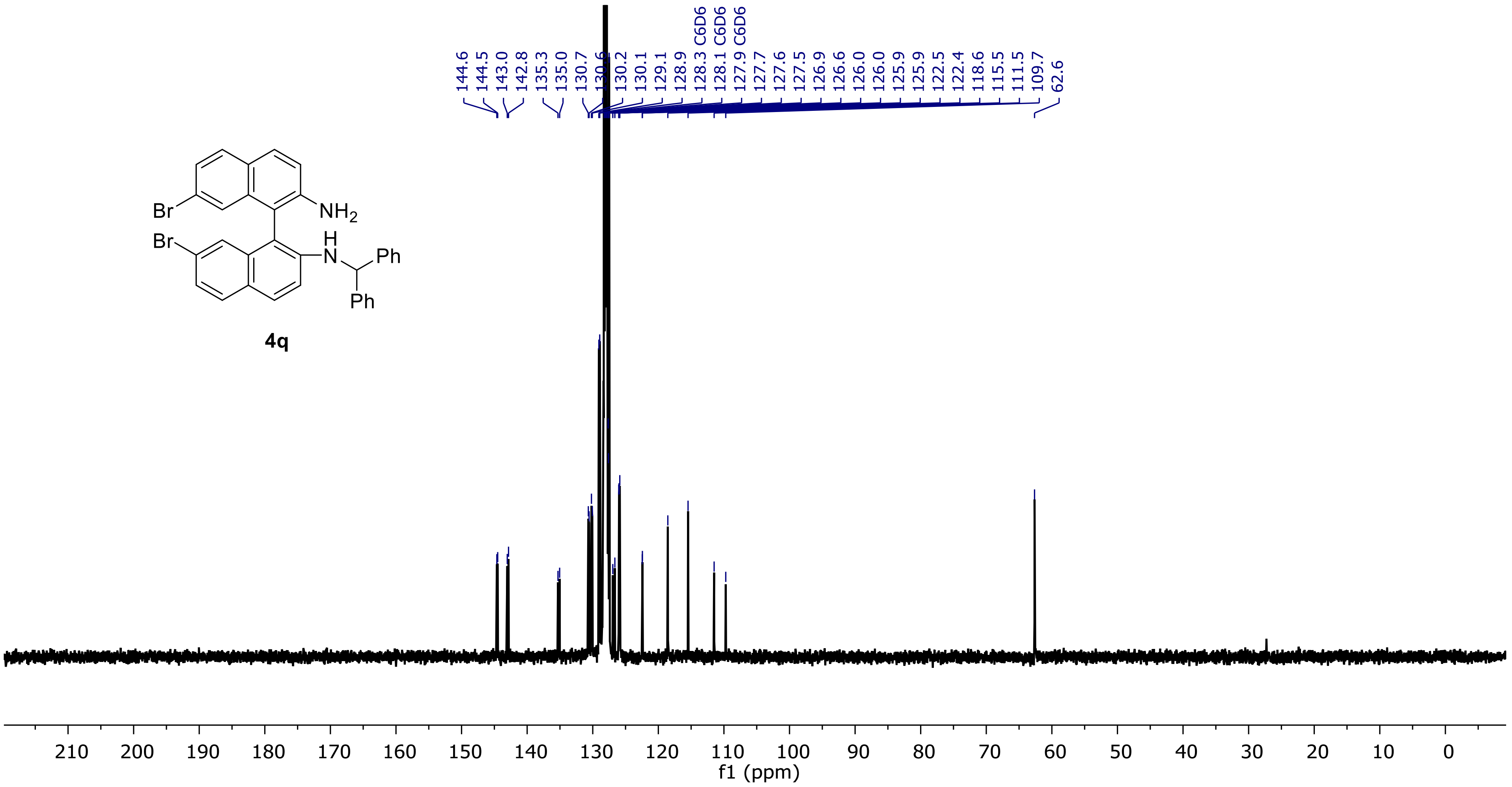

Figure S119. **^{1}H NMR** spectrum (500 MHz, C_6D_6, 298 K) of **4r**.

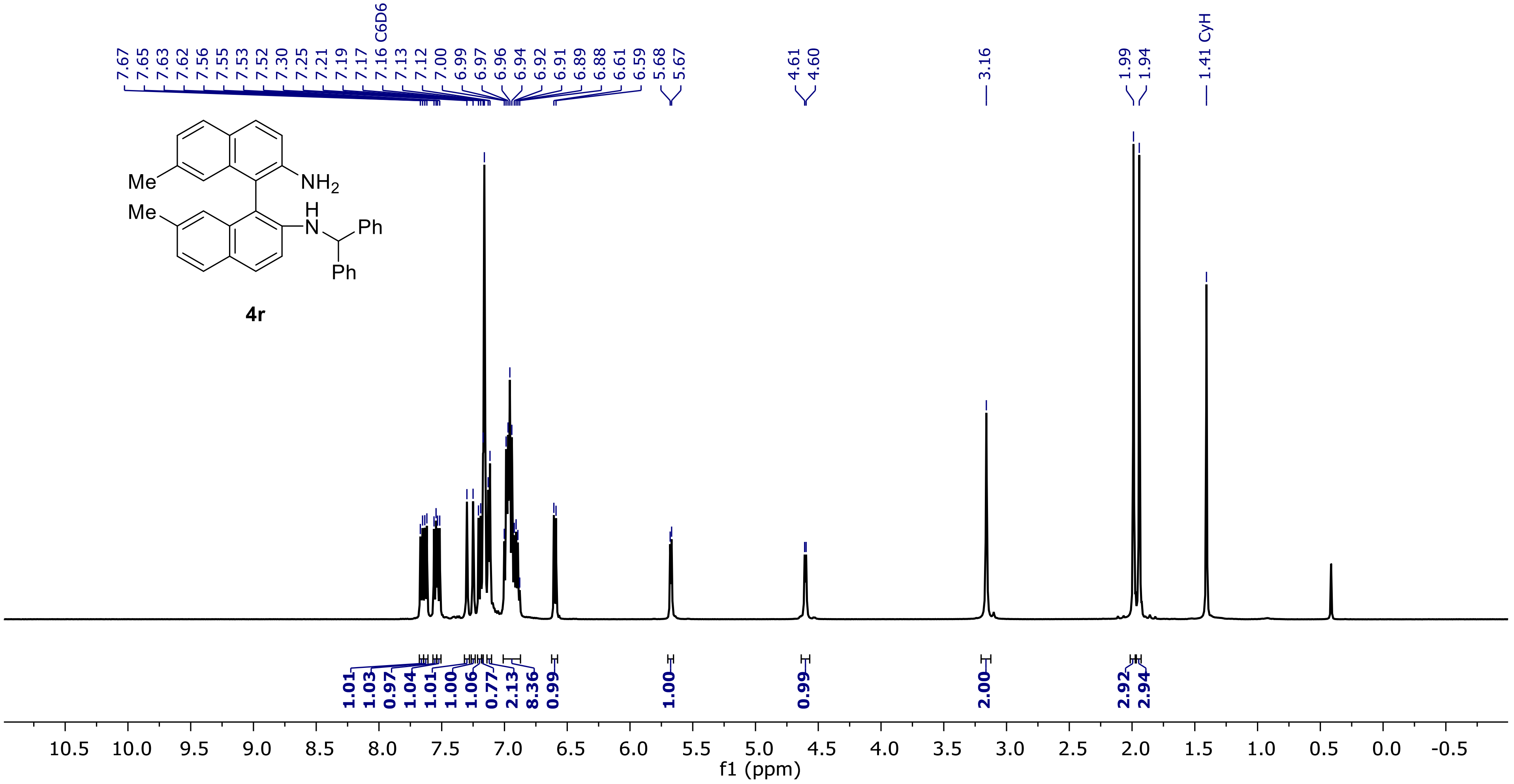

Figure S120. **$^{13}C\{^{1}H\}$ NMR** spectrum (126 MHz, C_6D_6, 298 K) of **4r**.

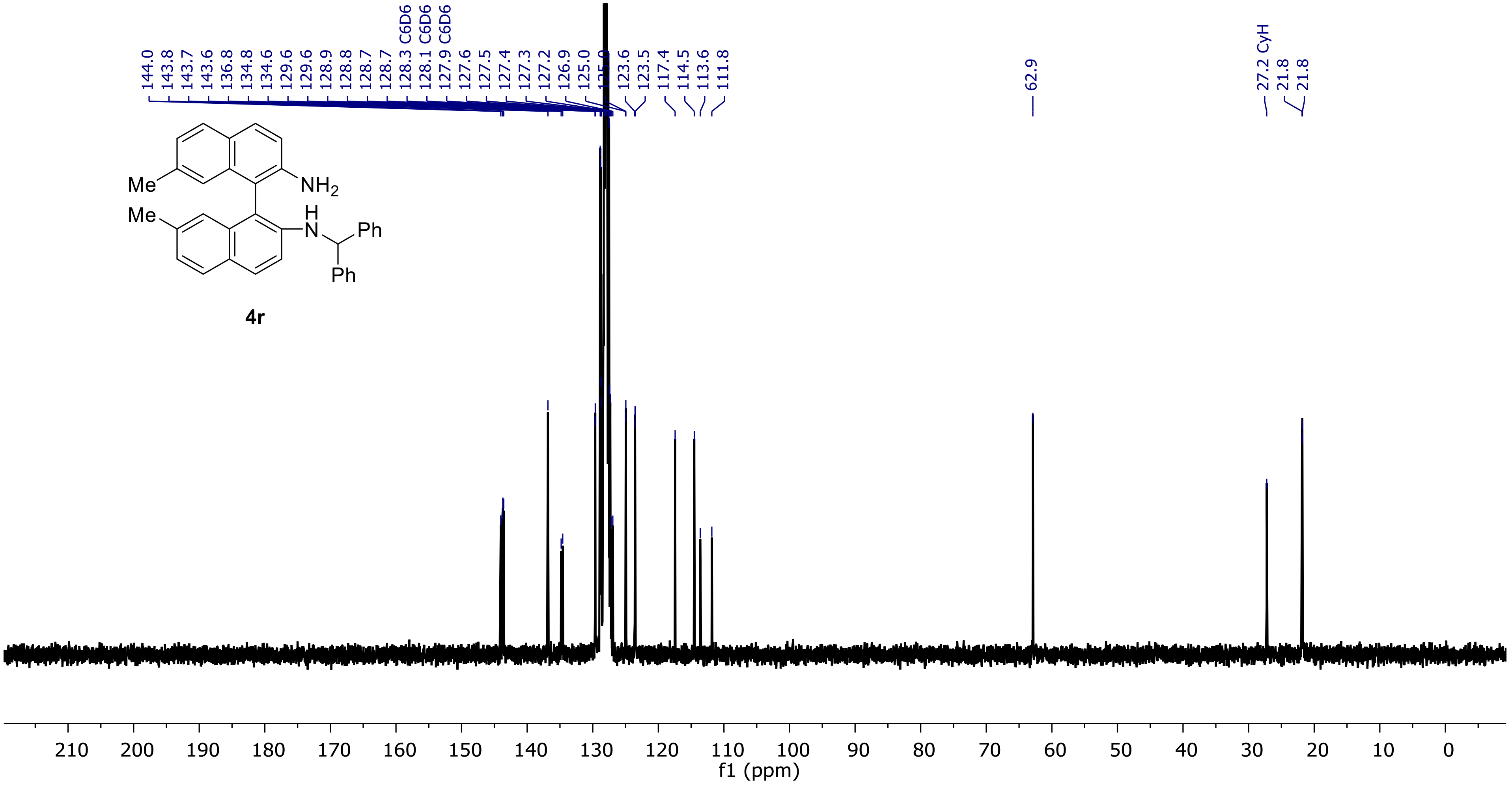

Figure S121. **¹H NMR** spectrum (500 MHz, $CDCl_3$, 298 K) of **S23**.

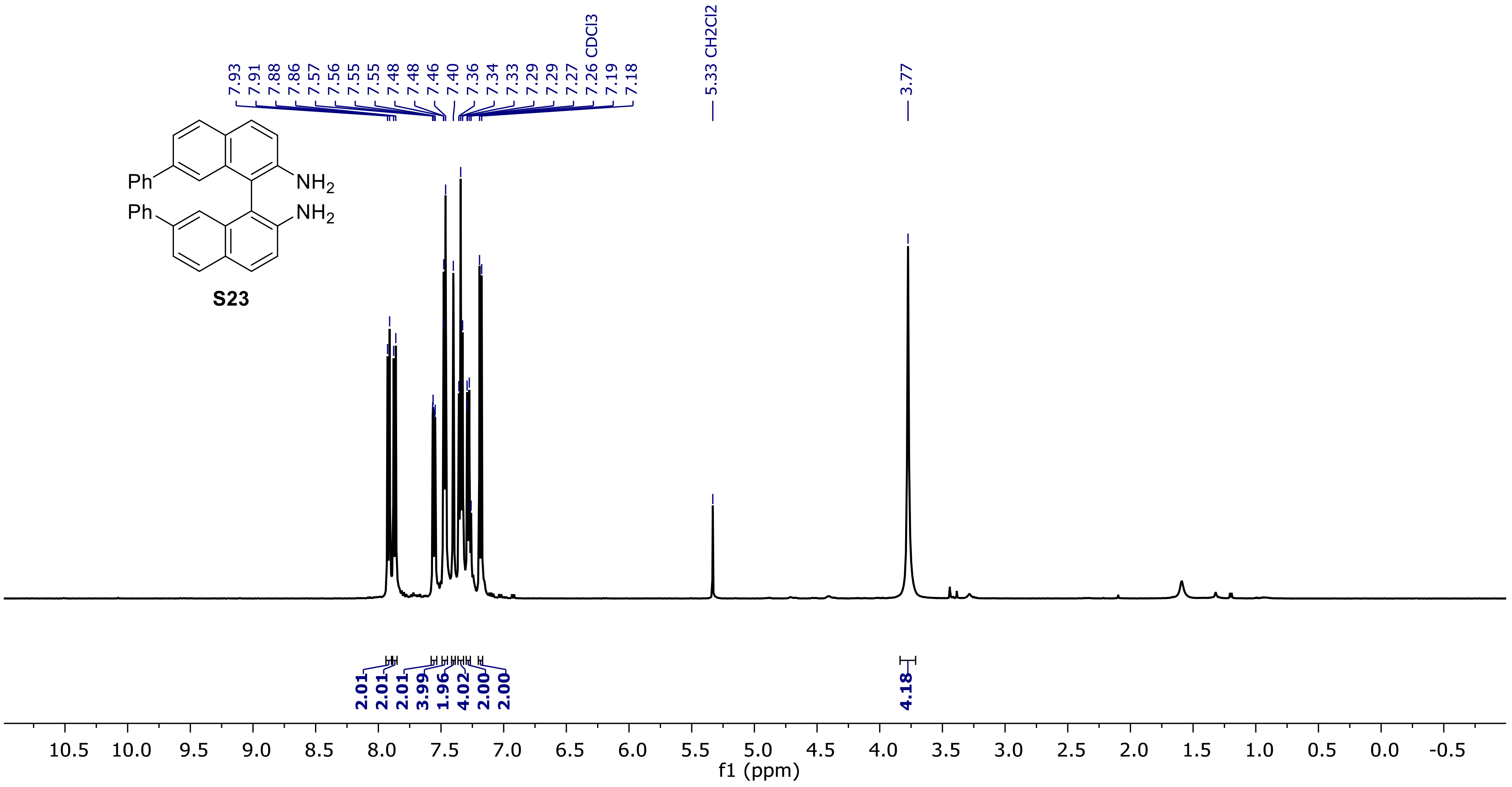

Figure S122. ^{13}**C{^{1}H} NMR** spectrum (126 MHz, $CDCl_3$, 298 K) of **S23**.

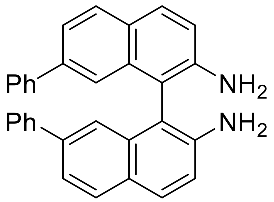

S23

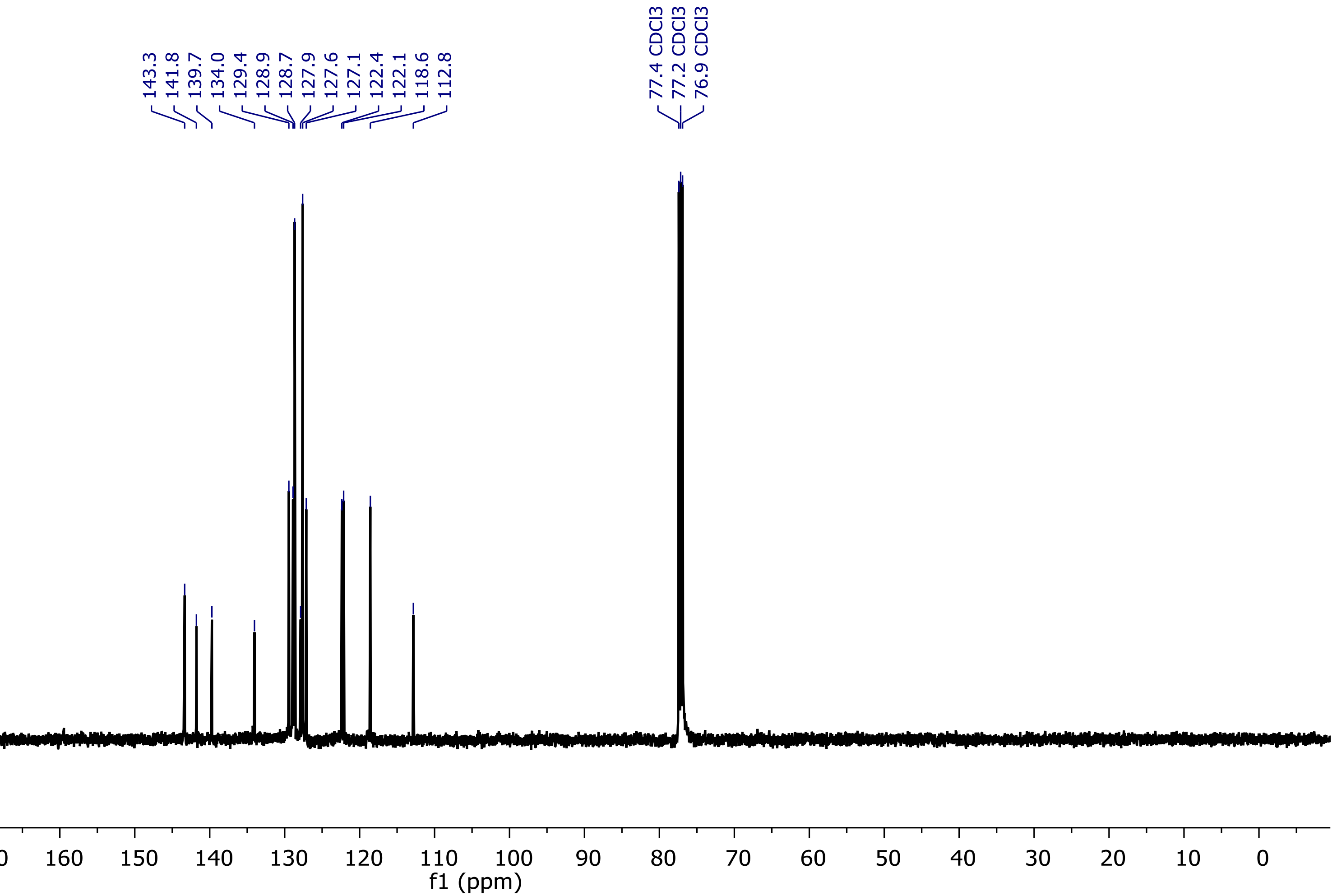

Figure S123. **¹H NMR** spectrum (500 MHz, C_6D_6, 298 K) of **4s**.

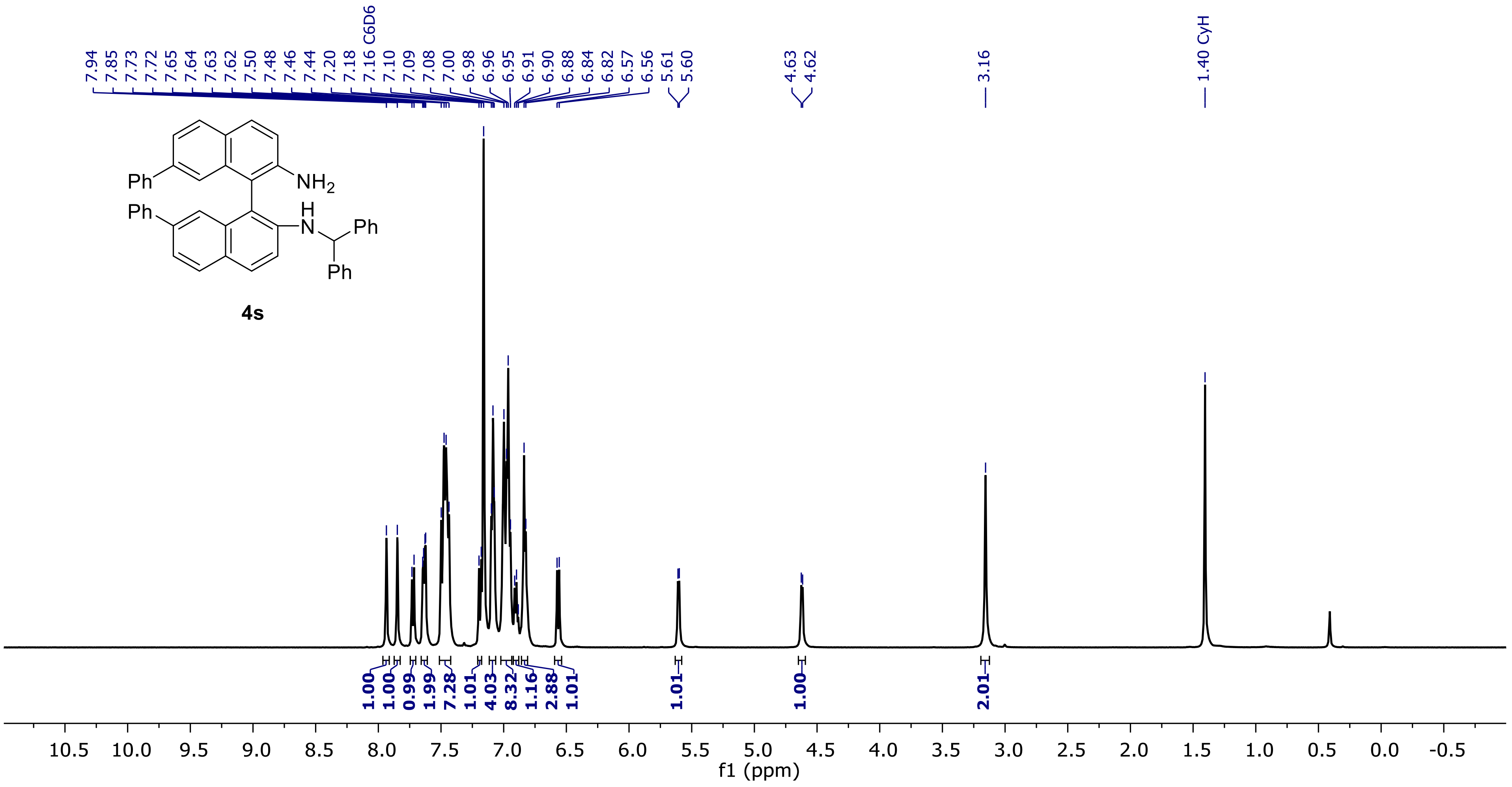

Figure S124. $^{13}C\{^{1}H\}$ **NMR** spectrum (126 MHz, C_6D_6, 298 K) of **4s**.

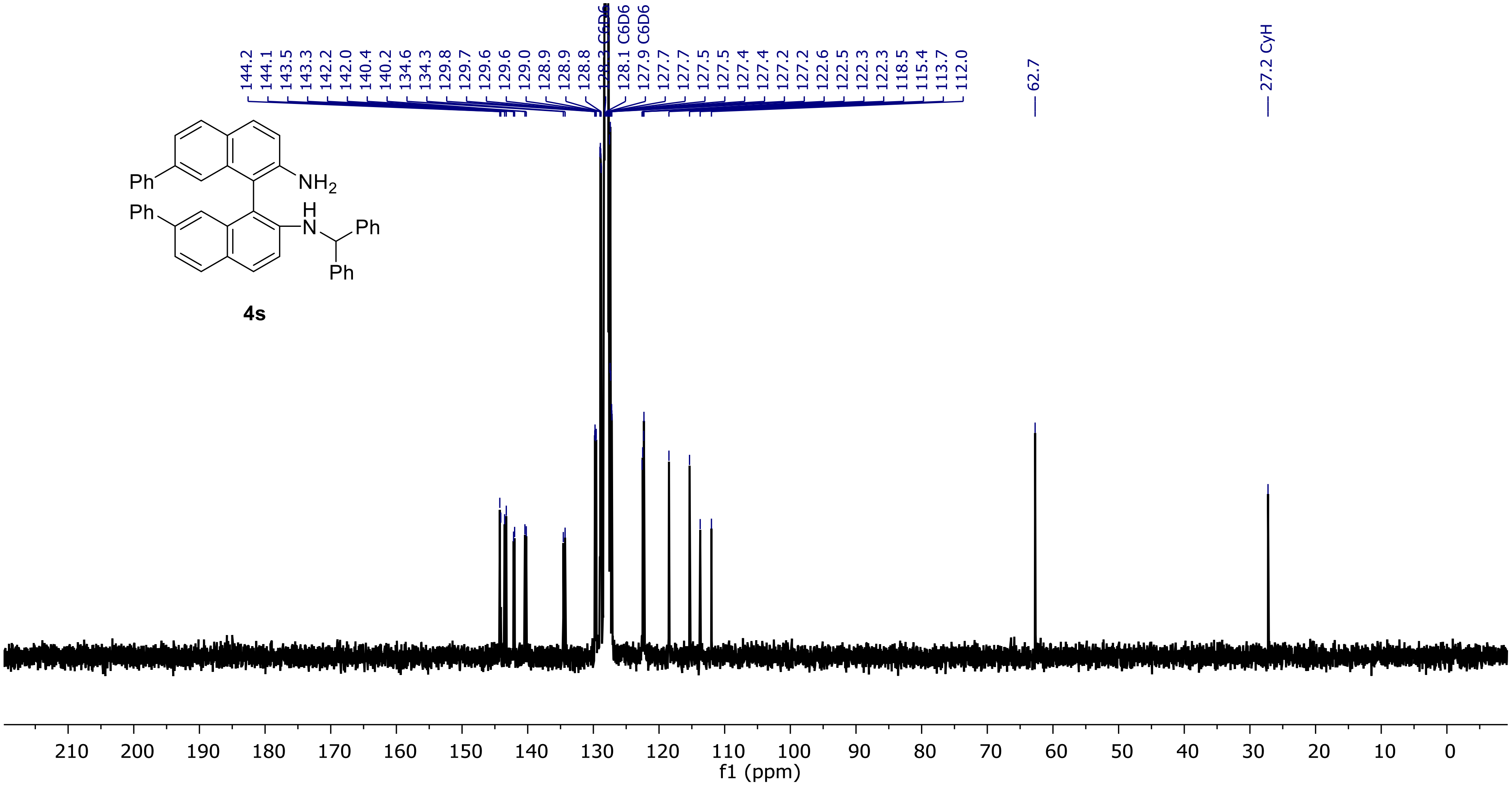

Figure S125. **^{1}H NMR** spectrum (500 MHz, C_6D_6, 298 K) of **4t**.

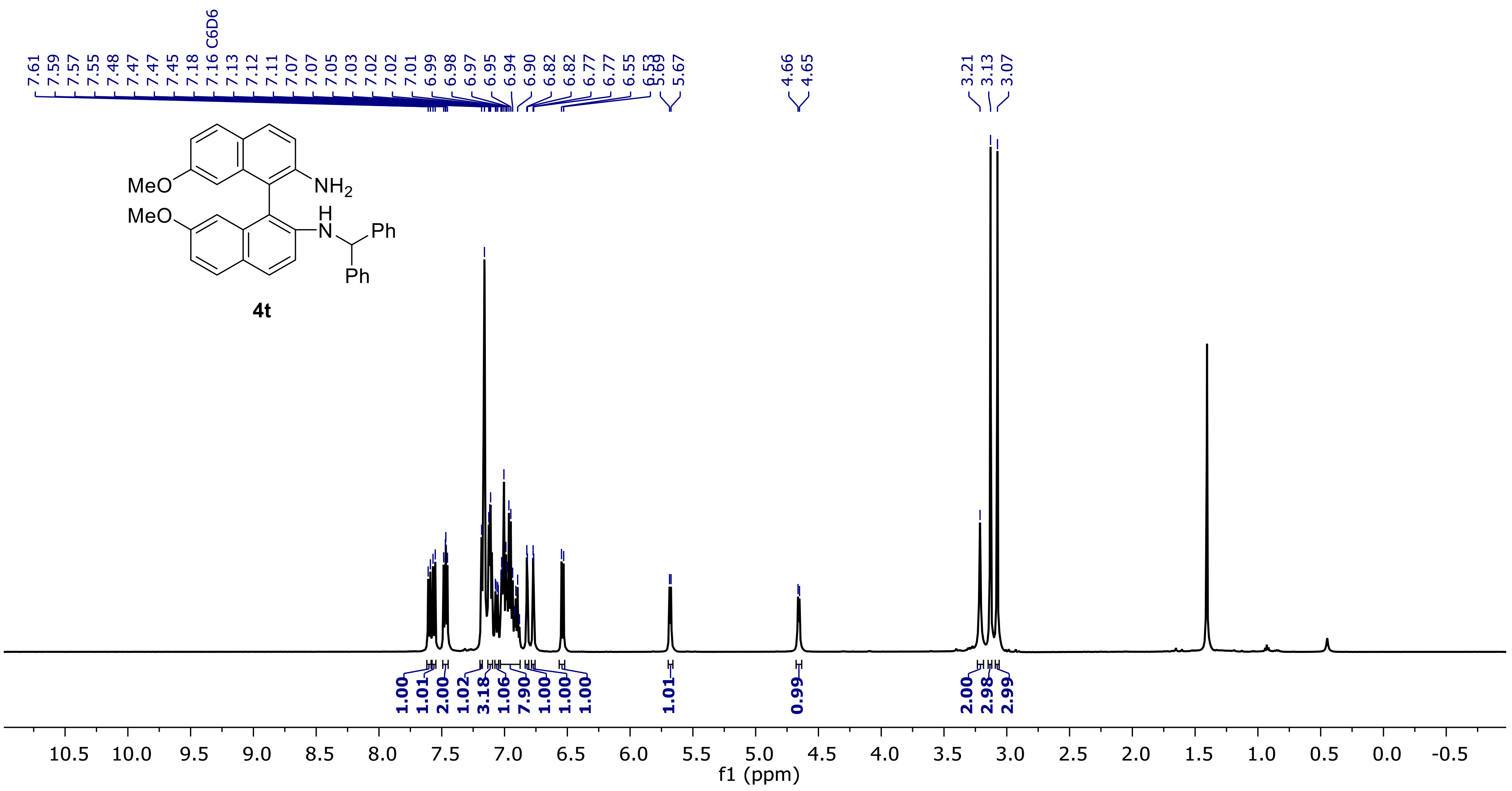

Figure S126. ^{13}**C{^{1}H} NMR** spectrum (126 MHz, C_6D_6, 298 K) of **4t**.

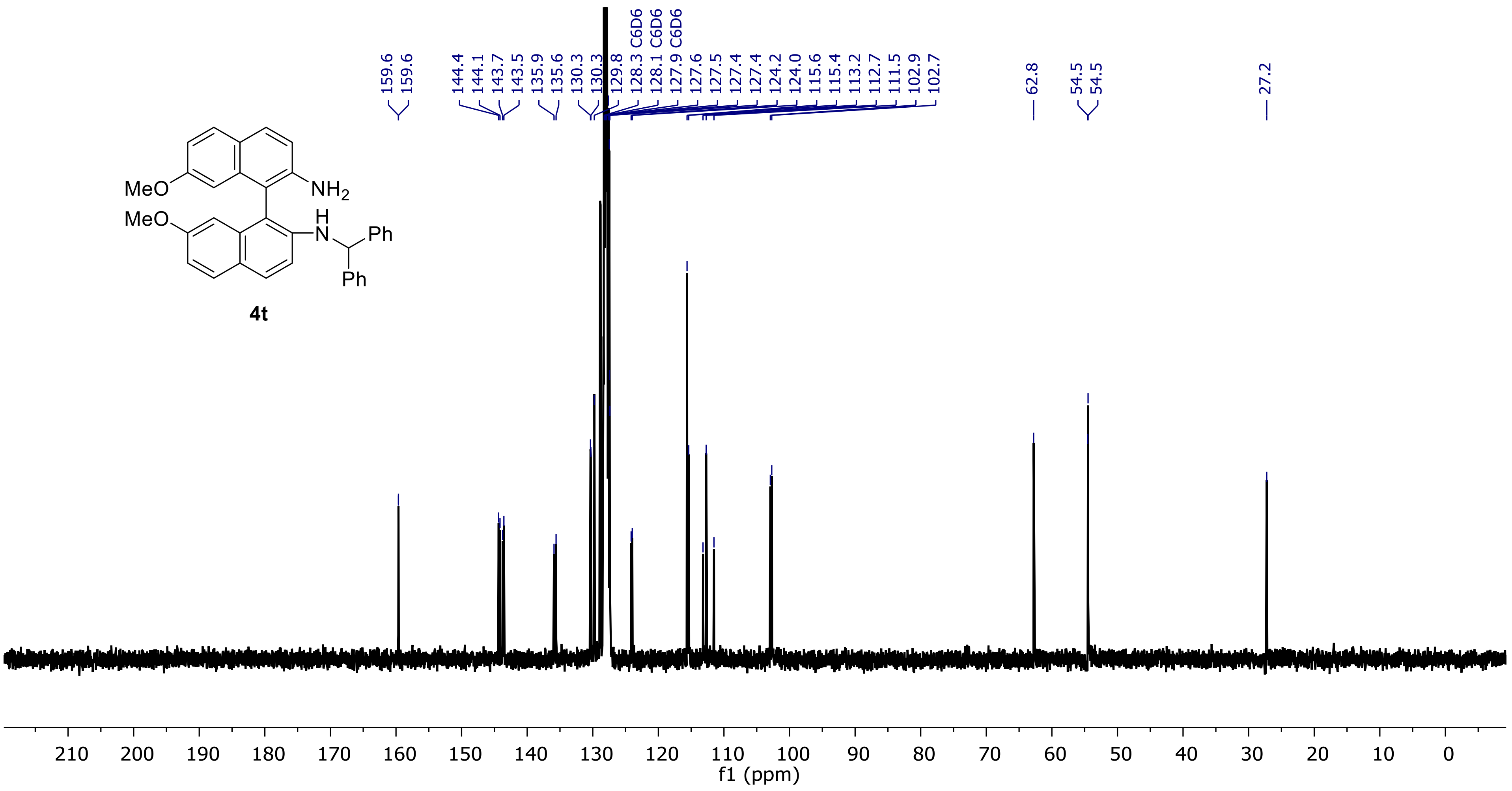

Figure S127. **¹H NMR** spectrum (500 MHz, $CDCl_3$, 298 K) of **S25**.

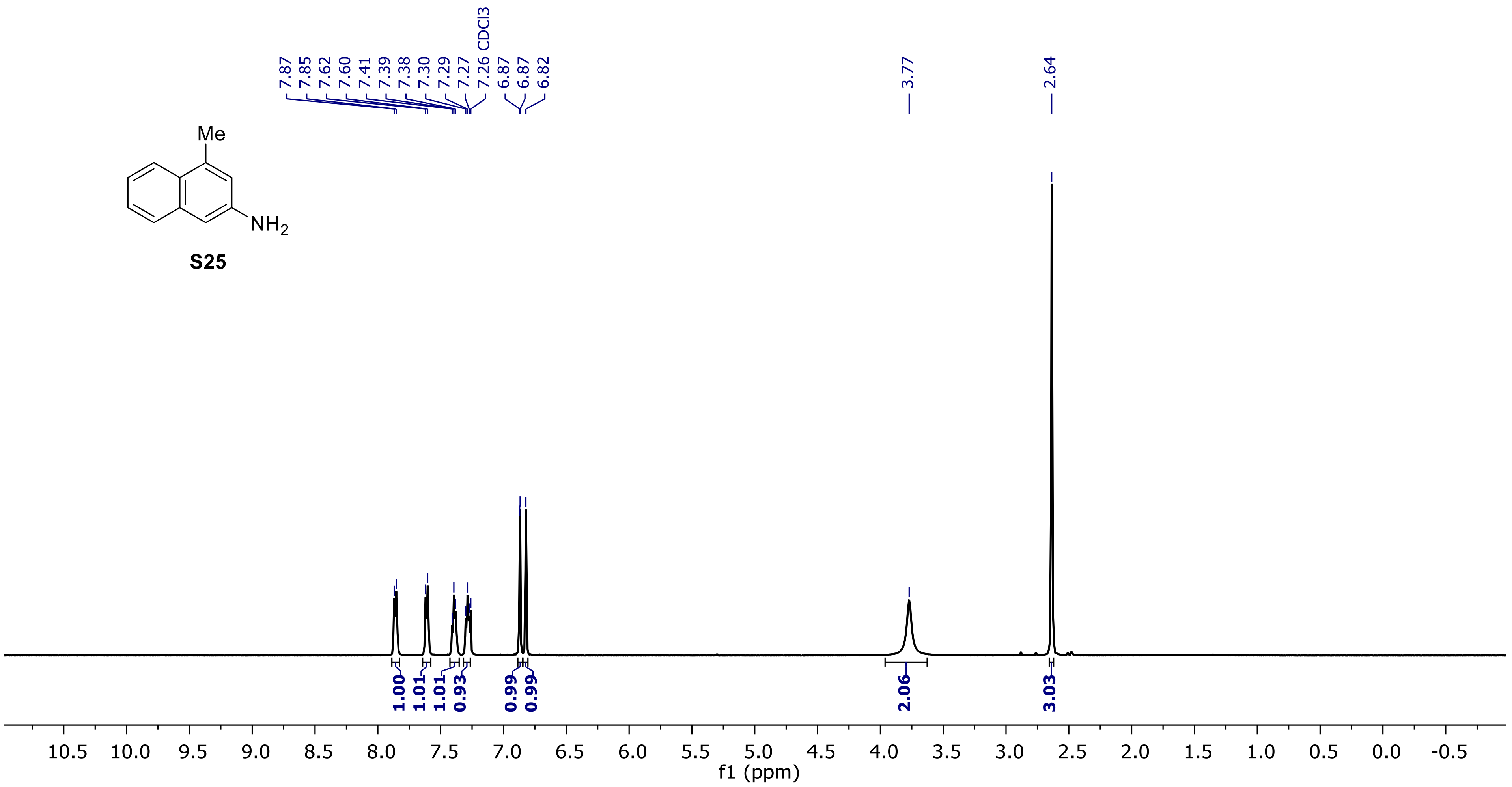

Figure SX. **^{13}C{^{1}H} NMR** spectrum (126 MHz, $CDCl_3$, 298 K) of **S25**.

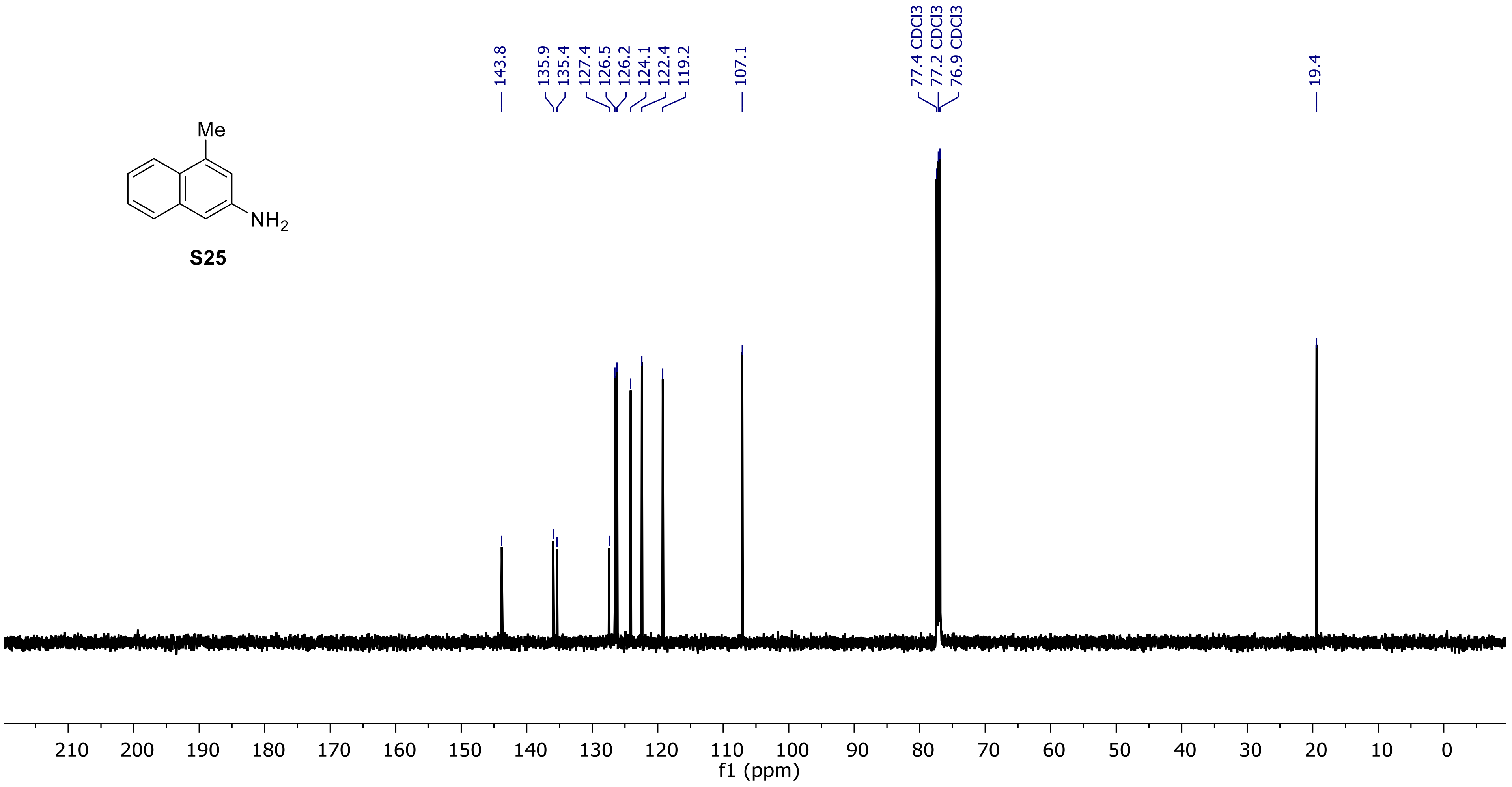

Figure S129. ^{1}H NMR spectrum (500 MHz, $CDCl_3$, 298 K) of **S26**.

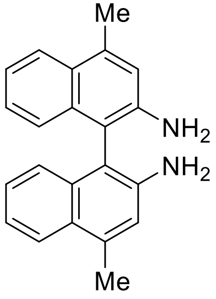

S26

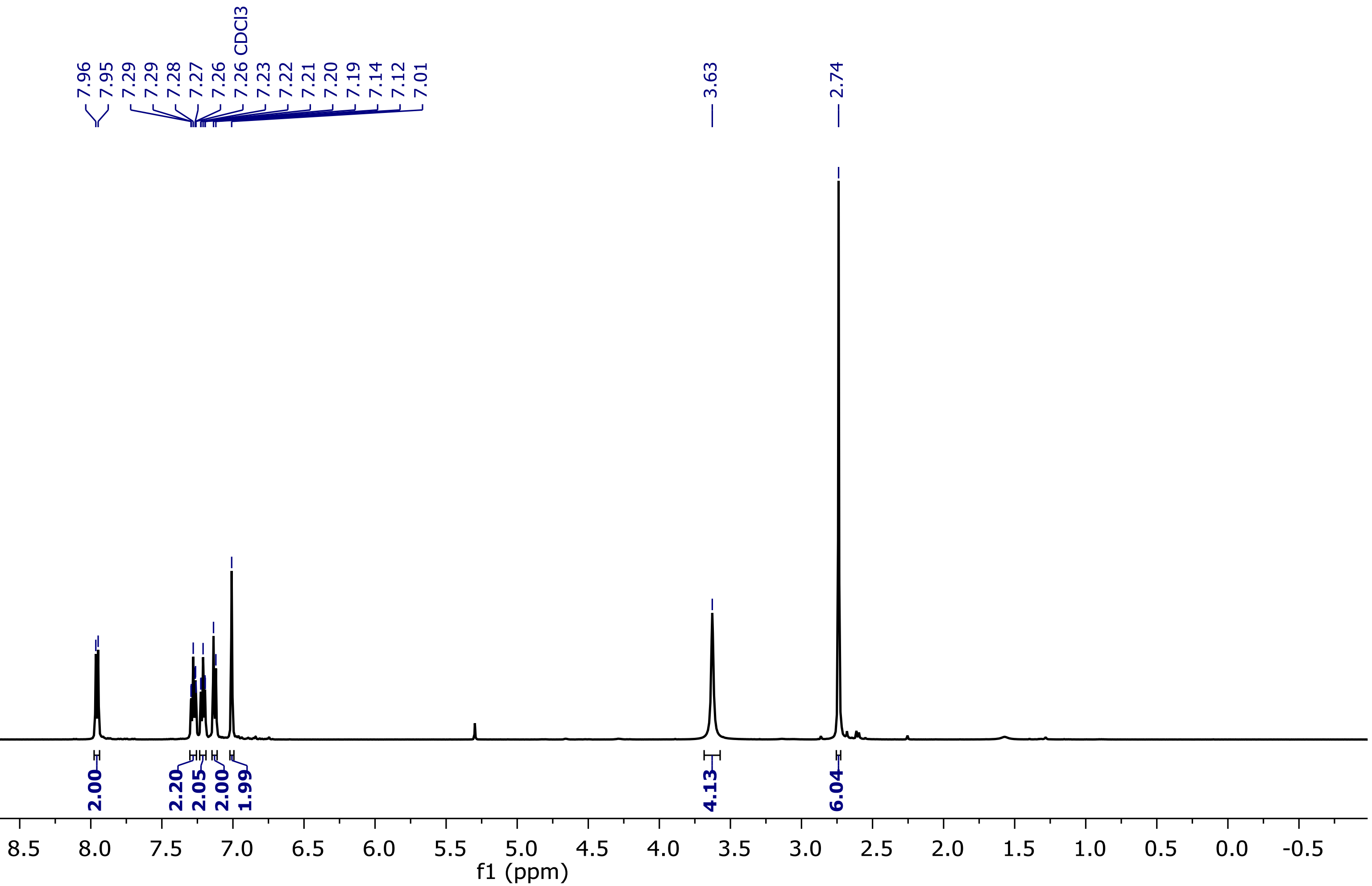

Figure S130. **$^{13}C\{^1H\}$ NMR** spectrum (126 MHz, $CDCl_3$, 298 K) of **S26**.

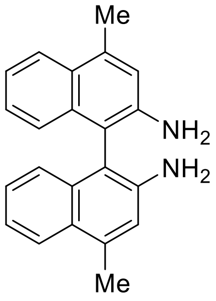

S26

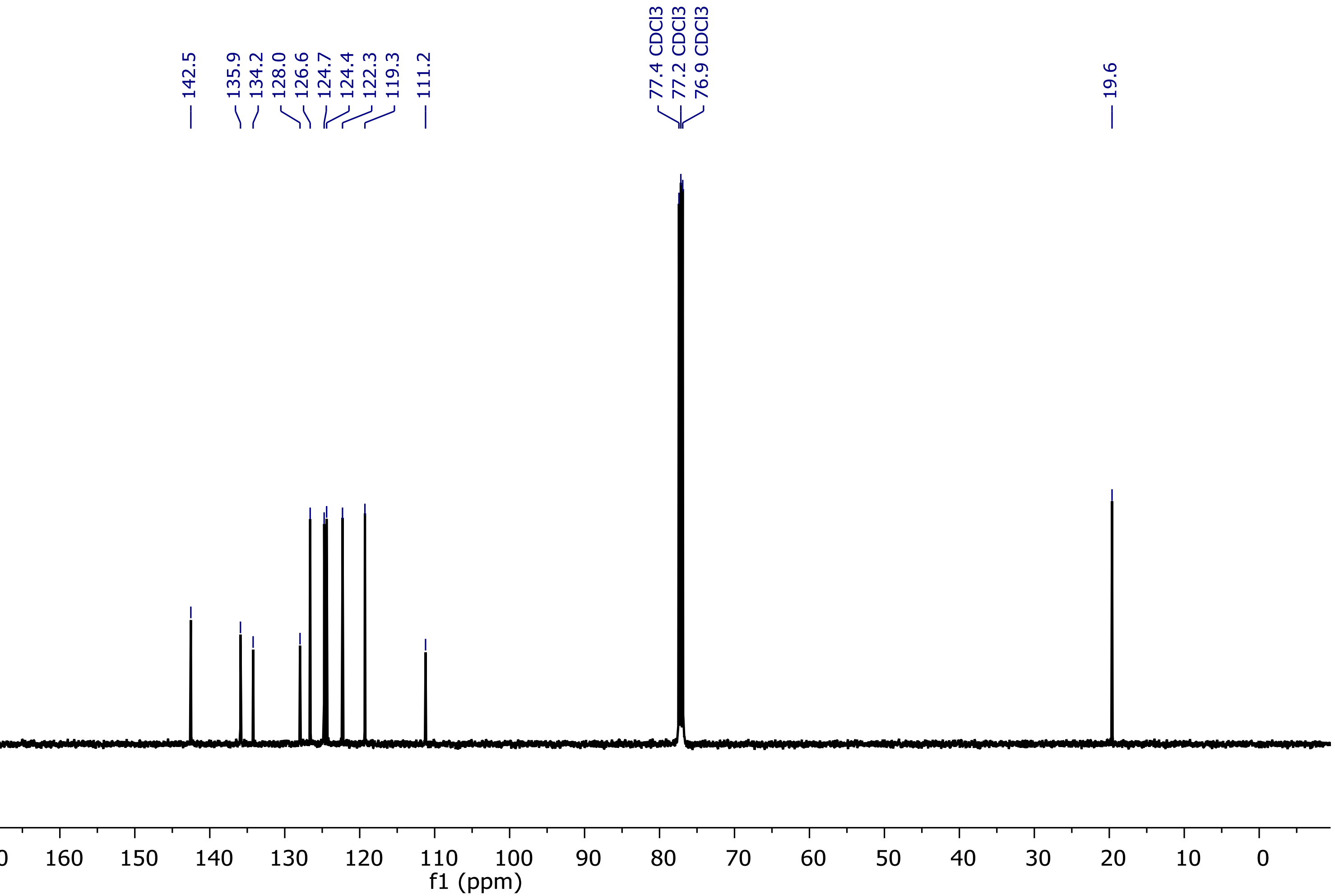

Figure S131. **^{1}H NMR** spectrum (500 MHz, C_6D_6, 298 K) of **4u**.

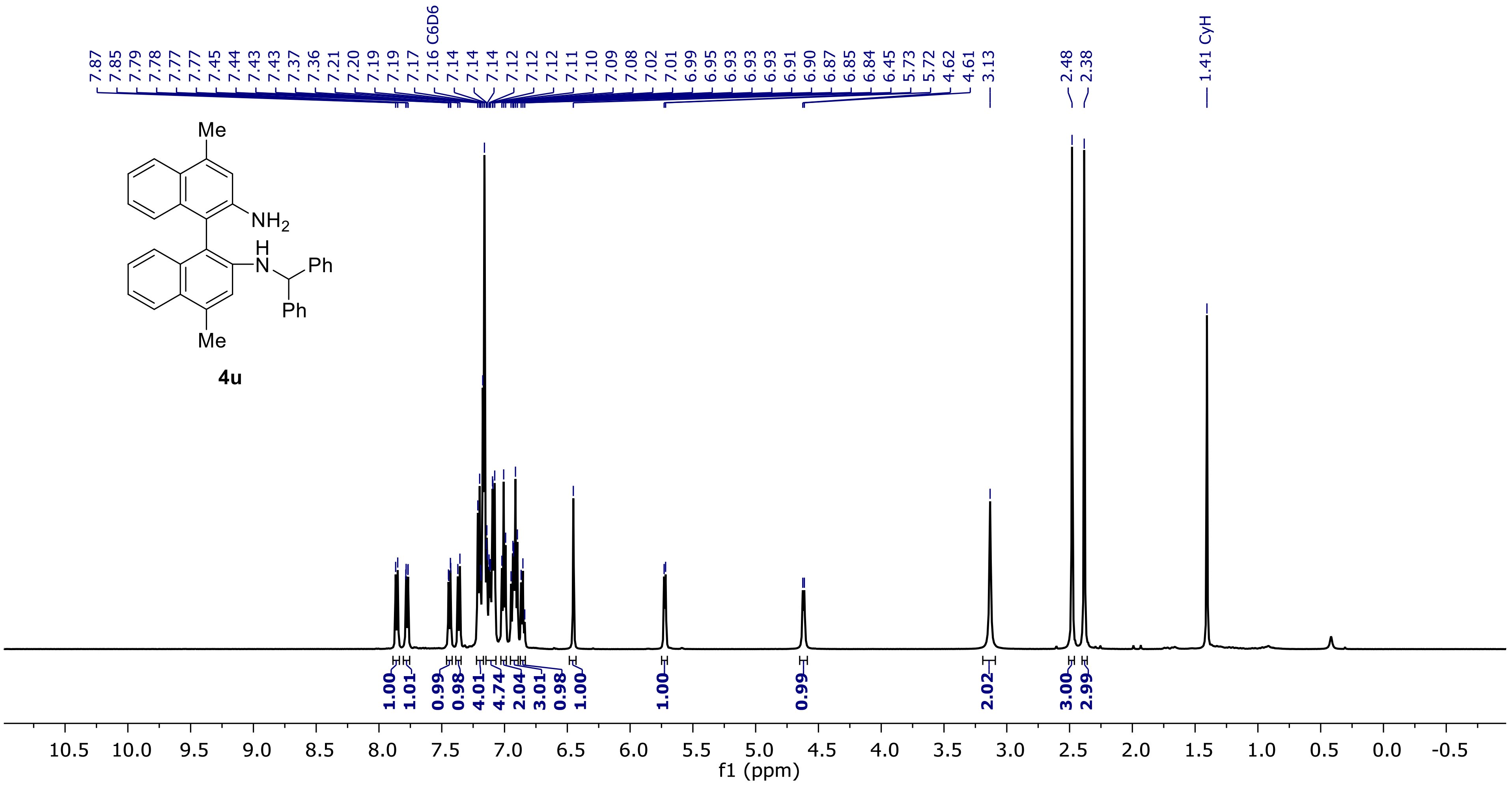

Figure S132. $^{13}C\{^1H\}$ **NMR** spectrum (126 MHz, C_6D_6, 298 K) of **4u**.

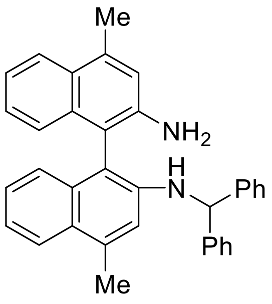

4u

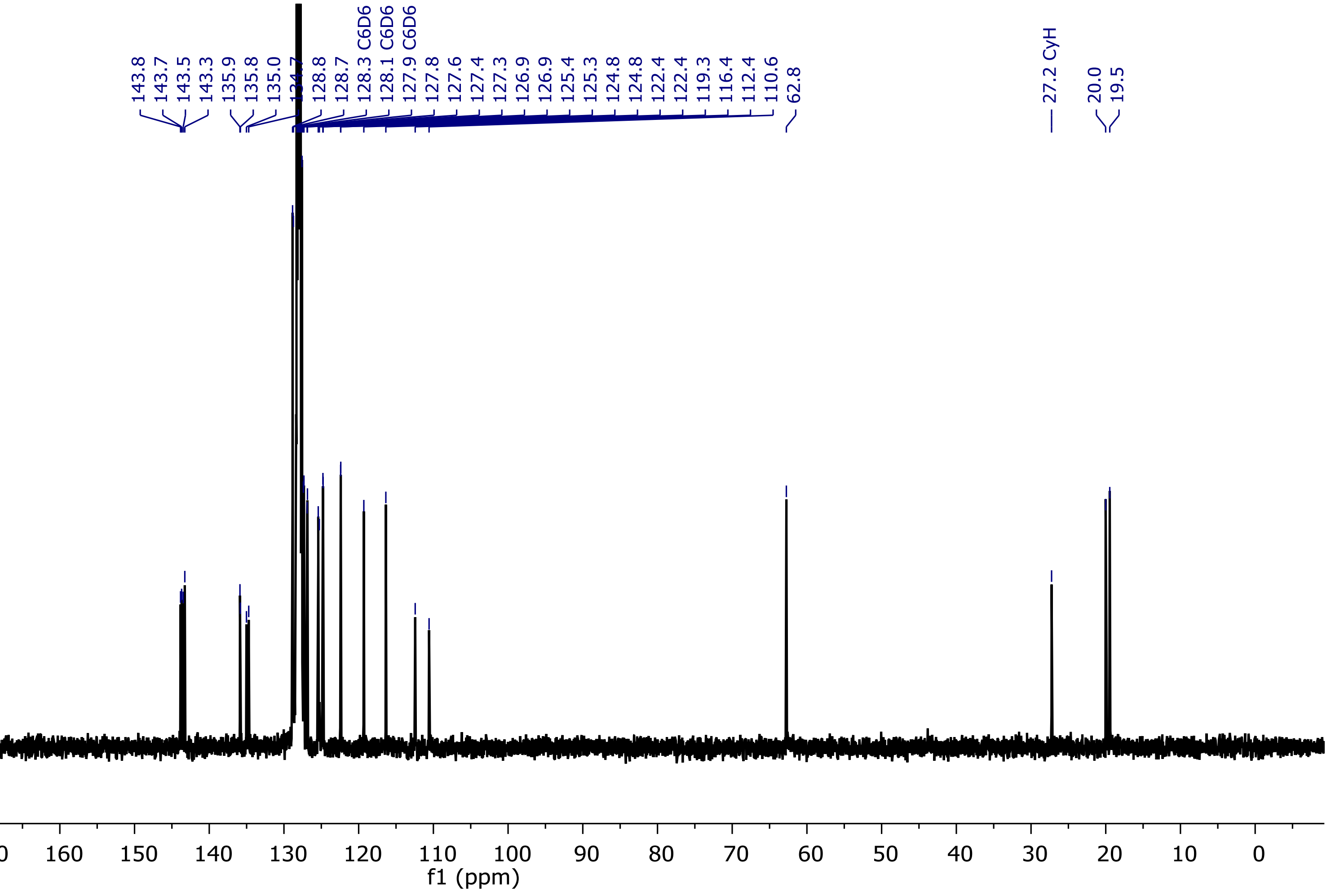

Figure S133. **^{1}H NMR** spectrum (500 MHz, C_6D_6, 298 K) of **4v**.

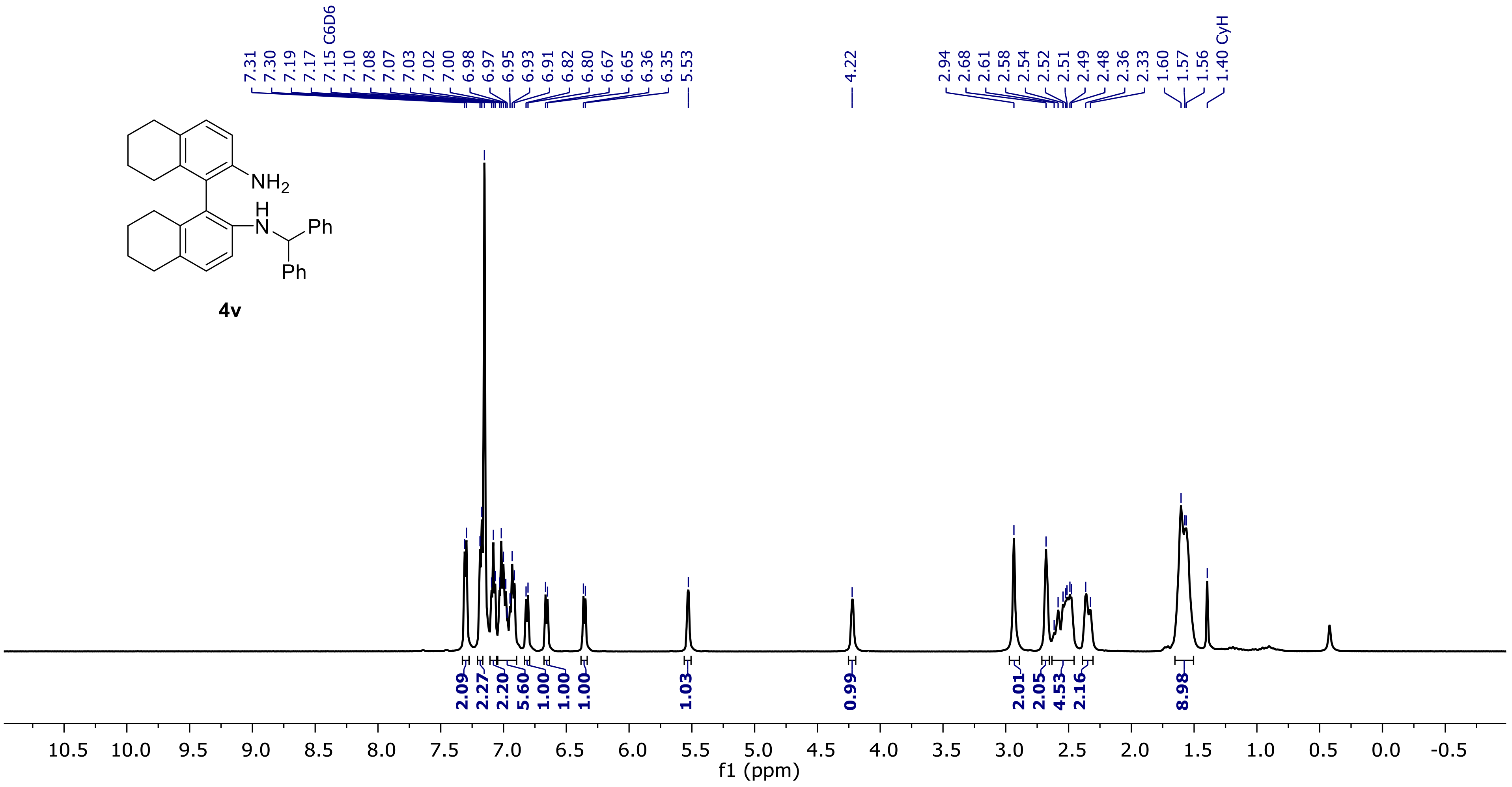

Figure S134. **$^{13}C\{^1H\}$ NMR** spectrum (126 MHz, C_6D_6, 298 K) of **4v**.

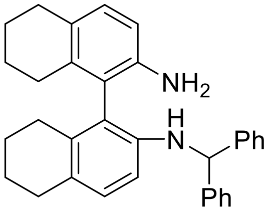

4v

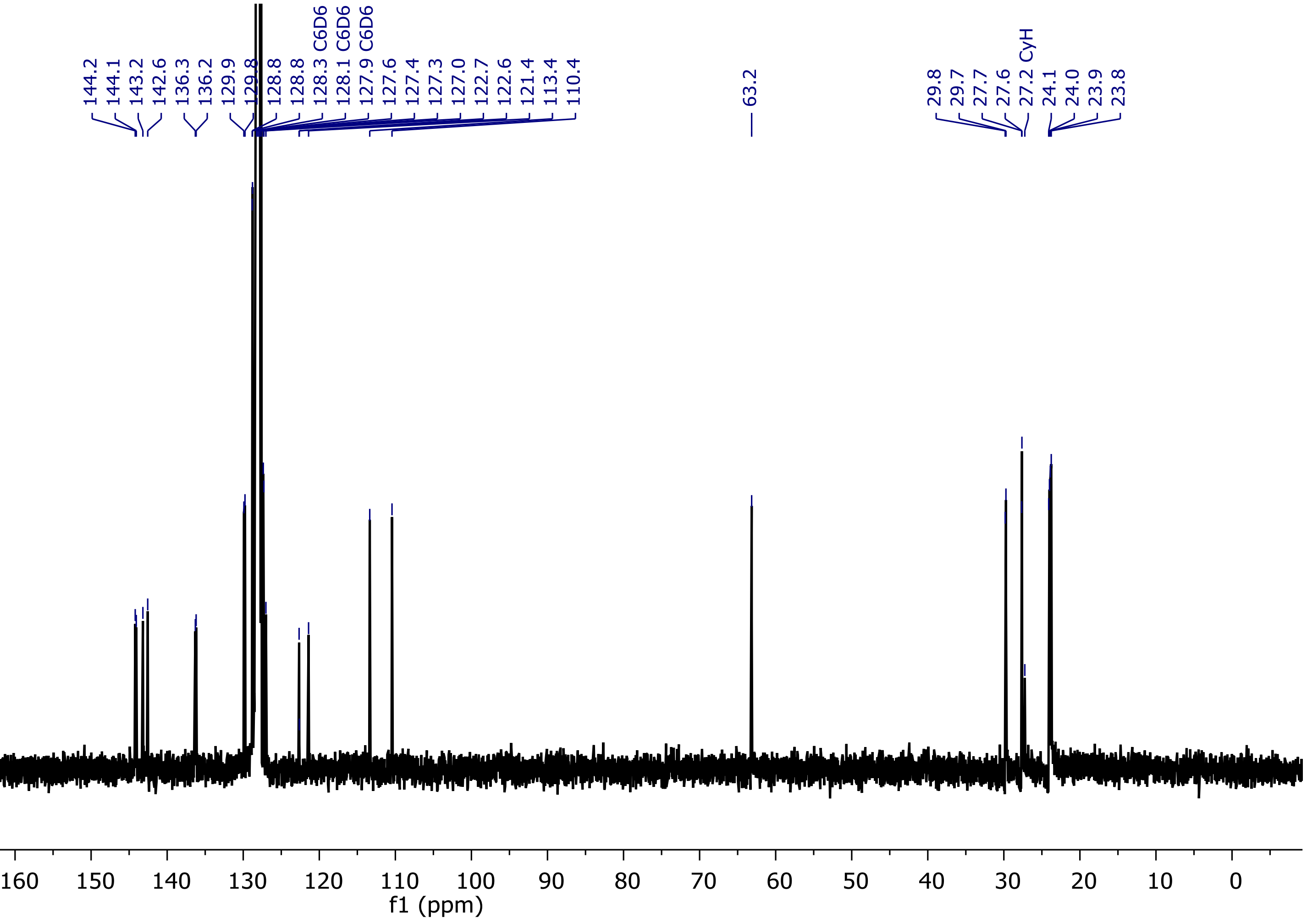

Figure S135. **^{1}H NMR** spectrum (400 MHz, Acetone-d_6, 298 K) of **S27**.

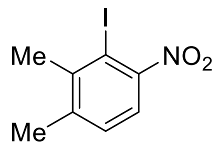

S27

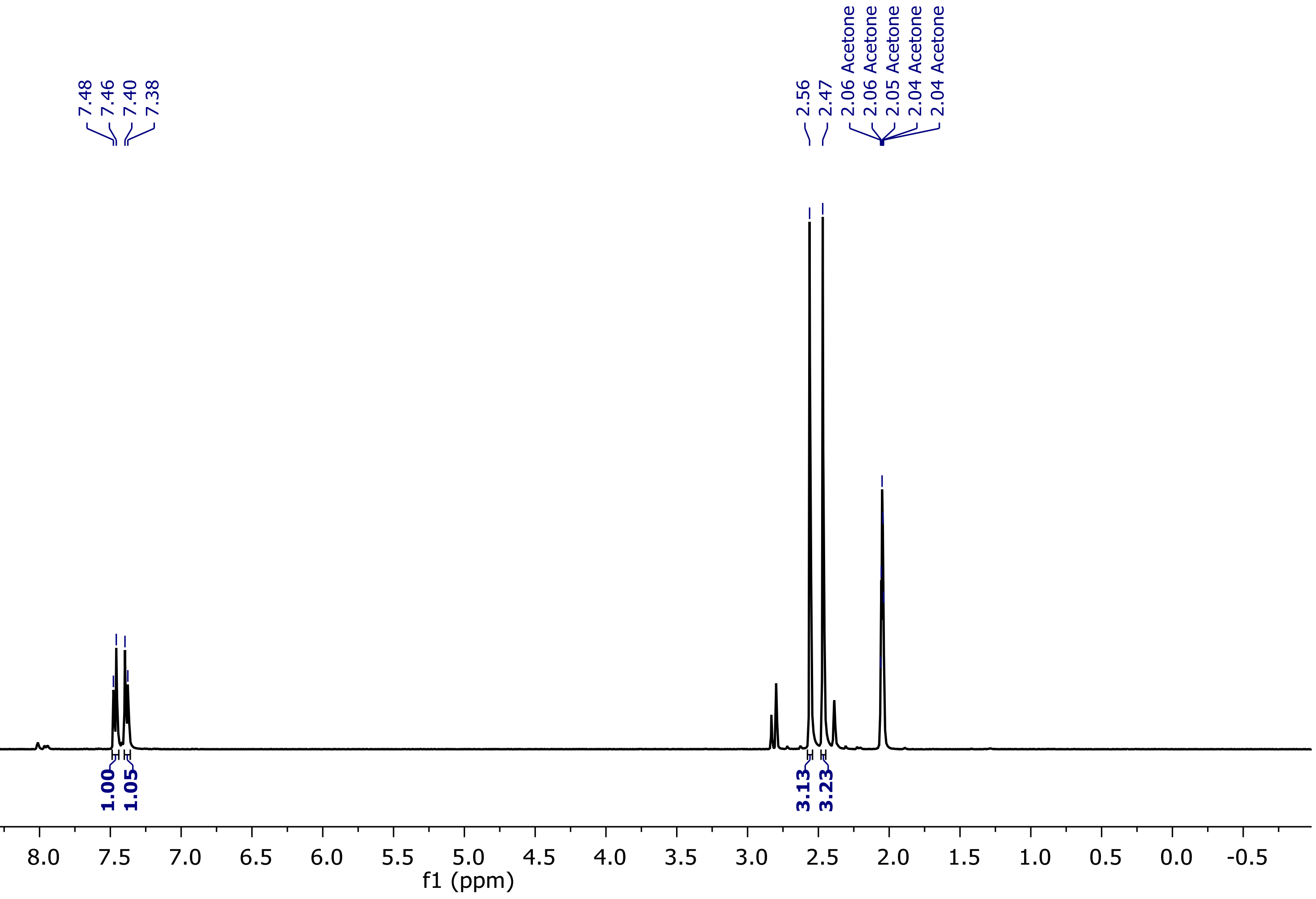

Figure S136. $^{13}C\{^1H\}$ **NMR** spectrum (101 MHz, Acetone-d_6, 298 K) of **S27**.

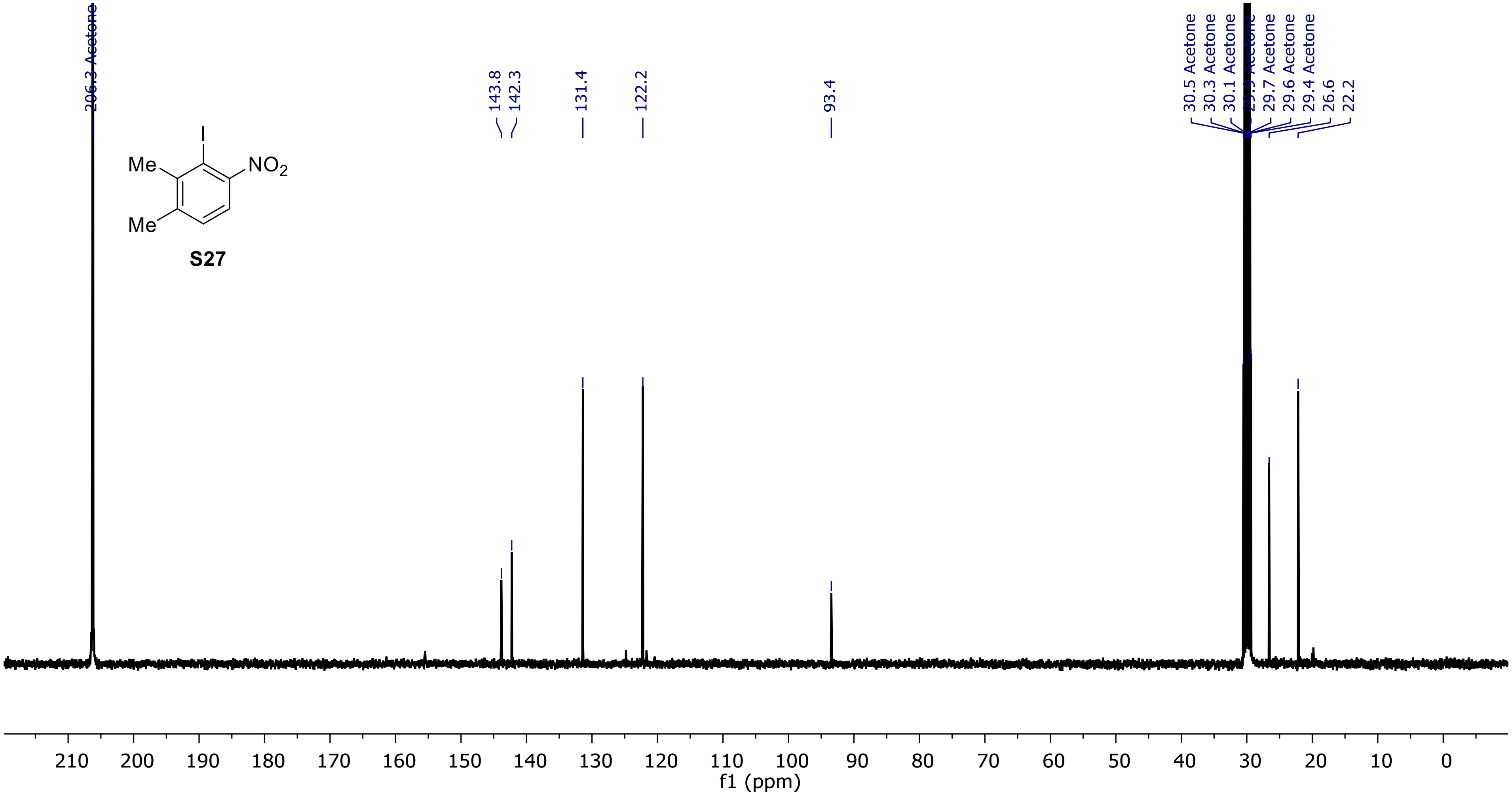

Figure S137. **^{1}H NMR** spectrum (400 MHz, Acetone-d_6, 298 K) of **S28**.

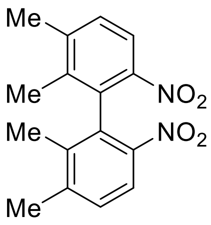

S28

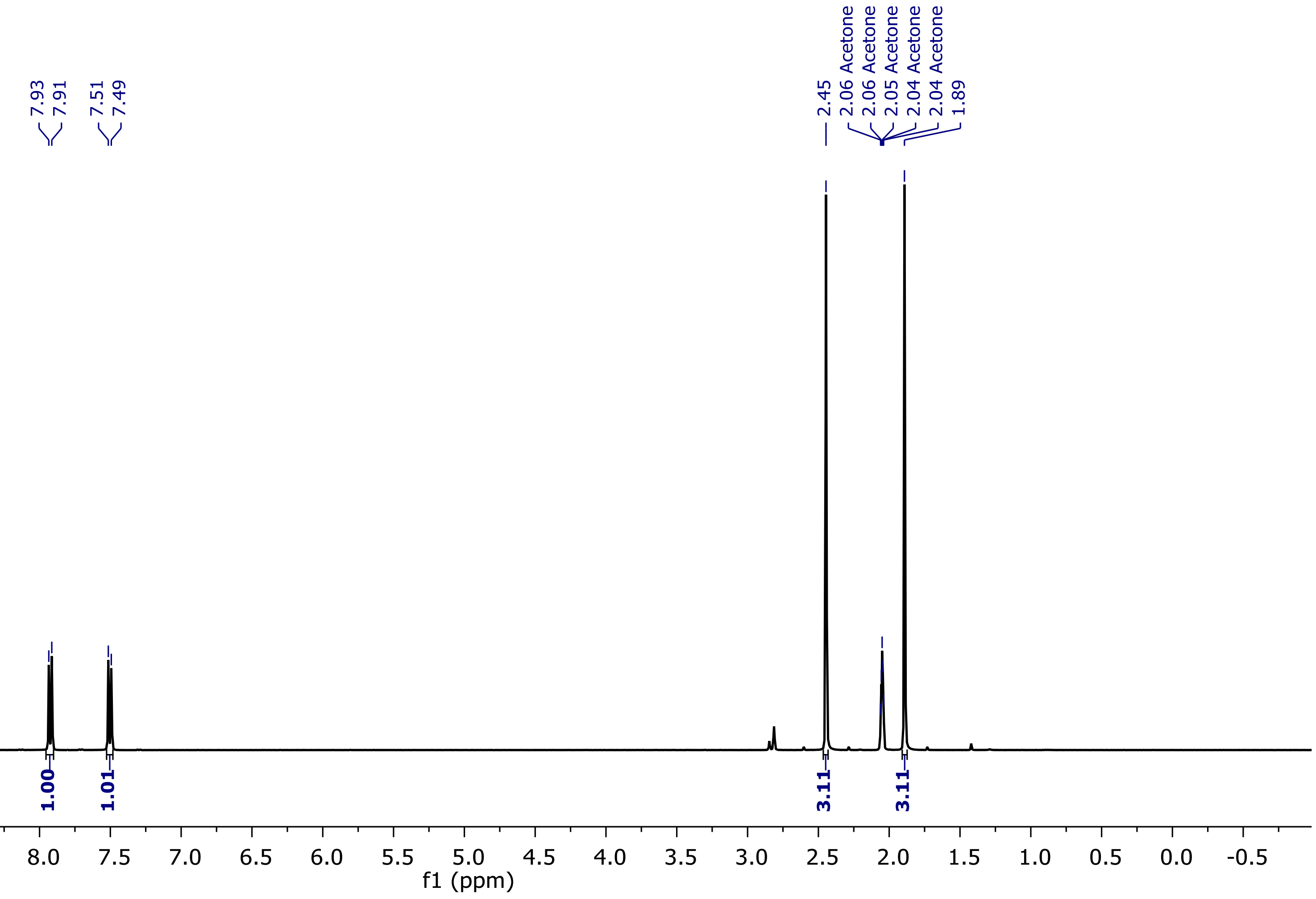

Figure S138. **$^{13}C\{^1H\}$ NMR** spectrum (101 MHz, Acetone-d_6, 298 K) of **S28**.

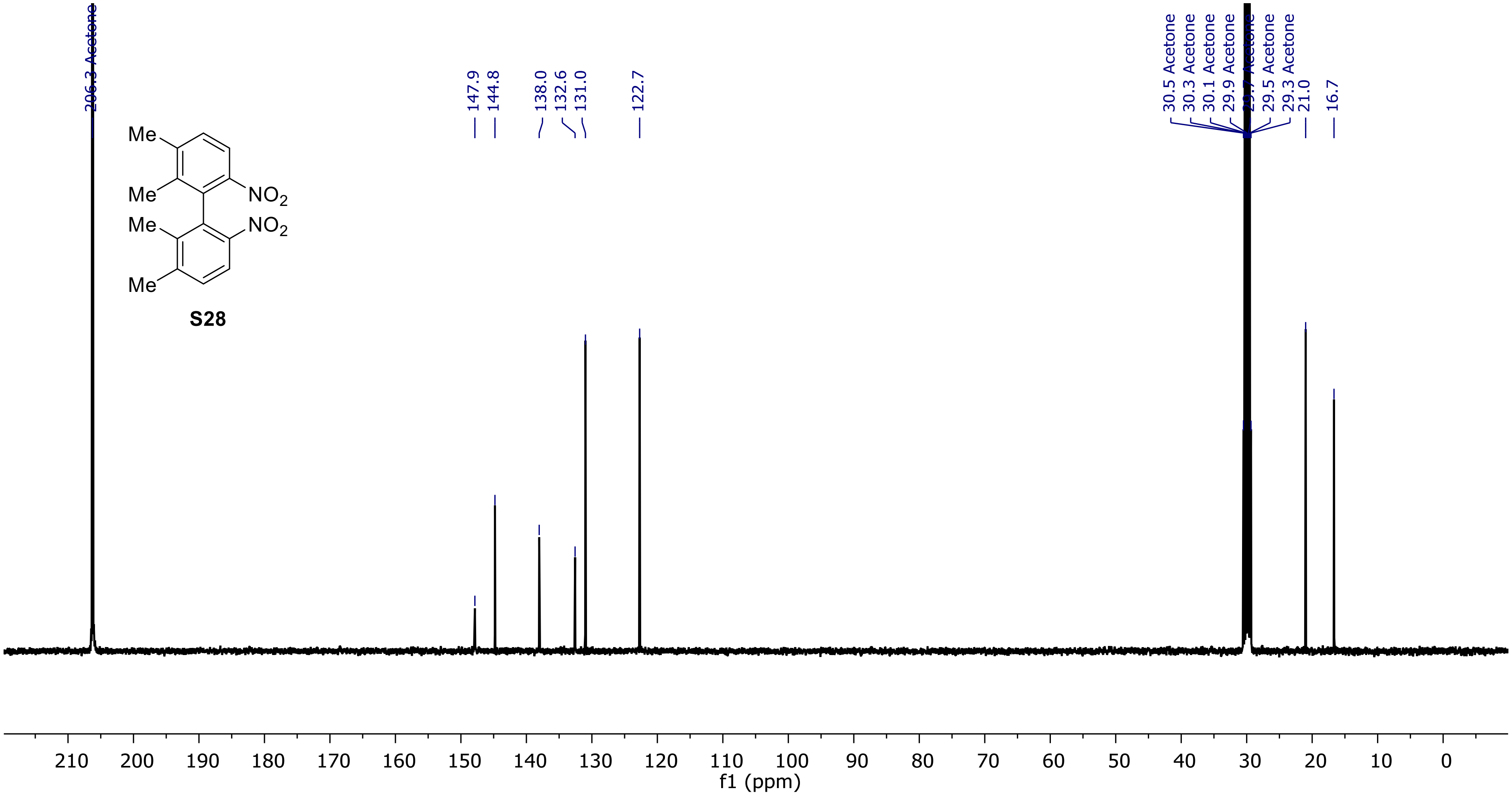

Figure S139. **¹H NMR** spectrum (500 MHz, C_6D_6, 298 K) of **4w**.

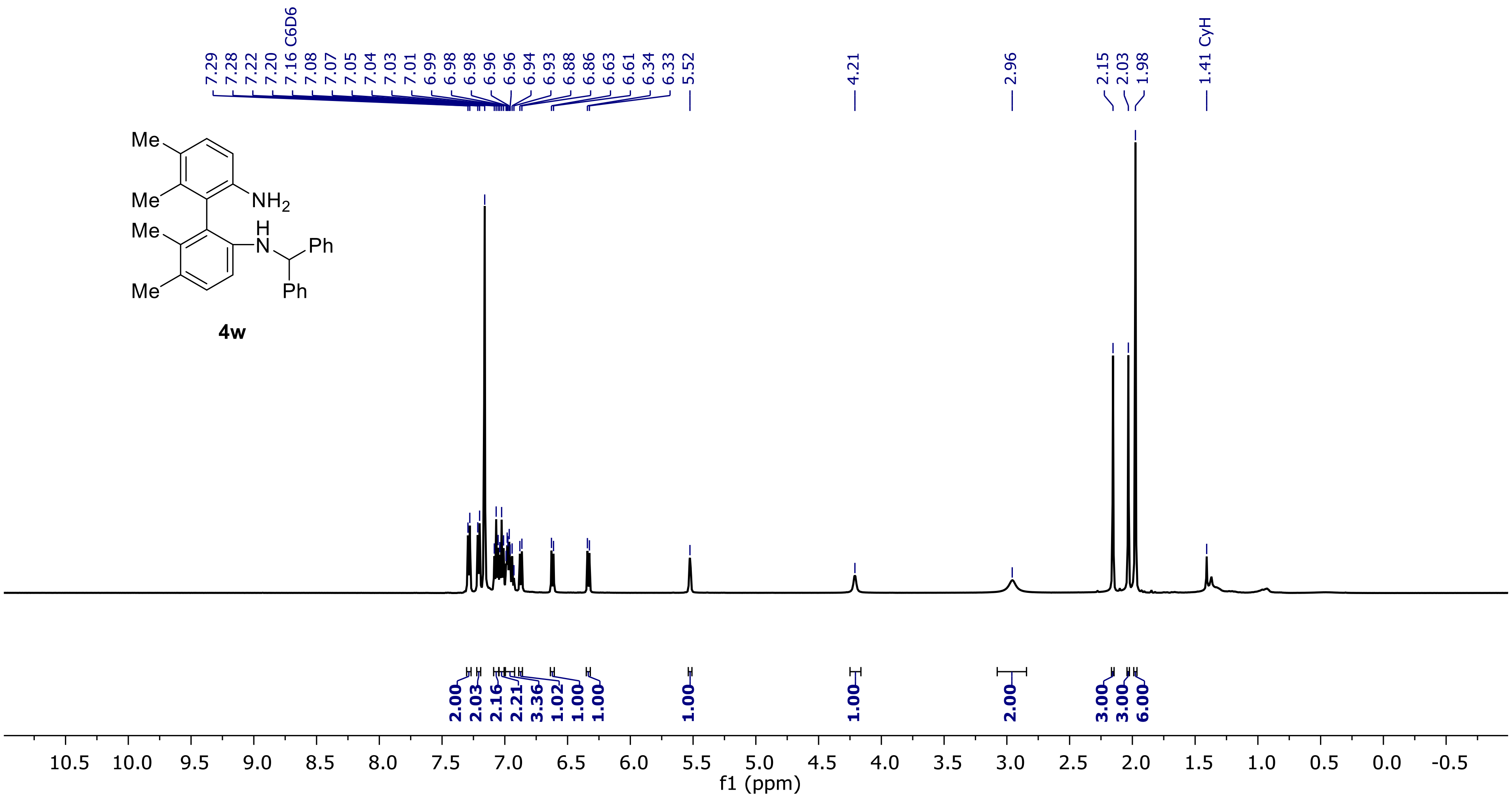

Figure S140. ^{13}**C{^{1}H} NMR** spectrum (126 MHz, C_6D_6, 298 K) of **4w**.

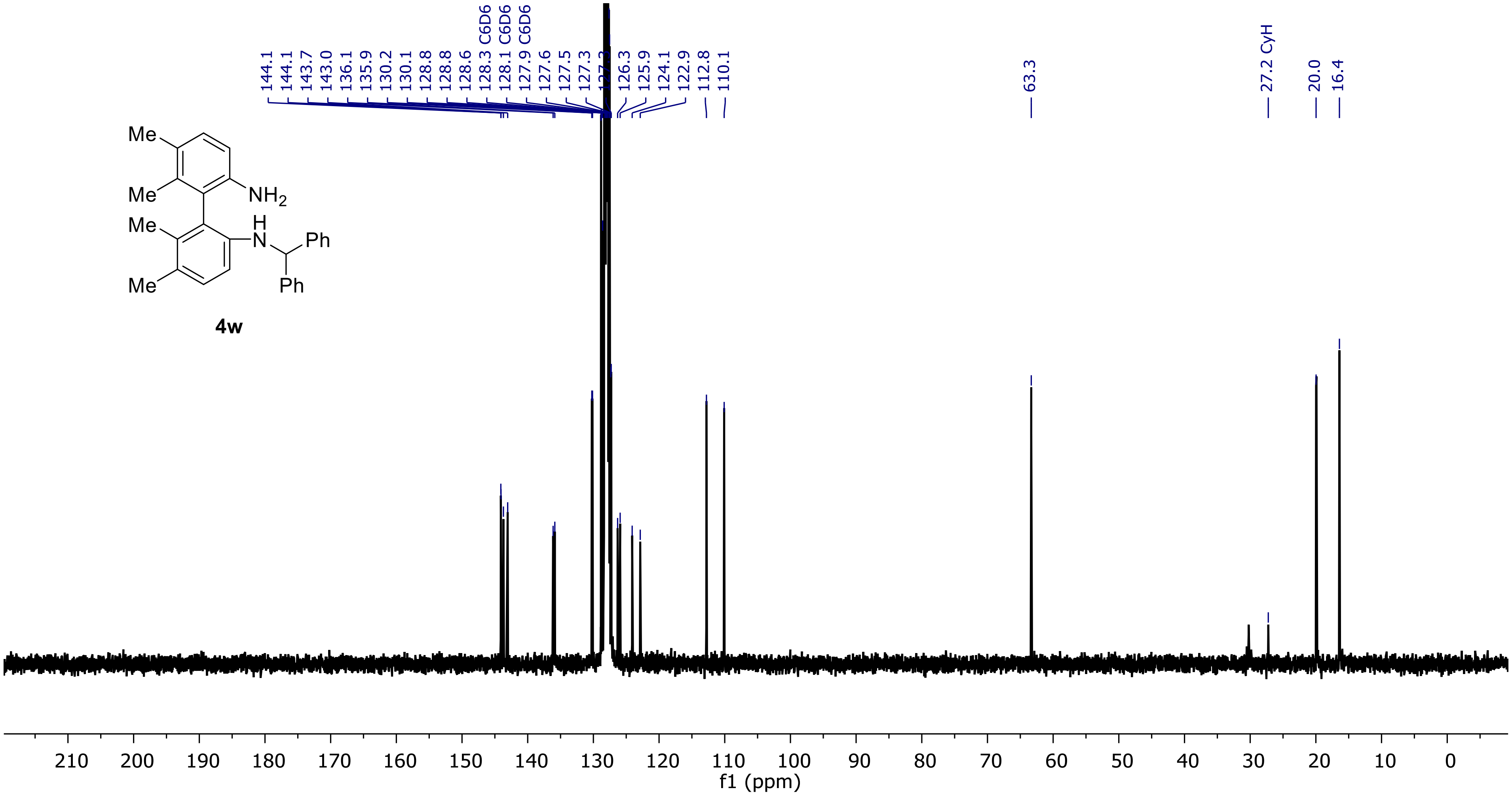

Supplement: Supplementary file 1 [file ol5c02258_si_001.pdf]
